# The Reactivity of Bispidine Ligand Based Iron(IV) and Iron(V) Oxido Species for the Demethylation of Acetic Acid

**DOI:** 10.1002/jcc.70369

**Published:** 2026-04-22

**Authors:** Gunasekaran Velmurugan, Peter Comba

**Affiliations:** ^1^ Universität Heidelberg Anorganisch‐Chemisches Institut und Interdisziplinäres Zentrum für Wissenschaftliches Rechnen Heidelberg Germany; ^2^ Department of Chemistry National Institute of Technology—Tiruchirappalli Tiruchirappalli Tamil Nadu India

## Abstract

The high valent bispidine iron‐oxido complexes LFe^IV^═O and LFe^V^═O show exceptionally high reactivity for C–H abstraction reactions. While the reactivity, electronics of the reactive species and general mechanisms have been studied in detail before, differences in reactivity of [(L)Fe^IV^═O]^2+^ and [(L)Fe^V^═O]^3+^, specifically for the demethylation of acetic acid, remained unexplored. Following experimental work of the C–H abstraction reaction by ferryl complexes of tetradentate bispidine ligands with acetic acid as substrate, this reaction was investigated with computational methods. A density functional theory (DFT) study of the iron bispidine catalyzed mechanism with hydrogen peroxide as oxidant and acetic acid as substrate and precursor of various small organic products, specifically methane, formaldehyde and formic acid, is used to analyze details of the reaction mechanism and specifically also to investigate the influence of different oxidation states of iron on this reaction. Therefore, two pathways, catalyzed by bispidine based [(L)Fe^IV^═O]^2+^ and [(L)Fe^V^═O]^3+^ species with acetic acid were considered. The computational analysis confirms that the rate‐determining step of the demethylation is C–H abstraction at C2 of acetic acid, leading to a radical intermediate that leads to the various products. The [(L)Fe^V^═O]^3+^ catalyzed reaction is found to be more efficient than that based on [(L)Fe^IV^═O]^2+^. Reasons for the different reactivities have been explored by the WB index, EDA and an NBO analysis.

## Graphical abstract

In agreement with experiment, DFT shows that high‐valent iron bispidines readily react with acetic acid by HAA (hydrogen atom abstraction) to produce C1 products in various oxidation states.

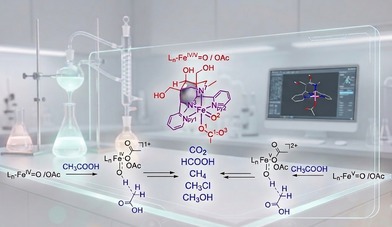

## 1 Introduction

In our environment organic matter undergoes numerous chemical reactions. Some yield compounds of relevance for the atmosphere, such as CO_2_, CH_4_, and small halogenated alkanes, generally referred to as volatile organic compounds (VOCs) [1]. Due to global warming these are a focus in environmental sciences, and the increasing amounts of VOCs in the atmosphere also harm human health, especially in children, where they cause respiratory allergic and immune effects [2]. Reactions with other components in the atmosphere can lead to the formation of further oxidation products and secondary aerosols, which also can lead to sensory irritation symptoms [3]. It is known that there is natural (biotic and abiotic) production of VOCs and in particular also of halogenated species, and this generally is the result of high‐valent iron‐oxido chemistry [4, 5, 6]. We have been interested in the iron/H_2_O_2_ chemistry with iron salts in aqueous solution and supported by amine/pyridine ligands, specifically with bispidine ligand systems since these have allowed us to thoroughly study the corresponding pathways mechanistically [7, 8, 9, 10].

**SCHEME 1:**
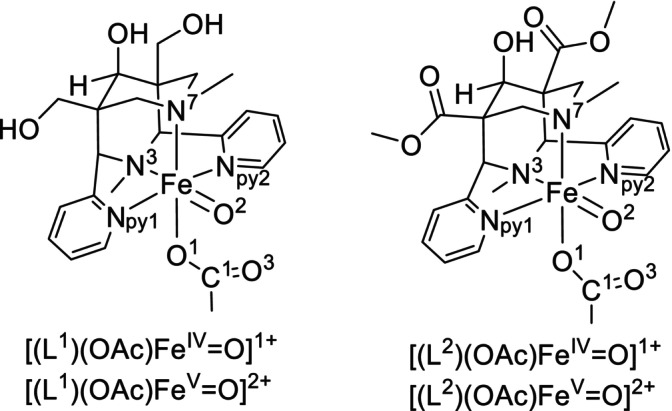
Structures of the high‐valent iron‐oxido complexes [(L^1,2^)(OAc)Fe^IV^═O]^1+^ and [(L^1,2^)(OAc)Fe^V^═O]^2+^.

It is known that C1 compounds such as CO_2_ and CH_4_ originate from aerobic systems like fungi [11], plants [12], algae [13], and humans [11] as well as animals [14, 15]. Apart from biotic systems that show formation of C1 compounds, in sediments and hypersaline soils there also is formation of organohalogen species such as chloromethane. However, the mechanism of methane formation under aerobic as well as anaerobic conditions in the environment remains at least partially unclear [4, 16], that is, for in vivo as well as in vitro methanogenesis the underlying mechanisms and precursor molecules are largely unknown. One possible precursor for C1 compounds in various oxidation states is acetic acid: it is present in biotic systems as well as in the soil, in sediments and the atmosphere, often produced by actobacter genus, a class of bacteria found commonly in soils and water [17]. Recently, an experimental study has shown that the iron catalyzed oxidation of acetic acid indeed yields C1 compounds, including methane, chloromethane, methanol and formaldehyde, and also has lead to preliminary ideas for probable mechanistic pathways [18]. These studies were based on tetra‐ and pentadentate bispidine ligands (see Scheme 1 for the structures of the bispidine‐iron(IV)‐oxido complexes discussed here), where a large body of experimental and computational mechanistic work is available, specifically also for C–H activation reactions [10, 19, 20, 21, 22, 23, 24, 25, 26, 27].

High‐valent nonheme iron complexes have been studied extensively in C–H activation [28, 29, 30]. An interesting feature in the context of the present communication is that acetate has been found to enhance the reactivity of ferryl groups [31, 32]. Experimental and computational mechanistic studies indicate that the active species is based on an equilibrium between Fe^III^–OOAc and Fe^V^═O [33, 34]. Therefore, acetate in the systems studied here may be both an additive involved in the formation of the catalytically active species as well as the substrate, and pathways on both Fe^IV^═O and Fe^V^═O are considered.

**SCHEME 2:**
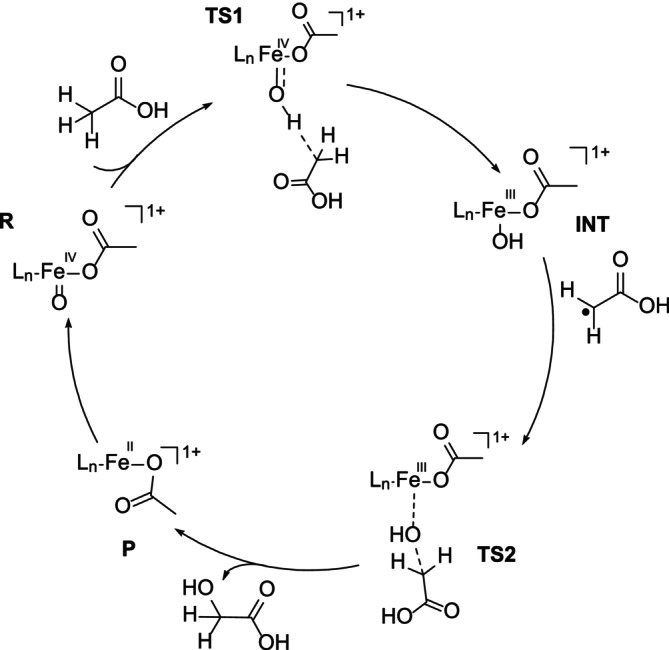
Proposed mechanism of the LFe^IV^═O‐catalyzed oxidation of acetic acid.

Here, we describe a computational study on intricate mechanistic details of the oxidative demethylation of acetic acid with [(L^1,2^)(OAc)Fe^IV^═O]^1+^ and [(L^1,2^)(OAc)Fe^V^═O]^2+^ (Scheme 1). From experiment it is known that C–H abstraction at C2 of acetic acid is the rate‐determining step [18], and the radical intermediate is known to form a range of products, including CH_4_ and CO_2_ as well as other possible C1 compounds with intermediate oxidation numbers and also halogenated species. For simplicity, we concentrate here on the rebound product, that is, glycolic acid, see Scheme 2 for the Fe^IV^═O based mechanism derived from the experimental studies (more detailed mechanistic schemes, also based on Fe^V^═O, are given in Supporting Information, Schemes S1 and S2) [18]. Probing the structure and bonding of [(L^1,2^)(OAc)Fe^IV^═O]^1+^ and [(L^1,2^)(OAc)Fe^V^═O]^2+^ and the corresponding transition states (TS) may help to analyze the reactivity of the high‐valent iron‐oxido species involved in product formation, and the corresponding questions of interest include: (i) What is the ground state electronic structure of [(L^1,2^)(OAc)Fe^IV^═O]^1+^ and [(L^1,2^)(OAc)Fe^V^═O]^2+^ and how do they differ? (ii) What electronic properties are responsible for the preferred pathway of the demethylation of CH_3_COOH? (iii) How important is the spin‐state in controlling the reactivity? (iv) Which oxidant is more reactive [(L^1,2^)(OAc)Fe^IV^═O]^1+^ or [(L^1,2^)(OAc)Fe^V^═O]^2+^, and why?

Although the oxidative demethylation of acetic acid appears chemically simple, it involves a complex interplay between electronic structure, spin state, and reaction geometry. Both intramolecular and intermolecular hydrogen abstraction mechanisms were evaluated in this study to account for the potential influence of substrate orientation and pre‐reaction interactions. Furthermore, the effect of hydrogen bonding between acetic acid and the carboxylate oxygen of the complex was examined since such interactions can in principle modulate C–H activation efficiency.

## 2 Computational Details

All DFT calculations were performed using the Gaussian 16 suite of programs [35]. The geometries were optimized using the B3LYP‐D3 functional with the LACVP basis set comprising the LanL2DZ‐Los Alamos effective core potential for Fe [36, 37, 38] and a 6‐31g(d) basis set [39] for the other atoms (carbon, nitrogen, oxygen, and hydrogen, B‐I) [40]. Single point calculations were performed using a def2‐TZVP basis set [41, 42, 43] (B‐II) on the optimized geometries. This kind of computational approach has been used before for several computational studies involving mononuclear metal complexes [10, 44, 45, 46, 47, 48, 49, 50, 51, 52]. Frequency calculations on the optimized structures were used to confirm minima on the potential energy surface (PES) and also to obtain free energy and zero‐point energy corrections. TS were characterized by a single negative frequency. Intrinsic reaction coordinate (IRC) calculations were carried out to confirm that the computed TS are connecting to the corresponding intermediates. Solvation energies were computed by using the polarizable continuum solvation model (PCM), where water was used as solvent. Unless otherwise mentioned, all reported energies are B3LYP‐D3 solvent‐phase energies, incorporating free energy corrections at the B‐I level at 298.15 K. Geometry optimizations were also performed including solvation effects using the PCM implicit solvent model for water, and the computed energetic trends remain similar. Therefore, in general we have only performed single point calculations for the computed solvation effects with the PCM model. Wiberg bond (WB) indices were computed from the natural atomic orbital analysis (NBO), and bond formation indices (BF_*i*_) or bond cleavage indices (BC_*j*_) were obtained from the calculated bond orders, following the Equation (1):

(1) $${\mathrm{BF}}_{i}\;\text{or}\;{\mathrm{BC}}_{j}\;=\;\frac{{\mathrm{BO}}_{i}^{\mathrm{TS}}-{\mathrm{BO}}_{i}^{R}}{{\mathrm{BO}}_{i}^{P}-{\mathrm{BO}}_{i}^{R}}\times 100$$

From the calculated BF_*i*_ and BC_*j*_ values, bond formation‐cleavage average values (BFC_Ave_) were calculated according to Equation (2) [53]:

(2) $${\mathrm{BFC}}_{\mathrm{Ave}}=½\;\left({\mathrm{BF}}_{i}+{\mathrm{BC}}_{f}\right)$$

Additionally, energy decomposition analysis calculations (EDA‐AOM) for the first TS, that is, the C–H abstraction, were performed [54]. It is worth noting that density functional methods can occasionally predict an incorrect ordering of energetically close spin states in high‐valent iron–oxido systems. To obtain a more reliable description, DLPNO‐CCSD(T) single‐point calculations were carried out on the B3LYP‐D3 optimized geometries. These high‐level correlated calculations refined the spin‐state energetics and reproduced ground states that are consistent with experiment [55, 56, 57]. The suitability of the DLPNO‐CCSD(T) approach for bispidine‐based iron–oxo complexes has been demonstrated previously, further supporting the reliability of this methodology for the present study [58]. All possible spin states of the complexes were refined with the ORCA 4.2 suite [59, 60]. The description of intermediate‐ and low‐spin Fe^IV^–oxido species can be affected by the multireference character of the electronics [61]. To assess the reliability of the present calculations, ⟨*S* ^2^⟩ values and DLPNO‐CCSD(T) T_1_ diagnostics were done and are provided in Supporting Information. The values indicate only minor spin contamination and an acceptable single‐reference description, supporting the consistency of the computed energetics. The states are generally labeled as ^*x*^ *Y*_Z_ where *x* is the spin multiplicity, *Y* corresponds to the designation of a reactant (R), TS, or product (P), while *Z* shows whether the state at hand possesses high spin (HS), intermediate spin (IS), or low spin (LS) electronic configuration.

## 3 Results and Discussion

### 3.1 Electronic Structure of [(L^1^ ^,2^)(OAc)Fe^IV^ ═O]^1+^ and [(L^1^ ^,2^)(OAc)Fe^V^ ═O]^2+^

**FIGURE 1:**
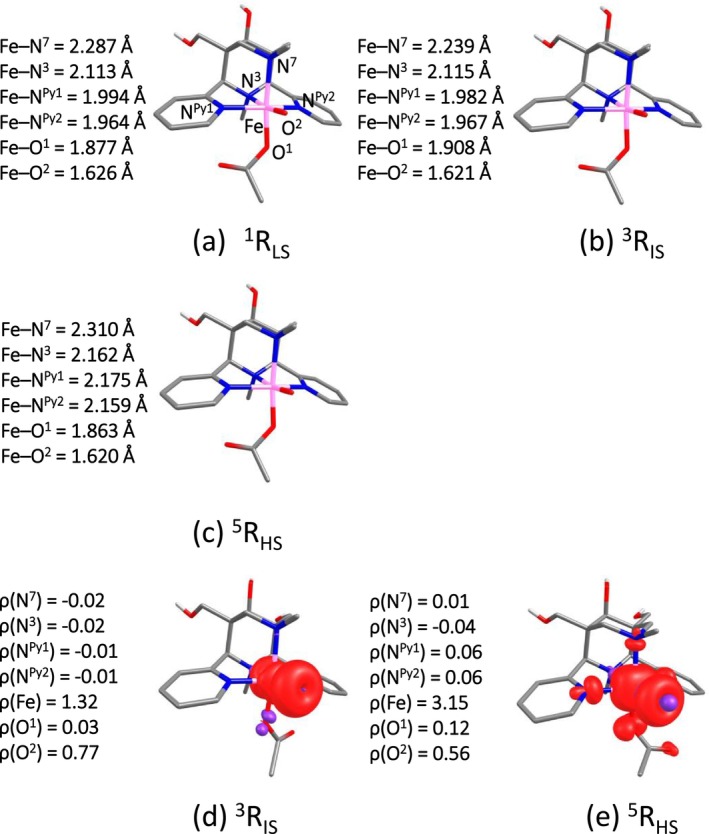
Optimized geometries (a–c) and spin density plots (d, e) for O *trans* N^3^‐[(L^1^)Fe^IV^═O]^1+^ complex.

The [(L^1,2^)(OAc)Fe^IV^═O]^1+^ oxidants can have either HS (*S* = 2), IS (*S* = 1), or LS (*S* = 0) electronic configuration, that is, ^5^R_HS_, ^3^R_IS_, and ^1^R_LS_. Among the three states computed for the more stable isomer [(L^1^)(OAc)Fe^IV^═O]^1+^ with the oxido group *trans* to N^3^ (O *trans* N^3^, see below) ^5^R_HS_ is found to be the ground state followed by ^3^R_IS_ by an energy margin of 14.2 kJ/mol, and ^1^R_LS_ found to lie 144.5 kJ/mol higher in energy (Table 1; structural data are given in Figure 1). The inclusion of solvent does not lead to any change in trend with respect to the gas‐phase calculation. The spin density values of the ^5^R_HS_ state are 3.15 for Fe and 0.56 for O, suggesting that [(L^1^)(OAc)Fe^IV^═O]^1+^ has a considerable amount of Fe^III^–O^●^ character (Figure 1), with a significant reduction of the spin density at Fe and some delocalization to the coordinated N atoms. The spin density values of the ^3^R_IS_ state are 2.31 for Fe and 0.77 for the oxido O. There is no significant difference in the Fe–O distance (1.620–1.626 Å) between the different spin states (see Figure 1). However, we have noticed a marginal difference in the Fe–N^7^ bond length (2.29, 2.24, and 2.31 Å, respectively, for ^1^R_LS_, ^3^R_IS_, and ^5^R_HS_), Fe–N^3^ (2.11, 2.11, and 2.16 Å, respectively, for ^1^R_LS_, ^3^R_IS_, and ^5^R_HS_), Fe–N^py1^ (1.99, 1.98, and 2.17 Å, respectively, for ^1^R_LS_, ^3^R_IS_, and ^5^R_HS_), and Fe–N^py2^ (1.96, 1.97, and 2.16 Å, respectively, for ^1^R_LS_, ^3^R_IS_, and ^5^R_HS_). Similar trends are obtained for [(L^2^)(OAc)Fe^IV^═O]^1+^ (see Figure 1). The comparison with experimental Fe–O bond lengths of similar systems, obtained from crystallography and x‐ray absorption spectroscopy supports the computational accuracy of our models. The calculated Fe ═ O distances (1.62 Å for Fe(IV) ═ O and 1.68 Å for Fe(V) ═ O) closely match experimentally observed ranges for related non‐heme iron‐oxido complexes [62].

**TABLE 1:** Computed optimized energies of spin states of [(L^1,2^)(OAc)Fe^IV^═O]^1+^ and [(L^1,2^)(OAc)Fe^V^═O]^2+^.

Spin state		Energy (kJ/mol)		
		Gaussian		ORCA
		UB3LYP/def2‐TZVP (gas)	UB3LYP/def2‐TZVP (solvent)	DLPNO‐CCSD(T)/cc‐PVQZ//UB3LYP/def2‐TZVP
[(L^1^)Fe^IV^═O]^1+^ (O *trans* N^3^)	^5^R_HS_	0.0	0.0	45.9
	^3^R_IS_	12.8	14.2	0.0
	^1^R_LS_	161.5	144.5	180.4
[(L^1^)Fe^IV^═O]^1+^ (O *trans* N^7^)	^5^R_HS_	0.0	0.0	39.8
	^3^R_IS_	18.2	22.9	0.0
	^1^R_LS_	115.7	127.9	120.7
[(L^2^)Fe^IV^═O]^1+^ (O *trans* N^3^)	^5^R_HS_	0.0	0.0	48.1
	^3^R_IS_	13.2	12.9	0.0
	^1^R_LS_	124.7	139.5	184.1
[(L^2^)Fe^IV^═O]^1+^ (O *trans* N^7^)	^5^R_HS_	0.0	0.0	49.5
	^3^R_IS_	19.5	21.2	0.0
	^1^R_LS_	117.1	127.7	123.8
[(L^1^)Fe^V^═O]^2+^ (O *trans* N^3^)	^4^R_HS_	0.0	0.0	0.0
	^2^R_LS_	95.8	99.0	159.0
[(L^1^)Fe^V^═O]^2+^ (O *trans* N^7^)	^4^R_HS_	0.0	0.0	0.0
	^2^R_LS_	42.7	58.4	167.2
[(L^2^)Fe^V^═O]^2+^ (O *trans* N^3^)	^4^R_HS_	0.00	0.00	0.0
	^2^R_LS_	94.5	96.4	121.8
[(L^2^)Fe^V^═O]^2+^ (O *trans* N^3^)	^4^R_HS_	0.00	0.00	0.0
	^2^R_LS_	73.1	85.6	128.1

The other possible isomer has the oxido group swapped with the acetate. This O *trans* N^7^ isomer (see Supporting Information for the computed structures, Figures S15–S26) is known from various DFT calculations to be less stable than the O *trans* to N^3^ isomer generally discussed here (the margin of the current calculations is 10.01 kJ/mol) [9, 10, 23, 63], and this has recently also been confirmed by experiment [64]. There is only a marginal difference of the computed Fe^IV^═O bond lengths of about 0.002 Å but the O *trans* to N^3^ isomer has a longer bond to the acetate (0.029 Å) and the N^7^ (0.042 Å), while the Fe–N^3^ bond is considerably shorter (0.108 Å). For the results of O trans to N^7^ we refer to Supporting Information. The predicted high‐spin ground state of [(L^1,2^)(OAc)Fe^IV^═O]^1+^ is in contrast to the experimental observation [25, 26], and we therefore have also computed all spin state energies with DLPNO‐CCSD(T); the results suggest that ^3^R_IS_ is the ground state, followed by ^5^R_HS_ at 45.9 kJ/mol (see Table 1), correcting the DFT‐predicted ordering. This reordering reflects improved dynamic correlation treatment at the coupled‐cluster level and supports the experimentally observed ground‐state.

**FIGURE 2:**
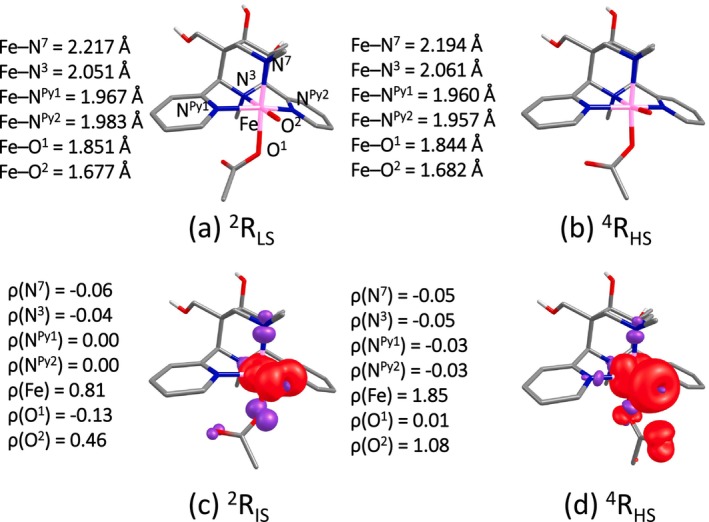
Optimized geometries (a, b) and spin density plots (c, d) for O‐trans N^3^‐[(L^1^)(OAc)Fe^V^═O]^2+^.

The higher valent [(L^1,2^)(OAc)Fe^V^═O]^2+^ species can be either HS (*S* = 3/2) or LS (*S* = 1/2), denoted as ^4^R_HS_ and ^2^R_LS_, respectively. Among the two spins states computed for [(L^1^)(OAc)Fe^V^═O]^2+^, ^4^R_HS_ is found to be the ground state, followed by ^2^R_IS_ with an energy margin of 99.0 kJ/mol (Table 1, see Figure 2 for structural data). The spin density values of the ^4^R_HS_ state are 1.85 for Fe and 1.08 for O, suggesting that [(L^1^)(OAc)Fe^V^═O]^2+^ has significant Fe^IV^–O^●^ character (see Figure 2), and the expected spin density at Fe is significantly reduced, indicating that some of the spin density is delocalized to the coordinated N donors. The computed spin densities of the ^2^R_LS_ state are 0.81 for Fe and 0.46 for O. There is no difference in the Fe–O bond length between the different spin states (1.682 vs. 1.677 Å). However, there are small difference for the other Fe‐donor distances, that is, Fe–N^7^ (2.22 vs. 2.19 Å for ^2^R_LS_ and ^4^R_HS_), Fe–N^3^ (2.05 vs. 206 Å), Fe–N^py1^ (1.97 vs. 1.96 Å) and N^py2^ (1.98 vs. 1.96 Å). Similar results are observed for [(L^2^)(OAc)Fe^V^═O]^2+^ (see Figure S11). Here also, the O *trans* N^3^ isomer is more stable than O *trans* to N^7^ by an energy margin of 10.01 kJ/mol, and all species discussed therefore corresponds to O *trans* N^3^ (see Supporting Information for data on the other isomer). Both DFT and DLPNO‐CCSD(T) show that ^4^R_HS_ is the ground state with ^2^R_LS_ at 99.0 (159.0) kJ/mol.

A slightly higher value of the WB index for Fe^IV^═O (1.563) than for Fe^V^═O (1.540) reflects a higher double bond character in the Fe^IV^═O bond. The computed spin densities on Fe and O in Fe^IV^═O are 3.15 and 0.56; for Fe^V^═O, it is 1.85 and 1.08. The increased spin density at the oxido oxygen is accompanied by a drastic reduction of spin density at the Fe center by 0.52 electrons (see Figure 2), suggesting a significant change from double bond oxido to oxyl radical character at the O atom. The NBO analysis reveals that the σ bonding interaction in Fe^IV^═O is composed of 29.6% Fe(d_z2_) and 70.4% from O(p_z_), while in Fe^V^═O it is 36.1% Fe(d_z2_) and 63.9% from O(p_z_) (see Table 2). These contributions suggest a higher degree of covalency in the Fe^V^═O than the Fe^IV^═O bond, and this also emerges from an EDA, showing that the Fe^IV^═O bond is stronger than the Fe^V^═O bond, and the stabilization essentially arises from the orbital stabilization (see Table 2). It is worth noting that, in contrast to some previously reported Fe(V) ═ O(carboxylate) systems with S = 1/2 ground state and weak Fe ═ O···O(carbonyl) interactions, our bispidine complexes favor an S = 3/2 configuration and do not exhibit such contacts [65, 66]. This difference primarily arises from the rigid coordination geometry of the bispidine ligand, which restricts bending at the Fe–O–C axis and stabilizes the high‐spin configuration. Therefore, the ligand architecture, rather than the computational approach, dictates the observed electronic structure.

**TABLE 2:** Computed NBO, EDA (kJ/mol), and WB index for [(L^1,2^)(OAc)Fe^IV^═O]^1+^ and [(L^1,2^)(OAc)Fe^V^═O]^2+^.

Species	NBO		EDA			WB index
			LFe + O			
	Metal (%)	O (%)	*E*_steric_	*E*_int_	*E*_orb_	
[(L^1^)Fe^IV^═O]^1+^	29.6	70.4	−1916.7	972.9	−943.8	1.563
[(L^2^)Fe^IV^═O]^1+^	28.9	71.1	−1921.1	955.4	−965.7	1.561
[(L^1^)Fe^V^═O]^2+^	36.1	63.9	−1850.3	939.0	−911.3	1.540
[(L^2^)Fe^V^═O]^2+^	34.0	66.0	−1871.9	926.5	−945.4	1.538

### 3.2 Initial Step of the Oxidative Demethylation of Acetic Acid

#### 3.2.1 The Fe^IV^ ═O Pathway

**FIGURE 3:**
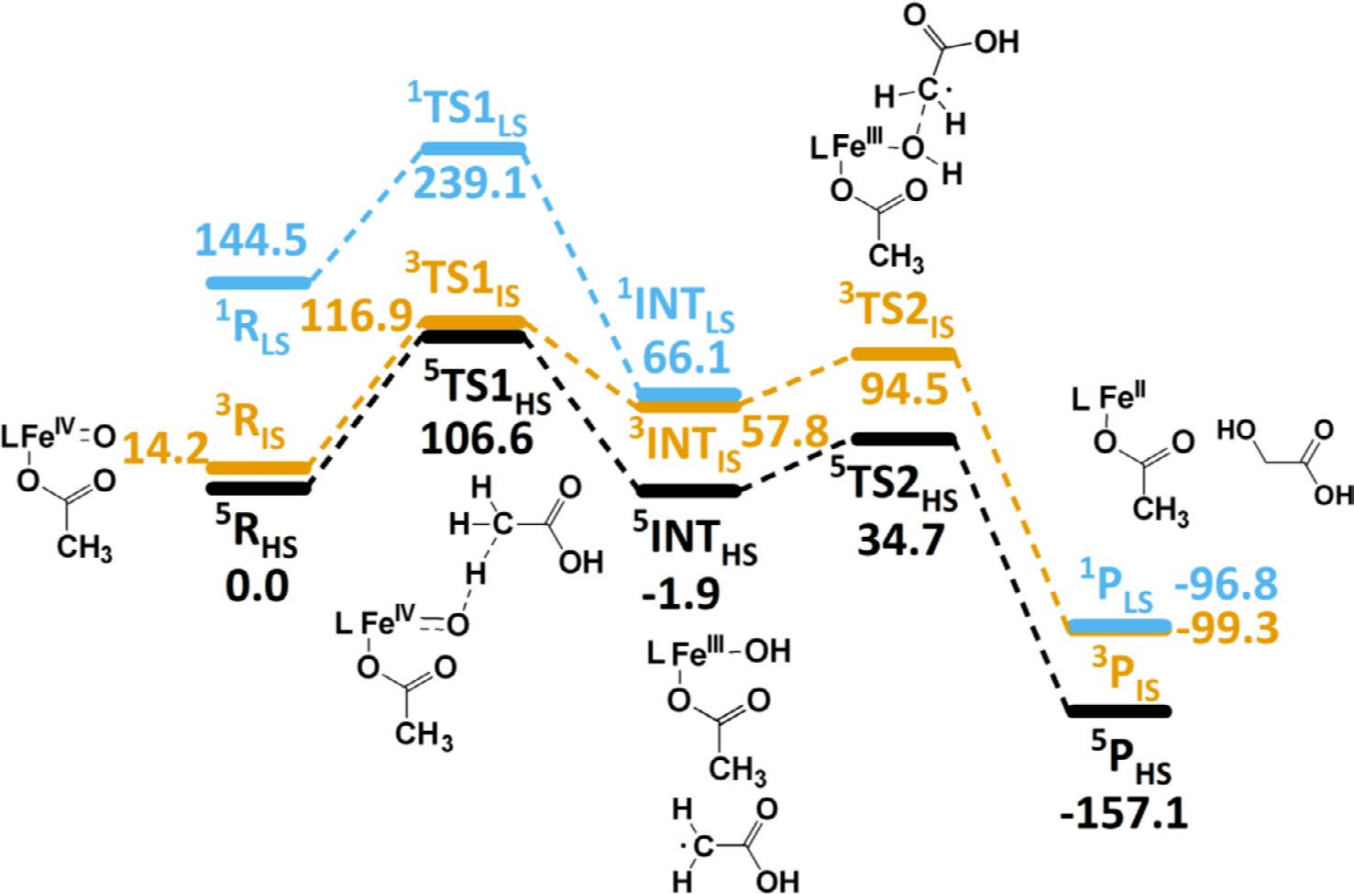
Computed energy profile diagram (kJ/mol) of the C–H abstraction by [(L^1^)Fe^IV^═O]^1+^ at C2 of acetic acid as the rate limiting step in the course of its demethylation (PCM(water)‐B3LYP‐D3/B‐II; Δ*G* in kJ/mol).

**FIGURE 4:**
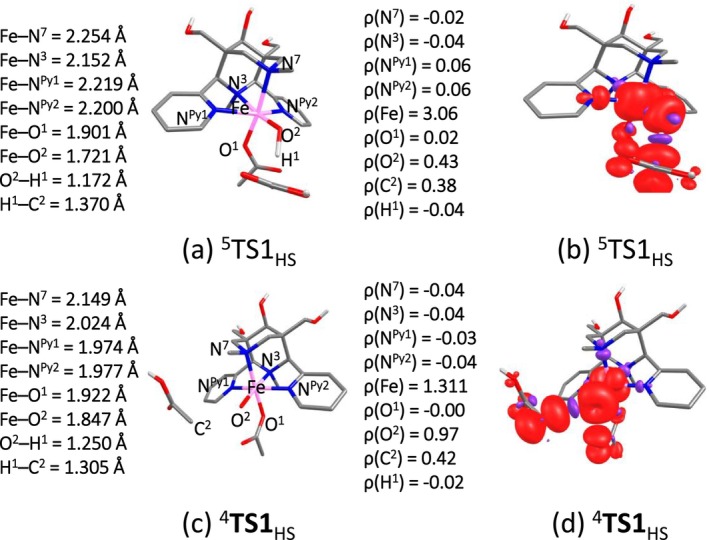
Selected structural parameters and computed spin density plots for the rate‐determining barrier of TS1 of (a, b) [(L^1^)Fe^IV^═O]^1+^ and (c, d) [(L^1^)Fe^V^═O]^2+^.

From experiment it is known that the demethylation of acetic acid by a high‐valent bispidine‐iron complex occurs via C–H abstraction at the methyl group C2.^18^ To support this pathway and, more importantly, to understand the reactivity differences of the demethylation by [(L^1,2^)(OAc)Fe^IV^═O]^1+^ and [(L^1,2^)(OAc)Fe^V^═O]^2+^, for the Fe^IV^═O system (see Scheme 1) we have concentrated on the electrophilic attack of the of Fe^IV^═O at a C–H bond via TS1, yielding Fe^III^–OH and the organic radical at intermediate INT. The radical rebound via TS2 produces the glycolic acid product (producing the observed oxidized C1 species, see Scheme S1 for details) and LFe^II^ which, under catalytic conditions is reoxidized by H_2_O_2_, see Figure 3 for the energy profile plots of [(L^1^)(OAc)Fe^IV^═O]^1+^ and [(L^2^)(OAc)Fe^IV^═O]^1+^, and Figure 4 for structural parameters and spin densities. The relative energies of closely lying spin states of the Fe^IV^═O species need to be interpreted with caution, as small energy differences may depend on the computational method.

For [(L^1^)Fe^IV^═O]^1+^, the computed barrier for C–H abstraction (TS1) is 106.6, 116.9, and 239.1 kJ/mol on the quintet (^5^R_HS_), triplet (^3^R_IS_) and singlet (^1^R_LS_) surfaces, respectively. The Fe–O bond elongates in ^5^TS1_HS_ from 1.62 to 1.72 Å, with an Fe–O^2^–H^1^ angle of 119.0°, indicating that the attack occurs on the generally less efficient π‐pathway [67, 68]. The Fe–O^2^ bond of the newly formed OH group is found to be only slightly longer for ^5^INT_HS_ (1.822 Å) than for ^3^INT_IS_ (1.780 Å) and ^1^INT_LS_ (1.777 Å) (see Tables S4 and S5).

The barrier height for the radical rebound step (TS2) is estimated to be 34.7 and 94.5 kJ/mol on the quintet and triplet surfaces, respectively. The ground state of the product (P) is ^5^P_HS_ with an energy of −157.1 kJ/mol, and ^3^P_IS_ is significantly higher in energy at −99.3 kJ/mol, with ^1^P_LS_ nearly degenerate at −96.8 kJ/mol (Figure 3). In addition to the intermolecular C–H abstraction pathway, an intramolecular hydrogen transfer mechanism, where the oxido group abstracts a hydrogen atom from the methyl group of the coordinated acetate ligand, was examined. Although this route is entropically favored, its activation barrier was calculated to be 22.7 kJ/mol^−1^ higher than that of the intermolecular process, making it kinetically less viable (Figure S5). We also evaluated a hydrogen‐bonding interaction between the acetic acid substrate and the carboxylate oxygen of the complex before the reaction starts. This configuration slightly stabilizes the reactant complex but leads to a higher overall barrier, indicating that hydrogen bonding does not significantly promote the reaction under the conditions studied (Figure S6).

For [(L^2^)(OAc)Fe^IV^═O]^1+^, the barrier height for C–H abstraction (TS1) is estimated to be 125.3, 126.6 and 239.6 kJ/mol on the quintet (^5^R_HS_), triplet (^3^R_IS_) and singlet (^1^R_LS_) surfaces, respectively. The barrier for the radical rebound step (TS2) is at 37.0 and 72.0 kJ/mol on the quintet and triplet surfaces, respectively. The ground state of the product (P) is found to be ^5^P_HS_ with an energy of −161.6 kJ/mol, whereas ^3^P_IS_ is at −102.3 kJ/mol and ^1^P_LS_ is again nearly degenerate at −102.4 kJ/mol (Figure S1). The comparison between the [(L^1^)(OAc)Fe^IV^═O]^1+^ and [(L^2^)(OAc)Fe^IV^═O]^1+^ complexes has been restricted to the mechanistic differences. Both ligands exhibit similar reactivity profiles; however, the quintet TS (^5^TS1_HS_) for the monocationic species of [(L^2^)(OAc)Fe^IV^═O]^1+^ shows a higher activation barrier by approximately 19 kJ/mol compared to [(L^1^)(OAc)Fe^IV^═O]^1+^. This is consistent with the more electron‐withdrawing nature of the carboxylate donor in [(L^2^)(OAc)Fe^IV^═O]^1+^, which slightly reduces spin delocalization on the acetate fragment and yields a more polar TS. Spin density and NBO results provide insight into the electronic changes accompanying the C–H activation step.

#### 3.2.2 The Fe^V^ ═O Pathway

**FIGURE 5:**
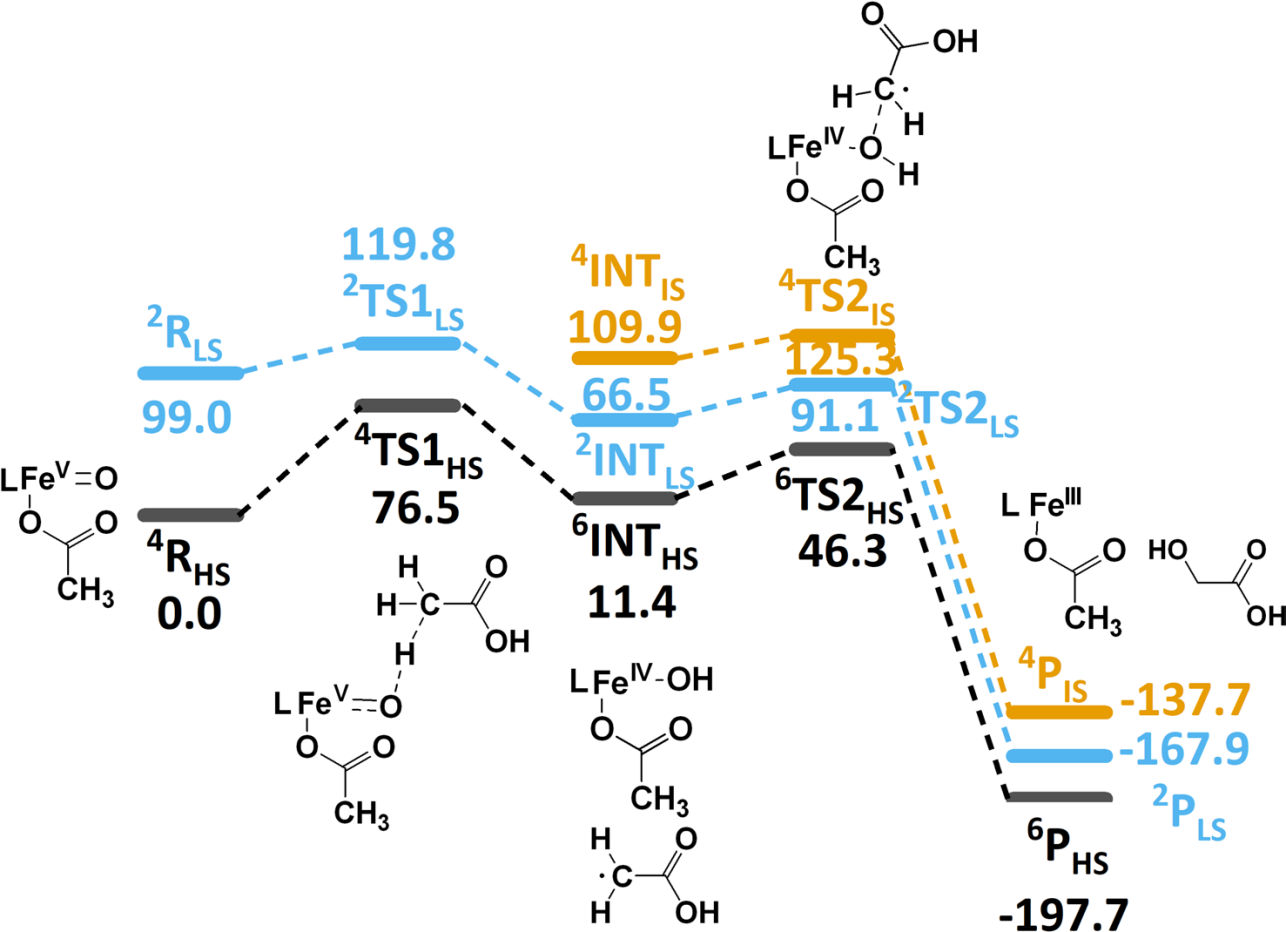
DFT/B3LYP‐D3 computed energy profile diagram (kJ/mol) of the C–H abstraction by [(L^1^)(OAc)Fe^V^═O]^2+^ at C2 of acetic acid as the rate limiting step in the course of its demethylation (PCM(water)‐B3LYP‐D3/B‐II; Δ*G* in kJ/mol).

We have explored intricate mechanistic details of demethylation of acetic acid with [(L^1,2^)(OAc)Fe^V^═O]^2+^ (see Scheme S1). The barrier height of C–H abstraction (TS1) is estimated to be 76.5 and 119.8 kJ/mol on the quartet (^4^R_HS_) and doublet (^2^R_LS_) surfaces, respectively. The Fe–O bonds are elongated in ^4^TS1_HS_ from 1.68 to 1.85 Å. The Fe–O^2^–H^1^ angle (attack of Fe^V^═O at a hydrogen atom at C2) was found to be 129.0°, that is, this follows as expected a π pathway [67, 68]. The barrier height of the radical rebound step (TS2) is estimated at 46.3 and 91.1 kJ/mol on the high‐ and low‐spin surfaces, respectively. The ground state of the product (P) is ^5^P_HS_ with an energy of −197.7 kJ/mol, ^3^P_IS_ is significantly higher at −167.9 kJ/mol and ^1^P_LS_ is at −137.7 kJ/mol (see Figure 5). For [(L^2^)Fe^V^═O]^2+^, the barrier of C–H abstraction (TS1) is at 79.1 and 119.8 kJ/mol on the quartet (^4^R_HS_) and doublet (^2^R_LS_) surfaces, respectively.

The barrier for the radical rebound (TS2) is at 37.1 and 101.2 kJ/mol on the high‐ and low‐spin surfaces, respectively. The ground state of the product (P) is ^5^P_HS_ with an energy of −201.4 kJ/mol, whereas ^3^P_IS_ is at −172.4 kJ/mol and ^1^P_LS_ at −140.0 kJ/mol (see Figure 5; structural parameters and spin densities are given in Figure 4).

Therefore, from the computed energy barriers of the rate determining C–H abstraction, it appears that the Fe^V^═O species are as expected more reactive than the corresponding Fe^IV^═O compounds by 30–45 kJ/mol. To further analyze the nature of the corresponding TS, we have calculated the “bond make‐break average” (BFC_ave_) at the TS with respect to the reactants and products (see Table S1) [45, 69]. A comparably large BFC_ave_ indicates that the TS is product‐like, while a smaller value indicates a reactant‐like TS. The C–H abstraction TS (TS1) of [(L^1,2^)(OAc)Fe^IV^═O]^1+^ are higher in energy and have relatively small BFC_ave_ values, that is, they are typical for an early TS (BFC_ave_ of 29.7 and 25.2, see Table S1), while the corresponding TS of [(L^1,2^)(OAc)Fe^V^═O]^2+^ are significantly lower in energy and have product‐like BFC_ave_ values (BFC_ave_ of 35.6 and 33.1, see Table S1). That is, the observed trend in reactivities is paralleled by the computed BFC_ave_ values.

## 4 Conclusion

With a combination of DFT calculations and in combination with a DLPNO‐CCSD(T) exploration of the electronic ground state of the high‐valent Fe ═ O oxidant, we have explored the mechanism of demethylation of acetic acid by the bispidine‐iron‐oxido complexes [(L^1,2^)(OAc)Fe^IV^═O]^1+^ and [(L^1,2^)(OAc)Fe^V^═O]^2+^. The computed results reveal that small changes in the ligand framework (L^1^ vs. L^2^) do not have a significant influence on the reactivity, as expected. Also, there is no significant difference in the pathway between the two high‐valent Fe^IV^═O and Fe^V^═O oxidants, but the reactivities are much different. The position of the oxido group has, as observed before, a significant influence on the reactivity, and the isomer with the oxido group *trans‐*N^3^ is more stable and therefore the relevant oxidant, and this is also based on published experimental and computational data [21, 63, 64]. A slightly higher value of the WB index for [(L^1,2^)(OAc)Fe^IV^═O]^+^ than for [(L^1,2^)(OAc)Fe^V^═O]^2+^ indicates a larger Fe ═ O double bond character in [(L^1,2^)(OAc)Fe^IV^═O]^+^, and this is also reflected in the computed spin density distributions between Fe and O: there is more radical character on the oxygen atom in the Fe^IV^‐O species. This is further supported by an NBO analysis that reveals that the σ‐bonding interaction in the Fe^IV^═O species is composed of 29.6% Fe(d_z2_) and 70.4% O‐p_z_ orbitals; for the Fe^V^═O species, this is 36.1% Fe(d_z2_) and 63.9% O‐p_z_, suggesting more covalency in the Fe^V^═O than the Fe^IV^═O bond. The activation barriers for the rate‐determining C–H abstraction for the L^1^ and L^2^ based Fe^IV^═O species are approximately 30 and 45 kJ/mol higher than for the corresponding Fe^V^═O species, and this is reflected by the computed BFC_ave_ values indicating early and late TS for the Fe^IV^═O and the Fe^V^═O systems, respectively.

## Funding

This work was supported by the German Science Foundation, CO188/45‐1, INST 40/575‐1 FUGG and German Federal Ministry of Education and Research.

## Conflicts of Interest

The authors declare no conflicts of interest.

## Supporting information


**Supporting Information:** jcc70369‐sup‐0001‐Supinfo.pdf.
**Appendix S1:** Supporting Information jcc70369‐sup‐0001‐Supinfo.docx.

## Data Availability

The data that support the findings of this study are available in the Supporting Information of this article.
