# Supplementary material for: The Reactivity of Bispidine Ligand Based Iron(IV) and Iron(V) Oxido Species for the Demethylation of Acetic Acid
Source: J Comput Chem. 2026 Apr 22;47:e70369. doi: 10.1002/jcc.70369 (PMC13100864; doi:10.1002/jcc.70369)
Supplement: Supplementary file 1 — Supporting Information: jcc70369‐sup‐0001‐Supinfo.pdf. Appendix S1: Supporting Information jcc70369‐sup‐0001‐Supinfo.docx. [file JCC-47-0-s001.docx]

**The reactivity of bispidine ligand based iron(IV) and iron(V) oxido species for the demethylation of acetic acid**

*Gunasekaran Velmurugan^1,2^ and Peter Comba^1^**

***Supporting Information***

^1^Universität Heidelberg, Anorganisch-Chemisches Institut und Interdisziplinäres Zentrum für Wissenschaftliches Rechnen, Im Neuenheimer Feld 270, D-69120 Heidelberg, Germany

^2^Department of Chemistry, National Institute of Technology – Tiruchirappalli, Tiruchirappalli, 620015, Tamil Nadu, India

**Correspondence**

E-mail: [peter.comba@aci.uni-heidelberg.de](mailto:peter.comba@aci.uni-heidelberg.de)

**Scheme S1.** Proposed reaction pathway for the oxidation of acetic acid via [(L^1,2^)Fe^IV^=O]^1+^ species.

**Scheme S2.** Proposed reaction pathway for the oxidation of acetic acid via [(L^1,2^)Fe^V^=O]^2+^ species.

**Figure S1.** DFT/B3LYP-D3 computed energy profile diagram (kJ/mol) of the C-H abstraction by [(L^2^)Fe^IV^=O]^1+^ at C2 of acetic acid, leading to its demethylation.

**Figure S2.** DF DFT/B3LYP-D3 computed energy profile diagram (kJ/mol) of the C-H abstraction by [(L^2^)Fe^V^=O]^2+^ at C2 of acetic acid, leading to its demethylation.

**Figure S3.** DFT/B3LYP-D3 computed energy profile diagram (kJ/mol) of the demethylation of acetic acid reaction with [(L^1^)Fe^IV^=O]^1+^ (O trans to N^7^).

**Figure S4.** DFT/B3LYP-D3 computed energy profile diagram (kJ/mol) of the demethylation of acetic acid reaction with [(L^2^)Fe^IV^=O]^1+^ (O trans to N^7^).


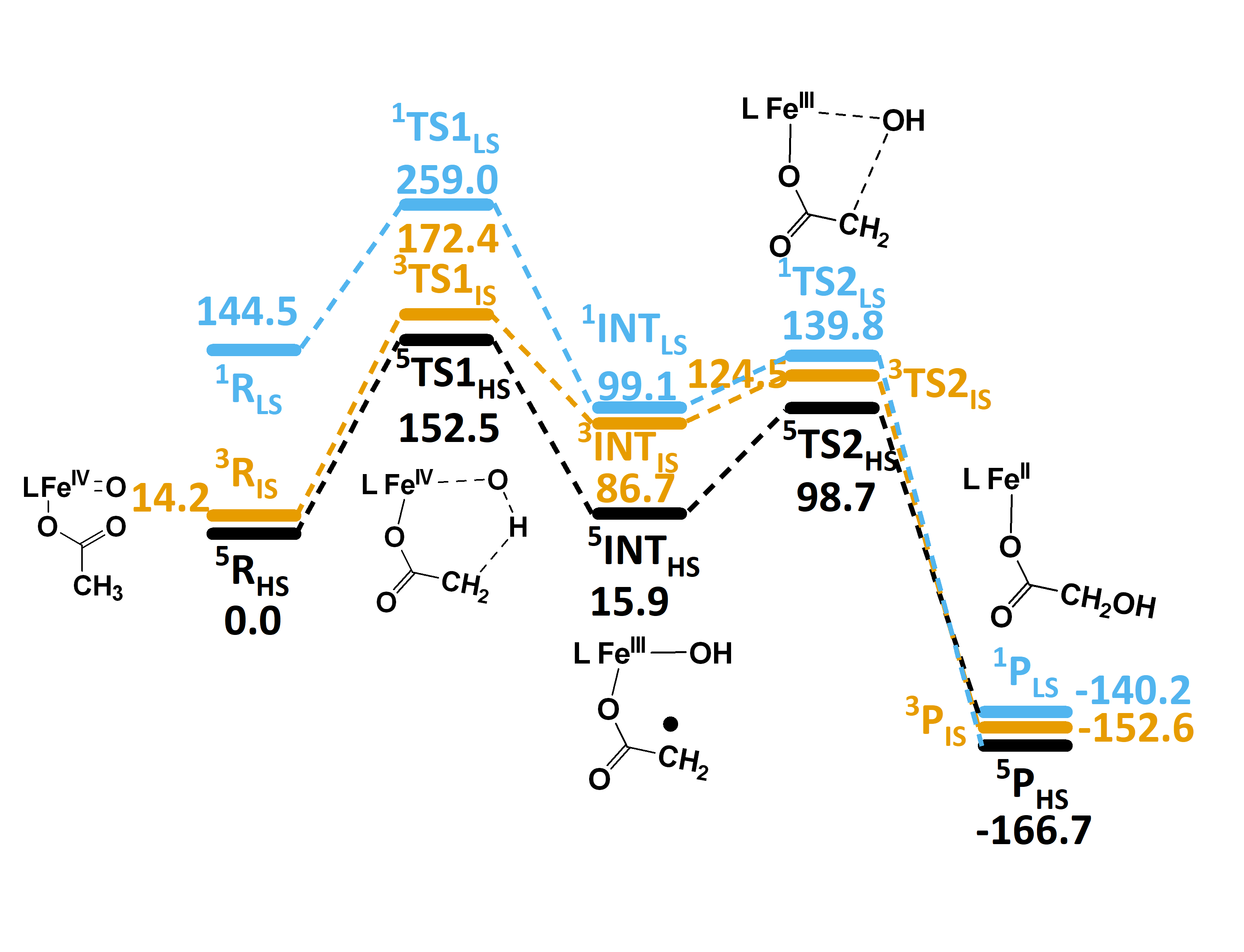


**Figure S5.** DFT/B3LYP-D3 computed energy profile diagram (kJ/mol) of the demethylation of acetic acid reaction with [(L^1^)Fe^IV^=O]^1+^ (intramolecular pathway).


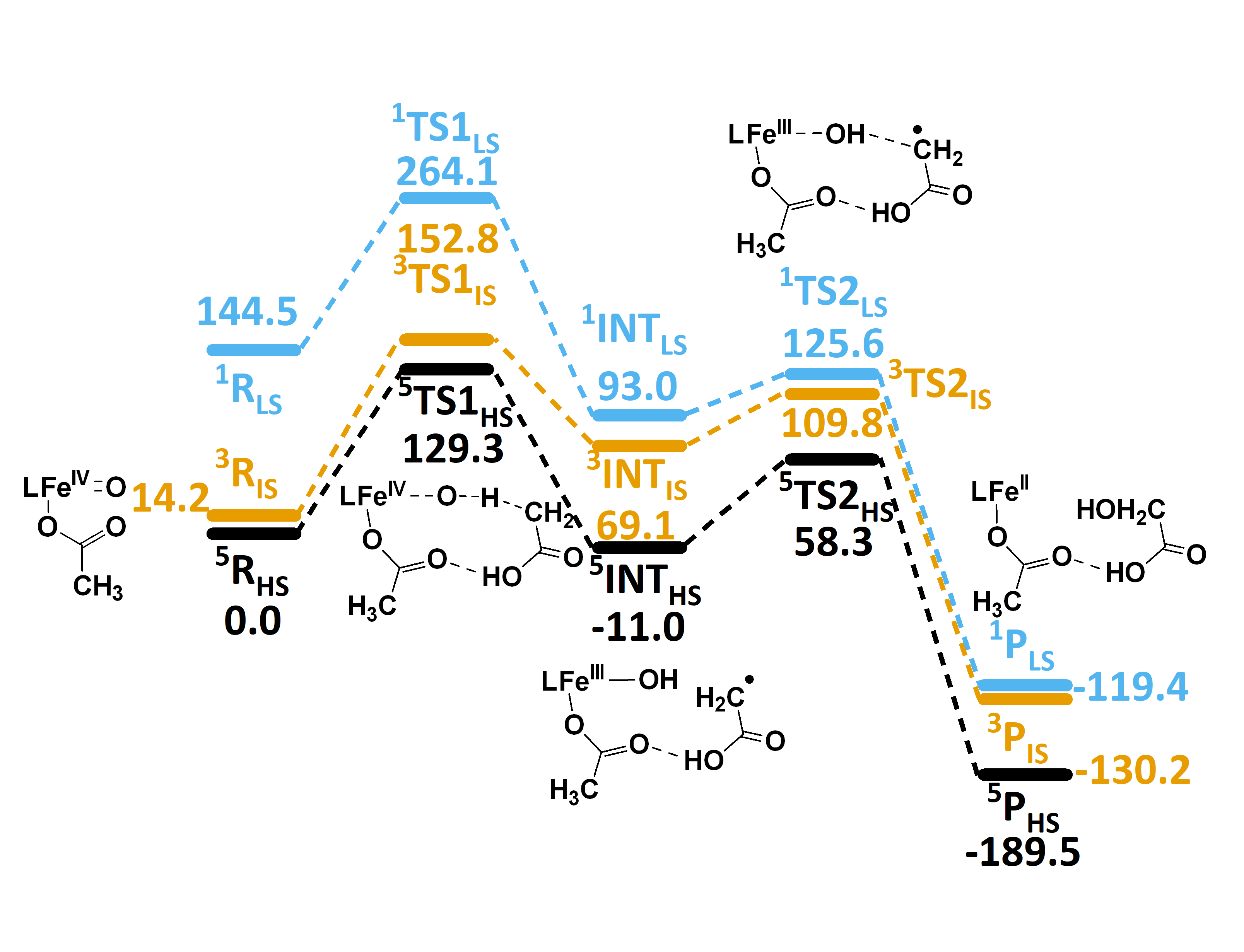


**Figure S6.** DFT/B3LYP-D3 computed energy profile diagram (kJ/mol) of the demethylation of acetic acid reaction with [(L^1^)Fe^IV^=O]^1+^ (H-bonding pathway).

**Figure S7.** DFT/B3LYP-D3 computed energy profile diagram (kJ/mol) of the demethylation of acetic acid reaction with [(L^1^)Fe^V^=O]^2+^ (O trans to N^7^).

**Figure S8.** DFT/B3LYP-D3 computed energy profile diagram (kJ/mol) of the demethylation of acetic acid reaction with [(L^2^)Fe^V^=O]^2+^ (O trans to N^7^).

**O *trans* to N^3^ isomer results**


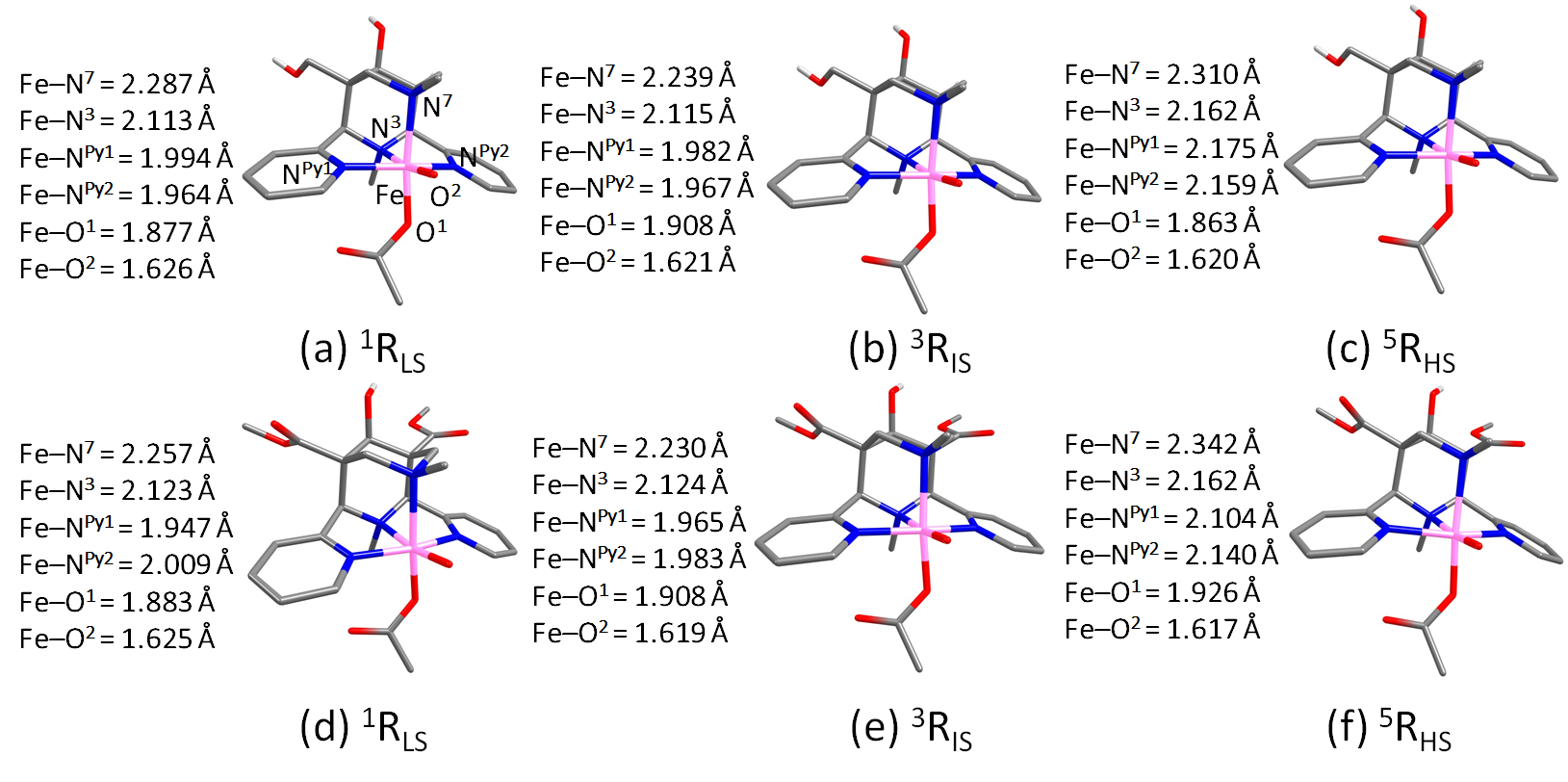


**Figure S9.** Optimized geometries of the various spin states for R of (a), (b), (c) O *trans* N^3^ [(L^1^)Fe^IV^=O]^1+^  and (d), (d), (f) O *trans* N^3^-[(L^2^)Fe^IV^=O]^1+^.


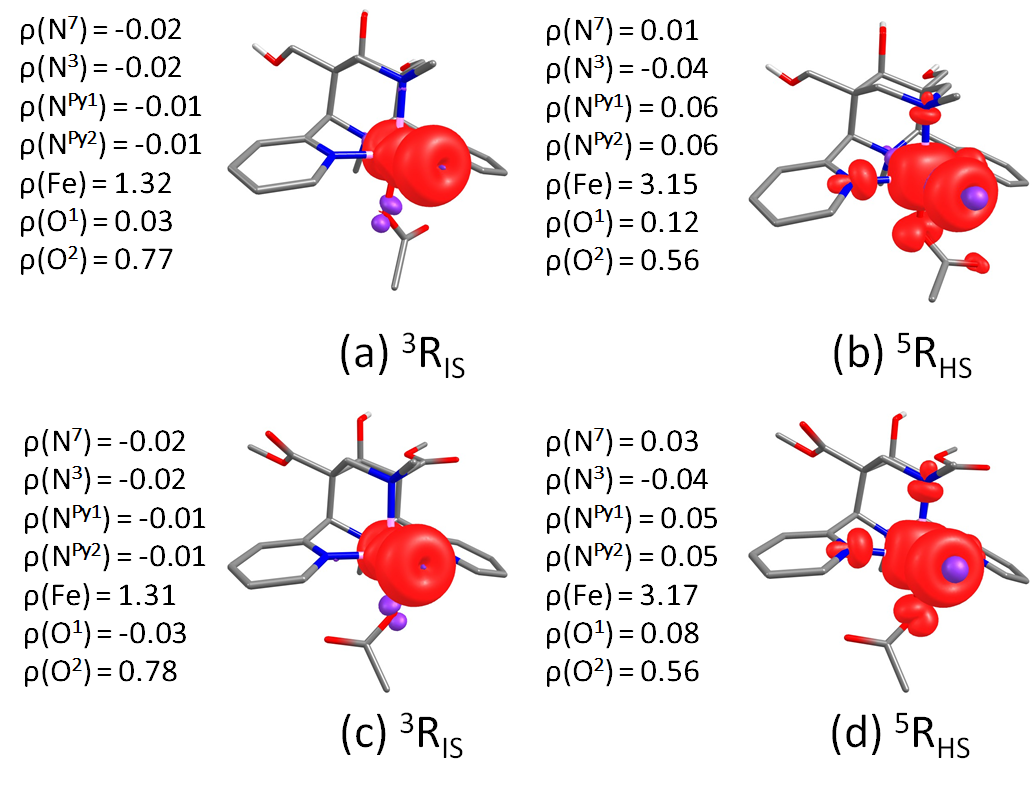


**Figure S10.** Computed spin density plots for R of (a), (b) [(L^1^)Fe^IV^=O]^1+^  and (c), (d) [(L^2^)Fe^IV^=O]^1+^.


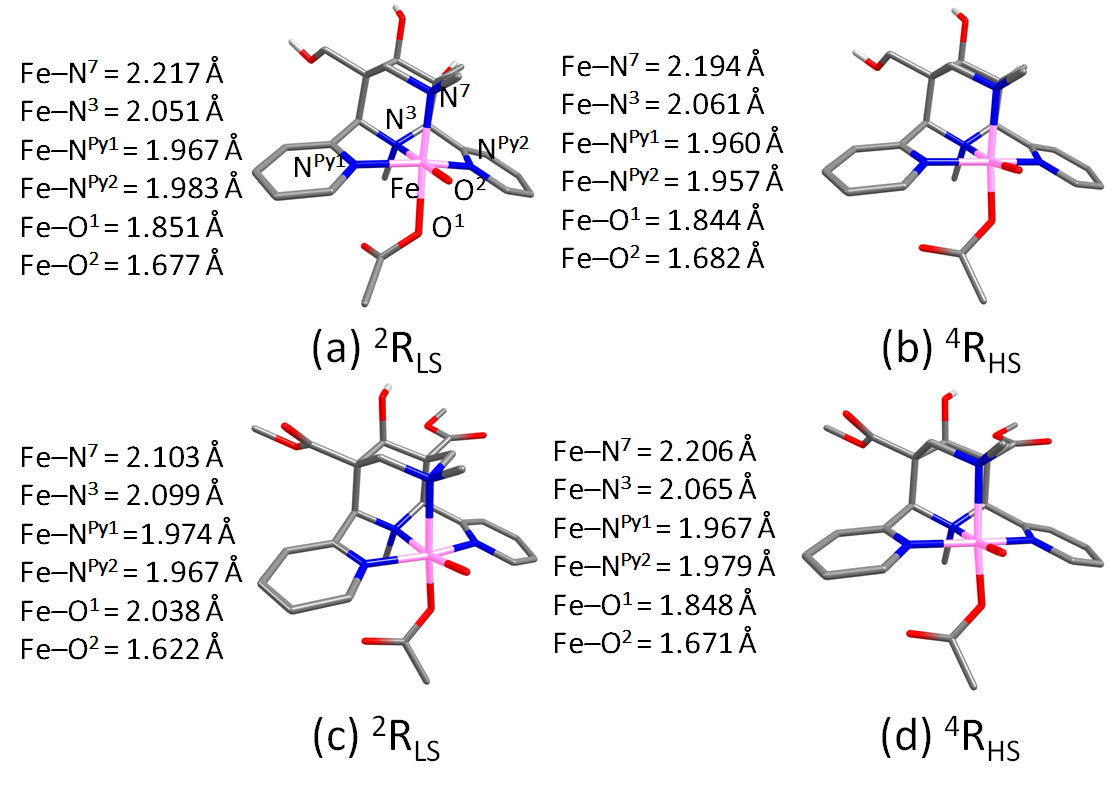


**Figure S11.** Optimized geometries of the various spin states for R of (a), (b) [(L^1^)Fe^V^=O]^2+^ and (c), (d) [(L^2^)Fe^V^=O]^2+^.


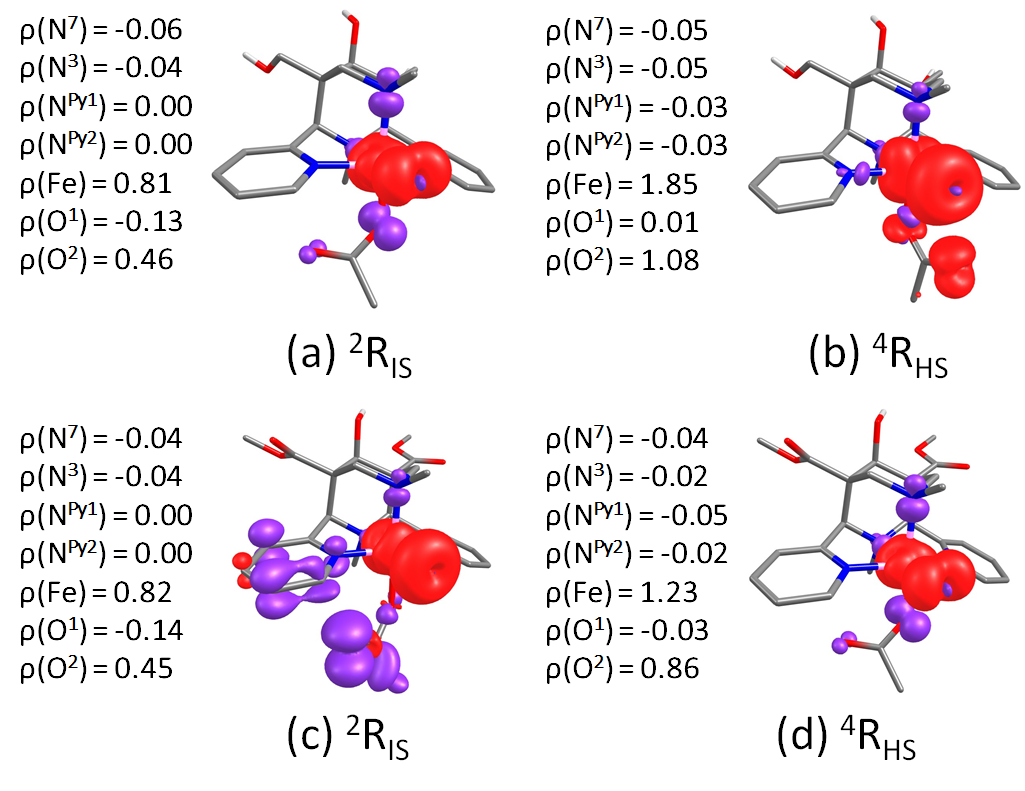


**Figure S12.** Computed spin density plots for R of (a), (b) [(L^1^)Fe^V^=O]^2+^  and (c), (d) [(L^2^)Fe^V^=O]^2+^.


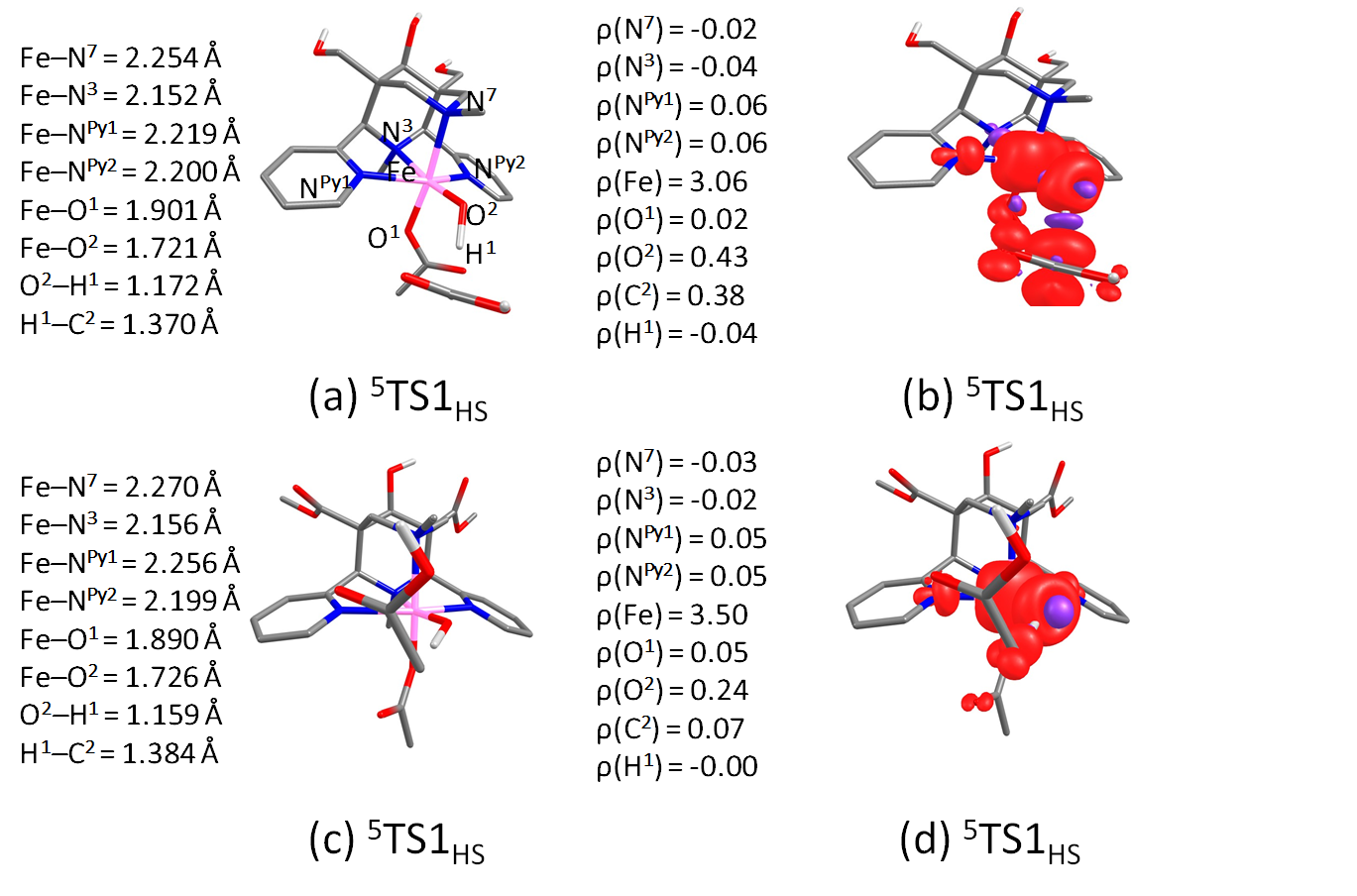


**Figure S13.** Selected structural parameters and computed spin density plots for the rate-determining barrier of TS1 of (a), (b) [(L^1^)Fe^IV^=O]^1+^  and (c), (d) [(L^2^)Fe^IV^=O]^1+^.


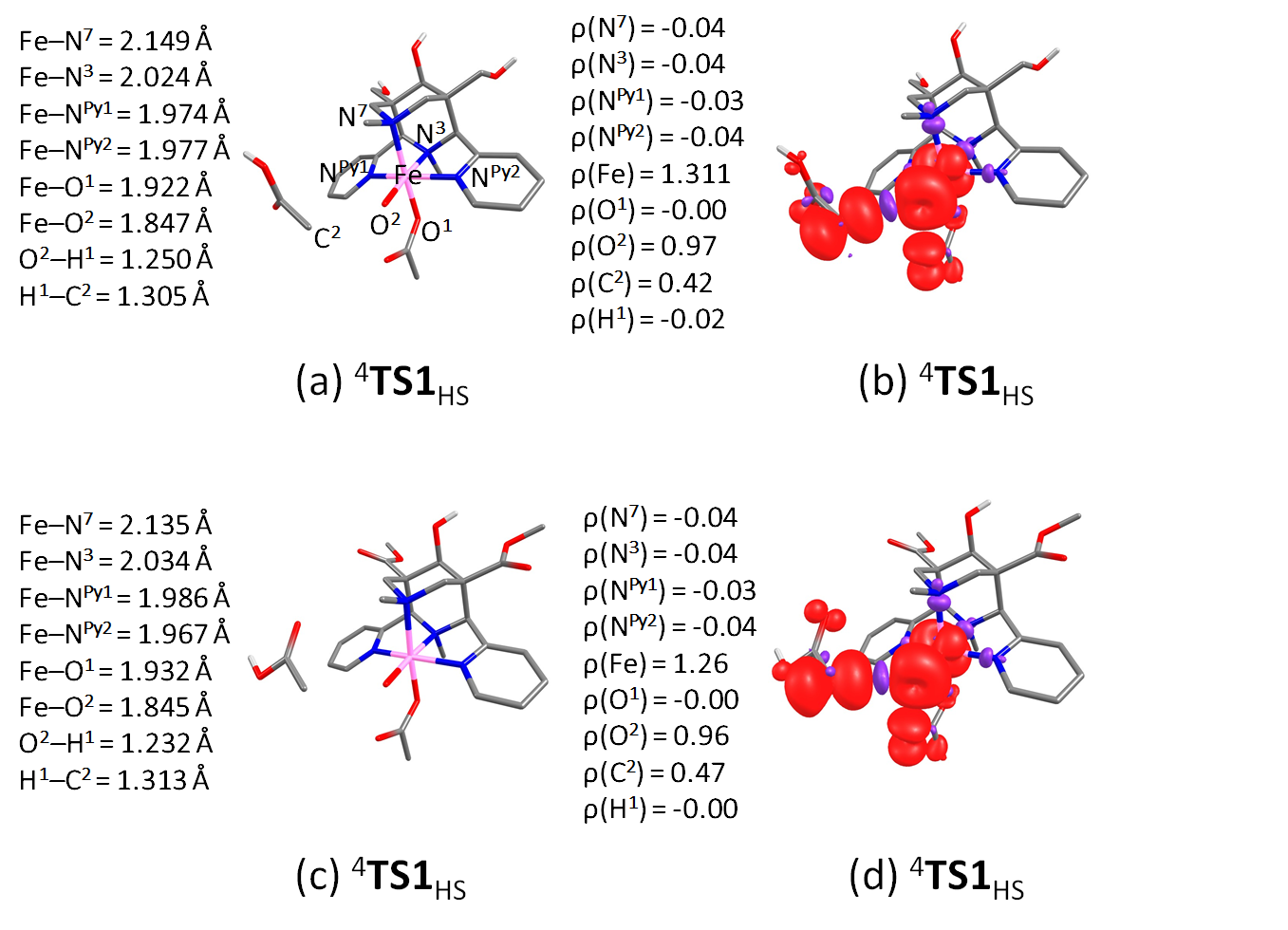


**Figure S14.** Selected structural parameters and computed spin density plots for TS1 of (a), (b) [(L^1^)Fe^V^=O]^2+^  and (c), (d) [(L^2^)Fe^V^=O]^2+^ .

**Table S1.** Percentages (%) of Bond Cleavage (BC_f_) and Bond Formation (BF_i_) in the TSs involving in the C-H abstraction and radical rebound step of the demethylation of acetic acid reactions with [(L^1,2^)Fe^IV^=O]^1+^ and [(L^1,2^)Fe^V^=O]^2+^ species.

|  | Species | BF_i_ | BC_f_ | BFC_ave_ |
| --- | --- | --- | --- | --- |
| TS1 | [(L^1^)Fe^IV^=O]^1+^ | 28.8 | 27.9 | 29.7 |
|  | [(L^2^)Fe^IV^=O]^1+^ | 27.9 | 26.8 | 25.2 |
|  | [(L^1^)Fe^V^=O]^2+^ | 34.3 | 32.9 | 35.6 |
|  | [(L^2^)Fe^V^=O]^2+^ | 32.0 | 30.3 | 33.1 |
| TS2 | [(L^1^)Fe^IV^=O]^1+^ | 22.4 | 23.0 | 27.2 |
|  | [(L^2^)Fe^IV^=O]^1+^ | 21.9 | 22.0 | 26.9 |
|  | [(L^1^)Fe^V^=O]^2+^ | 26.1 | 25.3 | 25.7 |
|  | [(L^2^)Fe^V^=O]^2+^ | 21.7 | 23.9 | 25.7 |

**Table S2.** Structural parameters of [(L^1,2^)Fe^IV^=O]^1+^ (R).

| Bond length (Å) | [(L^1^)Fe^IV^=O]^1+^ | | | [(L^2^)Fe^IV^=O]^1+^ | | |
| --- | --- | --- | --- | --- | --- | --- |
|  | ^5^­R_HS_ | ^3^­R_IS_ | ^1^R_LS_ | ^5^­R_HS_ | ^3^­R_IS_ | ^1^R_LS_ |
| Fe-N^7^ | 2.310 | 2.239 | 2.287 | 2.342 | 2.230 | 2.257 |
| Fe-N^Py1^ | 2.175 | 1.982 | 1.994 | 2.104 | 1.965 | 1.947 |
| Fe-N^Py2^ | 2.159 | 1.967 | 1.964 | 2.140 | 1.983 | 2.009 |
| Fe-N^3^ | 2.162 | 2.115 | 2.113 | 2.162 | 2.124 | 2.123 |
| Fe-O^1^ | 1.863 | 1.908 | 1.877 | 1.926 | 1.908 | 1.883 |
| Fe-O^2^ | 1.620 | 1.621 | 1.626 | 1.617 | 1.619 | 1.625 |

**Table S3.** Structural parameters of [(L^1,2^)Fe^IV^=O]^1+^ (R).

|  | [(L^1^)Fe^IV^=O]^1+^ | | | [(L^2^)Fe^IV^=O]^1+^ | | |
| --- | --- | --- | --- | --- | --- | --- |
| Bond angle (°) | ^5^­R_HS_ | ^3^­R_IS_ | ^1^R_LS_ | ^5^­R_HS_ | ^3^­R_IS_ | ^1^R_LS_ |
| N^Py1-^Fe-N^Py2^ | 155.5 | 163.2 | 162.8 | 157.6 | 1.6 | 163.8 |
| N^Py1^-Fe-N^3^ | 77.6 | 81.4 | 81.0 | 79.1 | 82.3 | 83.1 |
| N^Py1-^Fe-N^7^ | 88.7 | 91.4 | 91.0 | 88.5 | 90.4 | 88.7 |
| N^Py1-^Fe-O^1^ | 88.3 | 81.4 | 95.4 | 97.8 | 95.9 | 97.1 |
| N^Py1-^Fe-O^2^ | 103.4 | 99.1 | 92.5 | 98.7 | 97.5 | 101.1 |
| O^1^-Fe-O^2^ | 96.0 | 92.8 | 97.4 | 95.1 | 92.8 | 96.1 |
| N^3^-Fe-N^7^ | 81.5 | 84.0 | 82.9 | 81.2 | 84.2 | 83.5 |

**Table S4.** Structural parameters of [(L^1,2^)Fe^IV^=O]^1+^ (INT).

|  | [(L^1^)Fe^IV^=O]^1+^ | | | [(L^2^)Fe^IV^=O]^1+^ | | |
| --- | --- | --- | --- | --- | --- | --- |
| Bond length (Å) | ^5^­INT_HS_ | ^3^­INT_IS_ | ^1^INT_LS_ | ^5^­INT_HS_ | ^3^­INT_IS_ | ^1^INT_LS_ |
| Fe-N^7^ | 2.429 | 2.222 | 2.177 | 2.406 | 2.212 | 2.170 |
| Fe-N^Py1^ | 2.145 | 2.180 | 1.968 | 2.143 | 2.183 | 1.968 |
| Fe-N^Py2^ | 2.169 | 2.225 | 1.993 | 2.161 | 2.220 | 1.990 |
| Fe-N^3^ | 2.274 | 2.123 | 2.061 | 2.301 | 2.142 | 2.070 |
| Fe-O^1^ | 1.915 | 1.942 | 1.954 | 1.915 | 1.937 | 1.953 |
| Fe-O^2^ | 1.840 | 1.783 | 1.786 | 0.972 | 0.976 | 0.976 |

**Table S5.** Structural parameters of [(L^1,2^)Fe^IV^=O]^1+^ (INT).

|  | [(L^1^)Fe^IV^=O]^1+^ | | | [(L^2^)Fe^IV^=O]^1+^ | | |
| --- | --- | --- | --- | --- | --- | --- |
| Bond angle (°) | ^5^­INT_HS_ | ^3^INT_IS_ | ^1^INT_LS_ | ^5^­INT_HS_ | ^3^INT_IS_ | ^1^INT_LS_ |
| N^Py1-^Fe-N^Py2^ | 151.2 | 154.6 | 163.0 | 151.0 | 154.5 | 163.2 |
| N^Py1^-Fe-N^3^ | 76.2 | 78.4 | 82.7 | 76.1 | 78.3 | 82.8 |
| N^Py1-^Fe-N^7^ | 86.9 | 91.3 | 91.2 | 86.7 | 91.2 | 91.1 |
| N^Py1-^Fe-O^1^ | 93.9 | 96.3 | 94.6 | 93.5 | 96.4 | 94.6 |
| N^Py1-^Fe-O^2^ | 104.9 | 101.2 | 97.4 | 104.6 | 101.2 | 97.1 |
| O^1^-Fe-O^2^ | 96.4 | 85.3 | 84.7 | 96.7 | 85.4 | 84.8 |
| Fe-O^2^-H^1^ | 111.8 | 105.1 | 103.3 | 112.5 | 105.3 | 103.3 |
| N^3^-Fe-N^7^ | 77.7 | 91.3 | 86.2 | 77.9 | 84.4 | 86.4 |

|  | | Fe | N^Py1^ | N^Py2^ | N^3^ | N^7^ | O^1^ | O^2^ | C^1^ | H^1^ |
| --- | --- | --- | --- | --- | --- | --- | --- | --- | --- | --- |
| [(L^1^)Fe^IV^=O]^1+^ | ^5^­R_HS_ | 0.607 | -0.124 | -0.139 | -0.161 | -0.107 | -0.460 | -0.538 | 0.348 | - |
|  | ^3^R_IS_ | 0.223 | 0.061 | 0.056 | -0.145 | -0.037 | -0.424 | -0.527 | 0.388 | - |
|  | ^1^R_LS_ | 0.149 | 0.049 | 0.079 | -0.199 | 0.058 | -0.523 | -0.495 | 0.351 | - |
| [(L^2^)Fe^IV^=O]^1+^ | ^5^­R_HS_ | 0.582 | -0.106 | -0.108 | -0.180 | -0.123 | -0.480 | -0.507 | 0.404 | - |
|  | ^3^R_IS_ | 0.218 | 0.062 | 0.056 | -0.156 | -0.031 | -0.426 | -0.516 | 0.390 | - |
|  | ^1^R_LS_ | 0.240 | 0.075 | 0.038 | -0.145 | 0.038 | -0.426 | -0.522 | 0.391 | - |
| [(L^1^)Fe^IV^=O]^1+^ | ^5^­INT_HS_ | 0.741 | -0.145 | -0.134 | -0.275 | -0.184 | -0.504 | -0.709 | 0.408 | 0.325 |
|  | ^3^INT_IS_ | 0.542 | -0.155 | -0.148 | -0.224 | -0.070 | -0.506 | -0.628 | 0.394 | 0.333 |
|  | ^1^INT_LS_ | 0.092 | 0.067 | 0.083 | -0.136 | -0.011 | -0.493 | -0.624 | 0.392 | 0.308 |
| [(L^2^)Fe^IV^=O]^1+^ | ^5^­INT_HS_ | 0.733 | -0.149 | -0.133 | -0.280 | -0.181 | -0.501 | -0.704 | 0.414 | 0.328 |
|  | ^3^INT_IS_ | 0.538 | -0.162 | -0.152 | -0.239 | -0.239 | -0.500 | -0.620 | 0.398 | 0.336 |
|  | ^1^INT_LS_ | 0.082 | 0.064 | 0.085 | -0.148 | -0.004 | -0.493 | -0.614 | 0.396 | 0.311 |

**Table S6.** Mulliken charges of [(L^1,2^)Fe^IV^=O]^1+^ (R and INT).

**Table S7.** Spin density values of [(L^1,2^)Fe^IV^=O]^1+^ (R and INT).

|  | | Fe | N^Py1^ | N^Py2^ | N^3^ | N^7^ | O^1^ | O^2^ | C^1^ | H^1^ |
| --- | --- | --- | --- | --- | --- | --- | --- | --- | --- | --- |
| [(L^1^)Fe^IV^=O]^1+^ | ^5^­R_HS_ | 3.151 | 0.059 | 0.061 | -0.036 | 0.010 | 0.118 | 0.560 | 0.003 | - |
|  | ^3^R_IS_ | 1.318 | -0.012 | -0.013 | -0.021 | -0.018 | -0.025 | 0.771 | -0.001 | - |
|  | ^1^R_LS_ | 0.000 | 0.000 | 0.000 | 0.000 | 0.000 | 0.000 | 0.000 | 0.000 | - |
| [(L^2^)Fe^IV^=O]^1+^ | ^5^­R_HS_ | 3.174 | 0.052 | 0.054 | -0.038 | 0.026 | 0.084 | 0.564 | 0.003 | - |
|  | ^3^R_IS_ | 1.308 | -0.013 | -0.011 | -0.021 | -0.017 | -0.025 | 0.777 | -0.001 | - |
|  | ^1^R_LS_ | 0.000 | 0.000 | 0.000 | 0.000 | 0.000 | 0.000 | 0.000 | 0.000 | 0.000 |
| [(L^1^)Fe^IV^=O]^1+^ | ^5^­INT_HS_ | 4.200 | 0.069 | 0.063 | 0.053 | 0.049 | 0.142 | -0.993 | 0.005 | 0.008 |
|  | ^3^INT_IS_ | 2.620 | 0.052 | 0.049 | -0.028 | -0.054 | -0.028 | -0.856 | 0.002 | 0.013 |
|  | ^1^INT_LS_ | 0.949 | -0.012 | -0.009 | -0.014 | -0.021 | -0.021 | -0.727 | 0.001 | -0.005 |
| [(L^2^)Fe^IV^=O]^1+^ | ^5^­INT_HS_ | 4.196 | 0.069 | 0.069 | 0.045 | 0.047 | 0.146 | -0.930 | 0.005 | 0.008 |
|  | ^3^INT_IS_ | 2.414 | 0.052 | 0.050 | -0.024 | -0.056 | -0.034 | -0.862 | 0.002 | 0.013 |
|  | ^1^INT_LS_ | 0.943 | -0.011 | -0.009 | -0.014 | -0.021 | -0.021 | -0.731 | 0.001 | -0.005 |

**Table S8.** Structural parameters of [(L^1,2^)Fe^IV^=O]^1+^ (TS1).

|  | [(L^1^)Fe^IV^=O]^1+^ | | | [(L^2^)Fe^IV^=O]^1+^ | | |
| --- | --- | --- | --- | --- | --- | --- |
| Bond length (Å) | ^5^­TS1_HS_ | ^3^­TS1_IS_ | ^1^TS1_LS_ | ^5^­TS1_HS_ | ^3^­TS1_IS_ | ^1^TS1_LS_ |
| Fe-N^7^ | 2.254 | 2.208 | 2.183 | 2.270 | 2.240 | 2.181 |
| Fe-N^Py1^ | 2.219 | 1.999 | 1.987 | 2.255 | 2.008 | 1.987 |
| Fe-N^Py2^ | 2.200 | 1.999 | 2.002 | 2.199 | 1.997 | 2.002 |
| Fe-N^3^ | 2.152 | 2.099 | 2.081 | 2.156 | 2.104 | 2.081 |
| Fe-O^1^ | 1.901 | 1.910 | 1.928 | 1.890 | 1.880 | 1.929 |
| Fe-O^2^ | 1.721 | 1.723 | 1.755 | 1.726 | 1.723 | 1.754 |
| O^2^-H^1^ | 1.172 | 1.173 | 1.078 | 1.159 | 1.169 | 1.078 |
| H^1^-C^2^ | 1.370 | 2.214 | 1.519 | 1.384 | 1.380 | 1.520 |

**Table S9.** Structural parameters of [(L^1,2^)Fe^IV^=O]^1+^ (TS2).

|  | [(L^1^)Fe^IV^=O]^1+^ | | [(L^2^)Fe^IV^=O]^1+^ | |
| --- | --- | --- | --- | --- |
| Bond length (Å) | ^5^­TS2_HS_ | ^3^­TS2_IS_ | ^5^­TS2_HS_ | ^3^­TS2_IS_ |
| Fe-N^7^ | 2.238 | 2.197 | 2.220 | 2.179 |
| Fe-N^Py1^ | 2.239 | 1.998 | 2.239 | 1.980 |
| Fe-N^Py2^ | 2.209 | 2.009 | 2.212 | 1.993 |
| Fe-N^3^ | 2.164 | 2.124 | 2.182 | 2.088 |
| Fe-O^1^ | 1.984 | 1.939 | 1.977 | 2.088 |
| Fe-O^2^ | 1.803 | 1.856 | 1.802 | 1.945 |
| O^2^-H^1^ | 0.999 | 0.994 | 0.999 | 0.989 |
| O^2^-C^2^ | 2.360 | 2.100 | 2.376 | 3.427 |

**Table S10.** Structural parameters of [(L^1,2^)Fe^IV^=O]^1+^ (TS1).

|  | [(L^1^)Fe^IV^=O]^1+^ | | | [(L^2^)Fe^IV^=O]^1+^ | | |
| --- | --- | --- | --- | --- | --- | --- |
| Bond angle (°) | ^5^­TS1_HS_ | ^3^­TS1_IS_ | ^1^TS1_LS_ | ^5^­TS1_HS_ | ^3^­TS1_IS_ | ^1^TS1_LS_ |
| N^Py1-^Fe-N^Py2^ | 154.7 | 163.3 | 162.4 | 154.7 | 162.5 | 162.3 |
| N^Py1^-Fe-N^3^ | 77.6 | 81.9 | 81.5 | 77.2 | 81.9 | 81.4 |
| N^Py1-^Fe-N^7^ | 89.8 | 91.8 | 96.3 | 88.9 | 91.8 | 96.3 |
| N^Py1-^Fe-O^1^ | 85.2 | 82.0 | 83.5 | 90.7 | 90.2 | 83.4 |
| N^Py1-^Fe-O^2^ | 104.4 | 101.4 | 102.4 | 114.2 | 106.7 | 102.5 |
| O^1^-Fe-O^2^ | 96.6 | 95.6 | 89.9 | 114.2 | 96.5 | 89.9 |
| Fe-O^2^-H^1^ | 119.0 | 118.0 | 127.5 | 125.4 | 127.0 | 127.4 |
| N^3^-Fe-N^7^ | 83.3 | 85.3 | 85.7 | 82.4 | 84.2 | 85.8 |

**Table S11.** Structural parameters of [(L^1,2^)Fe^IV^=O]^1+^ (TS2)

|  | [(L^1^)Fe^IV^=O]^1+^ | | [(L^2^)Fe^IV^=O]^1+^ | |
| --- | --- | --- | --- | --- |
| Bond angle (°) | ^5^­TS2_HS_ | ^3^­TS2_IS_ | ^5^­TS2_HS_ | ^3^­TS2_IS_ |
| N^Py1-^Fe-N^Py2^ | 150.0 | 161.9 | 149.5 | 161.9 |
| N^Py1^-Fe-N^3^ | 76.0 | 81.6 | 75.6 | 81.7 |
| N^Py1-^Fe-N^7^ | 94.4 | 91.8 | 95.2 | 92.4 |
| N^Py1-^Fe-O^1^ | 83.0 | 85.3 | 83.4 | 85.0 |
| N^Py1-^Fe-O^2^ | 102.5 | 98.0 | 102.6 | 97.4 |
| O^1^-Fe-O^2^ | 92.6 | 92.4 | 92.9 | 93.9 |
| Fe-O^2^-H^1^ | 103.8 | 102.8 | 104.1 | 105.5 |
| N^3^-Fe-N^7^ | 83.5 | 85.2 | 83.6 | 86.0 |
| O^2^-H^1^-C^2^ | 145.5 | 128.2 | 145.2 | 105.5 |

**Table S12.** Mulliken charges of [(L^1,2^)Fe^IV^=O]^1+^ (TS1 and TS2).

|  | | Fe | N^Py1^ | N^Py2^ | N^3^ | N^7^ | O^1^ | O^2^ | O^3^ | C^1^ | C^2^ | H^1^ | C^3^ | O^4^ |
| --- | --- | --- | --- | --- | --- | --- | --- | --- | --- | --- | --- | --- | --- | --- |
| [(L^1^)Fe^IV^=O]^1+^ | ^5^­TS1_HS_ | 0.660 | -0.159 | -0.190 | -0.144 | -0.083 | -0.455 | -0.589 | -0.448 | 0.376 | -0.418 | 0.288 | 0.365 | -0.415 |
|  | ^3^TS1_IS_ | 0.257 | 0.077 | 0.019 | -0.095 | -0.007 | -0.430 | -0.612 | -0.465 | 0.358 | -0.428 | 0.271 | 0.364 | -0.418 |
|  | ^1^TS1_LS_ | 0.148 | 0.102 | 0.035 | -0.068 | -0.007 | -0.424 | -0.500 | -0.410 | 0.368 | -0.455 | 0.282 | 0.301 | -0.397 |
| [(L^2^)Fe^IV^=O]^1+^ | ^5^­TS1_HS_ | 0.697 | -0.217 | -0.132 | -0.160 | -0.124 | -0.455 | -0.600 | -0.449 | 0.384 | -0.414 | 0.299 | 0.321 | -0.413 |
|  | ^3^TS1_IS_ | 0.266 | 0.049 | 0.090 | -0.097 | -0.049 | -0.433 | -0.602 | -0.462 | 0.356 | -0.372 | 0.269 | 0.302 | -0.395 |
|  | ^1^TS1_LS_ | 0.149 | 0.095 | 0.046 | -0.087 | -0.011 | -0.427 | -0.494 | -0.408 | 0.369 | -0.450 | 0.282 | 0.301 | -0.392 |
| [(L^1^)Fe^IV^=O]^1+^ | ^5^­TS2_HS_ | 0.614 | -0.174 | -0.171 | -0.194 | -0.106 | -0.479 | -0.657 | -0.540 | 0.450 | -0.177 | 0.348 | 0.303 | -0.407 |
|  | ^3^TS2_IS_ | 0.228 | 0.047 | 0.042 | -0.142 | -0.039 | -0.448 | -0.645 | -0.527 | 0.412 | -0.209 | 0.343 | 0.313 | -0.437 |
| [(L^2^)Fe^IV^=O]^1+^ | ^5^­TS2_HS_ | 0.616 | -0.176 | -0.184 | -0.208 | -0.105 | -0.477 | -0.655 | -0.535 | 0.452 | -0.177 | 0.350 | 0.303 | -0.406 |
|  | ^3^TS2_IS_ | 0.183 | 0.052 | 0.055 | -0.119 | -0.011 | -0.450 | -0.680 | -0.534 | 0.428 | -0.248 | 0.174 | 0.374 | -0.407 |

|  | | Fe | N^Py1^ | N^Py2^ | N^3^ | N^7^ | O^1^ | O^2^ | O^3^ | C^1^ | C^2^ | H^1^ | C^3^ | O^4^ |
| --- | --- | --- | --- | --- | --- | --- | --- | --- | --- | --- | --- | --- | --- | --- |
| [(L^1^)Fe^IV^=O]^1+^ | ^5^­TS1_HS_ | 3.058 | 0.059 | 0.061 | -0.042 | -0.025 | 0.017 | 0.433 | 0.009 | 0.004 | 0.380 | -0.036 | -0.009 | 0.055 |
|  | ^3^TS1_IS_ | 1.168 | -0.011 | -0.016 | -0.022 | -0.022 | -0.023 | 0.539 | 0.001 | -0.001 | 0.388 | -0.044 | -0.007 | 0.055 |
|  | ^1^TS1_LS_ | 0.000 | 0.000 | 0.000 | 0.000 | 0.000 | 0.000 | 0.000 | 0.000 | 0.000 | 0.000 | 0.000 | 0.000 | 0.000 |
| [(L^2^)Fe^IV^=O]^1+^ | ^5^­TS1_HS_ | 3.497 | 0.055 | 0.055 | -0.018 | -0.015 | 0.052 | 0.240 | 0.013 | 0.003 | 0.073 | -0.009 | 0.001 | 0.006 |
|  | ^3^TS1_IS_ | 1.172 | -0.014 | -0.019 | -0.019 | -0.020 | -0.021 | 0.525 | 0.011 | 0.000 | 0.382 | -0.038 | -0.009 | 0.062 |
|  | ^1^TS1_LS_ | 0.000 | 0.000 | 0.000 | 0.000 | 0.000 | 0.000 | 0.000 | 0.000 | 0.000 | 0.000 | 0.000 | 0.000 | 0.000 |
| [(L^1^)Fe^IV^=O]^1+^ | ^5^­TS2_HS_ | 3.034 | 0.048 | 0.040 | -0.027 | -0.035 | -0.006 | -0.002 | 0.010 | 0.004 | 0.793 | 0.004 | -0.007 | 0.133 |
|  | ^3^TS2_IS_ | 1.231 | -0.018 | -0.028 | -0.010 | -0.027 | -0.023 | 0.063 | 0.003 | 0.001 | 0.669 | 0.002 | 0.032 | 0.118 |
| [(L^2^)Fe^IV^=O]^1+^ | ^5^­TS2_HS_ | 3.033 | 0.049 | 0.041 | -0.023 | -0.035 | -0.010 | -0.006 | 0.011 | 0.004 | 0.798 | 0.004 | -0.009 | 0.133 |
|  | ^3^TS2_IS_ | 0.930 | -0.013 | -0.009 | -0.015 | -0.019 | -0.013 | 0.145 | 0.003 | 0.004 | 0.893 | -0.029 | -0.031 | 0.162 |

**Table S13.** Spin density values of [(L^1,2^)Fe^IV^=O]^1+^ (TS1 and TS2).

**Table S14.** Structural parameters of [(L^1,2^)Fe^IV^=O]^1+^ (P).

| Bond length (Å) | [(L^1^)Fe^IV^=O]^1+^ | | | [(L^2^)Fe^IV^=O]^1+^ | | |
| --- | --- | --- | --- | --- | --- | --- |
|  | ^5^­P_HS_ | ^3^­P_IS_ | ^1^P_LS_ | ^5^­P_HS_ | ^3^­P_IS_ | ^1^P_LS_ |
| Fe-N^7^ | 2.259 | 2.327 | 2.107 | 2.249 | 2.312 | 2.104 |
| Fe-N^Py1^ | 2.195 | 1.981 | 1.986 | 2.192 | 1.976 | 1.985 |
| Fe-N^Py2^ | 2.200 | 1.978 | 1.987 | 2.192 | 1.977 | 1.984 |
| Fe-N^3^ | 2.209 | 2.040 | 1.997 | 2.228 | 2.057 | 2.002 |
| Fe-O^1^ | 2.019 | 1.996 | 2.043 | 2.009 | 1.997 | 2.038 |
| Fe-O^3^ | 2.292 | 2.373 | 2.035 | 2.301 | 2.377 | 2.034 |

**Table S15.** Structural parameters of [(L^1,2^)Fe^IV^=O]^1+^ (P)

|  | [(L^1^)Fe^IV^=O]^1+^ | | | [(L^2^)Fe^IV^=O]^1+^ | | |
| --- | --- | --- | --- | --- | --- | --- |
| Bond angle (°) | ^5^P_HS_ | ^3^­P_IS_ | ^1^P_LS_ | ^5^­P_HS_ | ^3^­P_IS_ | ^1^P_LS_ |
| N^Py1-^Fe-N^Py2^ | 151.0 | 165.5 | 165.4 | 151.3 | 165.7 | 165.9 |
| N^Py1^-Fe-N^3^ | 76.3 | 83.3 | 83.6 | 76.3 | 83.4 | 83.8 |
| N^Py1-^Fe-N^7^ | 92.6 | 91.6 | 93.2 | 92.1 | 92.0 | 93.0 |
| N^Py1-^Fe-O^1^ | 103.5 | 95.4 | 95.5 | 101.7 | 95.8 | 95.3 |
| N^Py1-^Fe-O^3^ | 89.3 | 89.6 | 88.2 | 89.1 | 88.5 | 88.3 |
| O^1^-Fe-O^3^ | 61.3 | 60.1 | 64.7 | 61.3 | 60.0 | 64.8 |
| N^3^-Fe-N^7^ | 82.0 | 83.8 | 88.2 | 82.0 | 83.9 | 88.4 |

**Table S16.** Structural parameters of [(L^1,2^)Fe^V^=O]^2+^ (R).

|  | [(L^1^)Fe^V^=O]^2+^ | | [(L^2^)Fe^V^=O]^2+^ | |
| --- | --- | --- | --- | --- |
| Bond length (Å) | ^4^R_HS_ | ^2^R_IS_ | ^4^R_HS_ | ^2^R_IS_ |
| Fe-N^7^ | 2.194 | 2.217 | 2.206 | 2.103 |
| Fe-N^Py1^ | 1.960 | 1.967 | 1.967 | 1.974 |
| Fe-N^Py2^ | 1.957 | 1.983 | 1.979 | 1.967 |
| Fe-N^3^ | 2.061 | 2.051 | 2.065 | 2.099 |
| Fe-O^1^ | 1.844 | 1.851 | 1.848 | 2.038 |
| Fe-O^2^ | 1.682 | 1.677 | 1.671 | 1.622 |

**Table S17.** Structural parameters of [(L^1,2^)Fe^V^=O]^2+^ (INT).

|  | [(L^1^)Fe^V^=O]^2+^ | | | [(L^2^)Fe^V^=O]^2+^ | | |
| --- | --- | --- | --- | --- | --- | --- |
| Bond length (Å) | ^6^INT_HS_ | ^4^­INT_IS_ | ^2^INT_LS_ | ^6^­INT_HS_ | ^4^­INT_IS_ | ^2^INT_LS_ |
| Fe-N^7^ | 2.310 | 2.217 | 2.161 | 2.29410 | 2.203 | 2.310 |
| Fe-N^Py1^ | 2.147 | 1.968 | 1.937 | 2.14732 | 1.968 | 1.974 |
| Fe-N^Py2^ | 2.171 | 1.990 | 1.976 | 2.16863 | 1.986 | 1.986 |
| Fe-N^3^ | 2.130 | 2.044 | 2.069 | 2.15536 | 2.055 | 2.043 |
| Fe-O^1^ | 1.825 | 1.866 | 1.955 | 1.81822 | 1.867 | 1.753 |
| Fe-O^2^ | 1.773 | 1.753 | 1.713 | 1.77563 | 1.750 | 1.735 |
| O^2^-H^1^ | 0.976 | 0.977 | 0.986 | 0.97648 | 0.977 | 0.977 |

**Table S18.** Structural parameters of [(L^1,2^)Fe^V^=O]^2+^ (R).

| Bond angle (°) | [(L^1^)Fe^V^=O]^2+^ | | [(L^2^)Fe^V^=O]^2+^ | |
| --- | --- | --- | --- | --- |
|  | ^4^R_HS_ | ^2^R_IS_ | ^4^R_HS_ | ^2^R_IS_ |
| N^Py1-^Fe-N^Py2^ | 165.6 | 163.2 | 163.3 | 162.4 |
| N^Py1^-Fe-N^3^ | 82.9 | 82.5 | 82.6 | 81.8 |
| N^Py1-^Fe-N^7^ | 86.1 | 91.1 | 91.1 | 92.9 |
| N^Py1-^Fe-O^1^ | 87.4 | 94.8 | 94.7 | 90.3 |
| N^Py1-^Fe-O^2^ | 97.3 | 97.6 | 97.4 | 97.3 |
| O^1^-Fe-O^2^ | 93.4 | 91.9 | 92.6 | 89.5 |
| N^3^-Fe-N^7^ | 86.1 | 85.4 | 85.7 | 87.4 |

**Table S19.** Structural parameters of [(L^1,2^)Fe^V^=O]^2+^ (INT).

| Bond angle (°) | [(L^1^)Fe^V^=O]^2+^ | | | [(L^2^)Fe^V^=O]^2+^ | | |
| --- | --- | --- | --- | --- | --- | --- |
|  | ^6^INT_HS_ | ^4^­INT_IS_ | ^2^INT_LS_ | ^6^­INT_HS_ | ^4^­INT_IS_ | ^2^INT_LS_ |
| N^Py1-^Fe-N^Py2^ | 155.7 | 163.6 | 152.8 | 155.2 | 163.8 | 167.1 |
| N^Py1^-Fe-N^3^ | 78.5 | 82.7 | 79.5 | 78.2 | 82.8 | 84.2 |
| N^Py1-^Fe-N^7^ | 89.1 | 90.8 | 95.5 | 88.9 | 90.8 | 89.1 |
| N^Py1-^Fe-O^1^ | 93.5 | 95.0 | 89.9 | 93.6 | 95.0 | 93.9 |
| N^Py1-^Fe-O^2^ | 101.4 | 97.2 | 104.2 | 101.5 | 97.0 | 91.5 |
| O^1^-Fe-O^2^ | 92.9 | 89.2 | 82.1 | 93.0 | 89.1 | 101.2 |
| Fe-O^2^-H^1^ | 111.6 | 108.6 | 106.0 | 111.9 | 108.5 | 113.2 |
| N^3^-Fe-N^7^ | 82.0 | 85.2 | 82.1 | 82.3 | 85.5 | 82.6 |

**Table S20.** Mulliken charges of [(L^1,2^)Fe^V^=O]^2+^ (R and INT).

|  | | Fe | N^Py1^ | N^Py2^ | N^3^ | N^7^ | O^1^ | O^2^ | C^1^ | H^1^ |
| --- | --- | --- | --- | --- | --- | --- | --- | --- | --- | --- |
| [(L^1^)Fe^V^=O]^2+^ | ^4^R_HS_ | -0.004 | 0.109 | 0.098 | -0.053 | -0.002 | -0.367 | -0.291 | 0.372 | - |
|  | ^2^R_LS_ | 0.019 | 0.093 | 0.103 | -0.094 | 0.009 | -0.345 | -0.296 | 0.390 | - |
| [(L^2^)Fe^V^=O]^2+^ | ^4^R_HS_ | 0.015 | 0.089 | 0.103 | -0.109 | 0.021 | -0.339 | -0.289 | 0.391 | - |
|  | ^2^R_LS_ | 0.114 | 0.026 | 0.061 | -0.165 | 0.068 | -0.348 | -0.465 | 0.450 | - |
| [(L^1^)Fe^V^=O]^2+^ | ^6^­INT_HS_ | 0.506 | -0.121 | -0.104 | -0.152 | -0.081 | -0.425 | -0.531 | 0.397 | 0.367 |
|  | ^4^­INT_IS_ | 0.021 | 0.098 | 0.123 | -0.103 | -0.009 | -0.421 | -0.514 | 0.397 | 0.368 |
|  | ^2^INT_LS_ | -0.037 | 0.157 | 0.125 | -0.146 | 0.031 | -0.471 | -0.409 | 0.391 | 0.366 |
| [(L^2^)Fe^V^=O]^2+^ | ^6^­INT_HS_ | 0.504 | -0.124 | -0.110 | -0.175 | -0.070 | -0.416 | -0.524 | 0.398 | 0.369 |
|  | ^4^­INT_IS_ | 0.006 | 0.096 | 0.124 | -0.118 | 0.003 | -0.419 | -0.502 | 0.398 | 0.372 |
|  | ^2^INT_LS_ | 0.053 | 0.058 | 0.086 | -0.046 | -0.038 | -0.345 | -0.465 | 0.359 | 0.359 |

**Table S21.** Spin density values of [(L^1,2^)Fe^V^=O]^2+^ (R and INT).

|  | | Fe | N^Py1^ | N^Py2^ | N^3^ | N^7^ | O^1^ | O^2^ | C^1^ | H^1^ |
| --- | --- | --- | --- | --- | --- | --- | --- | --- | --- | --- |
| [(L^1^)Fe^V^=O]^2+^ | ^4^R_HS_ | 1.850 | -0.033 | -0.030 | -0.050 | -0.045 | 0.014 | 1.081 | -0.018 | - |
|  | ^2^R_LS_ | 0.807 | 0.000 | 0.002 | -0.040 | -0.063 | -0.132 | 0.455 | 0.010 | - |
| [(L^2^)Fe^V^=O]^2+^ | ^4^R_HS_ | 0.816 | 0.001 | 0.001 | -0.038 | -0.063 | -0.144 | 0.454 | 0.010 | - |
|  | ^2^R_LS_ | 1.226 | -0.051 | -0.015 | -0.024 | -0.041 | -0.026 | 0.858 | 0.028 | - |
| [(L^1^)Fe^V^=O]^2+^ | ^6^INT_HS_ | 3.622 | 0.105 | 0.092 | -0.085 | -0.107 | 0.176 | 0.840 | -0.003 | 0.012 |
|  | ^4^INT_IS_ | 2.007 | -0.043 | -0.034 | -0.051 | -0.075 | -0.013 | 0.800 | -0.002 | 0.012 |
|  | ^2^INT_LS_ | 0.290 | 0.010 | -0.024 | -0.024 | -0.040 | -0.021 | 0.460 | 0.001 | 0.012 |
| [(L^2^)Fe^V^=O]^2+^ | ^6^­INT_HS_ | 3.615 | 0.108 | 0.098 | -0.069 | -0.116 | 0.172 | 0.133 | -0.003 | 0.012 |
|  | ^4^­INT_IS_ | 1.993 | -0.042 | -0.033 | -0.049 | -0.072 | -0.012 | 0.203 | -0.002 | 0.012 |
|  | ^2^INT_LS_ | 0.320 | 0.016 | -0.030 | -0.020 | -0.030 | 0-.022 | 0.523 | -0.003 | 0.012 |

**Table S22.** Structural parameters of [(L^1,2^)Fe^V^=O]^2+^ (TS1).

|  | [(L^1^)Fe^V^=O]^2+^ | | [(L^2^)Fe^V^=O]^2+^ | |
| --- | --- | --- | --- | --- |
| Bond length (Å) | ^4^TS1_HS_ | ^2^TS1_IS_ | ^4^TS1_HS_ | ^2^TS1_IS_ |
| Fe-N^7^ | 2.149 | 2.143 | 2.135 | 2.139 |
| Fe-N^Py1^ | 1.974 | 1.979 | 1.986 | 1.983 |
| Fe-N^Py2^ | 1.977 | 1.993 | 1.966 | 1.982 |
| Fe-N^3^ | 2.025 | 2.038 | 2.034 | 2.035 |
| Fe-O^1^ | 1.923 | 1.948 | 1.932 | 1.938 |
| Fe-O^2^ | 1.847 | 1.764 | 1.847 | 1.810 |
| O^2^-H^1^ | 1.250 | 1.228 | 1.232 | 1.255 |
| H^1^-C^2^ | 1.305 | 1.335 | 1.313 | 1.310 |

**Table S23.** Structural parameters of [(L^1,2^)Fe^V^=O]^2+^ (TS2).

|  | [(L^1^)Fe^V^=O]^2+^ | | | [(L^2^)Fe^V^=O]^2+^ | | |
| --- | --- | --- | --- | --- | --- | --- |
| Bond length (Å) | ^6^TS2_HS_ | ^4^­TS2_IS_ | ^2^TS2_LS_ | ^6^TS2_HS_ | ^4^­TS2_S_ | ^2^TS2_LS_ |
| Fe-N^7^ | 2.325 | 2.203 | 2.175 | 2.306 | 2.188 | 2.174 |
| Fe-N^Py1^ | 2.153 | 1.985 | 1.978 | 2.157 | 1.985 | 2.129 |
| Fe-N^Py2^ | 2.138 | 1.996 | 2.003 | 2.140 | 1.992 | 2.169 |
| Fe-N^3^ | 2.143 | 2.104 | 2.060 | 2.162 | 2.113 | 2.134 |
| Fe-O^1^ | 1.839 | 1.910 | 1.911 | 1.835 | 1.912 | 1.835 |
| Fe-O^2^ | 1.796 | 1.748 | 1.706 | 1.795 | 1.741 | 1.795 |
| O^2^-H^1^ | 0.995 | 1.037 | 1.072 | 0.996 | 1.041 | 0.996 |
| O^2^-C^2^ | 2.605 | 2.467 | 2.815 | 2.626 | 2.493 | 2.801 |

**Table S24.** Structural parameters of [(L^1,2^)Fe^V^=O]^2+^ (TS1).

| Bond angle (°) | [(L^1^)Fe^V^=O]^2+^ | | [(L^2^)Fe^V^=O]^2+^ | |
| --- | --- | --- | --- | --- |
|  | ^4^TS1_HS_ | ^2^TS1_LS_ | ^4^TS1_HS_ | ^2^TS1_LS_ |
| N^Py1-^Fe-N^Py2^ | 162.9 | 162.6 | 162.3 | 161.8 |
| N^Py1^-Fe-N^3^ | 82.8 | 82.6 | 82.3 | 82.3 |
| N^Py1-^Fe-N^7^ | 95.0 | 93.6 | 97.2 | 94.9 |
| N^Py1-^Fe-O^1^ | 86.7 | 86.4 | 86.5 | 86.5 |
| N^Py1-^Fe-O^2^ | 103.0 | 103.3 | 102.0 | 104.2 |
| O^1^-Fe-O^2^ | 88.0 | 87.8 | 87.3 | 88.4 |
| Fe-O^2^-H^1^ | 129.1 | 130.8 | 126.3 | 127.1 |
| N^3^-Fe-N^7^ | 86.8 | 86.6 | 87.3 | 86.8 |
| O^2^-H^1^-C^2^ | 165.7 | 164.6 | 170.3 | 164.3 |

**Table S25.** Structural parameters of [(L^1,2^)Fe^V^=O]^2+^ (TS2).

|  | [(L^1^)Fe^V^=O]^2+^ | | | [(L^2^)Fe^V^=O]^2+^ | | |
| --- | --- | --- | --- | --- | --- | --- |
| Bond length (Å) | ^6^TS2_HS_ | ^4^­TS2_IS_ | ^2^TS2_LS_ | ^6^TS2_HS_ | ^4^­TS2_S_ | ^2^TS2_LS_ |
| N^Py1-^Fe-N^Py2^ | 155.1 | 160.5 | 161.2 | 154.5 | 160.6 | 154.3 |
| N^Py1^-Fe-N^3^ | 77.3 | 80.8 | 81.5 | 77.0 | 80.8 | 78.2 |
| N^Py1-^Fe-N^7^ | 90.0 | 92.9 | 93.0 | 90.7 | 93.4 | 95.2 |
| N^Py1-^Fe-O^1^ | 87.4 | 85.0 | 84.8 | 87.7 | 85.1 | 84.1 |
| N^Py1-^Fe-O^2^ | 104.8 | 98.9 | 102.4 | 104.7 | 98.8 | 104.7 |
| O^1^-Fe-O^2^ | 92.3 | 93.7 | 93.0 | 92.5 | 93.8 | 92.5 |
| Fe-O^2^-H^1^ | 107.0 | 104.4 | 107.9 | 107.4 | 104.6 | 107.4 |
| N^3^-Fe-N^7^ | 81.4 | 84.8 | 85.7 | 81.6 | 85.0 | 85.4 |
| Fe-O^2^-C^2^ | 174.9 | 161.7 | 142.2 | 175.3 | 161.7 | 175.3 |

**Table S26.** Mulliken charges of [(L^1,2^)Fe^V^=O]^2+^ (TS1 and TS2).

|  | | Fe | N^Py1^ | N^Py2^ | N^3^ | N^7^ | O^1^ | O^2^ | O^3^ | C^1^ | C^2^ | H^1^ | C^3^ | O^4^ |
| --- | --- | --- | --- | --- | --- | --- | --- | --- | --- | --- | --- | --- | --- | --- |
| [(L^1^)Fe^V^=O]^2+^ | ^4^TS1_HS_ | 0.022 | 0.098 | 0.084 | -0.027 | 0.010 | -0.383 | -0.418 | -0.317 | 0.415 | -0.301 | 0.224 | 0.316 | -0.354 |
|  | ^2^TS1_IS_ | 0.077 | 0.101 | 0.080 | -0.058 | 0.036 | -0.368 | -0.491 | -0.236 | 0.423 | -0.319 | 0.221 | 0.313 | -0.364 |
| [(L^2^)Fe^V^=O]^2+^ | ^4^TS1_HS_ | 0.002 | 0.092 | 0.087 | -0.053 | 0.022 | -0.385 | -0.417 | -0.312 | 0.425 | -0.324 | 0.270 | 0.296 | -0.359 |
|  | ^2^TS1_IS_ | 0.089 | -0.068 | 0.036 | -0.375 | -0.461 | -0.252 | 0.435 | -0.320 | 0.229 | 0.318 | -0.354 | 0.318 | -0.354 |
| [(L^1^)Fe^V^=O]^2+^ | ^6^TS2_HS_ | 0.551 | -0.092 | -0.108 | -0.174 | -0.092 | -0.450 | -0.553 | -0.377 | 0.406 | -0.195 | 0.358 | 0.387 | -0.412 |
|  | ^4^TS2_IS_ | 0.149 | 0.090 | 0.092 | -0.110 | -0.001 | -0.393 | -0.583 | 0.174 | -0.363 | -0.186 | 0.363 | -0.186 | 0.315 |
|  | ^2^TS2_LS_ | 0.108 | 0.102 | 0.097 | -0.094 | 0.033 | -0.393 | -0.580 | -0.451 | 0.433 | -0.217 | 0.390 | 0.311 | -0.397 |
| [(L^2^)Fe^V^=O]^2+^ | ^6^TS2_HS_ | 0.557 | -0.097 | -0.120 | -0.189 | -0.089 | -0.444 | -0.553 | -0.374 | 0.408 | -0.191 | 0.361 | 0.390 | -0.348 |
|  | ^4^TS2_IS_ | 0.146 | 0.105 | -0.039 | -0.144 | 0.007 | -0.397 | -0.593 | -0.453 | 0.432 | -0.179 | 0.374 | 0.315 | -0.395 |
|  | ^2^TS2_LS_ | 0.111 | 0.101 | 0.099 | -0.092 | 0.032 | -0.389 | -0.588 | -0.469 | 0.443 | -0.223 | 0.399 | 0.315 | -0.391 |

**Table S27.** Spin density values of [(L^1,2^)Fe^V^=O]^2+^ (TS1 and TS2).

|  | | Fe | N^Py1^ | N^Py2^ | N^3^ | N^7^ | O^1^ | O^2^ | O^3^ | C^1^ | C^2^ | H^1^ | C^3^ | O^4^ |
| --- | --- | --- | --- | --- | --- | --- | --- | --- | --- | --- | --- | --- | --- | --- |
| [(L^1^)Fe^V^=O]^2+^ | ^4^TS1_HS_ | 1.311 | -0.033 | -0.037 | -0.040 | -0.041 | 0.004 | 0.970 | 0.357 | -0.026 | 0.420 | -0.016 | -0.022 | 0.072 |
|  | ^2^TS1_IS_ | 1.269 | -0.022 | -0.026 | -0.033 | -0.050 | -0.032 | 0.009 | -0.511 | 0.032 | 0.384 | -0.010 | -0.018 | 0.066 |
| [(L^2^)Fe^V^=O]^2+^ | ^4^TS1_HS_ | 1.269 | -0.031 | -0.038 | -0.037 | -0.040 | 0.003 | 0.956 | 0.370 | -0.026 | 0.471 | -0.016 | -0.018 | 0.046 |
|  | ^2^TS1_IS_ | 1.259 | -0.024 | -0.028 | -0.032 | -0.052 | -0.031 | -0.023 | -0.482 | 0.030 | 0.391 | -0.008 | -0.018 | 0.070 |
| [(L^1^)Fe^V^=O]^2+^ | ^6^TS2_HS_ | 3.755 | 0.084 | 0.093 | -0.025 | -0.041 | 0.183 | 0.010 | 0.033 | -0.001 | 0.792 | 0.011 | -0.018 | 0.105 |
|  | ^4^TS2_IS_ | 2.091 | -0.036 | -0.042 | -0.033 | -0.079 | -0.041 | 0.174 | 0.012 | -0.006 | 0.838 | -0.001 | 0.838 | -0.019 |
|  | ^2^TS2_LS_ | 1.796 | -0.033 | -0.026 | -0.046 | -0.065 | -0.031 | 0.346 | 0.036 | -0.002 | -0.877 | -0.002 | 0.032 | -0.153 |
| [(L^2^)Fe^V^=O]^2+^ | ^6^TS2_HS_ | 3.760 | 0.085 | 0.098 | -0.017 | -0.043 | 0.184 | 0.002 | 0.032 | -0.001 | 0.783 | 0.011 | -0.019 | 0.025 |
|  | ^4^TS2_IS_ | 2.080 | -0.039 | -0.034 | -0.023 | -0.067 | -0.043 | 0.189 | 0.028 | 0.000 | 0.828 | 0.007 | -0.025 | 0.135 |
|  | ^2^TS2_LS_ | 1.756 | -0.031 | -0.025 | -0.044 | -0.055 | -0.032 | 0.356 | 0.033 | -0.001 | -0.867 | -0.003 | 0.033 | -0.155 |

**Table S28.** Structural parameters of [(L^1,2^)Fe^V^=O]^2+^ (P).

| Bond length (Å) | [(L^1^)Fe^IV^=O]^1+^ | | | [(L^2^)Fe^IV^=O]^1+^ | | |
| --- | --- | --- | --- | --- | --- | --- |
|  | ^5^­P_HS_ | ^3^­P_IS_ | ^1^P_LS_ | ^5^­P_HS_ | ^3^­P_IS_ | ^1^P_LS_ |
| Fe-N^7^ | 2.174 | 2.245 | 2.084 | 2.164 | 2.321 | 2.075 |
| Fe-N^Py1^ | 2.125 | 1.993 | 1.985 | 2.121 | 1.989 | 1.983 |
| Fe-N^Py2^ | 2.125 | 1.993 | 1.985 | 2.116 | 1.985 | 1.979 |
| Fe-N^3^ | 2.161 | 1.983 | 1.981 | 2.182 | 2.023 | 1.992 |
| Fe-O^1^ | 1.945 | 1.939 | 1.960 | 1.940 | 1.940 | 1.955 |
| Fe-O^3^ | 2.265 | 2.232 | 1.966 | 2.267 | 2.381 | 1.966 |

**Table S29.** Structural parameters of [(L^1,2^)Fe^V^=O]^2+^ (P)

|  | [(L^1^)Fe^IV^=O]^1+^ | | | [(L^2^)Fe^IV^=O]^1+^ | | |
| --- | --- | --- | --- | --- | --- | --- |
| Bond angle (°) | ^5^P_HS_ | ^3^­P_IS_ | ^1^P_LS_ | ^5^­P_HS_ | ^3^­P_IS_ | ^1^P_LS_ |
| N^Py1-^Fe-N^Py2^ | 153.6 | 165.3 | 164.1 | 153.7 | 166.1 | 164.8 |
| N^Py1^-Fe-N^3^ | 78.0 | 83.6 | 83.2 | 77.9 | 83.6 | 83.4 |
| N^Py1-^Fe-N^7^ | 78.0 | 93.1 | 93.9 | 94.0 | 91.9 | 93.6 |
| N^Py1-^Fe-O^1^ | 99.1 | 95.5 | 95.9 | 98.9 | 95.2 | 95.6 |
| N^Py1-^Fe-O^3^ | 85.9 | 88.3 | 87.6 | 86.2 | 89.0 | 87.7 |
| O^1^-Fe-O^3^ | 80.4 | 85.2 | 84.7 | 80.7 | 85.7 | 85.0 |
| N^3^-Fe-N^7^ | 85.2 | 86.3 | 87.9 | 85.2 | 84.7 | 88.2 |

*O trans to N^7^ isomer results*


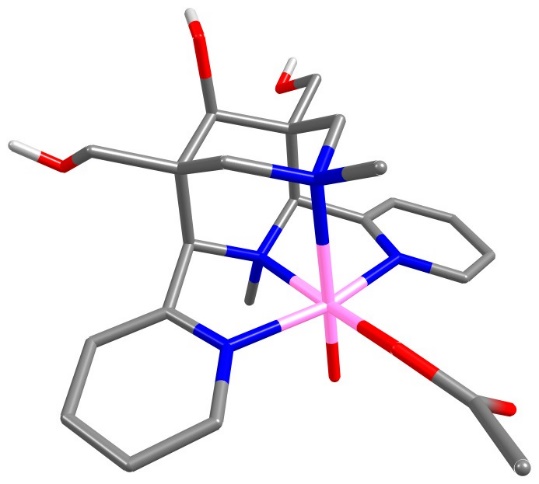

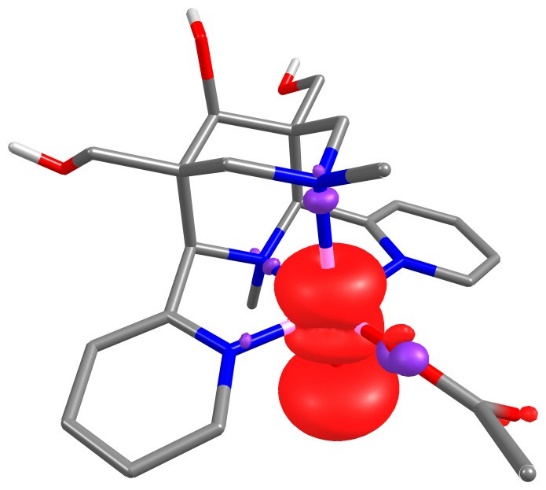


**Figure S15.** B3LYP-D3 optimized structure and spin density plot of the reactant (^5^R_HS_) of [(L^1^)Fe^IV^=O]^1+^  (O trans to N^7^).


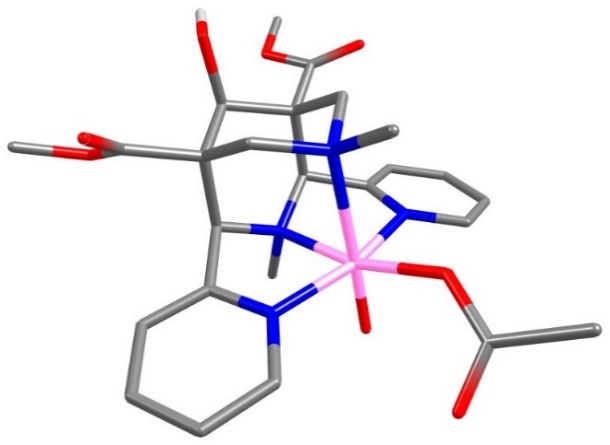

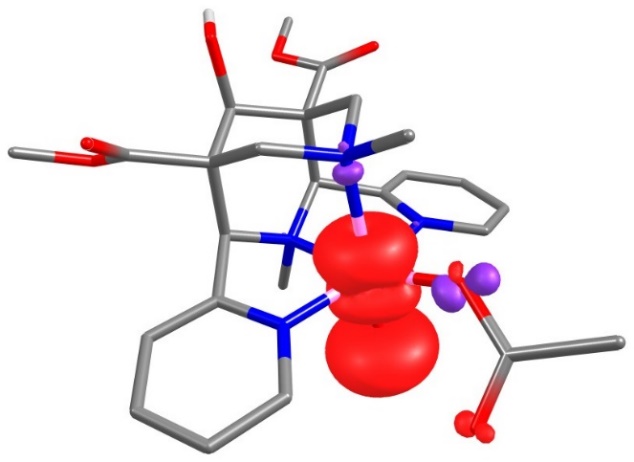


**Figure S16.** B3LYP-D3 optimized structure and spin density plot of the reactant (^5^R_HS_) of [(L^2^)Fe^IV^=O]^1+^  (O trans to N^7^).


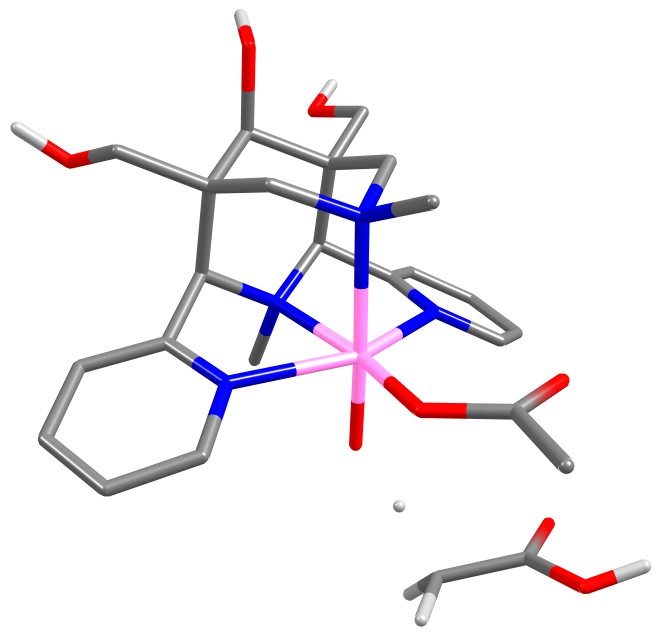

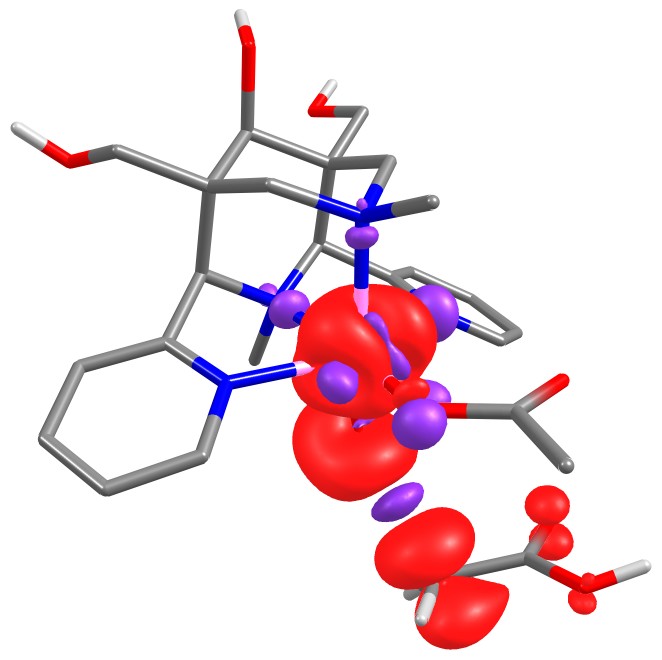


**Figure S17.** B3LYP-D3 optimized structure and spin density plot of the reactant (^5^TS1_HS_) of [(L^1^)Fe^IV^=O]^1+^  (O trans to N^7^).


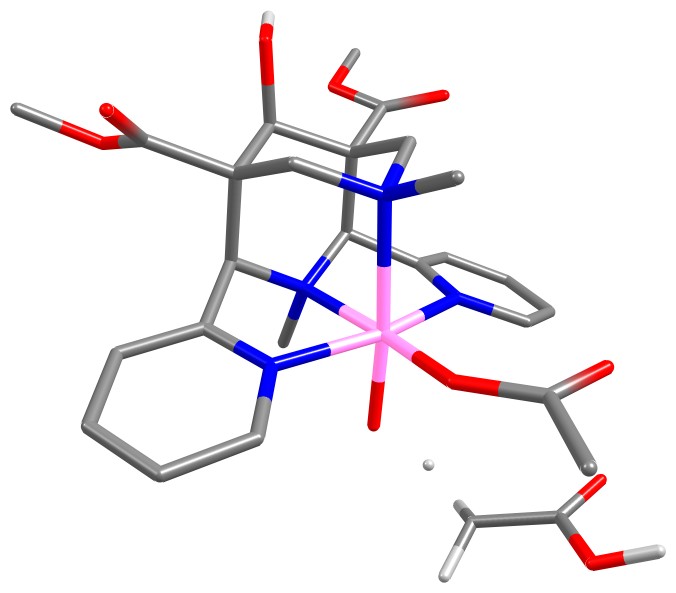

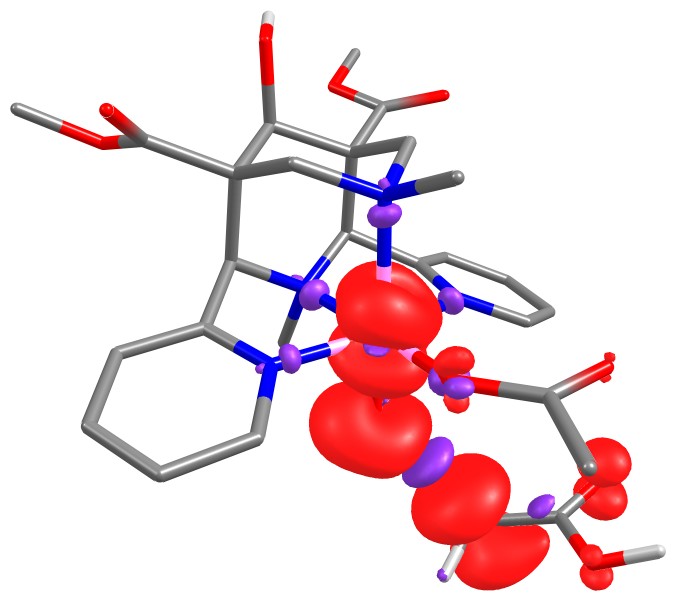


**Figure S18.** B3LYP-D3 optimized structure and spin density plot of the reactant (^5^TS1_HS_) of [(L^2^)Fe^IV^=O]^1+^  (O trans to N^7^).


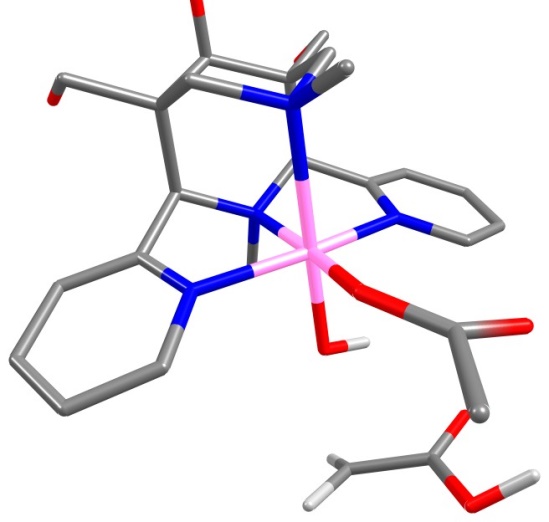

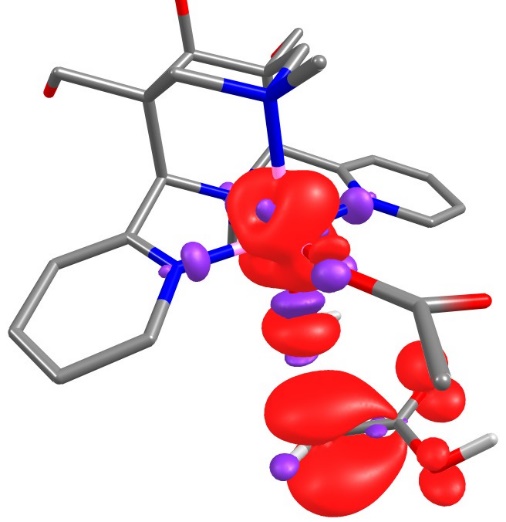

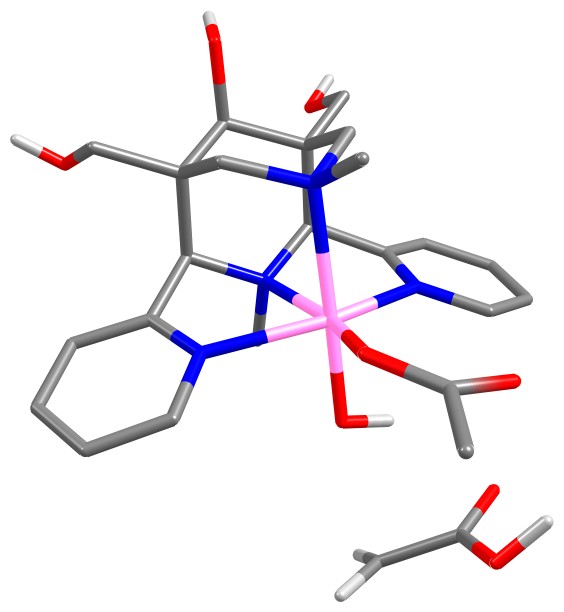

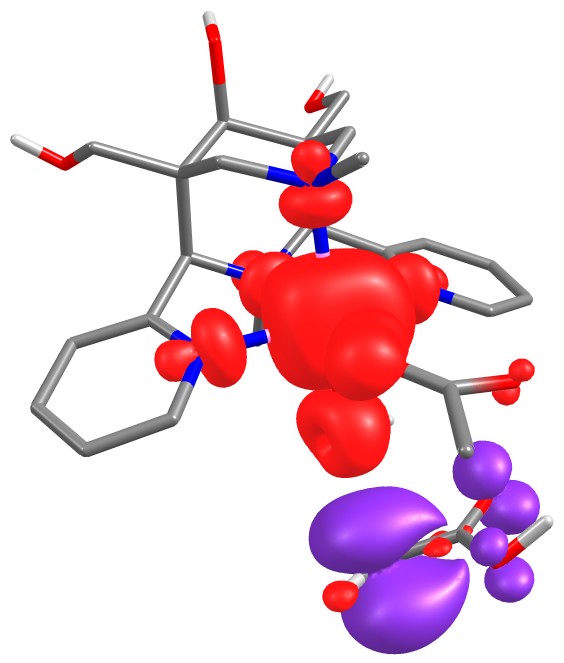


**Figure S19.** B3LYP-D3 optimized structures and spin density plots of the TS2 of [(L^1^)Fe^IV^=O]^1+^ (O trans to N^7^). ^3^TS2_IS_ (top) and ^5^TS2_HS_ (bottom).


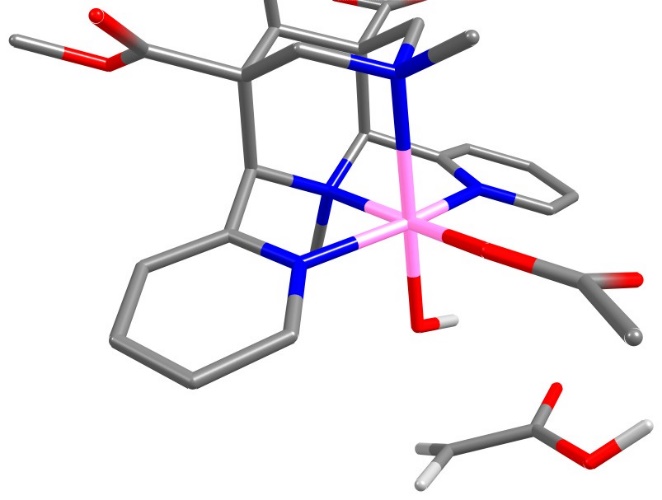

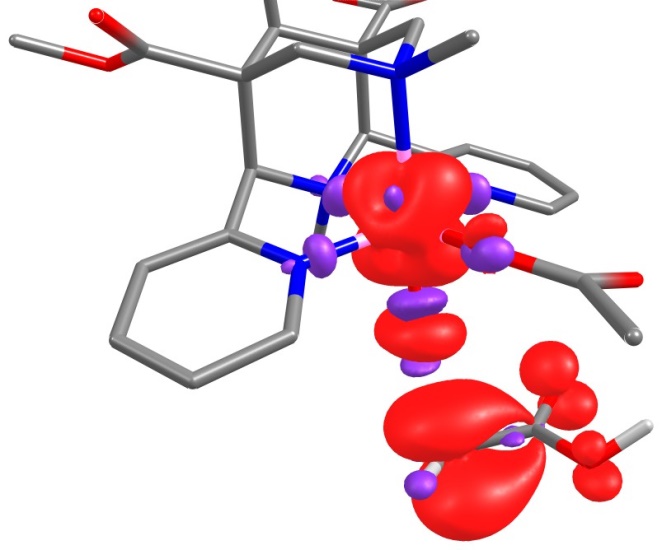


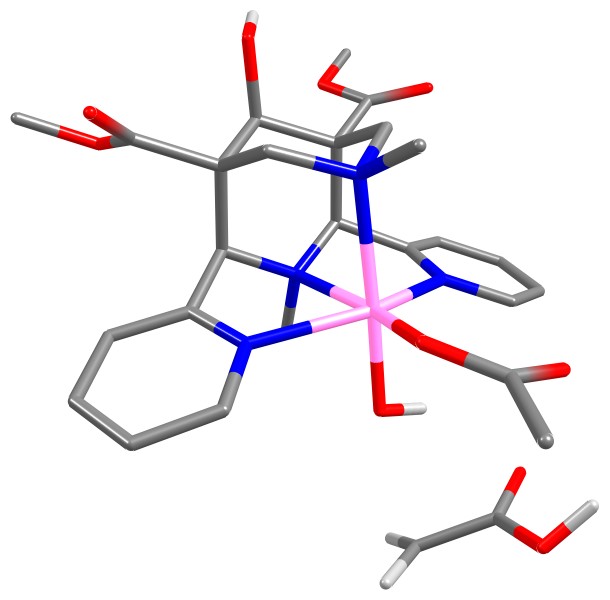

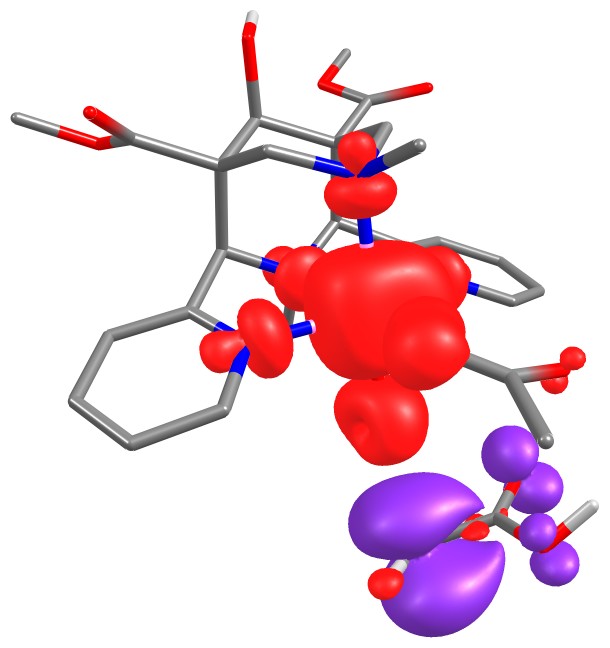


**Figure S20.** B3LYP-D3 optimized structures and spin density plots of the TS2 of [(L^2^)Fe^IV^=O]^1+^ (O trans to N^7^). ^3^TS2_IS_ (top) and ^5^TS2_HS_ (bottom).


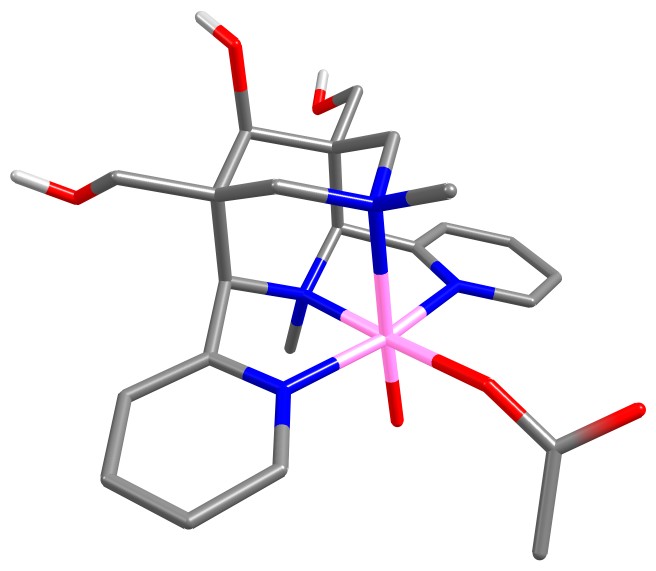

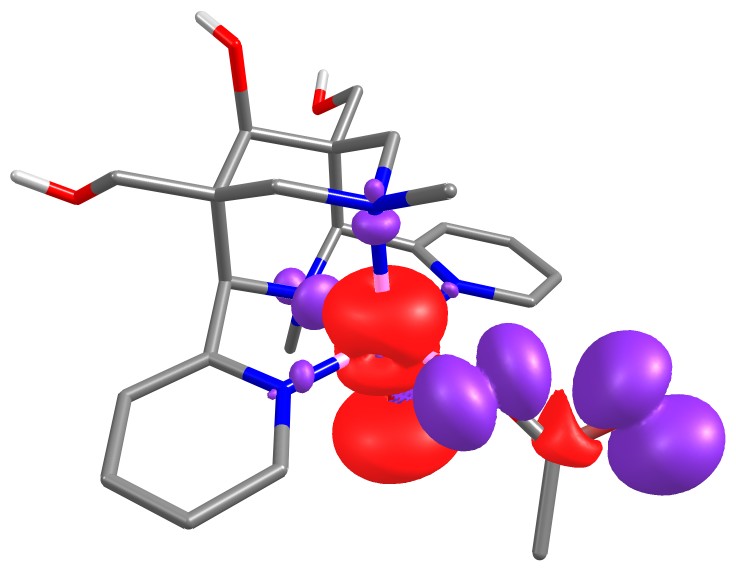


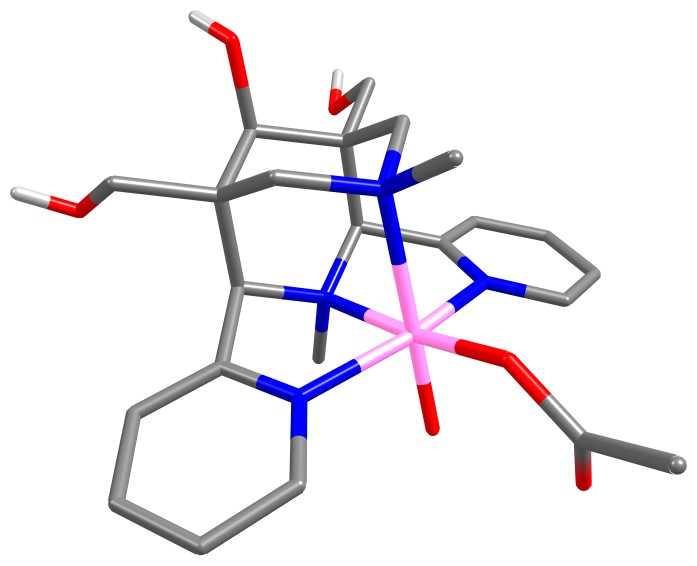

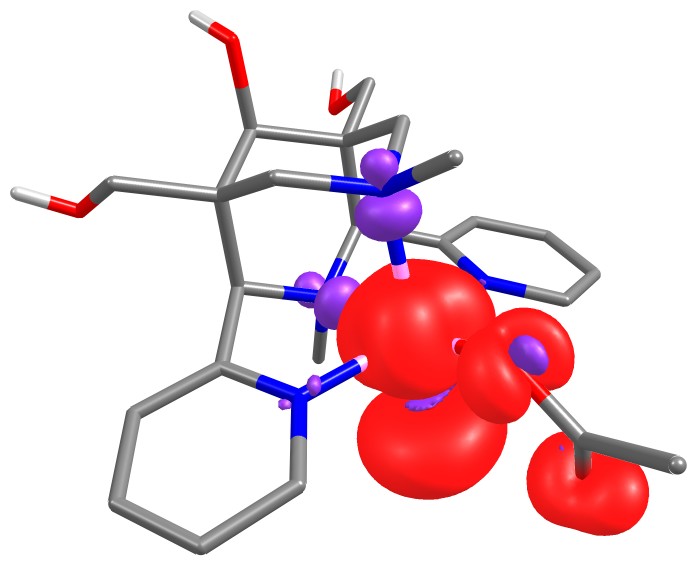


**Figure S21.** B3LYP-D3 optimized structures and spin density plots of the R of [(L^1^)Fe^V^=O]^2+^ (O trans to N^7^). ^2^R_LS_ (top) and ^4^R_HS_ (bottom).


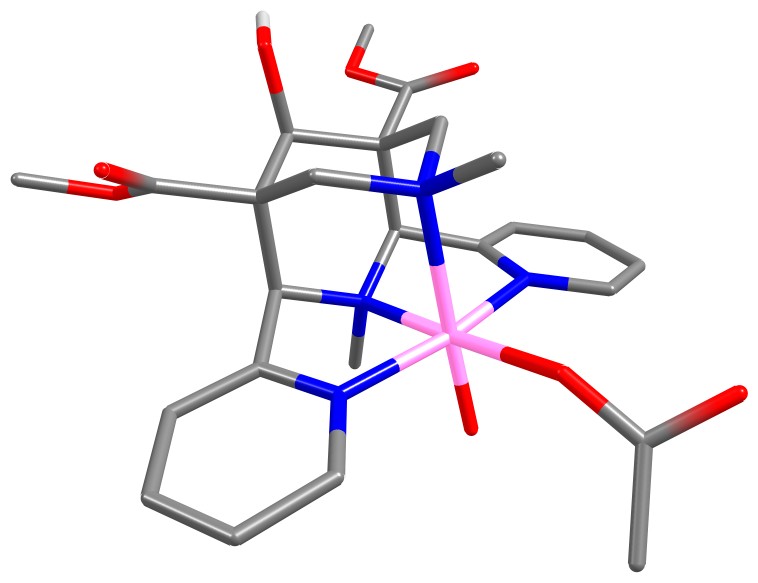

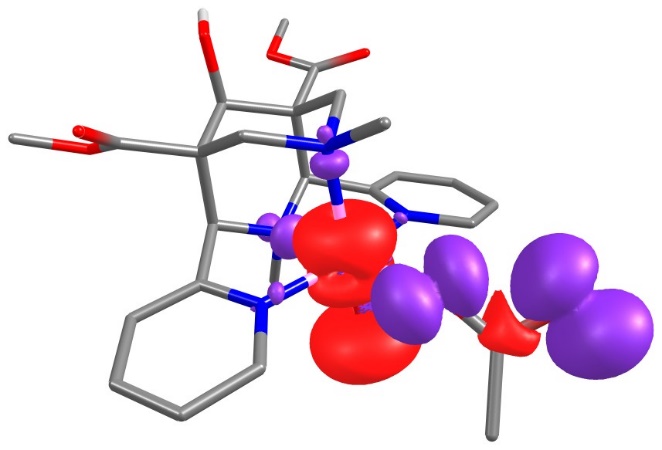


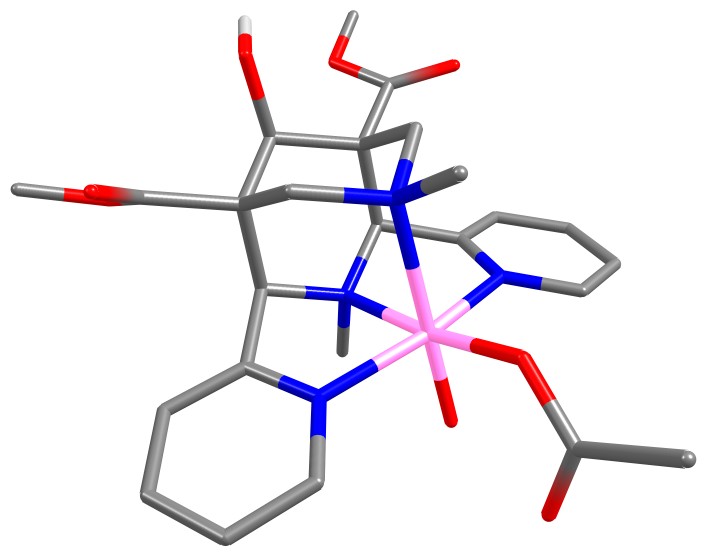

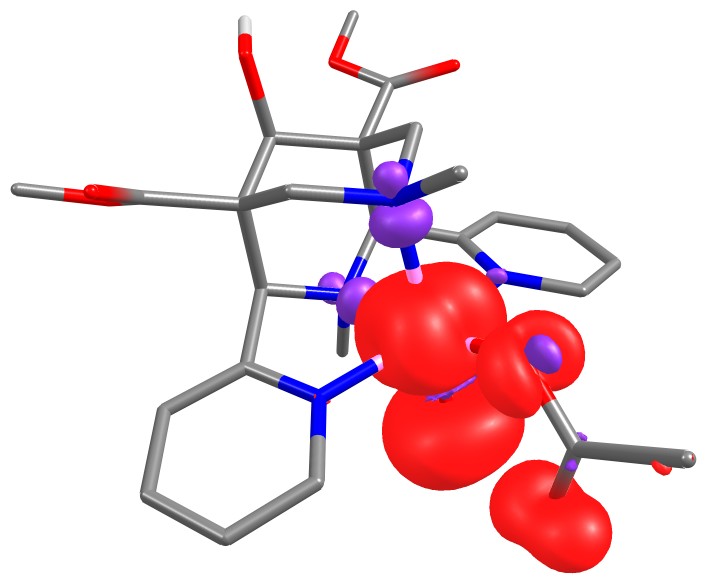


**Figure S22.** B3LYP-D3 optimized structures and spin density plots of the R of [(L^2^)Fe^V^=O]^2+^ (O trans to N^7^). ^2^R_LS_ (top) and ^4^R_HS_ (bottom).


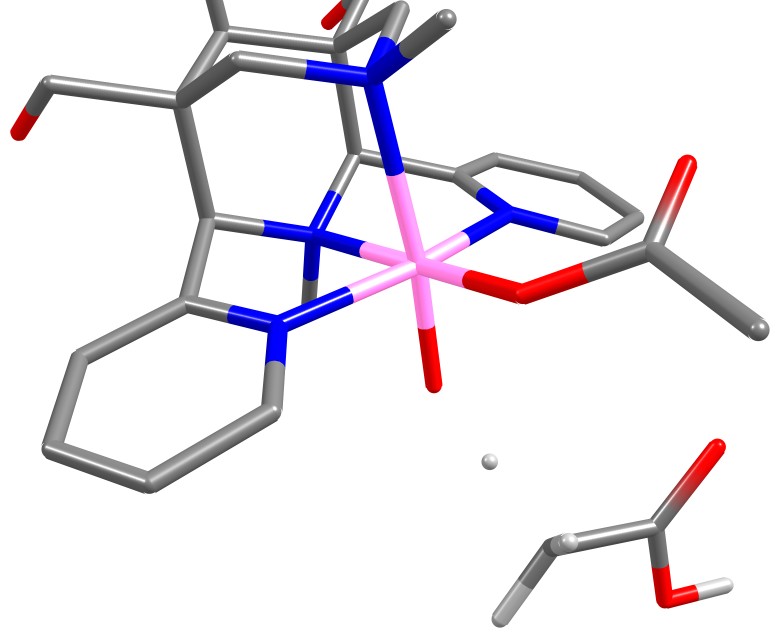

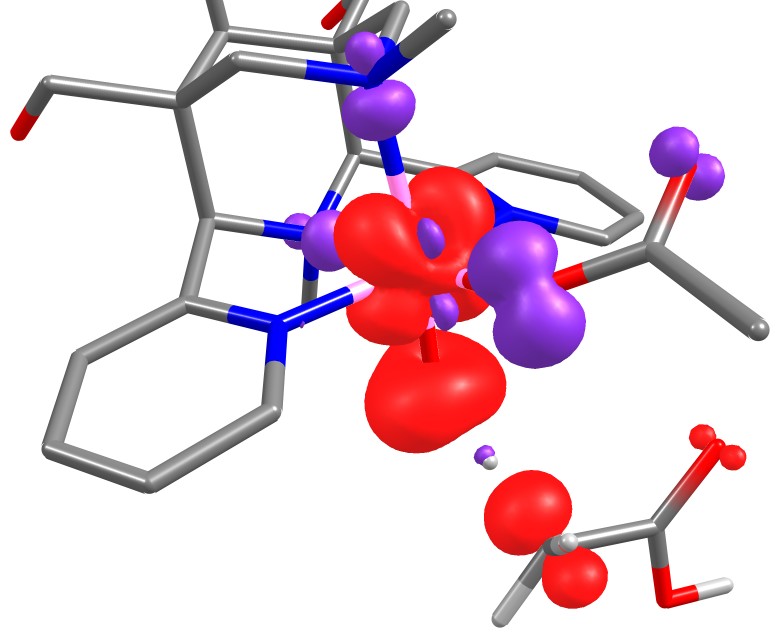


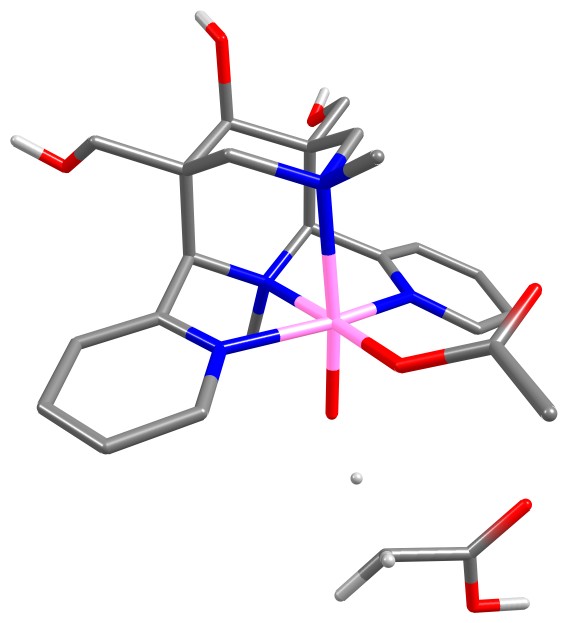

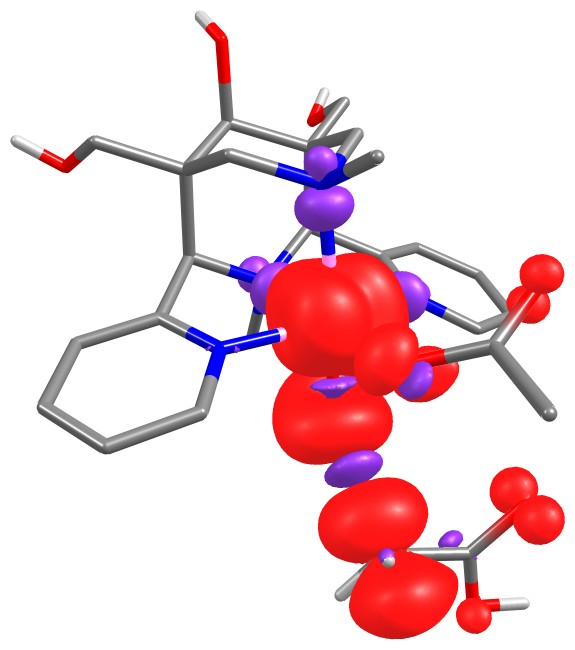


**Figure S23.** B3LYP-D3 optimized structure and spin density plot of the TS1 of [(L^1^)Fe^V^=O]^2+^ (O trans to N^7^). ^2^TS1_LS_ (top) and ^4^TS1_HS_ (bottom).


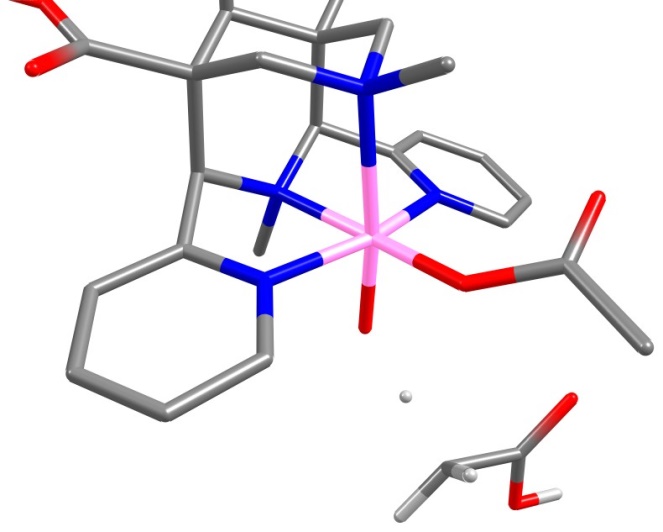

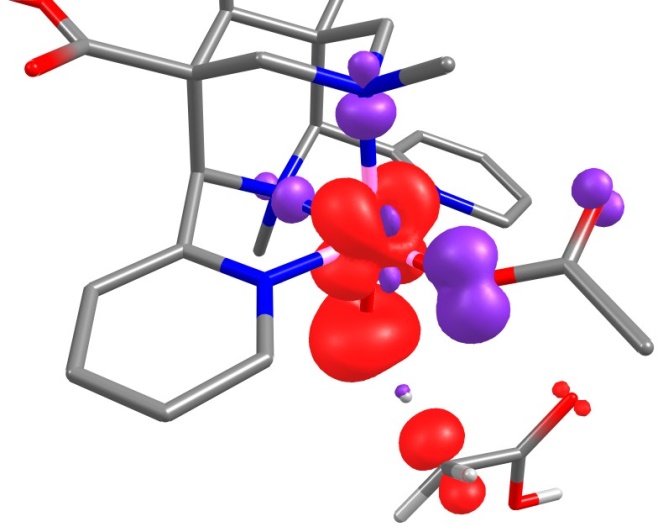


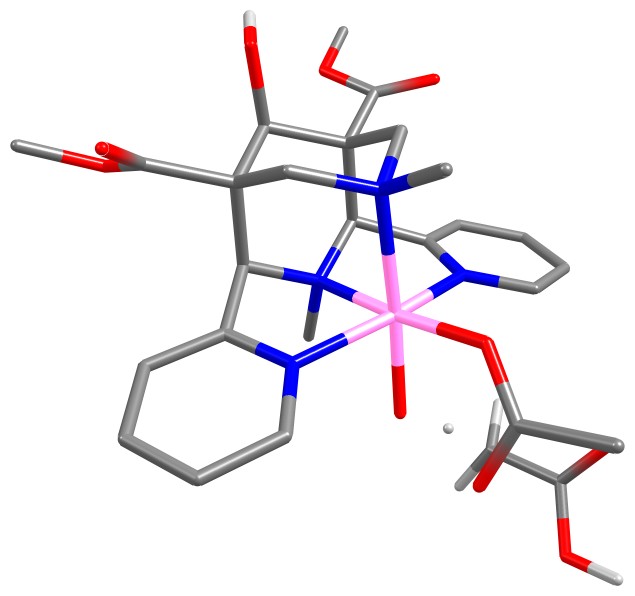

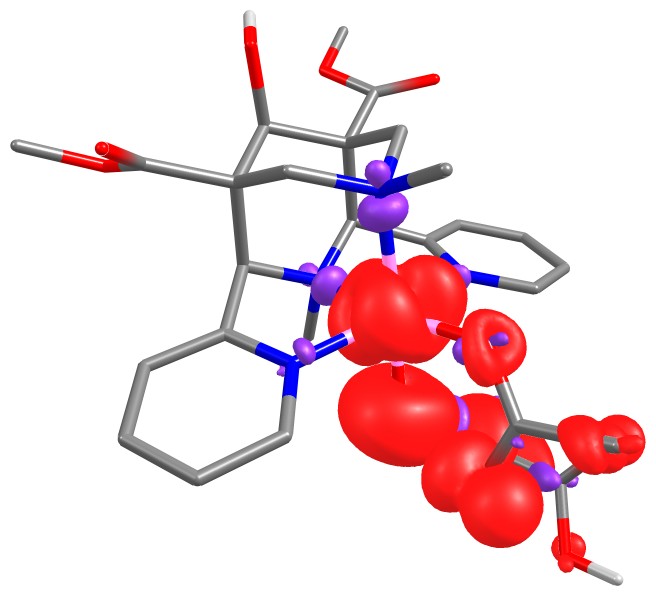


**Figure S24.** B3LYP-D3 optimized structure and spin density plot of the TS1 of [(L^2^)Fe^V^=O]^2+^ (O trans to N^7^). ^2^TS1_LS_ (top) and ^4^TS1_HS_ (bottom).


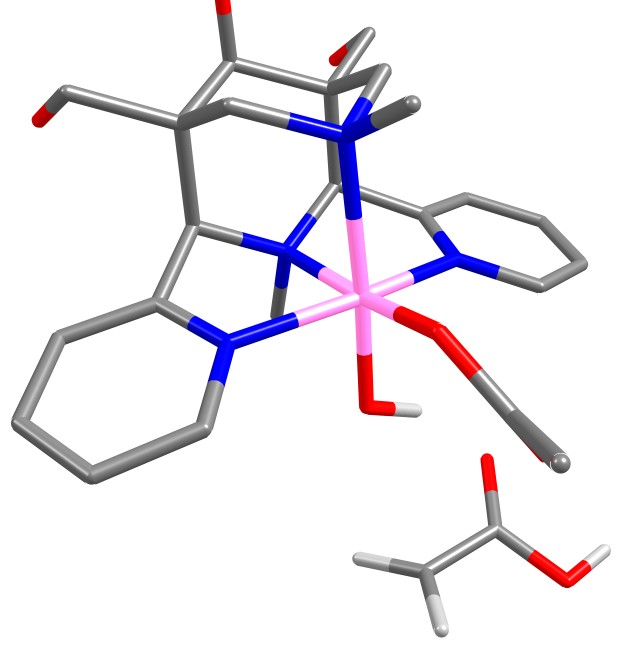

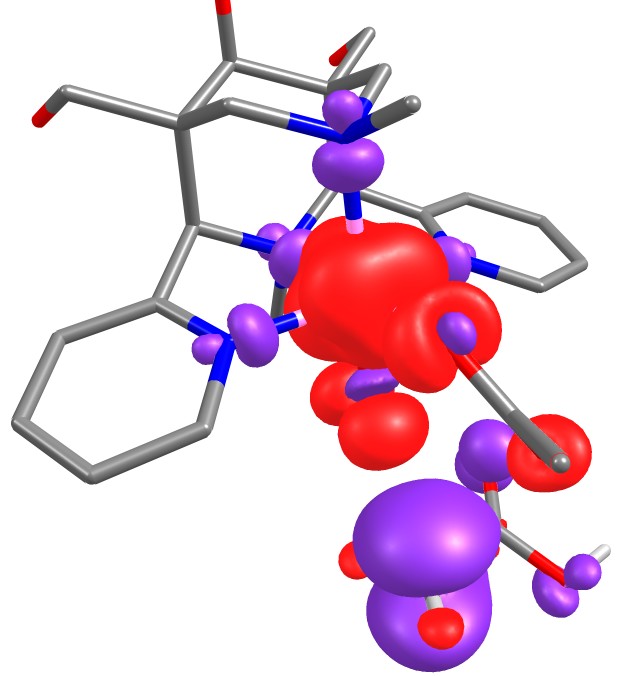


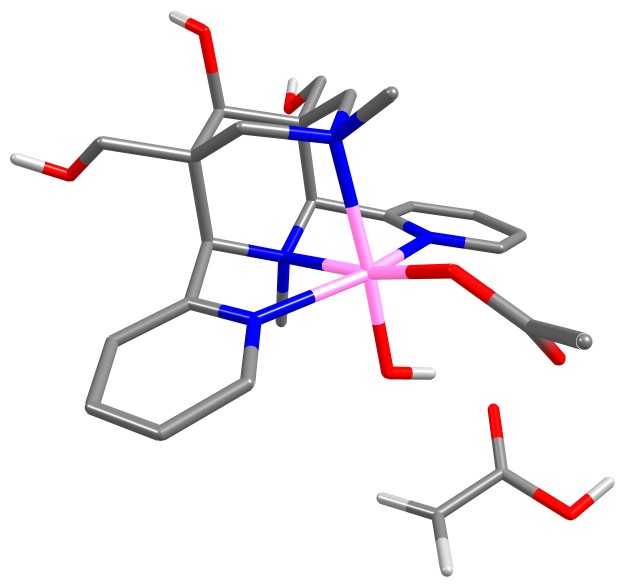

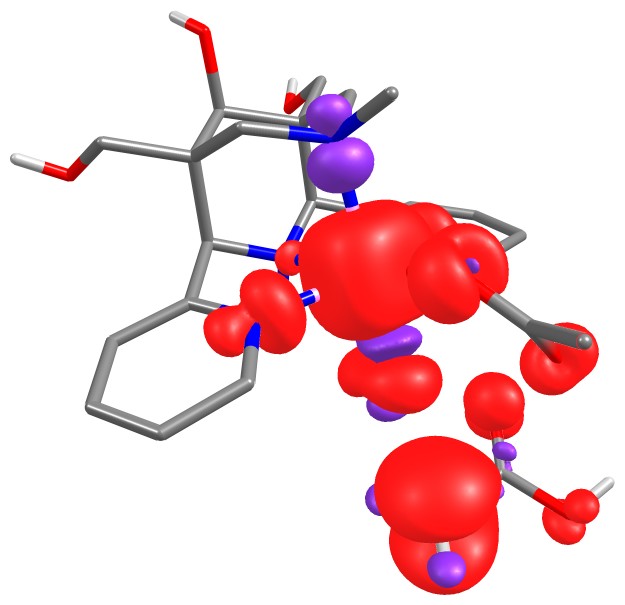


**Figure S25.** B3LYP-D3 optimized structure and spin density plot of the TS2 of [(L^1^)Fe^V^=O]^2+^ (O trans to N^7^). ^2^TS2_LS_ (top) and ^6^TS2_HS_ (bottom).


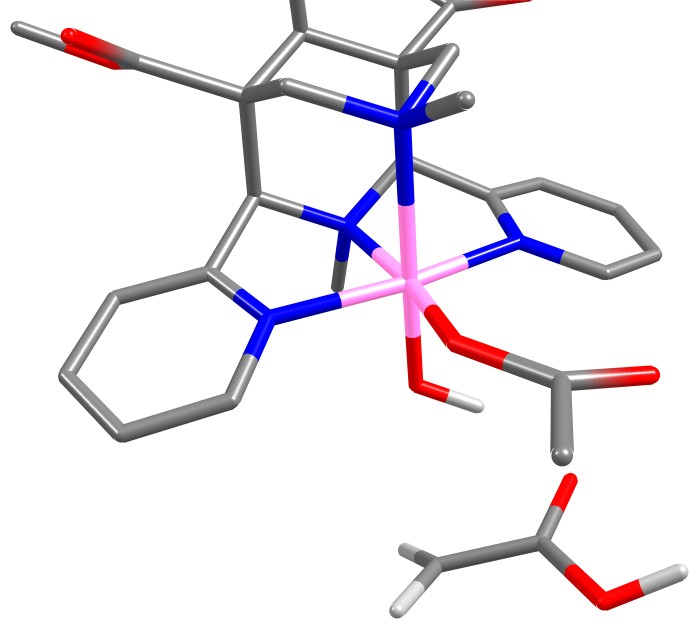

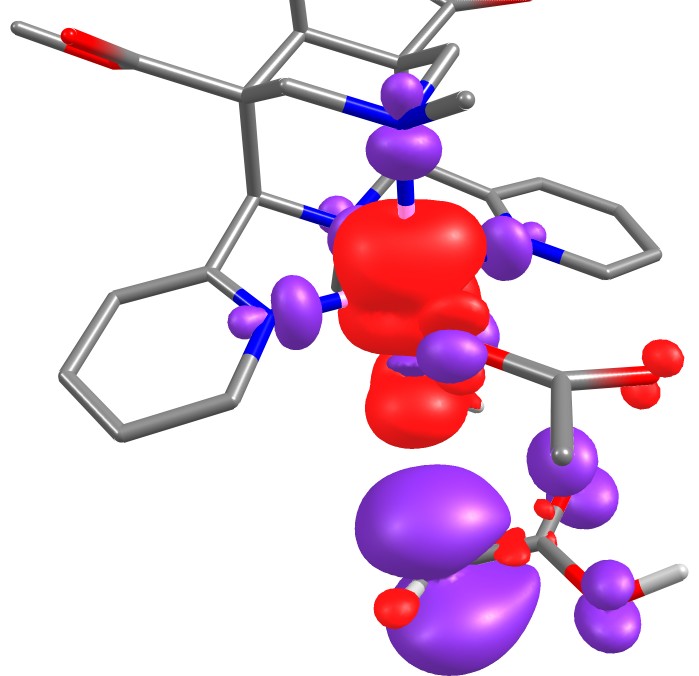


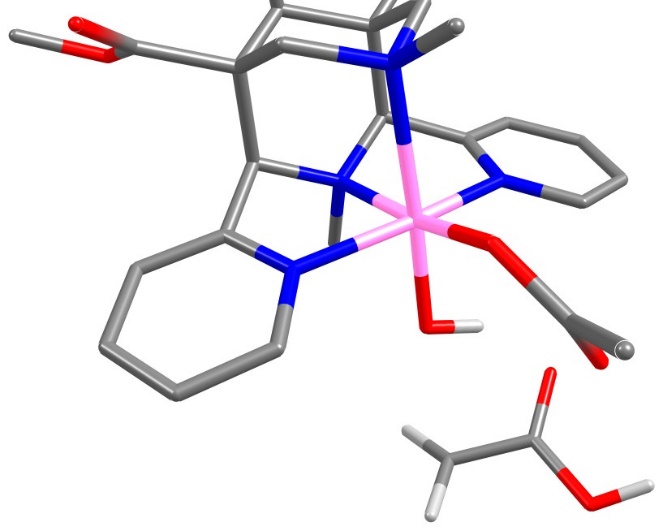

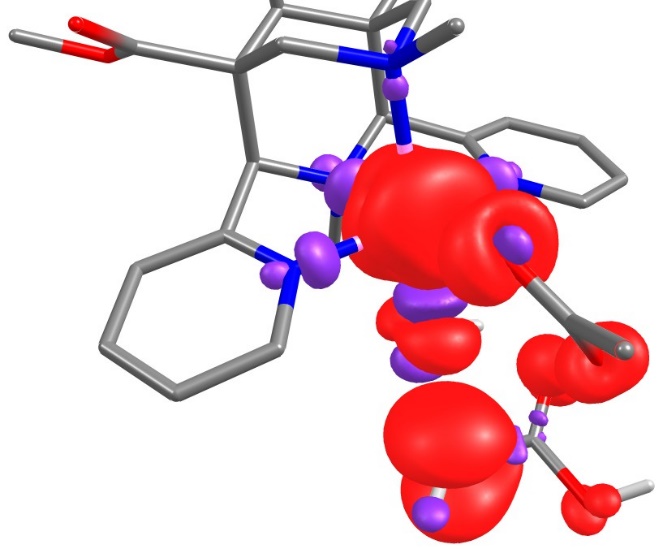


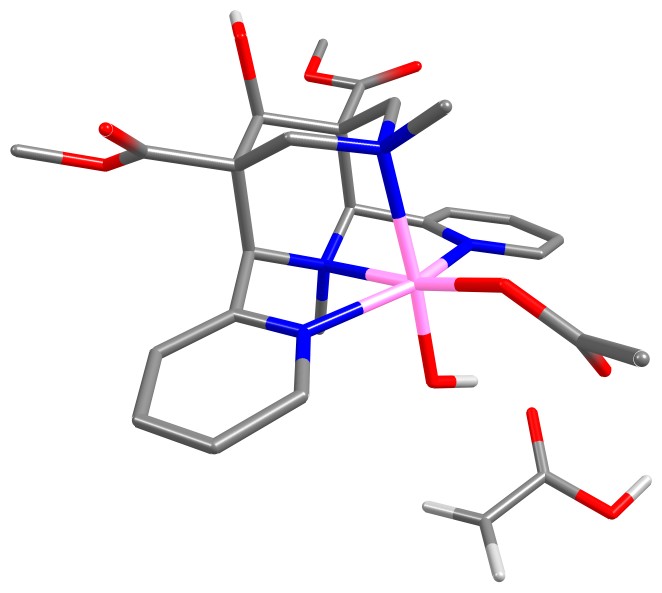

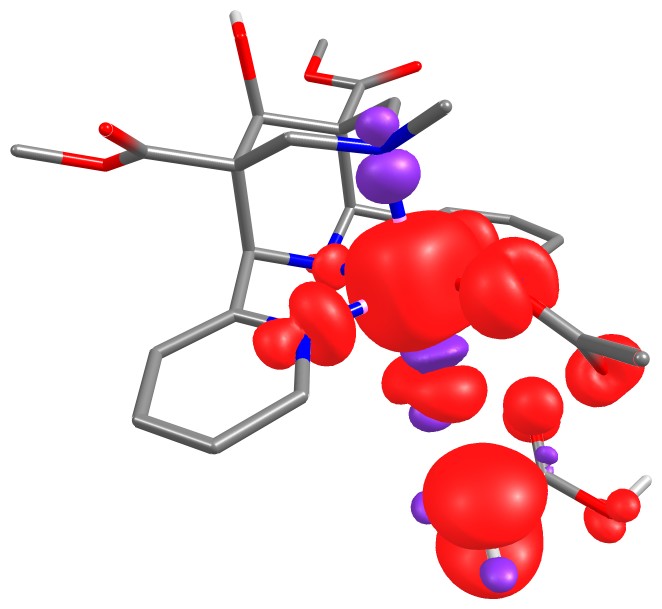


**Figure S26.** B3LYP-D3 optimized structure and spin density plot of the TS2 of [(L^2^)Fe^V^=O]^2+^ (O trans to N^7^). ^2^TS2_LS_ (top), ^4^TS2_IS_ (middle) and ^6^TS2_HS_ (bottom).

**Table S30.** Structural parameters of [(L^1,2^)Fe^IV^=O]^1+^ (R).

| Bond length (Å) | [(L^1^)Fe^IV^=O]^1+^ | | | [(L^2^)Fe^IV^=O]^1+^ | | |
| --- | --- | --- | --- | --- | --- | --- |
|  | ^5^R_Hs_ | ^3^R_IS_ | ^1^R_LS_ | ^5^R_Hs_ | ^3^R_IS_ | ^1^R_LS_ |
| Fe-N^7^ | 2.162 | 2.352 | 2.396 | 2.162 | 2.345 | 2.376 |
| Fe-N^Py1^ | 2.159 | 1.997 | 1.998 | 2.140 | 2.006 | 2.005 |
| Fe-N^Py2^ | 2.175 | 2.013 | 2.041 | 2.104 | 1.998 | 2.037 |
| Fe-N^3^ | 2.309 | 2.054 | 2.029 | 2.342 | 2.065 | 2.041 |
| Fe-O^1^ | 1.863 | 1.892 | 1.866 | 1.923 | 1.885 | 1.863 |
| Fe-O^2^ | 1.620 | 1.618 | 1.625 | 1.617 | 1.619 | 1.626 |

**Table S31.** Structural parameters of [(L^1,2^)Fe^IV^=O]^1+^ (R).

| bond angle (°) | [(L^1^)Fe^IV^=O]^1+^ | | | [(L^2^)Fe^IV^=O]^1+^ | | |
| --- | --- | --- | --- | --- | --- | --- |
|  | ^5^R_Hs_ | ^3^R_IS_ | ^1^R_LS_ | ^5^R_Hs_ | ^3^R_IS_ | ^1^R_LS_ |
| N^Py1^-Fe-N^Py2^ | 155.5 | 164.8 | 163.1 | 155.6 | 165.1 | 163.1 |
| N^Py1^-Fe-N^3^ | 89.7 | 82.5 | 82.1 | 87.6 | 82.6 | 82.0 |
| N^Py1^-Fe-N^7^ | 78.0 | 89.7 | 90.4 | 78.4 | 89.0 | 90.5 |
| N^Py1^-Fe-O^1^ | 90.2 | 88.9 | 87.4 | 83.9 | 105.3 | 86.9 |
| N^Py1^-Fe-O^2^ | 101.0 | 90.4 | 89.6 | 103.5 | 90.6 | 89.6 |
| O^1^-Fe-O^2^ | 96.0 | 96.6 | 98.5 | 95.1 | 96.8 | 98.5 |
| N^Py1^-Fe-O^1^-C^1^ | 78.9 | 130.0 | 176.4 | 169.9 | 55.0 | 175.7 |
| N^3^-Fe-N^7^ | 81.5 | 82.5 | 81.3 | 87.6 | 82.6 | 81.6 |

**Table S32.** Structural parameters of [(L^1,2^)Fe^IV^=O]^1+^ (TS1).

|  | [(L^1^)Fe^IV^=O]^1+^ | | [(L^2^)Fe^IV^=O]^1+^ | |
| --- | --- | --- | --- | --- |
| Bond length (Å) | ^5^TS1_Hs_ | ^3^TS1_IS_ | ^5^TS1_Hs_ | ^3^TS1_IS_ |
| Fe-N^7^ | 2.152 | 2.263 | 2.156 | 2.274 |
| Fe-N^Py1^ | 2.200 | 2.263 | 2.200 | 2.021 |
| Fe-N^Py2^ | 2.219 | 2.322 | 2.256 | 2.060 |
| Fe-N^3^ | 2.254 | 2.174 | 2.269 | 2.079 |
| Fe-O^1^ | 1.901 | 1.897 | 1.890 | 1.908 |
| Fe-O^2^ | 1.721 | 1.735 | 1.730 | 1.737 |
| O^2^-H^1^ | 1.172 | 1.240 | 1.159 | 1.177 |
| H^1^-C^2^ | 1.370 | 1.284 | 1.384 | 1.377 |

**Table S33.** Structural parameters of [(L^1,2^)Fe^IV^=O]^1+^ (TS1).

| bond angle (°) | [(L^1^)Fe^IV^=O]^1+^ | | [(L^2^)Fe^IV^=O]^1+^ | |
| --- | --- | --- | --- | --- |
|  | ^5^TS1_Hs_ | ^3^TS1_IS_ | ^5^TS1_Hs_ | ^3^TS1_IS_ |
| N^Py1^-Fe-N^Py2^ | 154.7 | 153.0 | 154.7 | 163.5 |
| N^Py1^-Fe-N^3^ | 90.7 | 76.8 | 90.5 | 81.3 |
| N^Py1^-Fe-N^7^ | 77.4 | 90.1 | 77.6 | 91.6 |
| N^Py1^-Fe-O^1^ | 91.8 | 88.1 | 86.6 | 86.9 |
| N^Py1^-Fe-O^2^ | 100.9 | 92.2 | 91.1 | 85.8 |
| O^1^-Fe-O^2^ | 96.6 | 94.8 | 95.7 | 93.8 |
| Fe-O^2^-H^1^ | 119.0 | 120.8 | 125.4 | 121.6 |
| N^Py1^-Fe-O^1^-C^1^ | 55.6 | 173.5 | 139.5 | 177.4 |
| N^Py1^-Fe-O^2^-H^1^ | 108.0 | 105.6 | 158.7 | 139.7 |
| N^3^-Fe-N^7^ | 77.4 | 82.3 | 82.4 | 84.0 |
| O^2^-H^1^-C^2^ | 167.8 | 166.7 | 167.6 | 161.7 |

**Table S34.** Structural parameters of [(L^1,2^)Fe^IV^=O]^1+^ (INT).

|  | [(L^1^)Fe^IV^=O]^1+^ | | | [(L^2^)Fe^IV^=O]^1+^ | | |
| --- | --- | --- | --- | --- | --- | --- |
| Bond length (Å) | ^5^INT_Hs_ | ^3^INT_ls_ | ^1^INT_LS_ | ^5^INT_Hs_ | ^3^INT_ls_ | ^1^INT_LS_ |
| Fe-N^3^ | 2.506 | 2.213 | 2.266 | 2.482 | 2.205 | 2.259 |
| Fe-N^Py1^ | 2.157 | 2.208 | 1.980 | 2.156 | 2.203 | 1.981 |
| Fe-N^Py2^ | 2.182 | 2.231 | 2.022 | 2.177 | 2.216 | 2.017 |
| Fe-N^7^ | 2.247 | 2.183 | 2.037 | 2.272 | 2.210 | 2.046 |
| Fe-O^1^ | 1.929 | 1.904 | 1.918 | 1.924 | 1.904 | 1.913 |
| Fe-O^2^ | 1.822 | 1.780 | 1.777 | 1.823 | 1.779 | 1.776 |
| O^2^-H^1^ | 0.984 | 0.983 | 0.985 | 0.985 | 0.984 | 0.986 |

**Table S35.** Structural parameters of [(L^1,2^)Fe^IV^=O]^1+^ (INT).

|  | [(L^1^)Fe^IV^=O]^1+^ | | | [(L^2^)Fe^IV^=O]^1+^ | | |
| --- | --- | --- | --- | --- | --- | --- |
| Bond angle (°) | ^5^INT_Hs_ | ^3^INT_ls_ | ^1^INT_LS_ | ^5^INT_Hs_ | ^3^INT_ls_ | ^1^INT_LS_ |
| N^Py1^-Fe-N^Py2^ | 151.5 | 152.2 | 165.6 | 151.3 | 151.8 | 166.0 |
| N^Py1^-Fe-N^3^ | 75.9 | 76.7 | 82.6 | 75.8 | 76.4 | 82.8 |
| N^Py1^-Fe-N^7^ | 86.7 | 92.2 | 91.1 | 86.4 | 92.1 | 90.9 |
| N^Py1^-Fe-O^1^ | 101.5 | 96.9 | 93.3 | 100.9 | 96.3 | 93.1 |
| N^Py1^-Fe-O^2^ | 93.1 | 87.3 | 87.1 | 93.3 | 87.4 | 87.4 |
| O^1^-Fe-O^2^ | 99.0 | 93.9 | 93.9 | 99.2 | 93.9 | 94.1 |
| Fe-O^2^-H^1^ | 108.7 | 106.0 | 105.3 | 108.5 | 106.1 | 105.3 |
| N^Py1^-Fe-O^1^-C^1^ | 120.5 | 122.9 | 117.5 | 120.8 | 123.7 | 117.3 |
| N^Py1^-Fe-O^2^-H^1^ | 128.1 | 135.1 | 131.0 | 127.1 | 135.0 | 130.1 |
| N^3^-Fe-N^7^ | 76.7 | 83.3 | 84.8 | 76.9 | 83.3 | 85.0 |

**Table S36.** Structural parameters of [(L^1,2^)Fe^IV^=O]^1+^ (TS2).

|  | [(L^1^)Fe^IV^=O]^1+^ | | | [(L^2^)Fe^IV^=O]^1+^ | | |
| --- | --- | --- | --- | --- | --- | --- |
| Bond length (Å) | ^7^TS2 | ^5^TS2_HS_ | ^3^TS2_IS_ | ^7^TS2 | ^5^TS2_HS_ | ^3^TS2_IS_ |
| Fe-N^7^ | 2.329 | 2.238 | 2.307 | 2.322 | 2.232 | 2.299 |
| Fe-N^Py1^ | 2.138 | 2.195 | 2.006 | 2.118 | 2.194 | 2.006 |
| Fe-N^Py2^ | 2.090 | 2.175 | 2.027 | 2.102 | 2.162 | 2.025 |
| Fe-N^3^ | 2.284 | 2.253 | 2.046 | 2.305 | 2.282 | 2.057 |
| Fe-O^1^ | 1.890 | 1.946 | 1.924 | 1.876 | 1.943 | 1.919 |
| Fe-O^2^ | 2.045 | 1.847 | 1.879 | 2.045 | 1.848 | 1.879 |
| O^2^-H^1^ | 0.977 | 0.975 | 0.974 | 0.977 | 0.975 | 0.975 |
| O^2^-C^2^ | 1.758 | 2.410 | 2.139 | 1.766 | 2.409 | 2.142 |

**Table S37.** Structural parameters of [(L^1,2^)Fe^IV^=O]^1+^ (TS2).

|  | [(L^1^)Fe^IV^=O]^1+^ | | | [(L^2^)Fe^IV^=O]^1+^ | | |
| --- | --- | --- | --- | --- | --- | --- |
| Bond angle (°) | ^7^TS2 | ^5^TS2_HS_ | ^3^TS2_IS_ | ^7^TS2 | ^5^TS2_HS_ | ^3^TS2_IS_ |
| N^Py1^-Fe-N^Py2^ | 104.6 | 149.0 | 163.0 | 150.2 | 148.6 | 163.2 |
| N^Py1^-Fe-N^3^ | 74.6 | 74.6 | 82.2 | 74.8 | 74.3 | 82.3 |
| N^Py1^-Fe-N^7^ | 91.7 | 93.2 | 93.2 | 91.5 | 92.9 | 92.6 |
| N^Py1^-Fe-O^1^ | 90.7 | 88.4 | 86.8 | 92.8 | 88.6 | 86.8 |
| N^Py1^-Fe-O^2^ | 89.5 | 86.3 | 87.0 | 89.8 | 86.3 | 87.3 |
| O^1^-Fe-O^2^ | 94.6 | 93.1 | 92.1 | 94.9 | 93.4 | 92.3 |
| Fe-O^2^-H^1^ | 109.2 | 106.8 | 106.4 | 109.1 | 106.8 | 106.4 |
| N^Py1^-Fe-O^1^-C^1^ | 176.7 | 149.9 | 150.3 | 175.1 | 149.4 | 149.2 |
| N^Py1^-Fe-O^2^-H^1^ | 150.9 | 164.2 | 166.7 | 152.2 | 165.2 | 168.4 |
| N^3^-Fe-N^7^ | 79.8 | 81.6 | 64.1 | 79.8 | 81.5 | 84.2 |
| Fe-O^2^-C^2^ | 126.4 | 156.8 | 153.7 | 125.4 | 156.6 | 153.9 |

**Table S38.** Structural parameters of [(L^1,2^)Fe^IV^=O]^1+^ (P).

|  | [(L^1^)Fe^IV^=O]^1+^ | | | [(L^2^)Fe^IV^=O]^1+^ | | |
| --- | --- | --- | --- | --- | --- | --- |
| Bond length (Å) | ^5^P_HS_ | ^3^P_lS_ | ^1^P_LS_ | ^5^P_HS_ | ^3^P_lS_ | ^1^P_LS_ |
| Fe-N^7^ | 2.259 | 2.327 | 2.107 | 2.249 | 2.312 | 2.104 |
| Fe-N^Py1^ | 2.195 | 1.981 | 1.986 | 2.192 | 1.976 | 1.985 |
| Fe-N^Py2^ | 2.200 | 1.978 | 1.987 | 2.192 | 1.977 | 1.984 |
| Fe-N^3^ | 2.209 | 2.040 | 1.997 | 2.228 | 2.057 | 2.002 |
| Fe-O^1^ | 2.019 | 1.996 | 2.043 | 2.009 | 1.997 | 2.038 |
| Fe-O^3^ | 2.292 | 2.373 | 2.035 | 2.301 | 2.377 | 2.034 |

**Table S39.**Structural parameters of [(L^1,2^)Fe^IV^=O]^1+^ (P).

|  | [(L^1^)Fe^IV^=O]^1+^ | | | [(L^2^)Fe^IV^=O]^1+^ | | |
| --- | --- | --- | --- | --- | --- | --- |
| bond angle (°) | ^5^P_HS_ | ^3^P_lS_ | ^1^P_LS_ | ^5^P_HS_ | ^3^P_lS_ | ^1^P_LS_ |
| N^Py1^-Fe-N^Py2^ | 151.0 | 165.5 | 165.4 | 151.3 | 165.7 | 165.9 |
| N^Py1^-Fe-N^3^ | 76.3 | 83.3 | 83.6 | 76.3 | 83.4 | 83.8 |
| N^Py1^-Fe-N^7^ | 92.6 | 91.6 | 93.2 | 92.1 | 92.0 | 93.0 |
| N^Py1^-Fe-O^1^ | 103.5 | 95.4 | 95.5 | 101.7 | 95.8 | 95.3 |
| O^1^-Fe-O^3^ | 61.3 | 60.1 | 64.7 | 61.3 | 60.0 | 64.8 |
| N^Py1^-Fe-O^1^-C^1^ | 81.5 | 86.4 | 85.2 | 82.4 | 85.1 | 85.7 |
| N^Py1^-Fe-O^3^ | 89.3 | 89.6 | 88.2 | 89.1 | 88.5 | 88.3 |
| N^3^-Fe-N^7^ | 82.0 | 83.8 | 88.2 | 82.0 | 83.9 | 88.4 |

**Table S40.** Structural parameters of [(L^1,2^)Fe^V^=O]^2+^ (R).

|  | [(L^1^)Fe^V^=O]^2+^ | | [(L^2^)Fe^V^=O]^2+^ | |
| --- | --- | --- | --- | --- |
| Bond length (Å) | ^4^R_HS_ | ^2^R_IS_ | ^4^R_HS_ | ^2^R_IS_ |
| Fe-N^7^ | 2.253 | 2.259 | 2.229 | 2.245 |
| Fe-N^Py1^ | 1.991 | 1.991 | 1.990 | 1.990 |
| Fe-N^Py2^ | 1.990 | 1.999 | 1.986 | 1.993 |
| Fe-N^3^ | 2.047 | 1.972 | 2.057 | 1.981 |
| Fe-O^1^ | 1.808 | 2.081 | 1.810 | 2.073 |
| Fe-O^2^ | 1.671 | 1.621 | 1.682 | 1.623 |

**Table S41.** Structural parameters of [(L^1,2^)Fe^V^=O]^2+^ (R).

|  | [(L^1^)Fe^V^=O]^2+^ | | [(L^2^)Fe^V^=O]^2+^ | |
| --- | --- | --- | --- | --- |
| Bond angle (°) | ^4^R_HS_ | ^2^R_IS_ | ^4^R_HS_ | ^2^R_IS_ |
| N^Py1-^Fe-N^Py2^ | 161.0 | 168.0 | 161.4 | 168.3 |
| N^Py1^-Fe-N^3^ | 81.1 | 84.0 | 81.2 | 84.2 |
| N^Py1-^Fe-N^7^ | 92.1 | 90.0 | 92.5 | 89.6 |
| N^Py1-^Fe-O^1^ | 97.2 | 92.4 | 101.1 | 92.5 |
| N^Py1-^Fe-O^2^ | 87.4 | 90.6 | 87.7 | 90.9 |
| O^1^-Fe-O2 | 93.5 | 88.8 | 92.9 | 88.9 |
| N^Py1^-Fe-O^1^-C^1^ | 99.5 | 112.6 | 77.1 | 111.0 |
| N^3^-Fe-N^7^ | 84.4 | 85.4 | 84.9 | 85.7 |

**Table S42.** Structural parameters of [(L^1,2^)Fe^V^=O]^2+^ (TS1).

|  | [(L^1^)Fe^V^=O]^2+^ | | [(L^2^)Fe^V^=O]^2+^ | |
| --- | --- | --- | --- | --- |
| Bond length (Å) | ^4^TS1_HS_ | ^2^TS1_IS_ | ^4^TS1_HS_ | ^2^TS1_IS_ |
| Fe-N^7^ | 2.296 | 2.285 | 2.159 | 2.270 |
| Fe-N^Py1^ | 2.010 | 1.996 | 2.005 | 1.994 |
| Fe-N^Py2^ | 2.005 | 2.008 | 1.983 | 2.008 |
| Fe-N^3^ | 2.062 | 2.046 | 2.034 | 2.059 |
| Fe-O^1^ | 1.838 | 1.851 | 1.877 | 1.846 |
| Fe-O^2^ | 1.737 | 1.695 | 1.864 | 1.696 |
| O^2^-H^1^ | 1.254 | 1.302 | 1.224 | 1.313 |
| H^1^-C^2^ | 1.295 | 1.244 | 1.322 | 1.236 |

**Table S43.** Structural parameters of [(L^1,2^)Fe^V^=O]^2+^ (TS1).

|  | [(L^1^)Fe^V^=O]^2+^ | | [(L^2^)Fe^V^=O]^2+^ | |
| --- | --- | --- | --- | --- |
| Bond angle (°) | ^4^TS1_HS_ | ^2^TS1_LS_ | ^4^TS1_HS_ | ^2^TS1_LS_ |
| N^Py1^-Fe-N^Py2^ | 108.8 | 165.1 | 162.5 | 165.1 |
| N^Py1^-Fe-N^3^ | 81.9 | 83.2 | 81.8 | 83.1 |
| N^Py1^-Fe-N^7^ | 87.7 | 86.9 | 93.8 | 86.9 |
| N^Py1^-Fe-O^1^ | 92.2 | 89.5 | 102.0 | 89.4 |
| N^Py1^-Fe-O^2^ | 88.5 | 89.7 | 80.9 | 89.8 |
| O^1^-Fe-O^2^ | 90.5 | 94.6 | 89.0 | 94.6 |
| Fe-O^2^-H^1^ | 119.3 | 115.0 | 122.3 | 115.0 |
| N^Py1^-Fe-O^1^-C^1^ | 155.6 | 164.2 | 69.5 | 163.9 |
| N^Py1^-Fe-O^2^-H^1^ | 83.2 | 82.7 | 179.1 | 82.6 |
| N^3^-Fe-N^7^ | 83.9 | 83.6 | 86.3 | 83.8 |
| O^2^-H^1^-C^2^ | 170.9 | 172.8 | 173.1 | 172.9 |

**Table S44.** Structural parameters of [(L^1,2^)Fe^V^=O]^2+^ (INT).

|  | [(L^1^)Fe^V^=O]^2+^ | | | [(L^2^)Fe^V^=O]^2+^ | | |
| --- | --- | --- | --- | --- | --- | --- |
| Bond length (Å) | ^6^­INT_HS_ | ^4^­INT_IS_ | ^2^INT_LS_ | ^6^­INT_HS_ | ^4^­INT_IS_ | ^2^INT_LS_ |
| Fe-N^7^ | 2.304 | 2.159 | 2.214 | 2.165 | 2.146 | 2.190 |
| Fe-N^Py1^ | 2.116 | 1.983 | 1.986 | 2.203 | 1.982 | 1.987 |
| Fe-N^Py2^ | 2.144 | 1.993 | 2.004 | 2.241 | 1.987 | 1.998 |
| Fe-N^3^ | 2.189 | 2.015 | 2.003 | 2.136 | 2.026 | 2.015 |
| Fe-O^1^ | 1.997 | 1.900 | 1.870 | 1.889 | 1.898 | 1.872 |
| Fe-O^2^ | 1.935 | 1.886 | 1.729 | 1.876 | 1.887 | 1.736 |
| O^2^-H^1^ | 0.974 | 0.975 | 0.980 | 0.975 | 0.975 | 0.980 |

**Table S45.** Structural parameters of [(L^1,2^)Fe^V^=O]^2+^ (INT).

|  | [(L^1^)Fe^V^=O]^2+^ | | | [(L^2^)Fe^V^=O]^2+^ | | |
| --- | --- | --- | --- | --- | --- | --- |
|  | ^6^­INT_HS_ | ^4^­INT_IS_ | ^2^INT_LS_ | ^6^­INT_HS_ | ^4^­INT_IS_ | ^2^INT_LS_ |
| N^Py1^-Fe-N^Py2^ | 151.8 | 161.4 | 165.9 | 152.2 | 162.0 | 166.2 |
| N^Py1^-Fe-N^3^ | 76.7 | 82.2 | 83.7 | 77.3 | 82.4 | 83.8 |
| N^Py1^-Fe-N^7^ | 93.5 | 94.2 | 91.2 | 94.2 | 94.1 | 91.1 |
| N^Py1^-Fe-O^1^ | 100.2 | 96.6 | 96.0 | 100.9 | 96.5 | 95.9 |
| N^Py1^-Fe-O^2^ | 88.7 | 83.1 | 86.0 | 85.8 | 83.3 | 86.1 |
| O^1^-Fe-O^2^ | 85.9 | 87.5 | 91.1 | 88.4 | 87.7 | 90.6 |
| Fe-O^2^-H^1^ | 127.7 | 115.1 | 116.0 | 117.9 | 115.1 | 116.1 |
| N^Py1^-Fe-O^1-^C^1^ | 103.1 | 94.1 | 96.7 | 100.7 | 92.7 | 94.7 |
| N^Py1^-Fe-O^2^-H^1^ | 151.0 | 165.8 | 171.8 | 157.8 | 167.8 | 171.4 |
| N^3^-Fe-N^7^ | 81.6 | 86.5 | 84.9 | 85.4 | 86.8 | 85.5 |

**Table S46.** Structural parameters of [(L^1,2^)Fe^V^=O]^2+^ (TS2).

|  | [(L^1^)Fe^V^=O]^2+^ | | | [(L^2^)Fe^V^=O]^2+^ | | |
| --- | --- | --- | --- | --- | --- | --- |
| Bond length (Å) | ^6^INT_HS_ | ^4^­INT_IS_ | ^2^INT_LS_ | ^6^INT_HS_ | ^4^­INT_IS_ | ^2^INT_LS_ |
| Fe-N^7^ | 2.280 | 2.313 | 2.229 | 2.268 | 2.299 | 2.207 |
| Fe-N^Py1^ | 2.152 | 1.992 | 1.993 | 2.144 | 1.991 | 2.009 |
| Fe-N^Py2^ | 2.171 | 1.994 | 1.993 | 2.158 | 1.988 | 2.011 |
| Fe-N^3^ | 2.189 | 2.048 | 2.047 | 2.216 | 2.060 | 2.055 |
| Fe-O^1^ | 1.841 | 1.801 | 1.829 | 1.842 | 1.801 | 1.881 |
| Fe-O^2^ | 1.782 | 1.821 | 1.789 | 1.782 | 1.821 | 1.751 |
| O^2^-H^1^ | 0.998 | 0.984 | 0.994 | 0.999 | 0.985 | 1.001 |
| O^2^-C^2^ | 2.679 | 2.454 | 2.592 | 2.685 | 2.446 | 2.597 |

**Table S47.** Structural parameters of [(L^1,2^)Fe^V^=O]^2+^ (TS2).

|  | [(L^1^)Fe^V^=O]^2+^ | | | [(L^2^)Fe^V^=O]^2+^ | | |
| --- | --- | --- | --- | --- | --- | --- |
| Bond angle (°) | ^6^INT_HS_ | ^4^­INT_IS_ | ^2^INT_LS_ | ^6^INT_HS_ | ^4^­INT_IS_ | ^2^INT_LS_ |
| N^Py1^-Fe-N^Py2^ | 153.1 | 160.9 | 162.3 | 152.9 | 161.5 | 164.1 |
| N^Py1^-Fe-N^3^ | 77.0 | 81.2 | 82.0 | 76.8 | 81.5 | 82.1 |
| N^Py1^-Fe-N^7^ | 90.5 | 93.0 | 92.8 | 90.2 | 92.8 | 92.9 |
| N^Py1^-Fe-O^1^ | 101.9 | 95.8 | 93.8 | 101.5 | 95.4 | 85.6 |
| N^Py1^-Fe-O^2^ | 86.8 | 84.1 | 84.5 | 87.2 | 84.2 | 88.7 |
| O^1^-Fe-O^2^ | 94.8 | 95.1 | 96.2 | 94.8 | 95.2 | 94.2 |
| Fe-O^2-^H^1^ | 111.9 | 108.0 | 113.7 | 112.1 | 107.9 | 114.8 |
| N^Py1^-Fe-O^1^-C^1^ | 109.1 | 105.8 | 108.2 | 109.8 | 106.0 | 166.9 |
| N^Py1^-Fe-O^2^-H^1^ | 172.5 | 178.6 | 173.8 | 172.0 | 179.2 | 157.6 |
| N^3-^Fe-N^7^ | 81.8 | 83.6 | 84.9 | 81.9 | 83.9 | 85.3 |
| Fe-O^2^-C^2^ | 169.0 | 173.4 | 148.3 | 167.1 | 173.0 | 129.7 |

**Table S48.** Structural parameters of [(L^1,2^)Fe^V^=O]^2+^ (P).

|  | [(L^1^)Fe^V^=O]^2+^ | | | [(L^2^)Fe^V^=O]^2+^ | | |
| --- | --- | --- | --- | --- | --- | --- |
| Bond length (Å) | ^6^P_HS_ | ^4^P_lS_ | ^2^P_LS_ | ^6^P_HS_ | ^4^P_lS_ | ^2^P_LS_ |
| Fe-N^7^ | 2.174 | 2.245 | 2.084 | 2.164 | 2.321 | 2.075 |
| Fe-N^Py1^ | 2.125 | 1.993 | 1.985 | 2.121 | 1.989 | 1.983 |
| Fe-N^Py2^ | 2.125 | 1.993 | 1.985 | 2.116 | 1.985 | 1.979 |
| Fe-N^3^ | 2.161 | 1.983 | 1.981 | 2.182 | 2.023 | 1.992 |
| Fe-O^1^ | 1.945 | 1.939 | 1.960 | 1.940 | 1.940 | 1.955 |
| Fe-O^3^ | 2.265 | 2.232 | 1.966 | 2.267 | 2.381 | 1.966 |

**Table S49.** Structural parameters of [(L^1,2^)Fe^V^=O]^2+^ (P).

|  | [(L^1^)Fe^V^=O]^2+^ | | | [(L^2^)Fe^V^=O]^2+^ | | |
| --- | --- | --- | --- | --- | --- | --- |
| Bond angle (°) | ^6^P_HS_ | ^4^P_lS_ | ^2^P_LS_ | ^6^P_HS_ | ^4^P_lS_ | ^2^P_LS_ |
| N^Py1^-Fe-N^Py2^ | 153.6 | 165.3 | 164.1 | 153.7 | 166.1 | 164.8 |
| N^Py1^-Fe-N^3^ | 78.0 | 83.6 | 83.2 | 77.9 | 83.6 | 83.4 |
| N^Py1^-Fe-N^7^ | 78.0 | 93.1 | 93.9 | 94.0 | 91.9 | 93.6 |
| N^Py1^-Fe-O^1^ | 99.1 | 95.5 | 95.9 | 98.9 | 95.2 | 95.6 |
| O^1^-Fe-O^3^ | 62.0 | 62.7 | 66.4 | 62.0 | 60.0 | 66.5 |
| N^Py1^-Fe-O^1^-C^1^ | 80.4 | 85.2 | 84.7 | 80.7 | 85.7 | 85.0 |
| N^Py1^-Fe-O^3^ | 85.9 | 88.3 | 87.6 | 86.2 | 89.0 | 87.7 |
| N^3^-Fe-N^7^ | 85.2 | 86.3 | 87.9 | 85.2 | 84.7 | 88.2 |

**Table S50.** Computed spin density values of [(L^1,2^)Fe^IV^=O]^1+^ (R and INT).

|  | | Fe | N^Py1^ | N^Py2^ | N^3^ | N^7^ | O^1^ | O^2^ | O^3^ | C^1^ | H^1^ |
| --- | --- | --- | --- | --- | --- | --- | --- | --- | --- | --- | --- |
| [(L^1^)Fe^IV^=O]^1+^ | ^5^­R_HS_ | 1.301 | -0.018 | -0.016 | -0.018 | -0.023 | -0.019 | 0.790 | 0.006 | 0.002 | - |
|  | ^3^R_IS_ | 0.000 | 0.000 | 0.000 | 0.000 | 0.000 | 0.000 | 0.000 | 0.000 | 0.000 | - |
| [(L^2^)Fe^IV^=O]^1+^ | ^1^R_LS_ | 1.286 | -0.015 | -0.017 | -0.016 | -0.023 | -0.021 | 0.801 | 0.007 | 0.002 | - |
|  | ^5^­R_HS_ | 0.000 | 0.000 | 0.000 | 0.000 | 0.000 | 0.000 | 0.000 | 0.000 | 0.000 | - |
| [(L^1^)Fe^IV^=O]^1+^ | ^3^R_IS_ | 4.193 | 0.060 | 0.063 | 0.066 | 0.028 | 0.126 | 0.352 | 0.019 | 0.003 | 0.005 |
|  | ^1^R_LS_ | 2.870 | 0.047 | 0.041 | 0.000 | -0.048 | 0.009 | 0.026 | 0.010 | 0.010 | -0.003 |
|  | ^5^­INT_HS_ | 0.978 | -0.020 | -0.024 | -0.022 | -0.020 | -0.012 | 0.110 | 0.003 | 0.002 | -0.003 |
| [(L^2^)Fe^IV^=O]^1+^ | ^3^INT_IS_ | 4.191 | 0.060 | 0.063 | 0.057 | 0.027 | 0.131 | 0.359 | 0.020 | 0.002 | 0.005 |
|  | ^1^INT_LS_ | 2.869 | 0.046 | 0.039 | 0.003 | -0.048 | 0.013 | 0.020 | 0.010 | 0.010 | -0.003 |
|  | ^5^­INT_HS_ | 0.971 | 0.040 | -0.024 | -0.022 | 0.020 | -0.013 | 0.115 | 0.003 | 0.002 | -0.004 |

**Table S51.** Computed spin density values of [(L^1,2^)Fe^IV^=O]^1+^ (P).

|  | | Fe | N^Py1^ | N^Py2^ | N^3^ | N^7^ | O^1^ | O^3^ | C^1^ |
| --- | --- | --- | --- | --- | --- | --- | --- | --- | --- |
| [(L^1^)Fe^IV^=O]^1+^ | ^5^P_HS_ | 3.776 | 0.021 | 0.022 | 0.038 | 0.023 | 0.048 | 0.028 | 0.008 |
|  | ^3^P_lS_ | 2.023 | -0.034 | -0.034 | 0.000 | 0.030 | 0.002 | 0.027 | 0.005 |
|  | ^1^P_LS_ | 0.000 | 0.000 | 0.000 | 0.000 | 0.000 | 0.000 | 0.000 | 0.000 |
| [(L^2^)Fe^IV^=O]^1+^ | ^5^P_HS_ | 3.774 | 0.022 | 0.021 | 0.033 | 0.022 | 0.052 | 0.029 | 0.008 |
|  | ^3^P_lS_ | 2.018 | -0.034 | -0.034 | 0.003 | 0.029 | 0.003 | 0.027 | 0.005 |
|  | ^1^P_LS_ | 0.000 | 0.000 | 0.000 | 0.000 | 0.000 | 0.000 | 0.000 | 0.000 |

**Table S52.** Computed spin density values of [(L^1,2^)Fe^IV^=O]^1+^ (TS1 and TS2).

|  | | Fe | N^Py1^ | N^Py2^ | N^3^ | N^7^ | O^1^ | O^2^ | O^3^ | C^1^ | C^2^ | H^1^ | C^3^ | O^4^ |
| --- | --- | --- | --- | --- | --- | --- | --- | --- | --- | --- | --- | --- | --- | --- |
| [(L^1^)Fe^IV^=O]^1+^ | ^5^­TS1_HS_ | 3.001 | 0.049 | 0.062 | -0.040 | -0.026 | 0.007 | 0.533 | 0.012 | 0.002 | 0.291 | -0.035 | -0.008 | 0.056 |
|  | ^3^TS1_IS_ | 1.224 | -0.005 | -0.049 | -0.032 | -0.025 | -0.027 | 0.613 | 0.002 | 0.001 | 0.307 | -0.037 | -0.009 | 0.038 |
| [(L^2^)Fe^IV^=O]^1+^ | ^5^­TS1_HS_ | 3.391 | 0.053 | 0.051 | -0.015 | -0.018 | 0.021 | 0.432 | 0.011 | 0.001 | 0.071 | -0.008 | 0.003 | 0.009 |
|  | ^3^TS1_IS_ | 1.149 | -0.025 | -0.019 | -0.026 | -0.023 | -0.008 | 0.530 | 0.006 | -0.002 | 0.416 | -0.045 | -0.013 | 0.068 |
| [(L^1^)Fe^IV^=O]^1+^ | ^7^TS2 | 4.275 | 0.075 | 0.126 | 0.077 | 0.073 | 0.157 | 0.194 | 0.023 | 0.006 | 0.364 | 0.002 | 0.191 | 0.104 |
|  | ^5^­TS2_HS_ | 4.203 | 0.066 | 0.058 | 0.075 | 0.034 | 0.107 | 0.226 | 0.013 | 0.004 | -0.800 | 0.009 | 0.000 | -0.118 |
|  | ^3^TS2_IS_ | 1.291 | -0.029 | -0.031 | -0.036 | -0.014 | -0.014 | 0.014 | 0.004 | 0.005 | 0.708 | -0.001 | 0.016 | 0.110 |
| [(L^2^)Fe^IV^=O]^1+^ | ^7^TS2 | 4.269 | 0.075 | 0.127 | 0.066 | 0.069 | 0.170 | 0.197 | 0.027 | 0.006 | 0.371 | 0.002 | 0.182 | 0.102 |
|  | ^5^­TS2_HS_ | 4.199 | 0.065 | 0.058 | 0.067 | 0.032 | 0.112 | 0.235 | 0.013 | 0.004 | -0.800 | 0.009 | 0.000 | -0.118 |
|  | ^3^TS2_IS_ | 1.300 | -0.029 | -0.031 | -0.034 | -0.013 | -0.015 | 0.006 | 0.004 | 0.006 | 0.706 | -0.002 | 0.014 | 0.109 |

**Table S53.** Mulliken charges of [(L^1,2^)Fe^IV^=O]^1+^ (R, INT and P).

|  | | Fe | N^Py1^ | N^Py2^ | N^3^ | N^7^ | O^1^ | O^2^ | O^3^ | C^1^ | H^1^ |
| --- | --- | --- | --- | --- | --- | --- | --- | --- | --- | --- | --- |
| [(L^1^)Fe^IV^=O]^1+^ | ^5^R_HS_ | 0.361 | -0.158 | -0.191 | -0.084 | -0.154 | -0.465 | -0.561 | -0.445 | 0.321 | - |
|  | ^3^R_lS_ | 0.303 | 0.086 | 0.036 | -0.049 | -0.142 | -0.483 | -0.481 | -0.470 | 0.377 | - |
|  | ^1^R_LS_ | 0.316 | 0.104 | 0.027 | -0.061 | -0.147 | -0.468 | -0.500 | -0.452 | 0.411 | - |
| [(L^2^)Fe^IV^=O]^1+^ | ^5^R_HS_ | 0.392 | -0.201 | -0.143 | -0.161 | -0.125 | -0.409 | -0.580 | -0.349 | 0.399 | - |
|  | ^3^R_lS_ | 0.297 | 0.053 | 0.081 | -0.062 | -0.139 | -0.483 | -0.470 | -0.465 | 0.375 | - |
|  | ^1^R_LS_ | 0.313 | 0.104 | 0.033 | -0.077 | -0.140 | -0.467 | -0.490 | -0.449 | 0.408 | - |
| [(L^1^)Fe^IV^=O]^1+^ | ^5^INT_HS_ | 0.784 | -0.140 | -0.149 | -0.285 | -0.199 | -0.524 | -0.687 | -0.490 | 0.410 | 0.332 |
|  | ^3^INT_lS_ | 0.576 | -0.137 | -0.160 | -0.209 | -0.090 | -0.489 | -0.614 | -0.495 | 0.395 | 0.326 |
|  | ^1^INT_LS_ | 0.223 | 0.103 | 0.025 | -0.075 | -0.098 | -0.490 | -0.634 | -0.512 | 0.369 | 0.319 |
| [(L^2^)Fe^IV^=O]^1+^ | ^5^INT_HS_ | 0.781 | -0.135 | -0.148 | -0.292 | -0.197 | -0.525 | -0.680 | -0.486 | 0.408 | 0.333 |
|  | ^3^INT_lS_ | 0.573 | -0.134 | -0.153 | -0.225 | -0.085 | -0.491 | -0.606 | -0.494 | 0.396 | 0.329 |
|  | ^1^INT_LS_ | 0.216 | 0.106 | 0.028 | -0.083 | -0.094 | -0.489 | -0.628 | -0.508 | 0.368 | 0.322 |
| [(L^1^)Fe^IV^=O]^1+^ | ^5^P_HS_ | 0.605 | -0.198 | -0.203 | -0.323 | -0.185 | -0.557 | - | -0.482 | 0.454 | - |
|  | ^3^P_lS_ | 0.240 | 0.013 | 0.009 | -0.193 | -0.162 | -0.531 | - | -0.490 | 0.377 | - |
|  | ^1^P_LS_ | 0.079 | 0.030 | 0.024 | -0.110 | -0.033 | -0.485 | - | -0.462 | 0.359 | - |
| [(L^2^)Fe^IV^=O]^1+^ | ^5^P_HS_ | 0.602 | -0.198 | -0.206 | -0.331 | -0.184 | -0.556 | - | -0.480 | 0.457 | - |
|  | ^3^P_lS_ | 0.238 | 0.016 | 0.006 | -0.204 | -0.162 | -0.531 | - | -0.285 | -0.339 | - |
|  | ^1^P_LS_ | 0.077 | 0.030 | 0.021 | -0.120 | -0.031 | -0.482 | - | -0.461 | 0.362 | - |

**Table S54.** Mulliken charges of [(L^1,2^)Fe^IV^=O]^1+^ (TS1 and TS2).

|  | | Fe | N^Py1^ | N^Py2^ | N^3^ | N^7^ | O^1^ | O^2^ | O^3^ | C^1^ | C^2^ | H^1^ | C^3^ | O^4^ |
| --- | --- | --- | --- | --- | --- | --- | --- | --- | --- | --- | --- | --- | --- | --- |
| [(L^1^)Fe^IV^=O]^1+^ | ^5^­TS1_HS_ | 0.651 | -0.142 | -0.193 | -0.154 | -0.183 | -0.390 | -0.481 | -0.495 | 0.343 | -0.423 | 0.298 | 0.465 | -0.315 |
|  | ^3^TS1_IS_ | 0.574 | -0.134 | -0.221 | -0.139 | -0.097 | -0.476 | -0.515 | -0.471 | 0.441 | -0.383 | 0.286 | 0.337 | -0.395 |
| [(L^2^)Fe^IV^=O]^1+^ | ^5^­TS1_HS_ | 0.597 | -0.213 | -0.122 | -0.189 | -0.111 | -0.454 | -0.612 | -0.445 | 0.321 | -0.511 | 0.319 | 0.322 | -0.393 |
|  | ^3^TS1_IS_ | 0.291 | 0.078 | -0.039 | -0.068 | -0.087 | -0.478 | -0.533 | -0.481 | 0.396 | -0.358 | 0.267 | 0.321 | -0.388 |
| [(L^1^)Fe^IV^=O]^1+^ | ^7^TS2 | 0.758 | -0.088 | -0.191 | -0.247 | -0.148 | -0.583 | -0.553 | -0.446 | 0.443 | -0.165 | 0.334 | 0.240 | -0.466 |
|  | ^5^­TS2_HS_ | 0.743 | -0.114 | -0.173 | -0.237 | -0.127 | -0.550 | -0.633 | -0.523 | 0.416 | -0.220 | 0.332 | 0.331 | -0.424 |
|  | ^3^TS2_IS_ | 0.277 | 0.074 | -0.011 | -0.081 | 0.101 | -0.527 | -0.581 | -0.523 | 0.389 | -0.203 | 0.324 | 0.321 | -0.420 |
| [(L^2^)Fe^IV^=O]^1+^ | ^7^TS2 | 0.754 | -0.087 | -0.186 | -0.258 | -0.148 | -0.579 | -0.554 | -0.436 | 0.431 | -0.166 | 0.336 | 0.245 | -0.463 |
|  | ^5^­TS2_HS_ | 0.745 | -0.116 | -0.168 | -0.250 | -0.126 | -0.551 | -0.630 | -0.521 | 0.415 | -0.218 | 0.333 | 0.330 | -0.423 |
|  | ^3^TS2_IS_ | 0.276 | 0.084 | -0.012 | -0.095 | -0.098 | -0.529 | -0.580 | -0.418 | 0.389 | -0.198 | 0.325 | 0.320 | -0.418 |

**Table S55.** Computed spin density values of [(L^1,2^)Fe^V^=O]^2+^ (R, INT and P).

|  | | Fe | N^Py1^ | N^Py2^ | N^3^ | N^7^ | O^1^ | O^2^ | O^3^ | C^1^ | H^1^ |
| --- | --- | --- | --- | --- | --- | --- | --- | --- | --- | --- | --- |
| [(L^1^)Fe^V^=O]^2+^ | ^4^R_HS_ | 1.832 | -0.014 | -0.009 | -0.043 | -0.051 | 0.066 | 1.093 | 0.132 | -0.016 | - |
|  | ^2^R_LS_ | 1.265 | -0.023 | -0.020 | -0.058 | -0.033 | -0.346 | 0.825 | -0.669 | 0.074 | - |
| [(L^2^)Fe^V^=O]^2+^ | ^4^R_HS_ | 1.768 | -0.007 | -0.015 | -0.039 | -0.049 | 0.061 | 1.120 | 0.162 | -0.018 | - |
|  | ^2^R_LS_ | 1.242 | -0.020 | -0.020 | -0.054 | -0.032 | -0.321 | 0.845 | -0.699 | 0.072 | - |
| [(L^1^)Fe^V^=O]^2+^ | ^6^­INT_HS_ | 3.912 | 0.067 | 0.068 | -0.076 | -0.124 | -0.020 | 0.927 | 0.424 | -0.018 | -0.004 |
|  | ^4^­INT_IS_ | 1.202 | -0.033 | -0.029 | -0.029 | -0.041 | 0.013 | 0.705 | 0.364 | -0.017 | -0.010 |
|  | ^2^INT_LS_ | 0.391 | -0.002 | -0.021 | -0.052 | -0.031 | 0.061 | 0.690 | 0.123 | -0.017 | -0.010 |
| [(L^2^)Fe^V^=O]^2+^ | ^6^­INT_HS_ | 3.077 | 0.076 | 0.071 | -0.044 | -0.096 | 0.006 | 0.504 | 0.361 | -0.017 | -0.003 |
|  | ^4^­INT_IS_ | 1.188 | -0.033 | -0.030 | -0.027 | -0.040 | 0.012 | 0.503 | 0.377 | -0.017 | -0.010 |
|  | ^2^INT_LS_ | 0.321 | -0.003 | -0.011 | -0.023 | -0.021 | 0.041 | 0.711 | 0.120 | -0.016 | -0.009 |
| [(L^1^)Fe^V^=O]^2+^ | ^6^P_HS_ | 4.235 | 0.073 | 0.073 | 0.123 | 0.101 | 0.156 | - | 0.093 | 0.005 | - |
|  | ^4^P_lS_ | 2.963 | -0.070 | -0.070 | -0.019 | 0.073 | 0.030 | - | 0.056 | 0.014 | - |
|  | ^2^P_LS_ | 1.146 | -0.030 | -0.030 | -0.032 | -0.037 | -0.013 | - | -0.017 | 0.006 | - |
| [(L^2^)Fe^V^=O]^2+^ | ^6^P_HS_ | 4.232 | 0.074 | 0.074 | 0.112 | 0.099 | 0.165 | - | 0.097 | 0.005 | - |
|  | ^4^P_lS_ | 2.963 | -0.074 | -0.075 | -0.005 | 0.068 | 0.035 | - | 0.052 | 0.012 | - |
|  | ^2^P_LS_ | 1.145 | -0.030 | -0.031 | -0.031 | -0.037 | -0.014 | - | -0.018 | 0.006 | - |

**Table S56.** Computed spin density values of [(L^1,2^)Fe^V^=O]^2+^ (TS1 and TS2).

|  | | Fe | N^Py1^ | N^Py2^ | N^3^ | N^7^ | O^1^ | O^2^ | O^3^ | C^1^ | C^2^ | H^1^ | C^3^ | O^4^ |
| --- | --- | --- | --- | --- | --- | --- | --- | --- | --- | --- | --- | --- | --- | --- |
| [(L^1^)Fe^V^=O]^2+^ | ^4^TS1_HS_ | 1.984 | -0.017 | -0.048 | -0.065 | -0.052 | 0.041 | 0.684 | 0.024 | -0.003 | 0.438 | -0.020 | -0.016 | 0.046 |
|  | ^2^TS1_LS_ | 0.819 | -0.013 | -0.018 | -0.038 | -0.038 | -0.093 | 0.317 | -0.018 | 0.008 | 0.075 | -0.008 | -0.004 | 0.009 |
| [(L^2^)Fe^V^=O]^2+^ | ^4^TS1_HS_ | 1.247 | -0.019 | -0.020 | -0.028 | -0.039 | 0.021 | 0.960 | 0.330 | 0.490 | 0.490 | -0.018 | -0.021 | 0.063 |
|  | ^2^TS1_LS_ | 0.817 | -0.013 | -0.017 | -0.035 | -0.038 | -0.098 | 0.324 | -0.019 | 0.009 | 0.069 | -0.007 | -0.003 | 0.008 |
| [(L^1^)Fe^V^=O]^2+^ | ^6^TS2_HS_ | 3.695 | 0.095 | 0.087 | 0.000 | -0.086 | 0.108 | 0.103 | 0.039 | -0.001 | 0.845 | 0.005 | -0.018 | 0.118 |
|  | ^4^TS2_IS_ | 2.141 | -0.040 | -0.046 | -0.056 | -0.032 | 0.059 | 0.023 | 0.043 | -0.001 | 0.831 | -0.002 | -0.015 | 0.111 |
|  | ^2^TS2_LS_ | 1.790 | -0.043 | -0.037 | -0.046 | -0.053 | 0.030 | 0.236 | 0.038 | -0.005 | -0.872 | -0.001 | 0.012 | -0.092 |
| [(L^2^)Fe^V^=O]^2+^ | ^6^TS2_HS_ | 3.703 | 0.092 | 0.082 | 0.010 | -0.078 | 0.117 | 0.081 | 0.041 | -0.001 | 0.833 | 0.006 | -0.018 | 0.115 |
|  | ^4^TS2_IS_ | 2.149 | -0.039 | -0.047 | -0.052 | -0.028 | 0.053 | 0.020 | 0.045 | -0.002 | 0.824 | -0.002 | -0.016 | 0.107 |
|  | ^2^TS2_LS_ | 1.879 | -0.061 | -0.049 | -0.055 | -0.058 | -0.028 | 0.240 | 0.013 | 0.000 | -0.818 | 0.007 | 0.008 | -0.107 |

**Table S57.** Mulliken charges of [(L^1,2^)Fe^V^=O]^2+^ (R, INT and P).

|  | | Fe | N^Py1^ | N^Py2^ | N^3^ | N^7^ | O^1^ | O^2^ | O^3^ | C^1^ | H^1^ |
| --- | --- | --- | --- | --- | --- | --- | --- | --- | --- | --- | --- |
| [(L^1^)Fe^V^=O]^2+^ | ^4^R_HS_ | 0.089 | 0.124 | 0.112 | -0.058 | -0.066 | -0.424 | -0.214 | -0.325 | 0.337 | - |
|  | ^2^R_LS_ | 0.116 | 0.072 | 0.073 | 0.036 | -0.124 | -0.349 | -0.430 | -0.156 | 0.443 | - |
| [(L^2^)Fe^V^=O]^2+^ | ^4^R_HS_ | 0.071 | 0.113 | 0.120 | -0.079 | -0.048 | -0.419 | -0.203 | -0.312 | 0.336 | - |
|  | ^2^R_LS_ | 0.104 | 0.076 | 0.071 | 0.025 | -0.114 | -0.356 | -0.419 | -0.142 | 0.446 | - |
| [(L^1^)Fe^V^=O]^2+^ | ^6^­INT_HS_ | 0.523 | -0.119 | -0.126 | -0.211 | -0.096 | -0.442 | -0.474 | -0.301 | 0.450 | 0.369 |
|  | ^4^­INT_IS_ | 0.036 | 0.090 | 0.041 | -0.077 | -0.005 | -0.438 | -0.410 | -0.308 | 0.389 | 0.365 |
|  | ^2^INT_LS_ | 0.013 | 0.119 | 0.046 | -0.042 | -0.049 | -0.427 | -0.367 | -0.319 | 0.399 | 0.382 |
| [(L^2^)Fe^V^=O]^2+^ | ^6^­INT_HS_ | 0.472 | -0.159 | -0.209 | -0.183 | -0.045 | -0.422 | -0.425 | -0.301 | 0.450 | 0.381 |
|  | ^4^­INT_IS_ | 1.188 | 1.188 | 1.188 | 1.188 | 1.188 | 1.188 | 1.188 | -0.298 | 0.384 | 0.367 |
|  | ^2^INT_LS_ | -0.033 | -0.033 | -0.033 | -0.033 | -0.033 | -0.033 | -0.033 | -0.299 | 0.398 | 0.384 |
| [(L^1^)Fe^V^=O]^2+^ | ^6^P_HS_ | -0.030 | -0.030 | -0.030 | -0.030 | -0.030 | -0.030 | - | -0.409 | 0.479 | - |
|  | ^4^P_lS_ | -0.027 | -0.027 | -0.027 | -0.027 | -0.027 | -0.027 | - | -0.413 | 0.378 | - |
|  | ^2^P_LS_ | -0.040 | -0.040 | -0.040 | -0.040 | -0.040 | -0.040 | - | -0.375 | 0.376 | - |
| [(L^2^)Fe^V^=O]^2+^ | ^6^P_HS_ | 0.012 | 0.012 | 0.012 | 0.012 | 0.012 | 0.012 | - | -0.406 | 0.482 | - |
|  | ^4^P_lS_ | 0.503 | 0.503 | 0.503 | 0.503 | 0.503 | 0.503 | - | -0.422 | 0.411 | - |
|  | ^2^P_LS_ | 0.047 | 0.075 | 0.072 | -0.134 | -0.007 | -0.398 | - | -0.373 | 0.378 | - |

**Table S58.** Mulliken charges of [(L^1,2^)Fe^V^=O]^2+^ (TS1 and TS2).

|  | | Fe | N^Py1^ | N^Py2^ | N^3^ | N^7^ | O^1^ | O^2^ | O^3^ | C^1^ | C^2^ | H^1^ | C^3^ | O^4^ |
| --- | --- | --- | --- | --- | --- | --- | --- | --- | --- | --- | --- | --- | --- | --- |
| [(L^1^)Fe^V^=O]^2+^ | ^4^TS1_HS_ | 0.187 | 0.102 | 0.058 | -0.042 | -0.093 | -0.476 | -0.408 | -0.401 | 0.426 | -0.293 | 0.274 | 0.349 | -0.387 |
|  | ^2^TS1_LS_ | 0.141 | 0.113 | 0.040 | -0.008 | -0.092 | -0.465 | -0.383 | -0.400 | 0.437 | -0.265 | 0.254 | 0.354 | -0.377 |
| [(L^2^)Fe^V^=O]^2+^ | ^4^TS1_HS_ | 0.077 | 0.073 | 0.090 | -0.109 | 0.011 | -0.436 | -0.366 | -0.328 | 0.396 | -0.322 | 0.273 | 0.375 | -0.395 |
|  | ^2^TS1_LS_ | 0.140 | 0.108 | 0.046 | -0.030 | -0.081 | -0.463 | -0.376 | -0.395 | 0.434 | -0.263 | 0.253 | 0.354 | -0.376 |
| [(L^1^)Fe^V^=O]^2+^ | ^6^TS2_HS_ | 0.578 | -0.070 | -0.133 | -0.188 | -0.077 | -0.468 | -0.524 | -0.405 | 0.397 | -0.213 | 0.396 | 0.157 | -0.409 |
|  | ^4^TS2_IS_ | 0.199 | 0.134 | 0.062 | -0.092 | -0.071 | -0.463 | -0.507 | -0.398 | 0.371 | -0.207 | 0.356 | 0.354 | -0.420 |
|  | ^2^TS2_LS_ | 0.159 | 0.126 | 0.063 | -0.054 | -0.027 | -0.472 | -0.504 | -0.423 | 0.373 | -0.229 | 0.384 | 0.388 | -0.428 |
| [(L^2^)Fe^V^=O]^2+^ | ^6^TS2_HS_ | 0.576 | -0.062 | -0.127 | -0.212 | -0.073 | -0.470 | -0.518 | -0.403 | 0.397 | -0.208 | 0.398 | 0.375 | -0.409 |
|  | ^4^TS2_IS_ | 0.189 | 0.141 | 0.066 | -0.111 | -0.062 | -0.461 | -0.503 | -0.393 | 0.367 | -0.204 | 0.358 | 0.355 | -0.409 |
|  | ^2^TS2_LS_ | 0.152 | 0.109 | 0.052 | -0.053 | -0.039 | -0.482 | -0.519 | -0.448 | 0.394 | -0.215 | 0.381 | 0.355 | -0.400 |

**Table S59.** DFT ⟨S²⟩ Values and DLPNO-CCSD(T) Diagnostics

| Spin State | | UB3LYP/def2-TZVP (solvent) | DLPNO-CCSD(T)/cc-PVQZ//UB3LYP/def2-TZVP |
| --- | --- | --- | --- |
|  |  | ⟨S²⟩ (DFT) | T₁ Diagnostic |
| [(L^1^)Fe^IV^=O]^1+^  (O *trans* N^3^) | ^5^R_HS_ | 6.01 | 0.035 |
|  | ^3^R_IS_ | 2.00 | 0.025 |
|  | ^1^R_LS_ | 0.00 | 0.022 |
| [(L^1^)Fe^IV^=O]^1+^  (O *trans* N^7^) | ^5^R_HS_ | 6.04 | 0.034 |
|  | ^3^R_IS_ | 2.02 | 0.025 |
|  | ^1^R_LS_ | 0.00 | 0.021 |
| [(L^2^)Fe^IV^=O]^1+^  (O *trans* N^3^) | ^5^R_HS_ | 6.05 | 0.034 |
|  | ^3^R_IS_ | 2.05 | 0.025 |
|  | ^1^R_LS_ | 0.00 | 0.022 |
| [(L^2^)Fe^IV^=O]^1+^  (O *trans* N^7^) | ^5^R_HS_ | 6.04 | 0.033 |
|  | ^3^R_IS_ | 2.05 | 0.024 |
|  | ^1^R_LS_ | 0.00 | 0.022 |
| [(L^1^)Fe^V^=O]^2+^  (O *trans* N^3^) | ^4^R_HS_ | 3.75 | 0.022 |
|  | ^2^R_LS_ | 0.86 | 0.023 |
| [(L^1^)Fe^V^=O]^2+^  (O *trans* N^7^) | ^4^R_HS_ | 3.74 | 0.021 |
|  | ^2^R_LS_ | 0.85 | 0.023 |
| [(L^2^)Fe^V^=O]^2+^  (O *trans* N^3^) | ^4^R_HS_ | 3.78 | 0.023 |
|  | ^2^R_LS_ | 0.87 | 0.025 |
| [(L^2^)Fe^V^=O]^2+^  (O *trans* N^3^) | ^4^R_HS_ | 3.78 | 0.023 |
|  | ^2^R_LS_ | 0.86 | 0.024 |

# Cartesian coordinates (O trans to N^3^ isomer)

[(L^1^)Fe^IV^=O]^1+^ (^5^R_HS_)

Fe 1.01752 -0.66256 0.51812

N -0.35175 -2.30946 0.13804

C -1.34614 -2.01233 -0.71968

C -0.81285 -4.63851 -0.10876

H -0.57047 -5.66289 0.15254

C -0.07955 -3.58731 0.43582

H 0.75016 -3.74250 1.11702

C -2.12398 -3.00700 -1.30710

H -2.92897 -2.72825 -1.97906

C -1.85086 -4.34050 -0.99399

H -2.43844 -5.13725 -1.44002

N -0.60402 0.31056 1.84434

N -0.24148 0.13334 -1.04893

C 0.33213 -0.20947 -2.38245

H 0.40364 -1.29129 -2.47798

H -0.30502 0.20124 -3.17626

C -1.58338 -0.53057 -0.93294

H -2.12922 -0.38149 -1.86990

O -4.32826 -0.43529 -1.26986

H -5.28062 -0.60914 -1.30818

C -2.48635 0.04320 0.19846

C -1.95464 -0.26004 1.60834

H -2.65656 0.16418 2.34036

H -1.89715 -1.33500 1.78586

C -3.89134 -0.57863 0.07833

H -3.85414 -1.63937 0.36824

H -4.55366 -0.05253 0.77425

C -2.56212 1.56843 0.00019

H -2.88915 1.77981 -1.02298

O -3.49076 2.10424 0.93662

C -0.21053 0.04183 3.24858

H -0.15495 -1.03579 3.40377

N 1.89327 1.28256 0.18261

C 1.20708 2.05231 -0.68620

C 3.80561 2.68841 -0.05899

H 4.83661 2.89812 0.20284

C 3.16478 1.57664 0.48593

H 3.66037 0.88925 1.15926

C 1.78200 3.17110 -1.27804

H 1.20164 3.77180 -1.97095

C 3.10435 3.49329 -0.95460

H 3.58080 4.35792 -1.40677

H 1.34074 0.19174 -2.45607

C -0.23078 1.62970 -0.90846

H -0.60190 2.07356 -1.83898

O -1.71134 4.05378 -1.12785

H -1.76507 5.01997 -1.18378

C -1.15135 2.15892 0.22786

C -0.66733 1.77917 1.63634

H -1.36750 2.21062 2.36533

H 0.32430 2.18732 1.83740

C -1.21178 3.69451 0.15614

H -0.21303 4.11833 0.33219

H -1.87289 4.05009 0.95773

H -3.93523 2.85830 0.52204

H 0.78006 0.45897 3.42882

H -0.93865 0.48485 3.94209

O 1.84074 -1.15215 1.82515

O 2.17704 -1.40885 -0.73470

C 3.46299 -1.70544 -0.47116

O 4.34942 -0.88340 -0.59967

C 3.68392 -3.12901 -0.01192

H 3.30511 -3.20967 1.01405

H 3.12059 -3.82752 -0.63856

H 4.74690 -3.37490 -0.02581

[(L^1^)Fe^IV^=O]^1+^ (^3^R_IS_)

Fe -0.74222 0.81542 0.67254

N 0.70528 2.13542 0.36872

C 1.62118 1.77919 -0.55623

C 1.57284 4.35158 0.41713

H 1.52082 5.35726 0.81946

C 0.66043 3.38889 0.84042

H -0.13214 3.58696 1.55256

C 2.55622 2.69298 -1.03147

H 3.28457 2.37197 -1.76888

C 2.53192 3.99805 -0.53347

H 3.24894 4.73131 -0.88990

N 0.64899 -0.53325 1.79425

N 0.13479 -0.06678 -1.03756

C -0.42383 0.46411 -2.31367

H -0.27725 1.54263 -2.34736

H 0.08354 -0.01236 -3.16256

C 1.58202 0.31994 -0.96333

H 2.03626 0.20082 -1.95137

O 4.20854 -0.30561 -1.57470

H 5.16947 -0.34556 -1.69134

C 2.42654 -0.53539 0.02171

C 2.07844 -0.25304 1.49055

H 2.71089 -0.88632 2.12806

H 2.27972 0.78689 1.75233

C 3.91923 -0.21270 -0.18384

H 4.13465 0.79770 0.19521

H 4.50343 -0.93274 0.39913

C 2.14718 -2.01416 -0.30268

H 2.31924 -2.18183 -1.37086

O 3.02302 -2.82382 0.47267

C 0.42430 -0.29822 3.24358

H 0.61562 0.74991 3.47081

N -2.01950 -0.66354 0.44876

C -1.67768 -1.57197 -0.49038

C -4.18783 -1.63229 0.63405

H -5.16716 -1.62224 1.09899

C -3.24034 -0.68152 0.99932

H -3.43314 0.09925 1.72551

C -2.58765 -2.52598 -0.92781

H -2.29497 -3.22944 -1.70041

C -3.86293 -2.55507 -0.35780

H -4.59216 -3.28786 -0.68920

H -1.49405 0.26576 -2.34787

C -0.22981 -1.51798 -0.93803

H -0.12422 -1.98850 -1.92167

O 0.75479 -4.17834 -1.47162

H 0.57657 -5.12144 -1.60711

C 0.67042 -2.32484 0.04023

C 0.40743 -1.97264 1.51062

H 1.07963 -2.57477 2.13671

H -0.61837 -2.20771 1.79880

C 0.40556 -3.82922 -0.13684

H -0.64838 -4.05393 0.07837

H 1.01835 -4.37456 0.59354

H 3.21771 -3.62436 -0.03735

H -0.61634 -0.51231 3.48432

H 1.08901 -0.94076 3.83683

O -1.32895 1.39337 2.06898

O -1.72290 2.07801 -0.36967

C -2.88825 1.97287 -0.97553

O -3.44257 0.93118 -1.31228

C -3.49143 3.34123 -1.26055

H -3.76299 3.82001 -0.31310

H -2.75707 3.98672 -1.75269

H -4.37861 3.23446 -1.88687

[(L^1^)Fe^IV^=O]^1+^ (^1^R_LS_)

Fe -0.61626 -0.80554 0.78145

N -1.99242 0.58714 0.42534

C -1.66910 1.52818 -0.48615

C -4.19907 1.46876 0.57321

H -5.18082 1.42054 1.02342

C -3.22994 0.55802 0.95330

H -3.42245 -0.20454 1.69433

C -2.60277 2.45592 -0.91878

H -2.31905 3.18954 -1.66129

C -3.88580 2.42547 -0.38482

H -4.63023 3.13999 -0.71113

N 0.68856 0.47357 1.81429

N 0.14030 0.04993 -1.00103

C -0.46874 -0.51541 -2.23839

H -1.53481 -0.30263 -2.26098

H -0.00979 -0.06410 -3.12106

C -0.22489 1.50108 -0.91743

H -0.11355 1.96629 -1.89802

O 0.44016 4.15379 -1.44228

H 0.48633 5.11165 -1.54077

C 0.65449 2.30983 0.07576

C 0.40880 1.91766 1.53726

H 1.07501 2.51167 2.16770

H -0.60877 2.13575 1.85170

C 0.33544 3.80932 -0.06946

H -0.67034 4.01926 0.31128

H 1.04655 4.36852 0.54064

C 2.13545 2.04395 -0.27574

H 2.30427 2.26582 -1.32982

O 2.96205 2.85301 0.54207

C 0.55242 0.26538 3.28887

H -0.46611 0.47151 3.60034

N 0.78133 -2.13563 0.29973

C 1.66601 -1.75676 -0.64531

C 1.67481 -4.34046 0.27182

H 1.65201 -5.34678 0.66607

C 0.78234 -3.40046 0.75466

H 0.05855 -3.64281 1.51878

C 2.57124 -2.65761 -1.18054

H 3.25846 -2.32982 -1.94874

C 2.57581 -3.96851 -0.71744

H 3.27272 -4.68914 -1.12497

H -0.32139 -1.59134 -2.27280

C 1.60015 -0.29416 -1.00120

H 2.01605 -0.12742 -1.99602

O 4.21837 0.48367 -1.61069

H 5.17163 0.50903 -1.75252

C 2.44004 0.55258 -0.00682

C 2.12239 0.23275 1.45791

H 2.73429 0.88059 2.09029

H 2.37069 -0.79422 1.71339

C 3.93541 0.26853 -0.23680

H 4.17366 -0.75948 0.05748

H 4.51218 0.93994 0.40204

H 3.54141 3.39773 -0.00096

H 0.78954 -0.76251 3.54174

H 1.23930 0.94008 3.80362

O -1.19653 -1.47127 2.14182

O -1.93773 -2.22346 -0.14523

C -2.98475 -1.97987 -1.08826

O -2.76691 -1.51141 -2.20154

C -4.40383 -2.34449 -0.61406

H -4.44495 -3.38804 -0.38122

H -4.64573 -1.77454 0.25860

H -5.10680 -2.12423 -1.39009

[(L^2^)Fe^IV^=O]^1+^ (^1^R_LS_)

Fe -1.23139 -0.92791 0.59885

N -2.20673 0.74851 0.43059

C -1.67122 1.63256 -0.43867

C -4.13585 2.12352 0.67622

H -5.10287 2.28469 1.13911

C -3.40883 0.97666 0.97680

H -3.76627 0.20990 1.65325

C -2.35431 2.78450 -0.80322

H -1.90828 3.47558 -1.51057

C -3.60804 3.03185 -0.23928

H -4.16187 3.92464 -0.51183

N 0.30659 0.15038 1.84988

N -0.10817 -0.14252 -1.02302

C -0.68429 -0.51393 -2.34836

H -1.70336 -0.13537 -2.41378

H -0.06514 -0.08821 -3.14933

C -0.24209 1.34176 -0.86476

H -0.03077 1.82689 -1.82393

O 1.15297 3.85868 -1.17689

C 0.73364 1.95206 0.18387

C 0.33541 1.61868 1.62596

H 1.06258 2.08217 2.30235

H -0.64019 2.03442 1.86843

C 0.71428 3.48212 0.03639

C 2.14678 1.42391 -0.10869

H 2.38703 1.60939 -1.16199

O 3.04514 2.11029 0.74161

C -0.02853 -0.08462 3.28070

H -1.03853 0.27251 3.47387

N 0.02036 -2.46977 0.29797

C 1.01653 -2.24177 -0.58096

C 0.48382 -4.80258 0.26626

H 0.24524 -5.79920 0.62111

C -0.25934 -3.71099 0.71036

H -1.09945 -3.78583 1.39217

C 1.78564 -3.28501 -1.08080

H 2.58221 -3.08460 -1.78912

C 1.51644 -4.58537 -0.64554

H 2.10494 -5.41716 -1.01990

H -0.71674 -1.59853 -2.43350

C 1.24524 -0.77580 -0.90912

H 1.76812 -0.68588 -1.87092

O 4.30998 0.00167 -0.84065

C 2.14451 -0.09568 0.16756

C 1.67400 -0.37393 1.60393

H 2.37666 0.11493 2.29101

H 1.69010 -1.44066 1.81665

C 3.55059 -0.69522 0.02765

H 3.93615 2.01058 0.37000

H 0.00209 -1.15443 3.48584

H 0.69056 0.44186 3.92189

O -1.85869 -1.51866 1.97634

O -2.30964 -1.94365 -0.56412

O 3.91940 -1.69286 0.60285

O 0.33007 4.24065 0.89478

C -3.43611 -1.61815 -1.17617

O -3.79147 -0.48363 -1.47410

C -4.25498 -2.85093 -1.52418

H -3.63693 -3.57708 -2.06179

H -4.59361 -3.33329 -0.60055

H -5.11619 -2.56727 -2.13113

C 5.64181 -0.52636 -1.07046

H 6.10168 0.15287 -1.78691

H 6.20193 -0.54956 -0.13310

H 5.57686 -1.53812 -1.47695

C 1.21307 5.28778 -1.40029

H 1.89409 5.74797 -0.68089

H 1.58120 5.40680 -2.41855

H 0.21958 5.72892 -1.28873

[(L^2^)Fe^IV^=O]^1+^ (^3^R_IS_)

Fe -1.22181 -0.90515 0.61449

N -2.19036 0.79312 0.41486

C -1.63775 1.66863 -0.45099

C -4.11194 2.18397 0.62492

H -5.08622 2.35163 1.06987

C -3.39906 1.03147 0.93974

H -3.77500 0.26519 1.60704

C -2.30369 2.82583 -0.82905

H -1.84111 3.50949 -1.53302

C -3.56267 3.08669 -0.28267

H -4.10441 3.98391 -0.56485

N 0.33278 0.11012 1.85026

N -0.11363 -0.13478 -1.02580

C -0.70945 -0.49542 -2.34440

H -1.72283 -0.09997 -2.39802

H -0.09052 -0.08365 -3.15272

C -0.21221 1.35150 -0.86198

H 0.02138 1.83588 -1.81629

O 1.20337 3.85278 -1.13417

C 0.76911 1.93037 0.19975

C 0.37120 1.58147 1.64036

H 1.10298 2.03411 2.31903

H -0.60159 2.00035 1.88848

C 0.76904 3.46290 0.07664

C 2.17374 1.38854 -0.10754

H 2.40981 1.58347 -1.16008

O 3.08707 2.05156 0.74504

C 0.00484 -0.14426 3.27816

H -0.98687 0.25161 3.49279

N -0.02555 -2.45631 0.30748

C 0.97145 -2.25296 -0.57896

C 0.39922 -4.79844 0.29121

H 0.14548 -5.78746 0.65629

C -0.32306 -3.69207 0.73026

H -1.16021 -3.75733 1.41529

C 1.71873 -3.31421 -1.07393

H 2.51487 -3.13243 -1.78739

C 1.43039 -4.60609 -0.62792

H 2.00304 -5.45054 -0.99832

H -0.76422 -1.57968 -2.42696

C 1.22778 -0.79446 -0.91800

H 1.74459 -0.72065 -1.88442

O 4.31356 -0.04822 -0.86935

C 2.15263 -0.13399 0.15128

C 1.69640 -0.41794 1.59104

H 2.40792 0.06521 2.27290

H 1.71173 -1.48576 1.79869

C 3.54794 -0.75275 -0.01339

H 3.97410 1.94483 0.36594

H -0.00917 -1.21874 3.45675

H 0.75025 0.33702 3.92480

O -1.97525 -1.42062 1.95189

O -2.36294 -1.91812 -0.53121

O 3.90347 -1.77054 0.53480

O 0.40258 4.21275 0.95007

C -3.46452 -1.56705 -1.16490

O -3.78691 -0.42600 -1.48124

C -4.31840 -2.77774 -1.51211

H -3.70991 -3.54827 -1.99591

H -4.71925 -3.21149 -0.58911

H -5.13987 -2.48015 -2.16583

C 5.63547 -0.59180 -1.11997

H 6.10191 0.09702 -1.82286

H 6.20047 -0.64621 -0.18686

H 5.55185 -1.59230 -1.55018

C 1.27972 5.28441 -1.33554

H 1.97239 5.72458 -0.61474

H 1.64097 5.41491 -2.35486

H 0.29279 5.73597 -1.20860

[(L^2^)Fe^IV^=O]^1+^ (^5^R_HS_)

Fe -1.22775 -1.01869 0.68297

N -2.26583 0.79208 0.41830

C -1.72819 1.62309 -0.49559

C -4.27824 2.06863 0.42382

H -5.28329 2.20964 0.80506

C -3.51237 0.99294 0.86402

H -3.87716 0.26162 1.57633

C -2.43728 2.70607 -0.99687

H -1.98305 3.35914 -1.73471

C -3.73448 2.93069 -0.52624

H -4.31237 3.76878 -0.90318

N 0.38375 0.15584 1.91084

N -0.12472 -0.15393 -0.96323

C -0.73730 -0.57234 -2.25972

H -1.77978 -0.25668 -2.28088

H -0.17944 -0.12405 -3.09261

C -0.28064 1.33476 -0.84008

H -0.03077 1.79440 -1.80283

O 0.94986 3.91714 -1.12776

C 0.65880 1.97874 0.22400

C 0.30052 1.61711 1.67373

H 0.99607 2.14460 2.33762

H -0.70376 1.95572 1.92032

C 0.55280 3.50721 0.09006

C 2.09408 1.53068 -0.09255

H 2.31073 1.74182 -1.14605

O 2.96987 2.24982 0.75355

C 0.09035 -0.11638 3.33859

H -0.92248 0.21655 3.56447

N 0.26055 -2.51576 0.32977

C 1.16495 -2.19054 -0.61376

C 0.87116 -4.81375 0.15347

H 0.72704 -5.83789 0.47962

C 0.10164 -3.79184 0.70412

H -0.66915 -3.96799 1.44656

C 1.95753 -3.15730 -1.22110

H 2.67838 -2.87522 -1.98112

C 1.80959 -4.48912 -0.82646

H 2.41804 -5.26325 -1.28358

H -0.71462 -1.65801 -2.33038

C 1.27165 -0.70336 -0.90076

H 1.75228 -0.54934 -1.87678

O 4.30781 0.23213 -0.88503

C 2.16822 0.00949 0.16294

C 1.76195 -0.29654 1.61457

H 2.46785 0.22300 2.27662

H 1.83793 -1.36236 1.81919

C 3.59577 -0.52421 -0.02637

H 3.85949 2.19620 0.36928

H 0.14195 -1.19112 3.51466

H 0.80988 0.40559 3.98408

O -1.94474 -1.51409 2.04475

O -2.30452 -2.10884 -0.48331

O 4.01853 -1.52343 0.50772

O 0.14255 4.23775 0.96006

C -3.47670 -1.89489 -1.05592

O -3.90133 -0.78859 -1.37262

C -4.24662 -3.17661 -1.31593

H -3.61241 -3.89760 -1.84196

H -4.52538 -3.62831 -0.35716

H -5.14393 -2.96774 -1.90062

C 5.65667 -0.22949 -1.15434

H 6.07297 0.49052 -1.85751

H 6.23489 -0.25593 -0.22811

H 5.62827 -1.23060 -1.59031

C 0.92298 5.34889 -1.33940

H 1.58490 5.84302 -0.62473

H 1.27029 5.49759 -2.36112

H -0.09330 5.72957 -1.21132

[(L^1^)Fe^V^=O]^2+^ (^4^R_HS_)

Fe -1.04564 0.24933 0.41998

N -0.51114 2.12251 0.20422

C 0.53653 2.30706 -0.63700

C -0.92990 4.46369 0.26900

H -1.52521 5.28915 0.64374

C -1.24083 3.16312 0.64922

H -2.07243 2.91771 1.30059

C 0.88854 3.58303 -1.05847

H 1.73663 3.70578 -1.72416

C 0.14433 4.67507 -0.60017

H 0.39960 5.68059 -0.92039

N 0.65307 -0.12900 1.75557

N 0.28670 -0.04281 -1.12506

C -0.32913 0.09606 -2.48131

H -0.74933 1.09399 -2.59187

H 0.43687 -0.07622 -3.24518

C 1.31033 1.05015 -0.96190

H 1.85736 1.17218 -1.90029

O 3.91659 1.86108 -1.30982

H 4.73931 2.37078 -1.37606

C 2.36416 0.77738 0.14706

C 1.76115 0.85740 1.55624

H 2.54956 0.63601 2.28573

H 1.37417 1.85193 1.77992

C 3.48437 1.83258 0.04213

H 3.10699 2.81500 0.36361

H 4.28803 1.53958 0.72748

C 2.94481 -0.63998 -0.07310

H 3.32843 -0.72498 -1.09460

O 3.96833 -0.87215 0.88211

C 0.17114 -0.02257 3.16451

H -0.23151 0.97546 3.33706

N -1.36259 -1.66900 0.19642

C -0.48856 -2.27817 -0.64511

C -2.72059 -3.62177 0.26780

H -3.60559 -4.12166 0.64611

C -2.45853 -2.31031 0.64899

H -3.11257 -1.73813 1.29653

C -0.70493 -3.58385 -1.06552

H 0.01392 -4.05145 -1.73041

C -1.83756 -4.26353 -0.60458

H -2.02765 -5.28362 -0.92410

H -1.13523 -0.62708 -2.59164

C 0.74670 -1.46847 -0.96566

H 1.19105 -1.80864 -1.90448

O 2.74774 -3.31823 -1.31875

H 3.28236 -4.12485 -1.38631

C 1.81517 -1.67466 0.14393

C 1.23412 -1.49379 1.55323

H 2.04043 -1.63247 2.28358

H 0.45908 -2.22843 1.77351

C 2.37826 -3.10691 0.03569

H 1.62031 -3.83459 0.36253

H 3.23486 -3.18351 0.71498

H 4.80760 -1.03923 0.42879

H -0.61664 -0.75566 3.33664

H 1.00414 -0.20772 3.85231

O -2.05102 0.46522 1.75079

O -2.39444 0.58144 -0.79211

C -3.67186 0.67539 -0.42667

O -4.06233 0.55017 0.72820

C -4.60112 0.94398 -1.58707

H -5.62894 1.01562 -1.22992

H -4.30746 1.87667 -2.07968

H -4.51258 0.13753 -2.32215

[(L^1^)Fe^V^=O]^2+^ (^2^R_LS_)

Fe 0.66060 0.85044 0.61465

N 2.04691 -0.52782 0.39299

C 1.75483 -1.47561 -0.52915

C 4.26910 -1.35454 0.58961

H 5.24615 -1.27911 1.05371

C 3.26972 -0.45573 0.94453

H 3.42589 0.34064 1.66250

C 2.71724 -2.38463 -0.94459

H 2.45608 -3.13126 -1.68721

C 3.99542 -2.31870 -0.38118

H 4.76688 -3.01393 -0.69720

N -0.59674 -0.54359 1.79309

N -0.14377 -0.07500 -1.02946

C 0.38822 0.50474 -2.30952

H 1.46583 0.36214 -2.35101

H -0.09695 -0.00174 -3.15053

C 0.30415 -1.51248 -0.94458

H 0.19320 -1.97260 -1.92980

O -0.26340 -4.15428 -1.49793

H -0.17744 -5.11168 -1.62932

C -0.51928 -2.36673 0.05844

C -0.24744 -1.97073 1.51542

H -0.86739 -2.59804 2.16785

H 0.79426 -2.12598 1.79660

C -0.13592 -3.85021 -0.11784

H 0.89037 -4.01947 0.24013

H -0.81195 -4.45026 0.50298

C -2.02618 -2.16996 -0.23983

H -2.22730 -2.40621 -1.28928

O -2.78535 -2.99560 0.62932

C -0.37659 -0.27760 3.24902

H 0.67460 -0.43677 3.49310

N -0.85665 2.08633 0.29136

C -1.76812 1.65251 -0.60932

C -1.86729 4.23814 0.33423

H -1.87725 5.24920 0.72640

C -0.88114 3.34905 0.74978

H -0.09599 3.62126 1.44491

C -2.76984 2.49526 -1.07397

H -3.49064 2.11669 -1.79123

C -2.81981 3.80729 -0.59263

H -3.59048 4.48709 -0.94275

H 0.17074 1.57093 -2.33949

C -1.62740 0.19544 -0.97382

H -2.05272 0.00272 -1.96190

O -4.17743 -0.60274 -1.55052

H -5.13401 -0.68146 -1.69137

C -2.39588 -0.69471 0.03702

C -2.04866 -0.35193 1.49156

H -2.61828 -1.01760 2.15207

H -2.31359 0.67489 1.74558

C -3.91096 -0.47693 -0.16187

H -4.19931 0.51670 0.21267

H -4.44024 -1.23094 0.43141

H -3.24655 -3.67574 0.11668

H -0.65595 0.75144 3.47770

H -0.99303 -0.96173 3.84367

O 1.17611 1.44581 2.09555

O 1.59360 2.15912 -0.30430

C 2.80841 2.15709 -0.91052

O 3.34441 1.17297 -1.37373

C 3.36796 3.56223 -0.97422

H 2.65435 4.23141 -1.46471

H 3.53107 3.94074 0.04054

H 4.30892 3.54793 -1.52601

[(L^2^)Fe^V^=O]^2+^ (^4^R_HS_)

Fe -1.20067 -0.88870 0.55623

N -2.17304 0.81019 0.36209

C -1.61312 1.68808 -0.50290

C -4.08531 2.20703 0.59124

H -5.05412 2.38077 1.04612

C -3.38046 1.04866 0.89925

H -3.76513 0.29063 1.57108

C -2.27622 2.84911 -0.86905

H -1.81253 3.53791 -1.56695

C -3.53401 3.11087 -0.31651

H -4.07291 4.01201 -0.59132

N 0.32074 0.07696 1.82868

N -0.11771 -0.14158 -1.03475

C -0.73527 -0.51188 -2.35340

H -1.73950 -0.09709 -2.40884

H -0.11049 -0.11204 -3.15956

C -0.18931 1.35827 -0.88827

H 0.07543 1.81771 -1.84542

O 1.24617 3.85863 -1.09354

C 0.77908 1.91681 0.18964

C 0.35329 1.55542 1.61834

H 1.08134 1.98802 2.31279

H -0.61767 1.97422 1.87065

C 0.77508 3.45688 0.08993

C 2.19173 1.37505 -0.08842

H 2.46665 1.59835 -1.12534

O 3.06326 2.00321 0.82311

C -0.00928 -0.18521 3.26614

H -0.99142 0.23058 3.49439

N -0.04142 -2.45833 0.22565

C 0.97550 -2.25698 -0.64380

C 0.36229 -4.80152 0.21702

H 0.09784 -5.78830 0.58088

C -0.36166 -3.69348 0.64520

H -1.20625 -3.76596 1.31973

C 1.71872 -3.32457 -1.12656

H 2.53127 -3.15059 -1.82255

C 1.40969 -4.61504 -0.68666

H 1.98117 -5.46402 -1.04824

H -0.79700 -1.59597 -2.42910

C 1.24043 -0.79862 -0.94959

H 1.73677 -0.68930 -1.92091

O 4.36896 -0.03820 -0.76922

C 2.15843 -0.15525 0.13121

C 1.68399 -0.46704 1.55826

H 2.37980 0.00969 2.25875

H 1.69202 -1.53621 1.75788

C 3.55645 -0.78985 -0.02051

H 3.96784 1.94821 0.47364

H -0.00736 -1.26007 3.44959

H 0.74154 0.29436 3.90431

O -1.90839 -1.38070 1.98800

O -2.39403 -1.86249 -0.46545

O 3.84213 -1.85986 0.46667

O 0.36375 4.17314 0.97075

C -3.55517 -1.51709 -1.08180

O -3.78997 -0.41378 -1.52683

C -4.47817 -2.71129 -1.18325

H -3.96779 -3.54279 -1.67902

H -4.75662 -3.04811 -0.17879

H -5.36945 -2.42919 -1.74551

C 5.71103 -0.57171 -0.99163

H 6.21184 0.16883 -1.61232

H 6.21872 -0.69751 -0.03359

H 5.64355 -1.53389 -1.50253

C 1.34807 5.30102 -1.27987

H 2.03238 5.71639 -0.53779

H 1.73399 5.43265 -2.28907

H 0.36388 5.76071 -1.16858

[(L^2^)Fe^V^=O]^2+^ (^2^R_LS_)

Fe -1.22431 -0.72818 0.57327

N -2.03825 1.05251 0.31862

C -1.40055 1.85278 -0.55580

C -3.85115 2.61388 0.45915

H -4.81135 2.87756 0.88879

C -3.23307 1.41374 0.83524

H -3.63369 0.75790 1.59892

C -1.96037 3.06602 -0.96361

H -1.42375 3.69825 -1.66317

C -3.20697 3.44558 -0.45519

H -3.66000 4.38254 -0.76312

N 0.31081 0.03154 1.79403

N -0.08582 -0.11055 -1.07897

C -0.71481 -0.41177 -2.39872

H -1.67405 0.10038 -2.46929

H -0.05967 -0.07048 -3.20894

C -0.00725 1.38044 -0.92653

H 0.31768 1.83205 -1.87028

O 1.76056 3.71998 -1.04015

C 0.99063 1.83148 0.17871

C 0.51117 1.50184 1.60063

H 1.27495 1.84934 2.30337

H -0.40991 2.02290 1.84803

C 1.12977 3.36414 0.08186

C 2.34213 1.14695 -0.08764

H 2.64990 1.34152 -1.12108

O 3.26355 1.68601 0.83280

C -0.05781 -0.20055 3.22623

H -0.99662 0.30719 3.44201

N -0.26848 -2.41677 0.24889

C 0.74839 -2.33646 -0.64234

C -0.10135 -4.79395 0.26232

H -0.45525 -5.74396 0.64739

C -0.69382 -3.61062 0.69389

H -1.51695 -3.58365 1.39832

C 1.36634 -3.48001 -1.12827

H 2.18078 -3.39752 -1.83901

C 0.93669 -4.72784 -0.66740

H 1.41131 -5.63462 -1.02867

H -0.88536 -1.48335 -2.48615

C 1.18110 -0.91796 -0.96686

H 1.71145 -0.88820 -1.92687

O 4.37894 -0.47045 -0.73008

C 2.14860 -0.37179 0.12836

C 1.62803 -0.63289 1.55011

H 2.35516 -0.21968 2.25775

H 1.53610 -1.69727 1.75072

C 3.47454 -1.14571 -0.01050

H 4.16042 1.52618 0.49620

H -0.18454 -1.26908 3.39410

H 0.73479 0.19000 3.87380

O -2.13114 -1.18962 1.83664

O -2.59918 -1.54710 -0.68822

O 3.64088 -2.24841 0.45850

O 0.66945 4.12187 0.90378

C -3.81063 -1.29119 -0.85337

O -4.44439 -0.24447 -0.47857

C -4.71699 -2.27906 -1.57771

H -4.20458 -2.58275 -2.49598

H -4.83573 -3.14535 -0.91741

H -5.69672 -1.85788 -1.80917

C 5.65840 -1.14162 -0.93730

H 6.25019 -0.44908 -1.53304

H 6.12999 -1.33994 0.02699

H 5.49892 -2.08162 -1.46884

C 1.99780 5.14673 -1.21045

H 2.62801 5.51099 -0.39694

H 2.50212 5.24088 -2.17051

H 1.04853 5.68671 -1.20725

[(L^1^)Fe^IV^=O]^1+^ (^5^TS1_HS_)

Fe -0.71859 0.34350 -0.11338

N -0.99757 -1.77305 0.49099

C 0.05959 -2.26260 1.16684

C -2.33063 -3.60137 1.25503

H -3.29229 -4.10308 1.25643

C -2.17170 -2.42111 0.52869

H -2.98663 -1.99590 -0.04927

C -0.02213 -3.42982 1.92011

H 0.85580 -3.80035 2.43973

C -1.24289 -4.10924 1.96447

H -1.33807 -5.02285 2.54360

N 0.63623 -0.31955 -1.78811

N 1.02035 -0.02362 1.10078

C 0.72972 0.26647 2.53288

H -0.10541 -0.34677 2.86347

H 1.62007 0.06180 3.14139

C 1.34555 -1.48638 0.97726

H 2.05432 -1.75877 1.76749

O 3.28471 -3.60081 0.85450

C 2.03158 -1.86847 -0.36551

C 1.11890 -1.71397 -1.59040

H 1.68681 -2.01774 -2.48110

H 0.24395 -2.36060 -1.51438

C 2.47814 -3.34985 -0.28850

C 3.25731 -0.95526 -0.50874

H 3.84768 -1.00965 0.41397

O 4.03756 -1.40363 -1.60931

C -0.13263 -0.26355 -3.05741

H -1.00090 -0.91663 -2.97652

N 0.25895 2.31354 -0.06482

C 1.47213 2.29890 0.51997

C 0.23230 4.69825 0.02885

H -0.28967 5.62536 -0.18095

C -0.35609 3.47947 -0.31052

H -1.34204 3.40359 -0.74866

C 2.11322 3.47139 0.90571

H 3.08683 3.42404 1.38324

C 1.47820 4.69213 0.65380

H 1.95252 5.62418 0.94632

H 0.43638 1.30938 2.63735

C 2.09344 0.92322 0.64149

H 2.89167 0.94606 1.39292

O 4.91607 1.23798 0.04571

C 2.76265 0.49690 -0.69836

C 1.82196 0.56688 -1.91261

H 2.39427 0.25934 -2.79899

H 1.46411 1.58427 -2.07802

C 3.95891 1.41903 -0.99321

H 4.93063 -1.03972 -1.50415

H -0.48955 0.75368 -3.21609

H 0.50094 -0.57820 -3.89788

O -1.96042 0.62477 -1.27048

O -1.75567 0.75379 1.42625

H -3.07178 0.69983 -0.90561

C -4.40657 0.54606 -0.63945

H -4.79286 1.55061 -0.78751

H -4.38314 0.19140 0.38716

C -4.78078 -0.49228 -1.61610

O -4.60030 -1.69102 -1.45426

O -5.29811 0.01778 -2.75305

H -5.48676 -0.73676 -3.34294

C -2.58401 1.75672 1.66227

O -2.82444 2.67740 0.89289

C -3.23251 1.64595 3.03386

H -3.80511 0.71389 3.09797

H -2.46296 1.60500 3.81222

H -3.89147 2.49806 3.20674

H 4.37667 1.14411 -1.97194

H 1.59882 -3.99575 -0.20452

H 2.99897 -3.60724 -1.21567

H 4.21408 -3.49741 0.60236

H 5.65920 1.84251 -0.10304

H 3.62543 2.46338 -1.05954

[(L^1^)Fe^IV^=O]^1+^ (^3^TS1_IS_)

Fe 0.64392 0.24531 0.07551

N 0.90308 -1.68576 -0.37027

C -0.13890 -2.24183 -1.02859

C 2.22717 -3.61783 -0.82083

H 3.17724 -4.12654 -0.70028

C 2.06149 -2.35232 -0.26121

H 2.85804 -1.87636 0.29491

C -0.03550 -3.49089 -1.63016

H -0.89096 -3.90539 -2.15390

C 1.17060 -4.19023 -1.52639

H 1.27633 -5.16770 -1.98701

N -0.71688 -0.25038 1.74275

N -1.04425 -0.00344 -1.14727

C -0.75877 0.22669 -2.59130

H 0.02262 -0.45408 -2.92058

H -1.67565 0.07185 -3.17447

C -1.42403 -1.44567 -0.96960

H -2.09042 -1.75165 -1.78361

O -3.54564 -3.40525 -0.84918

C -2.18838 -1.73970 0.35122

C -1.30195 -1.61220 1.59678

H -1.91289 -1.83324 2.48328

H -0.48204 -2.33164 1.57278

C -2.74182 -3.18487 0.30034

C -3.34629 -0.73355 0.41395

H -3.88088 -0.75673 -0.54364

O -4.22478 -1.10596 1.46597

C 0.05747 -0.20974 3.01050

H 0.88094 -0.91986 2.94858

N 0.00692 2.12714 0.08667

C -1.19860 2.31810 -0.49709

C 0.32067 4.48464 0.24443

H 0.94543 5.31237 0.56026

C 0.75261 3.17645 0.46299

H 1.69465 2.93292 0.92920

C -1.66887 3.59392 -0.78065

H -2.62678 3.71722 -1.27543

C -0.89084 4.69587 -0.40719

H -1.23483 5.70351 -0.61996

H -0.39557 1.24384 -2.73037

C -2.00768 1.05332 -0.69137

H -2.77412 1.20775 -1.46000

O -4.87608 1.49365 -0.15708

C -2.76095 0.68305 0.61920

C -1.85412 0.69879 1.85834

H -2.46106 0.41713 2.72999

H -1.45049 1.69599 2.04172

C -3.90430 1.68182 0.86720

H -5.06875 -0.65235 1.30927

H 0.47990 0.78582 3.14126

H -0.59489 -0.45864 3.85856

O 1.92053 0.50751 1.20292

O 1.72109 0.47622 -1.48528

H 3.02040 0.51458 0.79665

C 4.35124 0.40464 0.48011

H 4.68308 1.43724 0.54419

H 4.30483 -0.01469 -0.52082

C 4.83445 -0.54266 1.49957

O 4.70207 -1.75711 1.43190

O 5.39236 0.06890 2.56570

H 5.64977 -0.63386 3.19247

C 2.47896 1.48728 -1.84508

O 2.72945 2.48648 -1.17846

C 3.07469 1.27712 -3.23232

H 2.27711 1.13262 -3.96944

H 3.68621 2.13641 -3.51154

H 3.68780 0.36906 -3.23979

H -4.33131 1.48180 1.85994

H -1.91150 -3.89575 0.24840

H -3.29418 -3.38019 1.22456

H -4.46425 -3.19764 -0.62165

H -5.57354 2.15944 -0.05612

H -3.51646 2.70902 0.87727

[(L^1^)Fe^IV^=O]^1+^ (^1^TS1_LS_)

Fe -0.86402 -0.86758 -0.01108

N -1.84245 0.72257 -0.68965

C -1.07027 1.61726 -1.34703

C -3.80277 1.87022 -1.43362

H -4.88476 1.92963 -1.42775

C -3.17524 0.81914 -0.76914

H -3.74196 0.03548 -0.28960

C -1.62342 2.67345 -2.05822

H -0.97895 3.37407 -2.57814

C -3.01383 2.81466 -2.08554

H -3.46868 3.64176 -2.62156

N 0.22887 0.17580 1.56126

N 0.59499 -0.13311 -1.30004

C 0.41596 -0.56154 -2.71652

H -0.56069 -0.24355 -3.07365

H 1.21174 -0.12826 -3.33659

C 0.41999 1.35936 -1.23932

H 0.93657 1.81540 -2.09502

O 2.05128 4.02000 -0.70579

C 1.02493 1.99515 0.04802

C 0.23800 1.64008 1.31995

H 0.71329 2.14910 2.16858

H -0.78999 1.99159 1.25407

C 0.94461 3.51857 -0.12163

C 2.48739 1.51227 0.16252

H 3.01062 1.68792 -0.78475

O 3.16681 2.13604 1.23496

C -0.44378 -0.07293 2.86487

H -1.41785 0.40602 2.85847

N 0.41029 -2.40519 0.13181

C 1.60564 -2.17593 -0.45822

C 1.01433 -4.68953 0.47000

H 0.74458 -5.66757 0.85308

C 0.11282 -3.63171 0.58674

H -0.87351 -3.73655 1.02029

C 2.54449 -3.18637 -0.62159

H 3.49375 -2.97093 -1.10121

C 2.24314 -4.46663 -0.14767

H 2.96000 -5.27395 -0.26102

H 0.44785 -1.64820 -2.76675

C 1.88011 -0.72282 -0.80382

H 2.63683 -0.65816 -1.59345

O 4.68882 -0.20991 -0.34767

C 2.45553 0.00186 0.44647

C 1.63997 -0.26992 1.71293

H 2.10849 0.26926 2.54381

H 1.65086 -1.32694 1.96911

C 3.88498 -0.50792 0.68865

H 3.44240 3.01688 0.93486

H -0.56499 -1.14628 3.00274

H 0.15890 0.34991 3.67953

O -2.03947 -1.60608 1.06033

O -1.69199 -1.74436 -1.51662

H -2.97550 -1.18789 1.39441

C -3.86586 -0.62206 2.48840

H -3.30310 -0.81298 3.39571

H -4.65677 -1.33590 2.26475

C -4.19849 0.76145 2.17677

O -5.11341 1.13234 1.45585

O -3.31829 1.65859 2.72192

H -3.63291 2.54278 2.45542

C -2.47214 -2.77411 -1.39126

O -2.76107 -3.34227 -0.33048

C -3.06643 -3.26082 -2.70630

H -2.28228 -3.33307 -3.46584

H -3.55625 -4.22610 -2.57222

H -3.80178 -2.52692 -3.05477

O -0.01166 4.18474 0.20128

O 4.23085 -1.11837 1.67207

C 6.07147 -0.60945 -0.19515

H 6.56715 -0.29590 -1.11312

H 6.50807 -0.11355 0.67470

H 6.13917 -1.69215 -0.06332

C 2.03816 5.45064 -0.94546

H 1.92906 5.98745 -0.00051

H 2.99489 5.67326 -1.41591

H 1.20785 5.71095 -1.60578

[(L^2^)Fe^IV^=O]^1+^ (^5^TS1_HS_)

Fe -0.98131 -0.84884 -0.04974

N -1.74894 1.16985 -0.69899

C -0.81004 1.88078 -1.35911

C -3.41450 2.71703 -1.44762

H -4.45664 3.01679 -1.44764

C -3.02546 1.57807 -0.74023

H -3.73089 0.98175 -0.18417

C -1.11931 3.01669 -2.09728

H -0.33455 3.55874 -2.61508

C -2.45063 3.44237 -2.14123

H -2.72345 4.32770 -2.70755

N 0.16906 0.17196 1.61934

N 0.64118 -0.08475 -1.24701

C 0.44795 -0.45501 -2.67951

H -0.48855 -0.03079 -3.03443

H 1.28720 -0.07266 -3.27516

C 0.61856 1.41125 -1.15951

H 1.25315 1.81545 -1.95671

O 2.07220 3.86346 -0.93349

C 1.17604 1.96120 0.18273

C 0.30214 1.63675 1.40085

H 0.76586 2.09833 2.27853

H -0.69465 2.05828 1.29283

C 1.26100 3.49413 0.07414

C 2.58824 1.38822 0.40355

H 3.20808 1.58597 -0.48458

O 3.11431 2.02811 1.53902

C -0.59164 -0.02628 2.88063

H -1.55801 0.46520 2.77827

N 0.44540 -2.52255 -0.03562

C 1.61219 -2.23876 -0.64165

C 0.87013 -4.85892 -0.25446

H 0.54541 -5.88008 -0.08621

C 0.07054 -3.79305 0.15375

H -0.89556 -3.92095 0.63106

C 2.45857 -3.24735 -1.09394

H 3.38873 -2.99572 -1.58830

C 2.07817 -4.57723 -0.89339

H 2.71729 -5.38353 -1.24059

H 0.37868 -1.53627 -2.77142

C 1.90371 -0.74943 -0.76735

H 2.68231 -0.59202 -1.52099

O 4.48282 -1.22094 -0.12502

C 2.43085 -0.15988 0.57160

C 1.51632 -0.43001 1.78417

H 1.99952 -0.00538 2.67021

H 1.38987 -1.49989 1.95003

C 3.83517 -0.67382 0.90841

H 3.83216 1.49265 1.92311

H -0.75285 -1.09024 3.04453

H -0.04029 0.41384 3.72228

O -1.99246 -1.65779 1.09132

O -1.74010 -1.65657 -1.58052

H -3.11872 -1.44331 1.26209

C -4.38902 -1.20092 1.75440

H -4.66326 -2.12842 2.25118

H -4.87027 -0.99468 0.80386

C -4.13679 -0.03580 2.61423

O -3.88783 1.09118 2.20701

O -4.11977 -0.34640 3.93077

H -3.94267 0.48282 4.41452

C -3.02602 -1.76492 -1.86910

O -3.92816 -1.20219 -1.26506

C -3.27345 -2.66715 -3.06596

H -2.92505 -3.68054 -2.83826

H -4.33719 -2.68721 -3.30683

H -2.70113 -2.31229 -3.92969

O 0.66231 4.25971 0.79030

O 4.34080 -0.49697 2.00155

C 2.25757 5.29069 -1.08626

H 2.69345 5.70823 -0.17599

H 2.93480 5.40634 -1.93168

H 1.29803 5.77559 -1.28222

C 5.85293 -1.62592 0.13280

H 6.21270 -2.03436 -0.81054

H 6.44592 -0.76091 0.43668

H 5.87544 -2.37916 0.92297

[(L^2^)Fe^IV^=O]^1+^ (^3^TS1_IS_)

Fe 0.91298 0.82781 -0.09706

N 1.71474 -0.91973 -0.67578

C 0.82966 -1.74946 -1.28488

C 3.47409 -2.46632 -1.16963

H 4.52698 -2.70637 -1.07911

C 3.01143 -1.26203 -0.63963

H 3.68220 -0.54291 -0.20669

C 1.22657 -2.94535 -1.86622

H 0.49008 -3.57681 -2.35231

C 2.57312 -3.31815 -1.79790

H 2.90400 -4.25593 -2.23335

N -0.22462 -0.16231 1.55957

N -0.67561 0.15698 -1.30186

C -0.50317 0.52164 -2.73757

H 0.42621 0.09280 -3.10673

H -1.35462 0.14093 -3.31681

C -0.62663 -1.33812 -1.19914

H -1.18454 -1.77063 -2.03694

O -2.18600 -3.75506 -1.06768

C -1.25814 -1.90227 0.10514

C -0.40642 -1.62115 1.34822

H -0.90595 -2.06593 2.21647

H 0.57267 -2.08942 1.26186

C -1.38969 -3.42780 -0.03397

C -2.65482 -1.28095 0.26099

H -3.21886 -1.43324 -0.66673

O -3.29304 -1.92183 1.34828

C 0.53174 0.02084 2.82696

H 1.51375 -0.43887 2.73881

N -0.24208 2.45401 -0.00067

C -1.47160 2.31206 -0.53732

C -0.54459 4.81492 0.11040

H -0.14491 5.78649 0.37945

C 0.22407 3.67060 0.31025

H 1.22921 3.68472 0.71589

C -2.28593 3.41084 -0.78151

H -3.27053 3.27330 -1.21505

C -1.81531 4.68299 -0.44838

H -2.43355 5.55721 -0.62677

H -0.42914 1.60264 -2.83162

C -1.88753 0.87319 -0.78183

H -2.68039 0.83537 -1.54103

O -4.83812 0.28576 0.22287

C -2.46519 0.23010 0.51685

C -1.56623 0.44541 1.74384

H -2.06003 -0.01690 2.60846

H -1.44911 1.50589 1.95666

C -3.80386 0.92395 0.80622

H -4.24552 -1.75194 1.27148

H 0.67065 1.08351 3.01451

H -0.01806 -0.44404 3.65641

O 1.99093 1.50621 1.06322

O 1.65769 1.67646 -1.60008

H 3.06014 1.12193 1.33900

C 4.30500 0.91474 1.89806

H 4.28971 1.69469 2.65714

H 4.92927 1.10580 1.03032

C 4.34681 -0.46746 2.40212

O 4.73469 -1.44109 1.77723

O 3.84149 -0.57778 3.66160

H 3.92684 -1.51790 3.91046

C 2.94111 1.87043 -1.83663

O 3.87128 1.47912 -1.14470

C 3.15650 2.67847 -3.10894

H 2.71222 3.67323 -2.99434

H 4.22405 2.77423 -3.31229

H 2.65766 2.19370 -3.95506

O -0.83338 -4.22500 0.68264

O -3.91240 1.94000 1.45268

C -2.41235 -5.17301 -1.25099

H -3.06628 -5.25264 -2.11856

H -1.46390 -5.68667 -1.42634

H -2.88871 -5.59143 -0.36153

C -6.13805 0.90296 0.40781

H -6.13562 1.91113 -0.01261

H -6.84021 0.26115 -0.12251

H -6.37884 0.95671 1.47180

[(L^2^)Fe^IV^=O]^1+^ (^1^TS1_LS_)

Fe -0.86402200 -0.86757800 -0.01107800

N -1.84244700 0.72257000 -0.68965000

C -1.07027300 1.61726200 -1.34703500

C -3.80276900 1.87021900 -1.43361700

H -4.88476500 1.92963200 -1.42775300

C -3.17524000 0.81914300 -0.76913700

H -3.74196100 0.03548200 -0.28959600

C -1.62341700 2.67345200 -2.05821900

H -0.97895100 3.37407200 -2.57813800

C -3.01383400 2.81466300 -2.08554300

H -3.46868300 3.64175700 -2.62156200

N 0.22887500 0.17580100 1.56125800

N 0.59499100 -0.13311300 -1.30004200

C 0.41595600 -0.56154200 -2.71652500

H -0.56068900 -0.24354700 -3.07364700

H 1.21174100 -0.12825500 -3.33659200

C 0.41998700 1.35936500 -1.23931700

H 0.93656700 1.81539800 -2.09502300

O 2.05127900 4.02000400 -0.70578600

C 1.02492700 1.99515200 0.04801700

C 0.23800100 1.64007700 1.31994800

H 0.71328600 2.14910200 2.16857600

H -0.78998500 1.99159300 1.25406600

C 0.94461300 3.51856900 -0.12163100

C 2.48738500 1.51227400 0.16251900

H 3.01061600 1.68791700 -0.78475400

O 3.16680500 2.13603900 1.23495500

C -0.44378200 -0.07292500 2.86487100

H -1.41784600 0.40601900 2.85847400

N 0.41029400 -2.40519000 0.13180600

C 1.60563800 -2.17592600 -0.45822100

C 1.01433200 -4.68952900 0.46999700

H 0.74458300 -5.66757100 0.85307600

C 0.11282100 -3.63171500 0.58673600

H -0.87350900 -3.73654900 1.02028700

C 2.54449200 -3.18637300 -0.62158600

H 3.49374600 -2.97093300 -1.10121000

C 2.24313600 -4.46663200 -0.14766900

H 2.95999600 -5.27394800 -0.26102200

H 0.44784900 -1.64819600 -2.76675400

C 1.88010700 -0.72282200 -0.80382400

H 2.63683000 -0.65815700 -1.59345100

O 4.68881600 -0.20991500 -0.34766600

C 2.45552900 0.00185600 0.44646800

C 1.63996800 -0.26992200 1.71293200

H 2.10849100 0.26925500 2.54380700

H 1.65086000 -1.32693600 1.96911500

C 3.88497900 -0.50792500 0.68865300

H 3.44239700 3.01687900 0.93486200

H -0.56499000 -1.14627800 3.00274300

H 0.15890300 0.34990700 3.67953200

O -2.03947500 -1.60608100 1.06032600

O -1.69199300 -1.74435900 -1.51661800

H -2.97549500 -1.18788800 1.39440800

C -3.86586100 -0.62206500 2.48840000

H -3.30310400 -0.81298200 3.39570800

H -4.65677500 -1.33589700 2.26474800

C -4.19848700 0.76145100 2.17676700

O -5.11340500 1.13233800 1.45585100

O -3.31828600 1.65858600 2.72192300

H -3.63291400 2.54278400 2.45541700

C -2.47214500 -2.77410500 -1.39125600

O -2.76107300 -3.34226700 -0.33047600

C -3.06643200 -3.26081800 -2.70630400

H -2.28228400 -3.33306700 -3.46584200

H -3.55624600 -4.22610000 -2.57222300

H -3.80177900 -2.52692500 -3.05477500

O -0.01165900 4.18473700 0.20128500

O 4.23085100 -1.11836900 1.67207300

C 6.07147400 -0.60945200 -0.19514600

H 6.56715000 -0.29590200 -1.11312000

H 6.50807300 -0.11355200 0.67470100

H 6.13917500 -1.69215400 -0.06331900

C 2.03816200 5.45064000 -0.94545700

H 1.92905600 5.98745400 -0.00051200

H 2.99489300 5.67325500 -1.41591200

H 1.20785500 5.71094600 -1.60577600

[(L^1^)Fe^V^=O]^2+^ (^4^TS1_HS_)

Fe 0.41204 0.68790 -0.06310

N 1.45274 -0.80241 -0.83318

C 0.67739 -1.73316 -1.44937

C 3.42039 -1.86881 -1.66612

H 4.50303 -1.88958 -1.71352

C 2.79067 -0.84789 -0.96239

H 3.34943 -0.05169 -0.49500

C 1.24137 -2.76586 -2.18581

H 0.59092 -3.49367 -2.65987

C 2.63517 -2.84119 -2.28658

H 3.09832 -3.64417 -2.85179

N -0.40095 -0.46947 1.55507

N -1.06574 -0.08574 -1.21042

C -1.02643 0.40378 -2.62543

H -0.06889 0.14836 -3.07473

H -1.84221 -0.06187 -3.18862

C -0.80858 -1.57046 -1.22493

H -1.36386 -2.01763 -2.05357

O -1.81112 -4.11988 -1.38557

C -1.25431 -2.30397 0.06748

C -0.37098 -1.93703 1.26620

H -0.73529 -2.47221 2.15161

H 0.66839 -2.22679 1.10443

C -1.15272 -3.82595 -0.16171

C -2.71669 -1.90468 0.36860

H -3.33915 -2.11589 -0.50655

O -3.16026 -2.63638 1.50184

C 0.40687 -0.22042 2.78451

H 1.42118 -0.58815 2.64078

N -0.89635 2.14865 0.18578

C -2.13795 1.85362 -0.26865

C -1.53174 4.41922 0.52956

H -1.26002 5.41825 0.85235

C -0.58618 3.39966 0.57002

H 0.43485 3.55068 0.90220

C -3.12397 2.82906 -0.34692

H -4.10952 2.56144 -0.71358

C -2.81566 4.13110 0.05997

H -3.56826 4.91173 0.00600

H -1.13139 1.48661 -2.63976

C -2.35165 0.39213 -0.59079

H -3.16242 0.27445 -1.31471

O -5.01535 -0.11947 -0.04946

C -2.76005 -0.38978 0.68585

C -1.81991 -0.10283 1.86150

H -2.14875 -0.69627 2.72294

H -1.84093 0.94770 2.15383

C -4.18671 0.02605 1.09446

H -4.00492 -3.06531 1.30116

H 0.43345 0.84915 2.99213

H -0.04108 -0.74855 3.63436

O 1.63978 1.52975 1.03011

O 1.07405 1.75624 -1.51800

H 2.70840 1.08560 1.50242

C 3.88355 0.93627 2.04881

H 3.74371 1.42440 3.01327

H 4.48844 1.49311 1.33509

C 4.19646 -0.51769 2.09526

O 4.76033 -1.12494 1.20554

O 3.74451 -1.09986 3.22708

H 4.04047 -2.03173 3.22324

C 2.16209 2.43857 -1.44203

O 2.86287 2.55541 -0.41074

C 2.60512 3.13653 -2.70942

H 1.79449 3.78446 -3.05763

H 3.50691 3.72401 -2.53547

H 2.79055 2.38447 -3.48311

H -4.50479 -0.62183 1.92005

H -0.09553 -4.12961 -0.19494

H -1.62477 -4.32663 0.69094

H -1.95338 -5.07687 -1.45331

H -5.94447 -0.01630 0.20943

H -4.18575 1.06337 1.46126

[(L^1^)Fe^V^=O]^2+^ (^2^TS1_LS_)

Fe 0.43545 0.67373 -0.06290

N 1.44025 -0.85067 -0.82747

C 0.65097 -1.75943 -1.45447

C 3.38903 -1.94723 -1.66741

H 4.47140 -1.98607 -1.71437

C 2.77598 -0.92080 -0.95532

H 3.35278 -0.14467 -0.47458

C 1.19625 -2.79622 -2.20087

H 0.53263 -3.50691 -2.68267

C 2.58790 -2.89835 -2.30004

H 3.03675 -3.70416 -2.87260

N -0.37253 -0.49058 1.54486

N -1.06954 -0.08498 -1.20812

C -1.03533 0.40259 -2.62409

H -0.08765 0.12808 -3.08318

H -1.86482 -0.05014 -3.17769

C -0.83224 -1.57441 -1.22601

H -1.39829 -2.01144 -2.05271

O -1.89519 -4.10188 -1.38428

C -1.28184 -2.30349 0.06714

C -0.37893 -1.95811 1.25684

H -0.74815 -2.48368 2.14582

H 0.65161 -2.27211 1.08641

C -1.21336 -3.82619 -0.16906

C -2.73223 -1.87696 0.38714

H -3.36964 -2.07645 -0.47991

O -3.17371 -2.60255 1.52525

C 0.44961 -0.26638 2.77032

H 1.45381 -0.65558 2.61456

N -0.87158 2.15743 0.18276

C -2.11523 1.87311 -0.27036

C -1.49514 4.43609 0.51253

H -1.21558 5.43414 0.83186

C -0.55818 3.40759 0.56287

H 0.46030 3.55436 0.90404

C -3.09546 2.85457 -0.35584

H -4.08319 2.58973 -0.71872

C -2.77987 4.15707 0.04223

H -3.52688 4.94259 -0.01679

H -1.12373 1.48691 -2.63919

C -2.34481 0.41114 -0.57957

H -3.16380 0.29424 -1.29435

O -5.00731 -0.06054 0.00965

C -2.74360 -0.36144 0.70492

C -1.77974 -0.08999 1.86551

H -2.11030 -0.67326 2.73324

H -1.77262 0.96172 2.15327

C -4.15564 0.07901 1.13747

H -4.03661 -3.00140 1.34067

H 0.49631 0.80072 2.98541

H -0.00249 -0.79308 3.61867

O 1.63427 1.48516 0.94525

O 1.10925 1.71221 -1.56696

H 2.64118 1.05204 1.49970

C 3.80119 0.92897 2.14881

H 3.55573 1.32122 3.13526

H 4.40705 1.58490 1.52557

C 4.21433 -0.49612 2.09278

O 4.85243 -0.98891 1.18197

O 3.76105 -1.20173 3.15284

H 4.12032 -2.10832 3.08321

C 2.14291 2.44342 -1.45608

O 2.81096 2.58947 -0.39017

C 2.61450 3.20836 -2.67221

H 3.57023 3.69957 -2.48613

H 2.69915 2.51449 -3.51363

H 1.85665 3.95916 -2.92074

H -4.46897 -0.55905 1.97254

H -0.16287 -4.15007 -0.21901

H -1.68289 -4.32099 0.68841

H -2.05504 -5.05587 -1.45464

H -5.92920 0.06502 0.28400

H -4.13168 1.11831 1.49755

[(L^2^)Fe^V^=O]^2+^ (^4^TS1_HS_)

Fe -0.79150 -0.87843 -0.04708

N -1.78032 0.72248 -0.68166

C -0.98718 1.63074 -1.30085

C -3.71472 1.97532 -1.30945

H -4.79543 2.06496 -1.30616

C -3.11619 0.87273 -0.71185

H -3.69653 0.07659 -0.26702

C -1.51804 2.75512 -1.91897

H -0.85743 3.46651 -2.40283

C -2.90340 2.93839 -1.91182

H -3.34144 3.81009 -2.38788

N 0.28034 0.07914 1.53164

N 0.63142 -0.16319 -1.31188

C 0.40352 -0.56903 -2.73576

H -0.57455 -0.22227 -3.06251

H 1.18673 -0.13251 -3.36536

C 0.49432 1.33582 -1.22258

H 1.01468 1.79110 -2.07136

O 1.82949 3.88596 -1.07353

C 1.09823 1.93657 0.07272

C 0.30414 1.56166 1.33154

H 0.78519 2.03075 2.19571

H -0.72030 1.92757 1.29689

C 1.06965 3.47344 -0.05082

C 2.55266 1.44608 0.18005

H 3.07946 1.67469 -0.75353

O 3.13701 2.11386 1.27493

C -0.39049 -0.20502 2.83750

H -1.35926 0.28755 2.85072

N 0.40261 -2.43939 0.01477

C 1.60943 -2.23929 -0.56778

C 0.85384 -4.76872 0.22316

H 0.52900 -5.74935 0.55307

C 0.02026 -3.66945 0.39940

H -0.96688 -3.73877 0.84254

C 2.47581 -3.29905 -0.79459

H 3.43865 -3.12449 -1.26049

C 2.09387 -4.58166 -0.39034

H 2.75975 -5.42337 -0.55243

H 0.42115 -1.65419 -2.81150

C 1.92620 -0.78335 -0.85458

H 2.66174 -0.70052 -1.66370

O 4.88434 0.12459 0.13568

C 2.51701 -0.08377 0.40678

C 1.69348 -0.38928 1.66173

H 2.15217 0.13929 2.50447

H 1.70017 -1.45196 1.89380

C 3.92584 -0.66584 0.63331

H 4.10248 2.06955 1.17942

H -0.49795 -1.28286 2.96063

H 0.22150 0.19796 3.65242

O -2.01073 -1.64914 1.10641

O -1.67022 -1.84004 -1.47345

H -2.93460 -1.07191 1.68245

C -3.92920 -0.64007 2.42265

H -3.55674 -0.93478 3.40506

H -4.77777 -1.22314 2.06760

C -4.00434 0.83816 2.23234

O -3.05455 1.59187 2.35819

O -5.22760 1.24066 1.85652

H -5.23054 2.21870 1.82875

C -2.82450 -2.38977 -1.33002

O -3.47944 -2.41104 -0.26187

C -3.41091 -3.05194 -2.55770

H -2.75944 -3.88322 -2.84821

H -4.41547 -3.42502 -2.35659

H -3.42843 -2.33400 -3.38304

O 0.41469 4.18608 0.67036

O 4.11499 -1.73259 1.17267

C 1.93615 5.32822 -1.23840

H 2.57677 5.47159 -2.10686

H 0.94630 5.76020 -1.40099

H 2.38198 5.76633 -0.34338

C 6.25244 -0.36190 0.28188

H 6.36076 -1.31686 -0.23576

H 6.87736 0.40431 -0.17322

H 6.48650 -0.48878 1.34052

[(L^2^)Fe^V^=O]^2+^ (^2^TS1_LS_)

Fe -0.75680 -0.86748 -0.09350

N -1.75060 0.70830 -0.77177

C -0.96595 1.62217 -1.39685

C -3.70110 1.92265 -1.42631

H -4.78156 2.00492 -1.40028

C -3.08769 0.83165 -0.81829

H -3.66387 0.04287 -0.35832

C -1.51229 2.71859 -2.04821

H -0.86136 3.43234 -2.54181

C -2.90209 2.88094 -2.04920

H -3.35067 3.73841 -2.54095

N 0.19369 0.18816 1.50564

N 0.70386 -0.12326 -1.29949

C 0.55364 -0.57015 -2.72253

H -0.41482 -0.25302 -3.10352

H 1.35677 -0.13014 -3.32362

C 0.52044 1.37234 -1.25993

H 1.06346 1.81815 -2.09954

O 1.78597 3.96561 -1.11170

C 1.04791 2.02456 0.04131

C 0.20397 1.66272 1.26844

H 0.63121 2.16661 2.13982

H -0.82212 2.00771 1.16067

C 0.97903 3.55704 -0.12816

C 2.50618 1.58583 0.26964

H 3.10293 1.80611 -0.62832

O 2.95326 2.31264 1.38149

C -0.54112 -0.05862 2.78304

H -1.53784 0.37246 2.72039

N 0.49895 -2.39716 0.01530

C 1.72964 -2.15107 -0.49444

C 1.02110 -4.71432 0.22909

H 0.71005 -5.70879 0.52981

C 0.14254 -3.64392 0.36735

H -0.86286 -3.75450 0.75754

C 2.64616 -3.17890 -0.67889

H 3.62040 -2.96015 -1.09714

C 2.28617 -4.47911 -0.31036

H 2.98629 -5.29729 -0.44808

H 0.60030 -1.65589 -2.77050

C 2.00081 -0.68250 -0.77410

H 2.76782 -0.57418 -1.54497

O 4.62302 -0.88261 -0.14998

C 2.49492 0.03636 0.50694

C 1.60646 -0.25844 1.72446

H 2.01717 0.27926 2.58317

H 1.59693 -1.31991 1.97134

C 3.94250 -0.34047 0.86060

H 3.78173 1.93756 1.73151

H -0.60927 -1.13107 2.96436

H -0.00636 0.42038 3.61093

O -1.92032 -1.67507 1.03269

O -1.56537 -1.85472 -1.55251

H -2.91597 -1.15516 1.59354

C -4.04353 -0.95539 2.22972

H -3.82614 -1.40399 3.19958

H -4.71466 -1.53148 1.59517

C -4.33751 0.50507 2.24044

O -5.02759 1.06370 1.41097

O -3.72317 1.14178 3.25902

H -4.00154 2.07928 3.24363

C -2.66577 -2.48603 -1.40083

O -3.30208 -2.55741 -0.31444

C -3.23143 -3.20791 -2.60231

H -3.18796 -2.54882 -3.47362

H -2.60453 -4.08373 -2.80427

H -4.25580 -3.53345 -2.41743

O 0.25444 4.26278 0.53208

O 4.41883 -0.06451 1.94324

C 1.86528 5.40625 -1.30989

H 2.24929 5.87734 -0.40315

H 2.55091 5.54349 -2.14414

H 0.87551 5.80628 -1.54012

C 6.03857 -1.14167 0.10278

H 6.41991 -1.56276 -0.82571

H 6.54408 -0.20598 0.34744

H 6.14114 -1.84400 0.93187

[(L^1^)Fe^IV^=O]^1+^ (^5^INT_HS_)

Fe 0.87570300 0.90055100 0.76999600

N 2.02380000 -0.87568600 0.41536100

C 1.56031600 -1.71902400 -0.52681600

C 4.13829400 -1.98667700 0.39562200

H 5.14984000 -2.05619100 0.77984600

C 3.28112400 -0.99784500 0.86530400

H 3.58318200 -0.27286900 1.61339600

C 2.36176100 -2.72665900 -1.05414000

H 1.95185000 -3.39305500 -1.80590300

C 3.67076900 -2.85915600 -0.58695600

H 4.31805600 -3.63274800 -0.98881400

N -0.75542200 -0.53197400 1.86011900

N -0.16983000 -0.07744800 -0.99629500

C 0.46621400 0.44618500 -2.23623700

H 1.53034000 0.21235800 -2.22703700

H -0.01429300 0.01019300 -3.12316400

C 0.10293000 -1.54171700 -0.91011100

H -0.06196700 -1.99399000 -1.89438300

O -0.73290600 -4.13610100 -1.48011000

H -0.77561500 -5.09909900 -1.57805000

C -0.81995200 -2.31075100 0.08273500

C -0.55158700 -1.96901000 1.55774600

H -1.23077300 -2.57263700 2.17842100

H 0.47113200 -2.22677700 1.83912000

C -0.59878400 -3.82469300 -0.09622800

C -2.27667100 -1.94652900 -0.26215900

H -2.45470400 -2.14247400 -1.32443900

O -3.15181300 -2.73454700 0.54055200

C -0.56369500 -0.32036000 3.31369200

H 0.46068800 -0.57891100 3.58169000

N -0.74229300 2.25175100 0.25762300

C -1.62767100 1.83327900 -0.66648000

C -1.60766800 4.47386400 0.11080700

H -1.56834000 5.50613400 0.44101500

C -0.72458400 3.53761600 0.64011700

H 0.02194600 3.79287400 1.38428200

C -2.53986500 2.71271100 -1.24609200

H -3.24275700 2.33991400 -1.98408500

C -2.52798200 4.05160100 -0.85098500

H -3.22561500 4.75757300 -1.29146800

H 0.36659000 1.53157400 -2.26030700

C -1.59766800 0.34853900 -0.97663700

H -2.03718700 0.18506400 -1.96790400

O -4.28536900 -0.28483500 -1.55200900

H -5.23455700 -0.13210500 -1.67515200

C -2.48333300 -0.44264300 0.02956900

C -2.14490700 -0.16167500 1.50356700

H -2.84527400 -0.73764300 2.12689100

H -2.28027300 0.89573100 1.74186100

C -3.96022400 -0.06651400 -0.18231100

H -3.89369700 -3.02170400 -0.01063100

H -0.70759500 0.73450400 3.54615400

H -1.27055900 -0.93471500 3.89070600

O 1.39320200 1.47401800 2.44033300

O 2.06769800 2.02530700 -0.22100600

C 3.18145300 1.90493400 -0.93190200

O 3.55331000 0.85833400 -1.44618100

C 3.95091900 3.20584600 -1.06005700

H 3.31336000 3.96734400 -1.52238200

H 4.22669600 3.57571100 -0.06615400

H 4.84823200 3.05745100 -1.66264200

H 2.09469800 2.14455300 2.39077400

H -4.56884900 -0.69527600 0.47994400

H -1.34831300 -4.34886600 0.50711000

H 0.39958600 -4.10028600 0.27446000

H -4.12599200 0.98224000 0.10437200

C 2.83433124 1.09669687 5.10798978

H 2.26376738 0.29349072 5.55174459

H 2.47227598 2.11473312 5.15684706

C 4.16347486 0.80397086 4.60209379

O 4.63899229 -0.32445692 4.51311785

O 4.79741674 1.91181140 4.16710556

H 5.54971437 1.61851709 3.61955542

[(L^1^)Fe^IV^=O]^1+^ (^3^INT_IS_)

Fe 0.58407400 0.91942100 0.79177100

N 2.22230900 -0.46136000 0.38945300

C 1.94581200 -1.32560700 -0.60307000

C 4.53687600 -1.02291200 0.24952900

H 5.54902200 -0.87104200 0.60783600

C 3.48422000 -0.30022000 0.80402100

H 3.62848600 0.43126700 1.59188400

C 2.94089000 -2.07544200 -1.21983600

H 2.67833600 -2.77110500 -2.00987800

C 4.25899800 -1.91792200 -0.78310200

H 5.05906000 -2.48538000 -1.24879400

N -0.55079800 -0.66088300 1.86506900

N -0.11016800 -0.06757900 -0.95534500

C 0.38803000 0.62908700 -2.17719300

H 1.47615800 0.62881000 -2.17181700

H 0.00268500 0.12969500 -3.07556200

C 0.47201100 -1.45236300 -0.93083200

H 0.35326000 -1.89694200 -1.92416100

O 0.22842100 -4.16421900 -1.49757800

H 0.43908900 -5.10358700 -1.60736000

C -0.23185600 -2.41193300 0.07498400

C -0.02265100 -2.01630300 1.54672700

H -0.54066800 -2.75312700 2.17584900

H 1.03440300 -2.02910800 1.81366400

C 0.33211000 -3.83220000 -0.11630600

C -1.74064200 -2.39786200 -0.23725300

H -1.89244200 -2.64029900 -1.29345400

O -2.39401400 -3.35604200 0.59103500

C -0.40398000 -0.42513800 3.32340500

H 0.65342400 -0.44776200 3.58390100

N -1.29247300 2.01058100 0.30150400

C -2.00720000 1.42574500 -0.67520400

C -2.57732300 4.00756000 0.06275100

H -2.76954600 5.02803100 0.37597900

C -1.55455600 3.27375300 0.65989100

H -0.91592700 3.68318400 1.43512600

C -3.04369900 2.09069300 -1.32517400

H -3.60537200 1.58248500 -2.10219100

C -3.33283200 3.40330200 -0.94440300

H -4.13253500 3.95052400 -1.43443800

H 0.05303000 1.66537300 -2.15502900

C -1.61132700 -0.00784500 -0.96053600

H -1.96860400 -0.29588200 -1.95511600

O -4.07635900 -1.24686200 -1.51322900

H -5.03637400 -1.29525100 -1.63745100

C -2.28008200 -0.97933200 0.05248900

C -1.99811900 -0.62532800 1.52240400

H -2.52526700 -1.35486100 2.15219900

H -2.37287000 0.36868900 1.77067900

C -3.80516400 -0.94692800 -0.14769800

H -3.04442700 -3.83089400 0.05430700

H -0.78494200 0.56643700 3.56477800

H -0.95154400 -1.19179700 3.88773900

O 1.10067300 1.62777100 2.34406700

O 1.41545200 2.47826100 -0.01365300

C 2.48219800 2.63269500 -0.77489500

O 3.16091200 1.72749800 -1.24848500

C 2.80089700 4.10003800 -1.02443700

H 1.92892400 4.60291100 -1.45631600

H 3.02487600 4.59908800 -0.07520000

H 3.65460500 4.18922100 -1.69799700

H 1.58947900 2.43752200 2.10417700

H -4.25292900 -1.69050900 0.52411300

H -0.25471400 -4.51702600 0.50651200

H 1.37856100 -3.86473800 0.22048600

H -4.19958800 0.04133300 0.12987300

C 2.83433124 1.09669687 5.10798978

H 2.26376738 0.29349072 5.55174459

H 2.47227598 2.11473312 5.15684706

C 4.16347486 0.80397086 4.60209379

O 4.63899229 -0.32445692 4.51311785

O 4.79741674 1.91181140 4.16710556

H 5.54971437 1.61851709 3.61955542

[(L^1^)Fe^IV^=O]^1+^ (^1^INT_LS_)

Fe 0.74488900 0.72520600 0.64208500

N 1.93596200 -0.82480000 0.41802100

C 1.52405200 -1.71015500 -0.51386100

C 4.03283000 -1.94094100 0.58011200

H 5.01570200 -1.99956200 1.03406300

C 3.15808100 -0.92691000 0.95582800

H 3.40391700 -0.16868000 1.68927100

C 2.35246100 -2.73393000 -0.95348800

H 1.98988600 -3.42638000 -1.70588100

C 3.62987900 -2.84737000 -0.39932800

H 4.30032400 -3.63453900 -0.73015600

N -0.66643000 -0.46949600 1.79079800

N -0.16304400 -0.06239700 -1.03219900

C 0.45088300 0.43944600 -2.29634900

H 1.50398800 0.16367300 -2.31793500

H -0.08328900 0.01800000 -3.15742700

C 0.08225100 -1.53999600 -0.94197000

H -0.07773800 -1.99256600 -1.92495700

O -0.85228400 -4.10165600 -1.50062200

H -0.91874300 -5.06294600 -1.60190200

C -0.85594200 -2.27800400 0.05798400

C -0.54331800 -1.92921300 1.52144600

H -1.24607700 -2.47273500 2.16748900

H 0.46585500 -2.23769000 1.79618600

C -0.68292500 -3.79754600 -0.12005200

C -2.30677700 -1.86808900 -0.26074500

H -2.50863600 -2.05566700 -1.32015200

O -3.18782700 -2.63227200 0.55642300

C -0.40884100 -0.24161500 3.23640700

H 0.61806700 -0.52350100 3.46406700

N -0.57397800 2.18068100 0.30188700

C -1.51769900 1.88173800 -0.61543800

C -1.27461600 4.45858700 0.30686000

H -1.14867900 5.46342800 0.69467700

C -0.44219200 3.43544500 0.75252800

H 0.34582200 3.58849200 1.47871200

C -2.37418300 2.85562600 -1.11761000

H -3.11795000 2.58160500 -1.85861800

C -2.25049600 4.16506500 -0.64659600

H -2.90423500 4.94567800 -1.02345400

H 0.38970000 1.52705500 -2.31330200

C -1.58377400 0.41455100 -0.97772600

H -2.04599900 0.28978200 -1.96248400

O -4.30437700 -0.18875000 -1.49000300

H -5.24456400 0.01855700 -1.60197800

C -2.46678100 -0.35941800 0.03888000

C -2.07413000 -0.08536800 1.49946800

H -2.74367600 -0.66491100 2.14953100

H -2.19459100 0.96939100 1.75239000

C -3.93595700 0.05868100 -0.13737200

H -3.96546200 -2.86543400 0.02898500

H -0.51959700 0.81865000 3.45739300

H -1.10940800 -0.83310500 3.84109700

O 1.47442600 1.35965700 2.14344100

O 1.94699800 1.97566600 -0.25669300

C 3.03947100 1.77357000 -0.95737200

O 3.41130400 0.69924500 -1.42726700

C 3.83859200 3.05459200 -1.16318700

H 3.19802000 3.83455200 -1.58795600

H 4.20204100 3.42464400 -0.19754700

H 4.68612600 2.86671900 -1.82420700

H 2.08760600 2.04705200 1.82203500

H -4.54419000 -0.53110700 0.56063400

H -1.43574700 -4.29856500 0.49877000

H 0.31407400 -4.10061300 0.23210900

H -4.05834500 1.11984700 0.12402400

C 2.83433124 1.09669687 5.10798978

H 2.26376738 0.29349072 5.55174459

H 2.47227598 2.11473312 5.15684706

C 4.16347486 0.80397086 4.60209379

O 4.63899229 -0.32445692 4.51311785

O 4.79741674 1.91181140 4.16710556

H 5.54971437 1.61851709 3.61955542

[(L^2^)Fe^IV^=O]^1+^ (^5^INT_HS_)

Fe -1.35088100 -0.99912200 0.71103600

N -2.19488800 0.94444800 0.39283500

C -1.57971400 1.74271500 -0.49958300

C -4.09966300 2.38528600 0.39138300

H -5.09375900 2.60380800 0.76496600

C -3.42580300 1.24919700 0.82706100

H -3.85744800 0.55161300 1.53661300

C -2.19409800 2.88734500 -0.99300100

H -1.67686200 3.51021200 -1.71540500

C -3.47484400 3.21202100 -0.54076800

H -3.97719800 4.09863800 -0.91487200

N 0.41113500 0.11410000 1.91326600

N -0.08499500 -0.11901000 -0.99662900

C -0.73302300 -0.49132400 -2.28348500

H -1.74965000 -0.09959800 -2.30380200

H -0.15222400 -0.09866300 -3.13039200

C -0.15087100 1.36176200 -0.84463800

H 0.12712200 1.83405300 -1.79523000

O 1.27769000 3.85797500 -1.08354700

C 0.82164800 1.92781900 0.23659600

C 0.43408800 1.57767200 1.68220800

H 1.16398300 2.05315400 2.34953200

H -0.54342600 1.98721000 1.92992200

C 0.82339900 3.46108100 0.11921900

C 2.22395500 1.38454400 -0.07560000

H 2.46334100 1.59262000 -1.12478500

O 3.14275900 2.03007000 0.78496000

C 0.12040400 -0.13153600 3.34699300

H -0.86626700 0.26526700 3.58472900

N 0.07411700 -2.54530900 0.21436200

C 1.03964300 -2.24880100 -0.67571300

C 0.59036200 -4.87020800 0.02983800

H 0.38716300 -5.89054200 0.33581500

C -0.15092900 -3.82062300 0.56401200

H -0.94623200 -3.97279000 1.28543300

C 1.81090200 -3.24550200 -1.26433200

H 2.57987000 -2.98462200 -1.98355800

C 1.58310800 -4.57491500 -0.90527900

H 2.17428400 -5.36916300 -1.35040800

H -0.79646000 -1.57736100 -2.35123100

C 1.25454800 -0.76543600 -0.92967100

H 1.76817900 -0.63715000 -1.89459700

O 4.34849700 -0.05347300 -0.86641500

C 2.18752200 -0.14131000 0.15983100

C 1.74846400 -0.43944600 1.60479100

H 2.49616000 0.00636000 2.27587900

H 1.73916000 -1.51253100 1.78855800

C 3.57637400 -0.76926400 -0.02374000

H 4.02831400 1.91776200 0.40428100

H 0.09993500 -1.20547500 3.53048000

H 0.88073000 0.34647500 3.98082300

O -2.06103500 -1.55513000 2.31243900

O -2.63228800 -1.87004200 -0.41414700

O 3.92635300 -1.80219400 0.49945200

O 0.44040400 4.21021600 0.98633100

C -3.69261300 -1.53701300 -1.13933000

O -3.86176400 -0.42746700 -1.62832000

C -4.67638700 -2.67690800 -1.31942800

H -4.17206500 -3.52952700 -1.78701800

H -5.03976900 -3.01356300 -0.34195200

H -5.51709700 -2.35794000 -1.93726300

H -2.85161000 -2.10792100 2.19584200

C 5.66396000 -0.60414600 -1.12875000

H 6.13678600 0.09351400 -1.81869400

H 6.22988900 -0.68239400 -0.19779300

H 5.57143700 -1.59557300 -1.57802300

C 1.35173000 5.28982600 -1.27599800

H 2.02806900 5.73024700 -0.53989900

H 1.73234700 5.42755400 -2.28742800

H 0.36072000 5.73722200 -1.16601900

C -3.89084986 -2.26545180 4.68845354

H -3.00578981 -1.98595615 5.24176398

H -3.98181420 -3.25887565 4.27047819

C -5.01300585 -1.34429166 4.66672871

O -4.98212117 -0.20246913 5.11691783

O -6.08137579 -1.85935136 4.02508481

H -6.68368617 -1.11839526 3.82510809

[(L^2^)Fe^IV^=O]^1+^ (^3^INT_IS_)

Fe -1.05381100 -1.08474300 0.73871900

N -2.36415000 0.62114600 0.36709000

C -1.89237700 1.45726400 -0.57332800

C -4.50147400 1.67328000 0.24136700

H -5.52800200 1.72632400 0.58656800

C -3.63823700 0.71287700 0.76282600

H -3.94985700 -0.00957800 1.50946100

C -2.69172500 2.43079500 -1.15857100

H -2.28335700 3.08820200 -1.91923300

C -4.02101800 2.53854800 -0.73985600

H -4.67207000 3.28761800 -1.17980200

N 0.32757700 0.17603500 1.92008100

N -0.10921600 -0.16941300 -0.95209900

C -0.69496500 -0.69582300 -2.21980900

H -1.76127800 -0.47865400 -2.23704900

H -0.18983900 -0.24296100 -3.08330400

C -0.41320800 1.29740200 -0.86356700

H -0.16991000 1.76536500 -1.82445100

O 0.51195100 4.00168800 -1.10601600

C 0.42494400 2.03222900 0.22774700

C 0.08522800 1.62266500 1.67057300

H 0.71128300 2.21960300 2.34347800

H -0.95269500 1.84578700 1.90552600

C 0.14800200 3.53977100 0.10373800

C 1.90844600 1.75380400 -0.05808500

H 2.12480900 2.00577100 -1.10230300

O 2.68183000 2.54975100 0.81907100

C 0.09010100 -0.09786200 3.36135600

H -0.94984600 0.12140200 3.59871200

N 0.59169800 -2.49169900 0.24707400

C 1.43417300 -2.03291000 -0.69333200

C 1.45636400 -4.69688500 -0.03930400

H 1.43658400 -5.74340000 0.24469600

C 0.58809400 -3.79141800 0.56673900

H -0.13635000 -4.08730100 1.31773200

C 2.32141500 -2.87289400 -1.35684400

H 2.98789600 -2.47778500 -2.11656100

C 2.33368800 -4.22779700 -1.01803100

H 3.01667200 -4.90857800 -1.51661900

H -0.57326300 -1.77791600 -2.24198700

C 1.34633400 -0.53547800 -0.91841100

H 1.79542000 -0.27754300 -1.88777500

O 4.25612200 0.70399300 -0.84378000

C 2.14165300 0.24567000 0.17565500

C 1.75030100 -0.12647000 1.61721700

H 2.38991600 0.45612300 2.29250300

H 1.93408600 -1.18107000 1.80891900

C 3.62183700 -0.12714300 0.00681800

H 3.57786400 2.60725400 0.45073500

H 0.26454100 -1.15518000 3.55634700

H 0.75810200 0.51901400 3.97620200

O -1.76208300 -1.74573100 2.23338000

O -2.14618000 -2.39738700 -0.17473100

O 4.14637400 -1.07233900 0.54857100

O -0.35251400 4.21273600 0.97286400

C -3.19862200 -2.28741900 -0.96463400

O -3.64937500 -1.23852800 -1.41289300

C -3.82106900 -3.63914600 -1.28176800

H -3.06280300 -4.31354100 -1.69358900

H -4.19731700 -4.09917400 -0.36130100

H -4.63972100 -3.51728900 -1.99265700

H -2.39389100 -2.42857700 1.93843000

C 5.65060500 0.39411300 -1.09602700

H 5.99339000 1.15510100 -1.79575800

H 6.21724900 0.43240800 -0.16304000

H 5.73836800 -0.60450700 -1.52990900

C 0.31763300 5.42120900 -1.31089700

H 0.90482300 5.98642300 -0.58368000

H 0.65992700 5.61726400 -2.32635100

H -0.73877400 5.67647800 -1.19684800

C -3.89084986 -2.26545180 4.68845354

H -3.00578981 -1.98595615 5.24176398

H -3.98181420 -3.25887565 4.27047819

C -5.01300585 -1.34429166 4.66672871

O -4.98212117 -0.20246913 5.11691783

O -6.08137579 -1.85935136 4.02508481

H -6.68368617 -1.11839526 3.82510809

[(L^2^)Fe^IV^=O]^1+^ (^1^INT_LS_)

Fe -1.21292000 -0.80202300 0.58919700

N -2.10752200 0.94028200 0.39425800

C -1.51400200 1.77710500 -0.48200500

C -3.96397300 2.42008800 0.57322100

H -4.93005900 2.64104600 1.01285700

C -3.30418300 1.24292100 0.91216100

H -3.70665000 0.51142700 1.60217600

C -2.12249600 2.95731600 -0.88254400

H -1.62598800 3.60787900 -1.59468400

C -3.37150700 3.28216800 -0.34706300

H -3.87173200 4.19786700 -0.64598500

N 0.32511600 0.08175200 1.83992500

N -0.10768400 -0.12069100 -1.02309500

C -0.74527000 -0.46397200 -2.32850100

H -1.73549100 -0.01238600 -2.37068400

H -0.11342800 -0.10815200 -3.15278400

C -0.10801600 1.37162100 -0.87379200

H 0.17267800 1.83046400 -1.82789700

O 1.44939100 3.78668300 -1.11278100

C 0.89156000 1.88585900 0.20542400

C 0.45901500 1.55016500 1.64046300

H 1.21338100 1.95055000 2.32708000

H -0.48632500 2.02835300 1.88571000

C 0.97846100 3.41640100 0.09089100

C 2.26681000 1.26654600 -0.08684500

H 2.52980000 1.45574600 -1.13405800

O 3.20521800 1.86774300 0.78390600

C -0.02916600 -0.15773600 3.26452700

H -1.00137300 0.28776800 3.46864200

N -0.14717300 -2.44591300 0.24180500

C 0.86947700 -2.29100800 -0.63169100

C 0.15535500 -4.80885600 0.19962300

H -0.15163400 -5.78637300 0.55467500

C -0.50650500 -3.67001800 0.64998600

H -1.33436300 -3.70392700 1.34641200

C 1.56100800 -3.38317900 -1.13932600

H 2.37147100 -3.23429200 -1.84420900

C 1.19870700 -4.66319300 -0.71432500

H 1.72574300 -5.53331000 -1.09283700

H -0.86842200 -1.54457600 -2.38827800

C 1.20314000 -0.84346000 -0.93398400

H 1.72352800 -0.76579700 -1.89803300

O 4.32896900 -0.28629000 -0.83054500

C 2.15084200 -0.25417000 0.15708300

C 1.65842800 -0.52462300 1.58928700

H 2.39044500 -0.09283400 2.28375500

H 1.60697200 -1.59353500 1.78531700

C 3.50830500 -0.95459600 0.00355500

H 4.08959700 1.71075900 0.41634900

H -0.10669600 -1.22960300 3.43833300

H 0.73508700 0.28161700 3.91873500

O -2.11180700 -1.35284000 2.02649700

O -2.56898900 -1.78588200 -0.41513100

O 3.79323000 -1.99961500 0.54161000

O 0.64244800 4.18223600 0.96258500

C -3.57981600 -1.36124400 -1.13879800

O -3.72474100 -0.22218100 -1.58129200

C -4.59929700 -2.45974400 -1.41121400

H -4.10221500 -3.34257700 -1.82636600

H -5.07223100 -2.76689200 -0.47118800

H -5.36337700 -2.10070700 -2.10243500

H -2.81612000 -1.91386400 1.65028600

C 5.61964100 -0.90529900 -1.06770200

H 6.13835900 -0.23688300 -1.75347400

H 6.16479700 -1.00578500 -0.12662400

H 5.48338300 -1.89336600 -1.51298200

C 1.60498400 5.21270400 -1.30670700

H 2.31093400 5.61257600 -0.57535400

H 1.98577500 5.32764000 -2.32080200

H 0.64215100 5.71613500 -1.19014500

C -3.89084986 -2.26545180 4.68845354

H -3.00578981 -1.98595615 5.24176398

H -3.98181420 -3.25887565 4.27047819

C -5.01300585 -1.34429166 4.66672871

O -4.98212117 -0.20246913 5.11691783

O -6.08137579 -1.85935136 4.02508481

H -6.68368617 -1.11839526 3.82510809

[(L^1^)Fe^V^=O]^2+^ (^6^INT1_HS_)

Fe 0.61394900 0.97905500 0.71398400

N 2.17866300 -0.45287100 0.38422800

C 1.91038600 -1.35087000 -0.58661500

C 4.47903800 -1.08794200 0.35676800

H 5.47963700 -0.95905400 0.75435000

C 3.43122700 -0.31747500 0.84994200

H 3.57420500 0.42676700 1.62561300

C 2.90660200 -2.14470200 -1.13960600

H 2.65367900 -2.86268300 -1.91262300

C 4.21374100 -2.00515500 -0.66138000

H 5.01412100 -2.60772200 -1.07949200

N -0.57566100 -0.64034600 1.85418200

N -0.13073900 -0.08392300 -0.97462200

C 0.37786700 0.59544200 -2.21387800

H 1.46415200 0.55222200 -2.23072900

H -0.03605300 0.08263700 -3.08892200

C 0.44237900 -1.47577200 -0.92546100

H 0.31956000 -1.92163800 -1.91723900

O 0.15942900 -4.15705700 -1.48386700

H 0.35167000 -5.09840500 -1.61826200

C -0.27827700 -2.41270500 0.08254900

C -0.05391200 -2.00191500 1.54420200

H -0.59003800 -2.71634300 2.18217800

H 0.99951700 -2.02782200 1.82242600

C 0.26212700 -3.84540400 -0.10212900

C -1.79659000 -2.38004500 -0.21353100

H -1.97371500 -2.64477100 -1.26029200

O -2.46596700 -3.27509600 0.66184000

C -0.40237900 -0.36784600 3.30584700

H 0.65502500 -0.41030900 3.56287900

N -1.22847500 2.03523100 0.26239100

C -1.99665300 1.44730800 -0.67755100

C -2.54828200 4.01918500 0.11966200

H -2.73744100 5.03186200 0.45885400

C -1.48740100 3.29407500 0.65425500

H -0.82407300 3.70660000 1.40633400

C -3.07074600 2.10935000 -1.26217900

H -3.67556800 1.59702400 -2.00295400

C -3.34855700 3.41763000 -0.85517500

H -4.17868800 3.96132300 -1.29559100

H 0.06137900 1.63663300 -2.21133600

C -1.63365500 0.00844800 -0.96328600

H -1.99076600 -0.27882400 -1.95692400

O -4.07860200 -1.06282700 -1.55644500

H -5.02095800 -1.23887700 -1.70461200

C -2.31550200 -0.94866200 0.05086900

C -2.02458100 -0.57045200 1.50963400

H -2.55574900 -1.28022300 2.15707800

H -2.37732500 0.43248100 1.75146900

C -3.84249400 -0.89201500 -0.16582300

H -2.85499200 -4.00135900 0.15283800

H -0.78332400 0.62495900 3.54066600

H -0.95388300 -1.12217500 3.88003800

O 1.05800600 1.62447700 2.30493800

O 1.49232400 2.31036000 -0.17280300

C 2.66306300 2.52648100 -0.84933500

O 3.30708800 1.62406900 -1.33027200

C 2.99775400 3.99507400 -0.93012700

H 2.16846800 4.53980000 -1.39302600

H 3.13727400 4.40171700 0.07749500

H 3.90855600 4.12922500 -1.51524400

H 1.58168100 2.44498500 2.22728900

H -4.29729900 -1.69119100 0.43000600

H -0.34207700 -4.51819900 0.51816500

H -4.23836100 0.07000600 0.19178000

H 1.30303400 -3.90431900 0.24792500

C 2.83433124 1.09669687 5.10798978

H 2.26376738 0.29349072 5.55174459

H 2.47227598 2.11473312 5.15684706

C 4.16347486 0.80397086 4.60209379

O 4.63899229 -0.32445692 4.51311785

O 4.79741674 1.91181140 4.16710556

H 5.54971437 1.61851709 3.61955542

[(L^1^)Fe^V^=O]^2+^ (^4^INT1_IS_)

Fe 0.64794000 0.85496100 0.61181200

N 2.05071300 -0.51004500 0.40307200

C 1.77359800 -1.45859400 -0.52243500

C 4.27926800 -1.32100100 0.60820700

H 5.25204700 -1.24039700 1.08034900

C 3.26933900 -0.43384600 0.96200300

H 3.41293100 0.35232400 1.69236200

C 2.74566000 -2.35760700 -0.93742900

H 2.49287300 -3.10495200 -1.68220300

C 4.02068200 -2.28241200 -0.36916200

H 4.79999200 -2.96927100 -0.68410600

N -0.59402600 -0.55567300 1.78694500

N -0.14221100 -0.08081400 -1.02440600

C 0.37841600 0.50593200 -2.30603500

H 1.45581300 0.36781500 -2.35545000

H -0.10979400 -0.00212200 -3.14407300

C 0.32557600 -1.51327300 -0.93996100

H 0.22120400 -1.97000800 -1.92750000

O -0.19502100 -4.16142500 -1.50082100

H -0.09229900 -5.11707700 -1.63267300

C -0.48844300 -2.38089900 0.05881900

C -0.22947300 -1.98052100 1.51686600

H -0.84324600 -2.61751900 2.16505400

H 0.81293500 -2.12372100 1.80183800

C -0.08163400 -3.85749000 -0.11876900

C -1.99646500 -2.20692100 -0.24829300

H -2.18755700 -2.44111400 -1.30029000

O -2.75004400 -3.04780700 0.61069700

C -0.38036800 -0.28425200 3.23894200

H 0.67129100 -0.42084800 3.48545900

N -0.89745700 2.06791100 0.29423700

C -1.79830600 1.61735100 -0.60924300

C -1.97378400 4.19097500 0.34830800

H -2.01428300 5.19734600 0.75046200

C -0.96663500 3.32566600 0.76184400

H -0.20423400 3.61703200 1.47236700

C -2.82148100 2.43487400 -1.07350400

H -3.53102700 2.03754500 -1.79183400

C -2.90945400 3.74217700 -0.58705800

H -3.69713500 4.40280700 -0.93587300

H 0.15266900 1.57053900 -2.33259600

C -1.63108200 0.16444800 -0.97265500

H -2.04635000 -0.03599700 -1.96340800

O -4.16580600 -0.65666800 -1.56379600

H -5.11955200 -0.75851200 -1.70859400

C -2.38999100 -0.73871100 0.03344500

C -2.05041300 -0.39348200 1.48891500

H -2.61102700 -1.06961300 2.14587900

H -2.33528500 0.62786100 1.74358700

C -3.90694900 -0.54322700 -0.17184500

H -3.18369800 -3.74140100 0.09216000

H -0.66465500 0.74228800 3.46414800

H -0.99234400 -0.97756000 3.82701900

O 1.21627200 1.52558300 2.12875600

O 1.58101900 2.18989000 -0.29867400

C 2.75580100 2.17761400 -0.97408900

O 3.30505400 1.18189700 -1.39659900

C 3.26981800 3.59041600 -1.16848800

H 2.51743400 4.19724000 -1.68203600

H 3.46240900 4.05922200 -0.19751300

H 4.18893400 3.55894200 -1.75510600

H 1.78125500 2.29869100 1.93549000

H -4.42649200 -1.31267100 0.40973200

H -0.75276300 -4.46885200 0.49619300

H 0.94440300 -4.01267300 0.24582400

H -4.21387400 0.44096800 0.21248100

C 2.83433124 1.09669687 5.10798978

H 2.26376738 0.29349072 5.55174459

H 2.47227598 2.11473312 5.15684706

C 4.16347486 0.80397086 4.60209379

O 4.63899229 -0.32445692 4.51311785

O 4.79741674 1.91181140 4.16710556

H 5.54971437 1.61851709 3.61955542

[(L^1^)Fe^V^=O]^2+^ (^2^INT1_LS_)

Fe 0.85228100 0.54766800 0.70431000

N 1.75491600 -1.11749800 0.29934900

C 1.14815700 -1.96587100 -0.56515600

C 3.63658200 -2.57252700 0.44251400

H 4.61607000 -2.77707000 0.86029300

C 2.98589300 -1.39476500 0.77936500

H 3.42424500 -0.66021700 1.44022000

C 1.76239300 -3.14270000 -0.96579400

H 1.24613600 -3.79489500 -1.66218700

C 3.02664100 -3.45121100 -0.45494300

H 3.53194600 -4.36206700 -0.76074600

N -0.77421300 -0.35904900 1.80095800

N -0.17845300 -0.03889500 -0.99067700

C 0.54930800 0.37103400 -2.23820400

H 1.52546600 -0.10630900 -2.26695200

H -0.04759200 0.08223800 -3.10984200

C -0.23730900 -1.53755100 -0.95364900

H -0.49979800 -1.91085400 -1.94735400

O -1.55027300 -3.87567600 -1.52846200

H -1.79794400 -4.80356000 -1.66593100

C -1.28231700 -2.10190000 0.04792500

C -0.89806600 -1.82129100 1.50551000

H -1.67857200 -2.23793900 2.15312800

H 0.04576200 -2.29765700 1.77588100

C -1.38364600 -3.62893100 -0.14022600

C -2.64916600 -1.43568200 -0.24592600

H -2.91565100 -1.59024800 -1.29647600

O -3.63100200 -1.98201300 0.61971200

C -0.53547600 -0.21786600 3.27059800

H 0.40706100 -0.69513300 3.53594500

N -0.09161600 2.20568300 0.18818800

C -1.16267000 2.08819000 -0.62821800

C -0.31504800 4.57703100 0.16783500

H 0.04930700 5.54175300 0.50384700

C 0.34946300 3.42352800 0.56425800

H 1.23679000 3.45223800 1.17862200

C -1.86215500 3.20418300 -1.07074900

H -2.71424400 3.06245100 -1.72726800

C -1.43473100 4.46895500 -0.66214800

H -1.95798800 5.35902600 -0.99771500

H 0.69682600 1.44989800 -2.23421600

C -1.50278000 0.66683300 -0.96929700

H -1.95367000 0.60607700 -1.96358700

O -4.16477300 0.77927200 -1.55498300

H -5.09742400 1.00503300 -1.69743600

C -2.52328000 0.07924100 0.03777800

C -2.08550200 0.29100400 1.49182400

H -2.84939800 -0.14008500 2.15012400

H -1.99709100 1.35144800 1.73616900

C -3.88454900 0.77740800 -0.16239700

H -4.30338400 -2.44843500 0.10180600

H -0.48148800 0.83704300 3.53455700

H -1.35659500 -0.69394100 3.81767400

O 1.52430500 1.03687300 2.20263800

O 2.38213100 1.54243400 0.00214000

C 3.36427000 1.24205800 -0.84035600

O 3.48012300 0.17652200 -1.43224300

C 4.35172900 2.38249900 -1.00160000

H 3.83453500 3.26582700 -1.39063300

H 4.77885400 2.65092200 -0.02967500

H 5.14632500 2.08676900 -1.68755000

H 2.33865900 1.54744500 1.98236400

H -4.63507600 0.22114100 0.41020000

H -0.47746700 -4.11691600 0.24860700

H -2.23587100 -3.98606600 0.44968600

H -3.84303000 1.80170800 0.23757600

C 2.83433124 1.09669687 5.10798978

H 2.26376738 0.29349072 5.55174459

H 2.47227598 2.11473312 5.15684706

C 4.16347486 0.80397086 4.60209379

O 4.63899229 -0.32445692 4.51311785

O 4.79741674 1.91181140 4.16710556

H 5.54971437 1.61851709 3.61955542

[(L^2^)Fe^V^=O]^2+^ (^6^INT1_HS_)

Fe -1.11993200 -1.11003600 0.65662900

N -2.31801300 0.64589600 0.35282600

C -1.83447600 1.50702300 -0.56517100

C -4.40163900 1.80966500 0.36547900

H -5.40772600 1.90195400 0.75928600

C -3.57242200 0.78512500 0.81106400

H -3.89652800 0.05494400 1.54428900

C -2.60706700 2.54220700 -1.07195800

H -2.19357000 3.21967800 -1.81161600

C -3.91511400 2.69273700 -0.59873100

H -4.54328200 3.49228900 -0.97862500

N 0.34133400 0.15338700 1.89410300

N -0.09984500 -0.15901900 -0.98671600

C -0.70589600 -0.65651300 -2.26643200

H -1.75610000 -0.37619000 -2.30030700

H -0.16791400 -0.21224700 -3.11156800

C -0.36509700 1.31813600 -0.87684500

H -0.11622600 1.78068300 -1.83806200

O 0.65494400 3.99324000 -1.09257000

C 0.49936100 2.01364200 0.20998800

C 0.13095500 1.60621400 1.64318500

H 0.77722800 2.16998900 2.32563300

H -0.89659100 1.86127100 1.88853700

C 0.27291000 3.53653800 0.10458900

C 1.97802600 1.68658800 -0.05999800

H 2.22330400 1.95428300 -1.09349900

O 2.74865500 2.42819300 0.85814900

C 0.06796100 -0.14178900 3.32662100

H -0.96165200 0.12029900 3.56443500

N 0.47490200 -2.50833100 0.20469100

C 1.36485600 -2.08380800 -0.71439500

C 1.32237300 -4.73205000 0.01808200

H 1.28262700 -5.76791500 0.33687200

C 0.44524700 -3.80134300 0.56715600

H -0.29930600 -4.07279900 1.30723400

C 2.25736900 -2.95714600 -1.32197700

H 2.96248500 -2.59415300 -2.06189800

C 2.23589900 -4.30340100 -0.94696900

H 2.92557200 -5.00712100 -1.40226200

H -0.62585600 -1.74120900 -2.30567100

C 1.34341200 -0.58657000 -0.94373200

H 1.79388900 -0.34415200 -1.91477800

O 4.31810700 0.58473600 -0.78835000

C 2.16076900 0.16619900 0.14855000

C 1.74940900 -0.21322700 1.57977500

H 2.39756200 0.34491500 2.26668300

H 1.89366200 -1.27337300 1.77266800

C 3.63499000 -0.25995200 -0.00806700

H 3.65083300 2.51137000 0.50822000

H 0.22198100 -1.20245500 3.51834200

H 0.75212100 0.45114300 3.94530900

O -1.75591600 -1.70893300 2.20249700

O -2.22486500 -2.17331100 -0.32034800

O 4.08415900 -1.26032500 0.50203100

O -0.20965800 4.19673200 0.99251600

C -3.40095500 -2.09185500 -1.02170700

O -3.81339400 -1.04997300 -1.47212900

C -4.05352400 -3.44380400 -1.16865000

H -3.35367800 -4.14618800 -1.63272900

H -4.31244000 -3.84095200 -0.18099400

H -4.95253200 -3.34882700 -1.77912400

H -2.44112800 -2.39262400 2.07379400

C 5.72137500 0.25051300 -1.01557100

H 6.10067200 1.03906600 -1.66279900

H 6.25254400 0.22829500 -0.06219800

H 5.79332400 -0.72546600 -1.49913000

C 0.54456200 5.43362400 -1.28472100

H 1.17405000 5.94823800 -0.55641400

H 0.88999000 5.61436000 -2.30106400

H -0.49369400 5.74691300 -1.15699600

C -3.89084986 -2.26545180 4.68845354

H -3.00578981 -1.98595615 5.24176398

H -3.98181420 -3.25887565 4.27047819

C -5.01300585 -1.34429166 4.66672871

O -4.98212117 -0.20246913 5.11691783

O -6.08137579 -1.85935136 4.02508481

H -6.68368617 -1.11839526 3.82510809

[(L^2^)Fe^V^=O]^2+^ (^4^INT1_IS_)

Fe -1.20470400 -0.87309700 0.55411100

N -2.15915900 0.83826300 0.37526500

C -1.59451700 1.70880100 -0.49370000

C -4.04957300 2.26472200 0.61517600

H -5.01115000 2.45363400 1.07924900

C -3.35752400 1.09819300 0.92121700

H -3.74386700 0.35424100 1.60620500

C -2.24365000 2.87806600 -0.85865300

H -1.77345700 3.55948900 -1.55940800

C -3.49422200 3.15830800 -0.29975800

H -4.02275700 4.06602300 -0.57292300

N 0.32562600 0.07576600 1.82297900

N -0.11861700 -0.13732000 -1.02727300

C -0.73649100 -0.50340600 -2.34711200

H -1.73082100 -0.06772600 -2.41058100

H -0.09915500 -0.11945100 -3.15085300

C -0.17634200 1.36474800 -0.88156000

H 0.09120700 1.81655300 -1.84151200

O 1.27435300 3.85092800 -1.09971200

C 0.79999700 1.91623700 0.19238300

C 0.37741300 1.55550600 1.62217300

H 1.11263500 1.98078400 2.31268800

H -0.58772100 1.98514400 1.87840400

C 0.80690000 3.45591600 0.08862800

C 2.20746600 1.36487000 -0.09303500

H 2.47786900 1.58365400 -1.13232800

O 3.08853000 1.99037700 0.81059300

C -0.00762900 -0.18831300 3.25506900

H -0.98715100 0.22774800 3.48392000

N -0.04720700 -2.45404000 0.23154700

C 0.96641400 -2.25729500 -0.64325800

C 0.37384800 -4.79731200 0.23659400

H 0.11889000 -5.78189800 0.61298000

C -0.34998700 -3.68941000 0.66357600

H -1.17714100 -3.76692500 1.35674000

C 1.71009500 -3.32442400 -1.12726800

H 2.51843900 -3.14929900 -1.82776800

C 1.41034500 -4.61372500 -0.68000300

H 1.98203600 -5.46251400 -1.04156600

H -0.81681800 -1.58667100 -2.41788500

C 1.23781000 -0.80142100 -0.94676400

H 1.72805400 -0.69093100 -1.92091300

O 4.37593300 -0.05947200 -0.77141100

C 2.16439000 -0.16491400 0.13060300

C 1.68932300 -0.47153200 1.55849700

H 2.38943800 0.00231600 2.25590100

H 1.69392800 -1.54054200 1.75883600

C 3.55701200 -0.80953600 -0.02713600

H 3.99078500 1.92314700 0.45747800

H -0.02513000 -1.26215000 3.43342100

H 0.75141500 0.28271100 3.88931700

O -2.00553300 -1.41414000 2.01312200

O -2.43501100 -1.85308700 -0.45078200

O 3.83578600 -1.88521100 0.45164400

O 0.40618000 4.17918000 0.96822500

C -3.53507500 -1.47729200 -1.14805900

O -3.75383200 -0.35143000 -1.54431300

C -4.43707200 -2.66816400 -1.40240700

H -3.87793400 -3.46605200 -1.90097900

H -4.80555600 -3.07060300 -0.45248400

H -5.27773300 -2.35475300 -2.02283100

H -2.75411500 -1.98924600 1.76028000

C 5.71411400 -0.60121200 -0.99471800

H 6.22106300 0.13867000 -1.61123200

H 6.21988100 -0.73518800 -0.03675800

H 5.64085500 -1.56052600 -1.51025300

C 1.38759300 5.29154100 -1.28925000

H 2.07984000 5.70259000 -0.55211400

H 1.76863700 5.41835000 -2.30097200

H 0.40826700 5.76038000 -1.17290000

C -3.89084986 -2.26545180 4.68845354

H -3.00578981 -1.98595615 5.24176398

H -3.98181420 -3.25887565 4.27047819

C -5.01300585 -1.34429166 4.66672871

O -4.98212117 -0.20246913 5.11691783

O -6.08137579 -1.85935136 4.02508481

H -6.68368617 -1.11839526 3.82510809

[(L^2^)Fe^V^=O]^2+^ (^2^INT1_LS_)

Fe -1.15068900 -1.06984300 0.42899100

N -2.27369400 0.55054600 0.33183300

C -1.77069200 1.50199600 -0.48992900

C -4.29115400 1.79763400 0.57519100

H -5.27574900 1.88553500 1.02087200

C -3.49972000 0.68912500 0.86007200

H -3.82774600 -0.11309600 1.51138400

C -2.51553300 2.62189000 -0.83221200

H -2.09541900 3.36850800 -1.49755200

C -3.79833800 2.76914600 -0.29616900

H -4.40010000 3.63528000 -0.55243600

N 0.30721900 0.06882100 1.81213100

N -0.06318700 -0.15076600 -1.03553400

C -0.55052500 -0.51942200 -2.41612900

H -1.58779800 -0.21365600 -2.52905700

H 0.08265200 -0.00966000 -3.15020600

C -0.30998400 1.32757300 -0.86058900

H -0.09309200 1.82714300 -1.80931100

O 0.86026200 3.96361900 -1.04008200

C 0.58796000 1.97267000 0.22432900

C 0.18744100 1.54608000 1.63738200

H 0.85204800 2.04503300 2.35045800

H -0.82854100 1.85121500 1.87694800

C 0.43109800 3.50464400 0.14005500

C 2.05153500 1.58154300 -0.04867500

H 2.30634900 1.83598100 -1.08383200

O 2.84707800 2.29671200 0.86877200

C -0.00637900 -0.23324000 3.24048200

H -1.04112600 0.02459600 3.45609700

N 0.18126600 -2.51974500 0.17199500

C 1.22155200 -2.16814000 -0.62274400

C 0.87325500 -4.80716100 0.09071100

H 0.70054700 -5.83531900 0.38915400

C -0.00217700 -3.80907600 0.50398600

H -0.88595500 -4.03841300 1.08985500

C 2.11993400 -3.11878700 -1.09165200

H 2.94497500 -2.81848300 -1.72696300

C 1.95086900 -4.45512300 -0.72289800

H 2.64608600 -5.21058000 -1.07487800

H -0.48081800 -1.59761800 -2.54721800

C 1.34648300 -0.68185500 -0.91101500

H 1.84591400 -0.53213100 -1.87518200

O 4.37516000 0.41920500 -0.71784700

C 2.18496100 0.05551400 0.16799500

C 1.72702800 -0.31632300 1.57965400

H 2.35610500 0.21936800 2.30039300

H 1.84681000 -1.38275700 1.76232200

C 3.64444500 -0.41990100 0.02359200

H 3.75423300 2.33637000 0.52427600

H 0.16296200 -1.29247300 3.43878500

H 0.66082100 0.35047000 3.88486000

O -1.89087500 -1.55030200 1.92259400

O -2.11929700 -1.95550400 -0.73288100

O 4.04540500 -1.45388900 0.50802500

O -0.04823600 4.16987600 1.02624300

C -3.40921800 -1.69431100 -1.16896500

O -3.65744400 -0.75699600 -1.88315200

C -4.35871300 -2.75095600 -0.67668200

H -4.10526000 -3.71523200 -1.13067900

H -4.27123900 -2.86003700 0.41032200

H -5.37855900 -2.47385400 -0.95141000

H -1.47817500 -2.34243400 2.31881000

C 5.76894400 0.03814000 -0.93016300

H 6.19121200 0.83335400 -1.54161400

H 6.27922300 -0.03804100 0.03175100

H 5.81293100 -0.92202900 -1.44754900

C 0.82021200 5.41014000 -1.20975600

H 1.45793500 5.88291900 -0.46050100

H 1.19398400 5.59125300 -2.21593700

H -0.20462800 5.77013800 -1.09731300

C -4.23626491 -2.38896510 5.16614430

H -3.35120486 -2.10946945 5.71945474

H -4.32722925 -3.38238895 4.74816895

C -5.35842090 -1.46780496 5.14441947

O -5.32753622 -0.32598243 5.59460859

O -6.42679084 -1.98286466 4.50277557

H -7.02910122 -1.24190856 4.30279885

[(L^1^)Fe^IV^=O]^1+^ (^5^TS2_HS_)

Fe 0.69228 0.25210 0.19578

N 0.84369 -1.89372 -0.42372

C -0.26693 -2.35508 -1.02684

C 2.03591 -3.83895 -1.13345

H 2.97124 -4.38748 -1.16585

C 1.98049 -2.60297 -0.48830

H 2.85883 -2.14919 -0.03681

C -0.29102 -3.57657 -1.69495

H -1.20923 -3.91509 -2.16449

C 0.88174 -4.33506 -1.73938

H 0.89547 -5.29078 -2.25500

N -0.79840 -0.14258 1.81715

N -1.01160 -0.04007 -1.10534

C -0.59827 0.13531 -2.52573

H 0.19847 -0.56718 -2.76017

H -1.45810 -0.02369 -3.18990

C -1.48035 -1.45062 -0.91604

H -2.18469 -1.69828 -1.71911

C -2.24442 -1.67292 0.41935

C -1.36753 -1.50765 1.67134

H -1.98989 -1.72683 2.55026

H -0.53307 -2.21161 1.66011

C -2.81361 -3.10228 0.44410

C -3.39947 -0.64749 0.47225

H -4.00049 -0.72292 -0.43955

O -4.21448 -0.86276 1.62077

C -0.10071 -0.05922 3.12340

H 0.70544 -0.79166 3.14504

N -0.05760 2.29913 -0.16186

C -1.28125 2.34876 -0.72084

C 0.20074 4.64904 -0.50520

H 0.82083 5.53423 -0.41349

C 0.67970 3.41628 -0.06528

H 1.67714 3.28768 0.34165

C -1.82878 3.54114 -1.18698

H -2.82449 3.53964 -1.61823

C -1.07429 4.71092 -1.06952

H -1.47348 5.65600 -1.42558

H -0.19696 1.13733 -2.66434

C -2.01595 1.02193 -0.77028

H -2.76831 1.05661 -1.56558

C -2.78888 0.76374 0.55682

C -1.90031 0.85535 1.80840

H -2.53600 0.69259 2.69022

H -1.44760 1.84465 1.89811

C -3.90570 1.81613 0.69785

H -4.97610 -1.39803 1.35442

H 0.34464 0.92901 3.22989

H -0.80558 -0.25161 3.94407

O 2.01224 0.39929 1.41536

O 1.94009 0.52654 -1.32251

C 3.07843 1.14253 -1.27091

O 3.48023 1.80838 -0.29454

C 3.97125 0.95155 -2.47889

H 4.60734 1.82796 -2.62269

H 4.61467 0.08841 -2.26787

H 3.39107 0.74230 -3.38021

H 2.67246 1.01067 0.98036

C 3.73442 -0.73545 2.56325

H 3.19353 -1.67069 2.54399

H 3.71500 -0.11216 3.44699

C 4.61511 -0.42241 1.45211

O 4.68764 -1.07986 0.41776

O 5.29810 0.72307 1.65166

H 5.65727 1.00001 0.78789

H -1.99217 -3.83269 0.44377

H -3.37840 -3.23150 1.37697

H -4.53312 1.53095 1.54938

H -3.46321 2.80160 0.90579

O -4.65214 1.83935 -0.51558

H -5.47170 2.33396 -0.36577

O -3.65160 -3.26271 -0.69635

H -4.03550 -4.15253 -0.67842

[(L^1^)Fe^IV^=O]^1+^ (^3^TS2_IS_)

Fe 0.62398 0.17518 0.07288

N 0.75262 -1.77972 -0.31833

C -0.33426 -2.29469 -0.93685

C 1.96959 -3.78017 -0.76775

H 2.90347 -4.32607 -0.68949

C 1.89029 -2.48835 -0.24915

H 2.74414 -1.99781 0.20712

C -0.32013 -3.57296 -1.48353

H -1.21416 -3.95471 -1.96605

C 0.85069 -4.33119 -1.39053

H 0.88860 -5.33123 -1.81203

N -0.78496 -0.17609 1.72223

N -1.07851 -0.00423 -1.18379

C -0.74581 0.14095 -2.62476

H -0.02098 -0.62010 -2.90811

H -1.65681 0.04412 -3.23069

C -1.56139 -1.40099 -0.94254

H -2.22595 -1.69912 -1.75986

C -2.36958 -1.58060 0.37337

C -1.48383 -1.48623 1.62407

H -2.11651 -1.62303 2.51219

H -0.72640 -2.27131 1.63008

C -3.03471 -2.96995 0.36707

C -3.44132 -0.47582 0.41872

H -4.02339 -0.50457 -0.50805

O -4.28557 -0.70512 1.54198

C -0.02043 -0.15418 2.99430

H 0.71870 -0.95318 2.97467

N 0.15982 2.12488 -0.07107

C -1.04619 2.35374 -0.64438

C 0.64863 4.46384 -0.17100

H 1.34865 5.26677 0.03216

C 0.98942 3.15469 0.16425

H 1.94744 2.90184 0.59661

C -1.44277 3.63328 -1.01817

H -2.41288 3.77429 -1.48386

C -0.58186 4.70713 -0.77776

H -0.86832 5.71437 -1.06458

H -0.28243 1.11170 -2.79321

C -1.94785 1.14005 -0.77650

H -2.69867 1.32544 -1.55322

C -2.73118 0.89328 0.54393

C -1.82721 0.88319 1.78617

H -2.45876 0.70844 2.66809

H -1.32442 1.84275 1.91963

C -3.77438 2.00647 0.73791

H -5.18476 -0.43721 1.30209

H 0.50974 0.79255 3.07987

H -0.69951 -0.29341 3.84666

O 2.05835 0.29972 1.24354

O 1.76648 0.39992 -1.47752

C 2.92430 0.98194 -1.54263

O 3.49441 1.55636 -0.59793

C 3.59883 0.87931 -2.89760

H 4.29036 1.71298 -3.03616

H 4.17529 -0.05335 -2.92416

H 2.86505 0.84846 -3.70686

H 2.69726 0.87854 0.74899

C 3.53091 -0.47524 2.52460

H 3.01026 -1.40026 2.72601

H 3.51533 0.30834 3.27011

C 4.59193 -0.51379 1.53286

O 4.72688 -1.39725 0.69100

O 5.38790 0.57836 1.58495

H 5.96237 0.54748 0.79777

H -4.32009 1.80541 1.66919

H -3.27152 2.97798 0.84750

H -3.69933 -3.03084 1.23593

H -2.26350 -3.74874 0.46324

O -4.64499 1.99453 -0.38844

H -5.32638 2.67277 -0.26503

O -3.75433 -3.11301 -0.85320

H -4.33146 -3.88816 -0.78443

[(L^2^)Fe^IV^=O]^1+^ (^5^TS2_HS_)

Fe -1.05356 -0.31406 0.18309

N -1.18786 1.82309 -0.47203

C -0.08192 2.27376 -1.09049

C -2.34845 3.81116 -1.11028

H -3.26989 4.38347 -1.10561

C -2.30858 2.55909 -0.49494

H -3.18669 2.11605 -0.03264

C -0.04327 3.50632 -1.73407

H 0.86666 3.83692 -2.22458

C -1.19807 4.29359 -1.73249

H -1.19978 5.26165 -2.22426

N 0.47128 0.05085 1.75481

N 0.63651 -0.05634 -1.17268

C 0.18205 -0.23449 -2.58035

H -0.61595 0.47299 -2.79325

H 1.02326 -0.08413 -3.27045

C 1.11933 1.34865 -1.00349

H 1.81821 1.58406 -1.81594

O 3.28343 3.19789 -0.70419

C 1.88697 1.57058 0.33005

C 1.02827 1.42050 1.59634

H 1.66105 1.66465 2.45584

H 0.19814 2.12470 1.58391

C 2.43996 3.00695 0.32553

C 3.05556 0.57092 0.38670

H 3.65317 0.65237 -0.53402

O 3.82195 0.90200 1.51816

C -0.19085 -0.02999 3.08221

H -0.98927 0.70913 3.12622

N -0.31317 -2.37015 -0.16101

C 0.87555 -2.44657 -0.78975

C -0.67421 -4.70273 -0.52947

H -1.31687 -5.56872 -0.41368

C -1.08045 -3.46321 -0.03804

H -2.04770 -3.30925 0.42809

C 1.34091 -3.64439 -1.32475

H 2.29288 -3.67462 -1.83982

C 0.55351 -4.79053 -1.18463

H 0.89193 -5.73674 -1.59614

H -0.23120 -1.23357 -2.70302

C 1.63778 -1.12779 -0.86068

H 2.36741 -1.17283 -1.67666

O 3.99933 -2.40820 -0.52200

C 2.42657 -0.86041 0.45431

C 1.56759 -0.95162 1.73119

H 2.22648 -0.79204 2.59073

H 1.12009 -1.94121 1.83077

C 3.61819 -1.81213 0.61238

H 4.36732 0.14088 1.78766

H -0.63925 -1.01555 3.20054

H 0.53955 0.15810 3.88021

O -2.35194 -0.42457 1.42756

O -2.31860 -0.61089 -1.30707

C -3.46918 -1.20431 -1.22671

O -3.86552 -1.84692 -0.23405

C -4.37719 -1.01337 -2.42278

H -5.03224 -1.87920 -2.54348

H -5.00038 -0.13520 -2.21305

H -3.80849 -0.82769 -3.33649

H -3.03067 -1.03136 1.01709

C -4.04996 0.76474 2.58766

H -3.49487 1.69075 2.54120

H -4.02785 0.15993 3.48419

C -4.94422 0.43635 1.49184

O -5.01765 1.06963 0.44241

O -5.63940 -0.69537 1.72397

H -6.01812 -0.98270 0.87204

O 2.13467 3.85490 1.12959

O 4.22186 -1.92042 1.66390

C 3.89546 4.50790 -0.76852

H 4.47594 4.69162 0.13822

H 4.54023 4.48374 -1.64623

H 3.12638 5.27804 -0.86699

C 5.19200 -3.23104 -0.43141

H 5.03155 -4.04115 0.28295

H 5.34914 -3.61831 -1.43711

H 6.03984 -2.62335 -0.10875

[(L^2^)Fe^IV^=O]^1+^ (^3^TS2_IS_)

Fe -0.83403 -0.29865 0.28540

N -1.10891 1.63312 -0.05108

C -0.15304 2.22456 -0.80145

C -2.48391 3.56555 -0.25283

H -3.42857 4.04813 -0.02958

C -2.25706 2.27029 0.21099

H -2.99897 1.71081 0.76364

C -0.31296 3.51063 -1.29699

H 0.47184 3.96254 -1.89467

C -1.49978 4.19506 -1.01130

H -1.65254 5.20091 -1.39025

N 0.72921 0.08722 1.75323

N 0.65416 -0.02696 -1.15304

C 0.14699 -0.18157 -2.54598

H -0.64527 0.54241 -2.72292

H 0.96986 -0.03282 -3.25737

C 1.10244 1.39313 -0.97401

H 1.65328 1.71400 -1.86507

O 3.32202 3.21695 -0.93158

C 2.03578 1.59951 0.24925

C 1.31960 1.44354 1.59783

H 2.05172 1.62506 2.39123

H 0.52871 2.18198 1.70768

C 2.59806 3.03057 0.18560

C 3.19369 0.58820 0.16547

H 3.67167 0.65714 -0.82407

O 4.09705 0.92463 1.18826

C 0.14037 0.00421 3.11739

H -0.67017 0.72587 3.19780

N -0.30413 -2.20534 0.05094

C 0.83114 -2.38739 -0.66474

C -0.72518 -4.55522 -0.00118

H -1.36574 -5.38282 0.28282

C -1.06580 -3.25971 0.38269

H -1.97094 -3.03334 0.92836

C 1.21603 -3.65018 -1.09978

H 2.11945 -3.76305 -1.68525

C 0.42452 -4.75164 -0.76218

H 0.70415 -5.74655 -1.09502

H -0.28429 -1.17326 -2.66602

C 1.64497 -1.12094 -0.89313

H 2.28266 -1.24160 -1.77407

O 4.00633 -2.44498 -0.76829

C 2.57019 -0.83884 0.32390

C 1.83142 -0.90797 1.67512

H 2.55759 -0.71827 2.47212

H 1.40998 -1.90010 1.84024

C 3.76304 -1.79975 0.37711

H 4.64121 0.15146 1.42482

H -0.28076 -0.98825 3.26662

H 0.91410 0.21102 3.86835

O -2.01602 -0.47341 1.59270

O -2.15306 -0.55885 -1.11946

C -3.29250 -1.17107 -1.02945

O -3.70883 -1.77776 -0.02566

C -4.14866 -1.12024 -2.28172

H -5.14469 -0.76316 -2.00571

H -3.71754 -0.47205 -3.04679

H -4.26524 -2.13441 -2.67899

H -2.75394 -1.01087 1.21267

C -5.42181 -0.27124 1.91987

H -4.48792 -0.00983 2.39989

H -6.05413 -1.05015 2.32624

C -5.76976 0.39237 0.68680

O -5.10428 1.27659 0.15004

O -6.93302 -0.05248 0.15032

H -7.09378 0.48140 -0.64949

O 2.39148 3.87771 1.02128

O 4.47944 -1.87906 1.35817

C 3.94448 4.51769 -1.05808

H 4.62979 4.68339 -0.22392

H 4.48198 4.49085 -2.00516

H 3.18296 5.30143 -1.06087

C 5.19519 -3.27931 -0.78096

H 5.11082 -4.05817 -0.02034

H 5.23284 -3.70762 -1.78153

H 6.07892 -2.66979 -0.58192

[(L^1^)Fe^V^=O]^2+^ (^6^TS2_HS_)

Fe -0.69085 0.41947 0.04097

N 0.31725 2.30497 -0.21343

C 1.55736 2.22511 -0.73888

C 0.30452 4.66696 -0.55957

H -0.22049 5.61239 -0.47744

C -0.30562 3.49245 -0.13087

H -1.30850 3.47428 0.28119

C 2.22963 3.35528 -1.19273

H 3.23200 3.25356 -1.59526

C 1.59142 4.59493 -1.09965

H 2.09046 5.49322 -1.44996

N 0.71248 -0.09165 1.82310

N 1.05713 -0.12704 -1.07278

C 0.73746 0.02130 -2.53090

H 0.44217 1.04957 -2.73231

H 1.62625 -0.23334 -3.11947

C 2.15673 0.83718 -0.72458

H 2.93213 0.75927 -1.49279

O 4.82738 1.37935 -0.41226

H 5.69803 1.78350 -0.27365

C 2.84558 0.54707 0.63743

C 1.91754 0.78540 1.83700

H 2.48498 0.57666 2.75363

H 1.57636 1.82061 1.88348

C 4.06702 1.47789 0.78449

H 3.73283 2.51125 0.96043

H 4.63706 1.14960 1.66077

C 3.30139 -0.92886 0.63870

H 3.93557 -1.11538 -0.23334

O 4.00463 -1.18900 1.84564

C -0.05886 0.13556 3.07054

H -0.38504 1.17478 3.10898

N -1.01248 -1.66738 -0.29364

C 0.02428 -2.31555 -0.86443

C -2.44122 -3.52635 -0.73666

H -3.42829 -3.97095 -0.67621

C -2.22194 -2.24784 -0.23618

H -3.01471 -1.65151 0.20235

C -0.12194 -3.58953 -1.39946

H 0.73505 -4.08444 -1.84401

C -1.37629 -4.20302 -1.33356

H -1.52028 -5.19538 -1.74947

H -0.09094 -0.63448 -2.79078

C 1.34161 -1.57723 -0.79394

H 2.01823 -1.94674 -1.57071

O 3.18538 -3.61647 -0.52849

H 3.58344 -4.49674 -0.44211

C 2.04458 -1.82772 0.56946

C 1.14538 -1.51667 1.77472

H 1.71389 -1.73471 2.68844

H 0.24887 -2.13795 1.78022

C 2.45539 -3.31150 0.65158

H 1.56136 -3.94530 0.74582

H 3.06175 -3.44542 1.55545

H 4.86216 -1.58902 1.64045

H -0.93763 -0.50803 3.07391

H 0.56854 -0.08757 3.94259

O -1.98094 0.74898 1.24615

O -1.77612 0.90591 -1.36140

C -2.60549 0.35373 -2.28950

O -2.42005 -0.76072 -2.72798

C -3.72456 1.28119 -2.68576

H -4.27293 0.85735 -3.52815

H -4.39480 1.40936 -1.82902

H -3.32403 2.26487 -2.94932

H -2.82225 0.89879 0.73714

C -4.00201 1.13303 2.84498

H -3.50653 2.07764 2.66762

H -3.98107 0.68726 3.83318

C -4.83583 0.55288 1.78872

O -4.56866 0.66618 0.58933

O -5.87628 -0.12769 2.25830

H -6.41168 -0.46181 1.51052

[(L^1^)Fe^V^=O]^2+^ (^4^TS2_IS_)

Fe 0.61016 0.18355 0.03192

N 0.74360 -1.75270 -0.38276

C -0.35429 -2.29862 -0.96050

C 1.98301 -3.74198 -0.79021

H 2.92346 -4.27549 -0.70554

C 1.89662 -2.43947 -0.30344

H 2.74757 -1.93632 0.14661

C -0.32379 -3.58827 -1.47463

H -1.21992 -3.99834 -1.92892

C 0.86341 -4.32275 -1.38623

H 0.91175 -5.33131 -1.78518

N -0.76817 -0.15811 1.71637

N -1.10711 -0.02188 -1.16561

C -0.80094 0.12951 -2.62219

H -0.07662 -0.62409 -2.92432

H -1.72714 0.01515 -3.19642

C -1.58719 -1.42664 -0.92691

H -2.27270 -1.70884 -1.73112

C -2.35483 -1.61143 0.40888

C -1.43611 -1.49267 1.63132

H -2.04323 -1.62990 2.53468

H -0.65948 -2.25854 1.63071

C -2.99652 -3.01300 0.42630

C -3.45247 -0.52327 0.49414

H -4.08237 -0.56502 -0.39999

O -4.22064 -0.71871 1.67200

C 0.02430 -0.11229 2.97721

H 0.78126 -0.89421 2.95259

N 0.10054 2.10812 -0.11204

C -1.13018 2.33125 -0.63692

C 0.56617 4.44793 -0.16617

H 1.26168 5.25521 0.03560

C 0.93733 3.13778 0.11898

H 1.91044 2.88893 0.52029

C -1.55464 3.61651 -0.95288

H -2.54921 3.75612 -1.36385

C -0.69386 4.69128 -0.71556

H -1.00292 5.70341 -0.95775

H -0.36817 1.11153 -2.80389

C -2.00795 1.10714 -0.75364

H -2.76420 1.25807 -1.52883

C -2.76863 0.86067 0.57795

C -1.83431 0.88636 1.79476

H -2.43258 0.70024 2.69539

H -1.34794 1.85536 1.91390

C -3.82747 1.96812 0.76081

H -5.11827 -0.99233 1.43285

H 0.51811 0.85434 3.06129

H -0.64219 -0.26899 3.83329

O 1.95657 0.31570 1.13804

O 1.69933 0.41980 -1.51911

C 2.89245 0.95826 -1.56769

O 3.50922 1.35053 -0.56239

C 3.49809 1.02856 -2.94539

H 4.17441 1.88403 -3.00346

H 4.08573 0.11675 -3.10715

H 2.72941 1.08685 -3.71827

H 2.70525 0.76625 0.57986

C 3.82815 -0.27866 2.63129

H 3.20504 -1.12033 2.89681

H 3.86779 0.59432 3.27148

C 4.76126 -0.43808 1.51854

O 4.65561 -1.31871 0.67169

O 5.71152 0.50573 1.50947

H 6.28083 0.36193 0.72893

H -4.46745 1.68500 1.60373

H -3.33516 2.92003 1.01043

H -3.63013 -3.08408 1.31858

H -2.21497 -3.78326 0.50490

O -4.55695 2.06899 -0.45388

H -5.36153 2.58962 -0.30445

O -3.74473 -3.15192 -0.77243

H -4.27799 -3.96106 -0.72856

[(L^1^)Fe^V^=O]^2+^ (^2^TS2_LS_)

Fe 0.58714 0.22958 0.04285

N 0.83097 -1.68618 -0.38399

C -0.23687 -2.27640 -0.97619

C 2.16361 -3.60768 -0.83871

H 3.13250 -4.09071 -0.77336

C 2.01612 -2.31937 -0.32997

H 2.85522 -1.78168 0.09802

C -0.14908 -3.55465 -1.51087

H -1.02647 -3.99613 -1.97215

C 1.07047 -4.23529 -1.43566

H 1.16510 -5.23421 -1.85022

N -0.75301 -0.19351 1.70348

N -1.06886 -0.02774 -1.15457

C -0.75604 0.15120 -2.60956

H 0.00029 -0.56954 -2.91233

H -1.67322 0.00323 -3.18934

C -1.50204 -1.45679 -0.94323

H -2.16711 -1.74592 -1.76129

C -2.27504 -1.69447 0.37970

C -1.36806 -1.55359 1.60722

H -1.97199 -1.72077 2.50713

H -0.56194 -2.28811 1.60401

C -2.85544 -3.12283 0.36772

C -3.41776 -0.65602 0.47412

H -4.04342 -0.71586 -0.42187

O -4.17647 -0.90534 1.64797

C 0.03588 -0.13245 2.96846

H 0.84572 -0.85898 2.92237

N 0.00290 2.14120 -0.08368

C -1.22796 2.31445 -0.62443

C 0.35866 4.50185 -0.12097

H 1.01304 5.33889 0.09662

C 0.78457 3.20843 0.16605

H 1.75906 3.00723 0.58942

C -1.70909 3.57881 -0.94193

H -2.70403 3.67382 -1.36440

C -0.90195 4.69109 -0.68917

H -1.25326 5.68920 -0.93177

H -0.36417 1.15225 -2.77764

C -2.03915 1.04933 -0.75033

H -2.79514 1.15461 -1.53280

C -2.79268 0.75508 0.57514

C -1.86266 0.80536 1.79432

H -2.45290 0.58148 2.69120

H -1.41690 1.79167 1.92683

C -3.89821 1.81355 0.76648

H -5.08655 -1.13127 1.40646

H 0.46333 0.86192 3.08348

H -0.62094 -0.35862 3.81595

O 1.86227 0.52650 1.13588

O 1.69433 0.53244 -1.48455

C 2.87601 1.08212 -1.49482

O 3.44421 1.50591 -0.46483

C 3.55776 1.14660 -2.83260

H 4.29647 0.33672 -2.87046

H 2.84861 1.02320 -3.65236

H 4.09657 2.09305 -2.92366

H 2.66336 0.98499 0.59096

C 3.86738 -0.77742 2.62061

H 3.35669 -1.73157 2.61748

H 3.82276 -0.13606 3.49226

C 4.70110 -0.43430 1.48106

O 4.73053 -1.08360 0.43750

O 5.39362 0.69992 1.66938

H 5.88068 0.89529 0.84589

H -4.52255 1.49887 1.61032

H -3.44843 2.78528 1.01927

H -3.50941 -3.22847 1.24131

H -2.04344 -3.85926 0.46057

O -4.63440 1.88526 -0.44624

H -5.45167 2.38636 -0.29845

O -3.56330 -3.28262 -0.85294

H -4.07908 -4.10359 -0.82234

[(L^2^)Fe^V^=O]^2+^ (^6^TS2_HS_)

Fe 1.06499 -0.47808 0.00253

N 0.12836 -2.39240 -0.33211

C -1.09309 -2.35006 -0.90224

C 0.21447 -4.75572 -0.65860

H 0.75890 -5.68689 -0.54632

C 0.77829 -3.56182 -0.21840

H 1.76361 -3.51470 0.23189

C -1.71601 -3.49843 -1.37576

H -2.69950 -3.43550 -1.82929

C -1.05122 -4.72142 -1.24760

H -1.51477 -5.63457 -1.60757

N -0.39812 -0.03022 1.72838

N -0.67174 0.03104 -1.18003

C -0.29317 -0.07930 -2.62693

H 0.05452 -1.09052 -2.83074

H -1.16842 0.14619 -3.24743

C -1.74090 -0.98158 -0.88830

H -2.50302 -0.91599 -1.67290

O -4.51329 -1.55482 -0.39770

C -2.45396 -0.74842 0.47000

C -1.55007 -0.97402 1.69062

H -2.16089 -0.82657 2.58708

H -1.16754 -1.99183 1.72089

C -3.61887 -1.75561 0.57636

C -3.00299 0.68909 0.50051

H -3.62863 0.86509 -0.38678

O -3.74527 0.79475 1.68689

C 0.33940 -0.23503 3.00185

H 0.70303 -1.26097 3.04597

N 1.30482 1.62966 -0.27662

C 0.27805 2.25242 -0.89258

C 2.70045 3.53364 -0.62552

H 3.66829 4.00743 -0.50551

C 2.49064 2.24467 -0.14790

H 3.27491 1.66669 0.32882

C 0.42096 3.53020 -1.41844

H -0.41361 4.00526 -1.91886

C 1.65253 4.17853 -1.28197

H 1.79071 5.17451 -1.69107

H 0.51604 0.61477 -2.84472

C -1.02090 1.46545 -0.89036

H -1.67658 1.82262 -1.68982

O -2.54680 3.67894 -0.57702

C -1.77021 1.65171 0.45652

C -0.89554 1.37318 1.69062

H -1.50637 1.55540 2.58042

H -0.03659 2.04213 1.73405

C -2.35010 3.06904 0.59366

H -3.98694 1.72043 1.86605

H 1.18980 0.44445 3.04064

H -0.33002 -0.04405 3.84931

O 2.32238 -0.79644 1.24389

O 2.20946 -0.88150 -1.37384

C 3.04610 -0.27212 -2.25991

O 2.82516 0.84258 -2.68072

C 4.21789 -1.14061 -2.63575

H 4.77709 -0.67267 -3.44710

H 4.86128 -1.26242 -1.75788

H 3.87009 -2.13349 -2.93701

H 3.18834 -0.91578 0.76748

C 4.30760 -1.19037 2.91653

H 3.81272 -2.12748 2.70150

H 4.26215 -0.76987 3.91504

C 5.16587 -0.58284 1.89581

O 4.92732 -0.66471 0.68753

O 6.19473 0.08365 2.40764

H 6.74818 0.43842 1.68285

O -3.67388 -2.62033 1.41727

O -2.68697 3.51754 1.67018

C -5.69726 -2.40069 -0.35275

H -6.23404 -2.22726 0.58181

H -6.29641 -2.10136 -1.21094

H -5.40742 -3.45162 -0.41863

C -3.21386 4.97561 -0.51506

H -2.61573 5.66920 0.07871

H -3.29307 5.30392 -1.54988

H -4.20038 4.86020 -0.06263

[(L^2^)Fe^V^=O]^2+^ (^4^TS2_IS_)

Fe -0.97912 -0.23670 -0.00020

N -1.10422 1.70027 -0.41527

C -0.01865 2.23843 -1.02248

C -2.31531 3.72334 -0.73132

H -3.23995 4.27398 -0.59713

C -2.23875 2.40732 -0.27911

H -3.08357 1.91144 0.19012

C -0.04041 3.53876 -1.50508

H 0.84086 3.94906 -1.98667

C -1.20680 4.29634 -1.35276

H -1.24577 5.31678 -1.72088

N 0.43729 0.07281 1.63798

N 0.71281 -0.05146 -1.25306

C 0.36331 -0.20030 -2.70025

H -0.36500 0.55817 -2.97919

H 1.27223 -0.09180 -3.30306

C 1.20581 1.35025 -1.02770

H 1.87928 1.62712 -1.84550

O 3.49054 3.07704 -0.68036

C 1.98443 1.52435 0.30073

C 1.09136 1.41278 1.54303

H 1.71872 1.56677 2.42570

H 0.32193 2.18118 1.55219

C 2.60385 2.93888 0.30978

C 3.09882 0.46448 0.37432

H 3.71603 0.51232 -0.53527

O 3.84090 0.77027 1.52346

C -0.31852 0.01949 2.92311

H -1.06899 0.80746 2.92762

N -0.48706 -2.16119 -0.14710

C 0.71962 -2.40761 -0.71790

C -1.00600 -4.49056 -0.17739

H -1.70886 -5.28272 0.05629

C -1.33460 -3.17194 0.12082

H -2.28411 -2.90088 0.56212

C 1.09433 -3.70161 -1.05897

H 2.05802 -3.87210 -1.52198

C 0.21980 -4.75770 -0.78654

H 0.49432 -5.77450 -1.04979

H -0.08238 -1.17883 -2.86861

C 1.61565 -1.19031 -0.86737

H 2.34012 -1.35115 -1.66990

O 3.90849 -2.57710 -0.47738

C 2.40249 -0.93775 0.44552

C 1.50435 -0.97206 1.69260

H 2.13318 -0.78820 2.56829

H 1.02964 -1.94429 1.82431

C 3.53849 -1.95528 0.64350

H 4.44199 0.04009 1.75613

H -0.81473 -0.94534 3.01409

H 0.37619 0.16613 3.75763

O -2.29844 -0.35314 1.13030

O -2.10323 -0.46842 -1.52940

C -3.30708 -0.98326 -1.54655

O -3.90672 -1.35620 -0.52282

C -3.94936 -1.05365 -2.90701

H -4.53750 -0.13969 -3.05427

H -3.20234 -1.11725 -3.70037

H -4.63157 -1.90566 -2.94520

H -3.07317 -0.78686 0.58603

C -4.14529 0.26803 2.68588

H -3.49457 1.09513 2.93069

H -4.19039 -0.60075 3.33160

C -5.09545 0.44063 1.59014

O -4.98581 1.31265 0.73456

O -6.06711 -0.48089 1.60556

H -6.65061 -0.32636 0.83763

O 2.28356 3.79508 1.09858

O 4.09180 -2.08095 1.71678

C 4.19946 4.34886 -0.72286

H 4.76858 4.47878 0.19951

H 4.85976 4.28011 -1.58552

H 3.48709 5.16921 -0.83294

C 5.07174 -3.45431 -0.36385

H 4.86448 -4.24834 0.35569

H 5.22469 -3.85319 -1.36499

H 5.93615 -2.87516 -0.03472

[(L^2^)Fe^V^=O]^2+^ (^2^TS2_LS_)

Fe 1.06498600 -0.47808000 0.00253000

N 0.23049533 -2.39022560 -0.42293224

C -0.99095767 -2.34788560 -0.99306024

C 0.31660333 -4.75354860 -0.74941824

H 0.86103433 -5.68472060 -0.63713724

C 0.88042333 -3.55964760 -0.30921524

H 1.86574033 -3.51252560 0.14107176

C -1.61387967 -3.49625760 -1.46658324

H -2.59736567 -3.43332860 -1.92011124

C -0.94908267 -4.71924260 -1.33841924

H -1.41263367 -5.63239960 -1.69838924

N -0.29598367 -0.02804660 1.63755876

N -0.56960567 0.03321740 -1.27084924

C -0.19103267 -0.07712260 -2.71774624

H 0.15665533 -1.08834960 -2.92156124

H -1.06628767 0.14836740 -3.33824924

C -1.63876667 -0.97940260 -0.97911624

H -2.40088267 -0.91381260 -1.76371524

O -4.41116067 -1.55265160 -0.48852024

C -2.35182667 -0.74624660 0.37918176

C -1.44793967 -0.97184860 1.59979776

H -2.05875567 -0.82439260 2.49626076

H -1.06540767 -1.98966060 1.63007276

C -3.51673967 -1.75344060 0.48553676

C -2.90085767 0.69126240 0.40968676

H -3.52649767 0.86726840 -0.47760424

O -3.64314067 0.79692240 1.59607176

C 0.44153733 -0.23285560 2.91102976

H 0.80516133 -1.25879960 2.95514776

N 1.40695033 1.63183640 -0.36743624

C 0.38018833 2.25459640 -0.98340224

C 2.80258133 3.53580940 -0.71634324

H 3.77042733 4.00960540 -0.59632924

C 2.59277433 2.24684840 -0.23871624

H 3.37703933 1.66886340 0.23799676

C 0.52308933 3.53237540 -1.50925924

H -0.31147767 4.00743540 -2.00968224

C 1.75466233 4.18070140 -1.37278524

H 1.89284733 5.17667940 -1.78189324

H 0.61816933 0.61694240 -2.93554124

C -0.91876467 1.46762040 -0.98117424

H -1.57444767 1.82479340 -1.78063424

O -2.44466667 3.68110940 -0.66783924

C -1.66807467 1.65388540 0.36570176

C -0.79340967 1.37535240 1.59979876

H -1.40423867 1.55757140 2.48960476

H 0.06554133 2.04430040 1.64323476

C -2.24796367 3.07121440 0.50284476

H -3.88481167 1.72260740 1.77523176

H 1.29192933 0.44662440 2.94982076

H -0.22788267 -0.04188060 3.75848676

O 2.32238300 -0.79644400 1.24388700

O 2.20945600 -0.88149700 -1.37383700

C 3.04610100 -0.27211700 -2.25990900

O 2.82515800 0.84258400 -2.68071900

C 4.21789400 -1.14060700 -2.63575000

H 4.77709100 -0.67266600 -3.44710100

H 4.86128300 -1.26242000 -1.75787500

H 3.87008800 -2.13349100 -2.93701300

H 3.18833600 -0.91577900 0.76747800

C 4.44033724 -1.21670688 3.02836743

H 3.94544924 -2.15381388 2.81333543

H 4.39488224 -0.79621188 4.02687543

C 5.29860224 -0.60917788 2.00764643

O 5.06004924 -0.69104588 0.79936043

O 6.32746724 0.05730812 2.51947243

H 6.88091624 0.41207912 1.79468243

O -3.57174267 -2.61815960 1.32645176

O -2.58483367 3.51971540 1.57935976

C -5.59513167 -2.39851160 -0.44356524

H -6.13190167 -2.22508260 0.49098976

H -6.19427767 -2.09918260 -1.30175824

H -5.30528867 -3.44944660 -0.50944624

C -3.11172367 4.97777940 -0.60587924

H -2.51359967 5.67137440 -0.01211124

H -3.19094067 5.30609840 -1.64069524

H -4.09824167 4.86237140 -0.15344624

[(L^1^)Fe^IV^=O]^1+^ (^5^P_HS_)

Fe 1.21549100 -0.48637000 0.36946000

N 0.17715900 -2.35988000 -0.11104000

C -0.97124900 -2.20131800 -0.79733800

C 0.07549600 -4.72224800 -0.48235700

H 0.52365300 -5.69988700 -0.34134000

C 0.69393100 -3.58824800 0.03868500

H 1.62564000 -3.64016100 0.59340800

C -1.65130800 -3.28420500 -1.35041700

H -2.58138100 -3.11322400 -1.88280000

C -1.11877500 -4.56475900 -1.18758600

H -1.62603500 -5.42626200 -1.61133300

N -0.38694400 0.14827700 1.83039500

N -0.31550800 0.12587600 -1.10113500

C 0.24978900 -0.10370100 -2.45519400

H 0.51134700 -1.15621400 -2.56646400

H -0.46963000 0.18405800 -3.23476900

C -1.48795200 -0.77362700 -0.89025500

H -2.15904600 -0.69764300 -1.75368300

O -4.24027800 -1.15636800 -0.92130200

H -5.13977000 -1.51191800 -0.86498500

C -2.34664800 -0.41130000 0.36327000

C -1.62384500 -0.65626700 1.70285700

H -2.31614700 -0.40512000 2.52073000

H -1.34910900 -1.70801100 1.80656100

C -3.62527700 -1.27074800 0.35914500

H -3.36859700 -2.31782100 0.57888600

H -4.28301500 -0.90141500 1.15348100

C -2.71174600 1.08082300 0.25001900

H -3.18066300 1.26112200 -0.72274900

O -3.61350500 1.41423200 1.30152700

C 0.25900500 -0.11021400 3.13472400

H 0.51867600 -1.16986700 3.20223300

N 1.74986400 1.59402900 -0.10722700

C 0.81516900 2.26138000 -0.81148300

C 3.31260800 3.36471400 -0.49760400

H 4.31089800 3.76417600 -0.35498900

C 2.97067900 2.12738400 0.04350000

H 3.67385300 1.53035700 0.61590900

C 1.07919400 3.50149800 -1.38879000

H 0.29855800 4.00924500 -1.94647700

C 2.34819500 4.06190000 -1.22678300

H 2.58196300 5.02525500 -1.66988300

H 1.16732000 0.47414300 -2.56917600

C -0.54473300 1.58586900 -0.89562500

H -1.08930200 1.99300000 -1.75679700

O -2.41148100 3.72726800 -0.84748400

H -2.66648600 4.66220700 -0.82983200

C -1.41135900 1.90967600 0.36130000

C -0.72265100 1.58404100 1.70153800

H -1.40292500 1.87739100 2.51503600

H 0.20190500 2.15492400 1.81158300

C -1.74814300 3.41061200 0.37270400

H -0.82810700 4.00057300 0.49273900

H -2.39001000 3.61078400 1.24069300

H -4.24657400 2.06260600 0.96059200

H 1.18403800 0.46877600 3.19809100

H -0.39961600 0.15889600 3.97369400

O 2.86687100 -1.10052000 1.35583500

C 3.53936000 -1.38523600 0.28159000

O 3.01905600 -1.20836000 -0.84725600

C 4.93857000 -1.92362900 0.44354600

H 5.54608500 -1.20268700 1.00113900

H 4.91051800 -2.84248100 1.03900900

H 5.39202800 -2.12168200 -0.52863600

[(L^1^)Fe^IV^=O]^1+^ (^3^P_IS_)

Fe 1.19899600 -0.09695200 0.22865100

N 0.92319400 -2.04582700 0.00508900

C -0.20217600 -2.34972900 -0.68776200

C 1.58121800 -4.34522900 -0.06425100

H 2.30770100 -5.10489500 0.20308500

C 1.79427100 -3.02168600 0.31129300

H 2.66703000 -2.70048400 0.86789700

C -0.47352700 -3.64785300 -1.10724700

H -1.38585800 -3.84588200 -1.66055200

C 0.43309500 -4.66251500 -0.79065500

H 0.24558200 -5.68336900 -1.10905600

N -0.51948900 0.03239000 1.79297400

N -0.32280200 0.03328000 -1.12368800

C 0.27953200 -0.01853600 -2.48540900

H 0.83656300 -0.94841100 -2.59947400

H -0.50424200 0.04827000 -3.25121100

C -1.16088800 -1.19215700 -0.90506700

H -1.76376900 -1.37808900 -1.79970600

O -3.66420400 -2.37068800 -1.08182300

H -4.43204700 -2.96123800 -1.06535700

C -2.16376000 -1.07824400 0.28680400

C -1.46897700 -1.08952300 1.66218900

H -2.23794100 -1.03334600 2.44852000

H -0.91574000 -2.02053300 1.80468100

C -3.13623600 -2.27159300 0.23784300

H -2.60410900 -3.19294100 0.51774000

H -3.92656100 -2.09191200 0.97443100

C -2.93382500 0.24499100 0.11205400

H -3.36418200 0.27749600 -0.89468200

O -3.96535100 0.30351800 1.09182000

C 0.20580100 -0.03377700 3.07372800

H 0.76661900 -0.97092600 3.11670000

N 1.25155900 1.86803800 0.00546400

C 0.19808200 2.35805000 -0.69414400

C 2.30356100 4.01594400 -0.07974300

H 3.15101400 4.63875200 0.18524000

C 2.27876400 2.67888000 0.30819500

H 3.07974500 2.21454800 0.87185000

C 0.16491800 3.67796700 -1.13109700

H -0.68831900 4.02870000 -1.70283300

C 1.23416400 4.52172900 -0.81865700

H 1.23190100 5.55517600 -1.15118800

H 0.98785100 0.80191600 -2.59968400

C -0.94439400 1.38012000 -0.90446000

H -1.51514900 1.66623700 -1.79535400

O -3.37634100 2.86327400 -0.98538600

H -3.89165700 3.68413000 -0.99628200

C -1.94496200 1.42177400 0.29246100

C -1.25877900 1.30233900 1.66676700

H -2.02962700 1.37149200 2.44979600

H -0.55408200 2.12353300 1.81818300

C -2.71049700 2.75477200 0.26893000

H -2.01365700 3.59013600 0.42778600

H -3.42414700 2.75309600 1.10313300

H -4.72377100 0.76254400 0.70202800

H 0.92289500 0.79006200 3.12032900

H -0.47586200 0.02546400 3.93597000

O 2.98616000 -0.25964100 1.10280800

C 3.70428600 -0.30163700 0.02369700

O 3.15851700 -0.24053700 -1.10221800

C 5.20496700 -0.39114200 0.17757000

H 5.61158200 0.61879500 0.31236700

H 5.47001200 -0.97590300 1.06201300

H 5.65303000 -0.82188900 -0.72004400

[(L^1^)Fe^IV^=O]^1+^ (^1^P_LS_)

Fe 0.10811700 -1.13938800 0.21882800

N -1.86079300 -1.23570600 -0.02223100

C -2.34512500 -0.19964800 -0.74694300

C -4.00171500 -2.30064500 -0.14295700

H -4.62556000 -3.14781300 0.12104800

C -2.67320300 -2.26231200 0.27479900

H -2.22640100 -3.05254400 0.86718600

C -3.65668200 -0.17004400 -1.20690100

H -4.00052200 0.68656400 -1.77753100

C -4.50050900 -1.24115900 -0.90082600

H -5.52902800 -1.24608300 -1.24831800

N -0.02969700 0.34205600 1.71140300

N -0.01974300 0.31344900 -1.14514800

C 0.02935300 -0.27392800 -2.51085700

H -0.79139900 -0.98111200 -2.62671100

H -0.04156200 0.51387000 -3.27243200

C -1.36369600 0.94023100 -0.92775600

H -1.63721100 1.54126000 -1.80072200

O -2.95589500 3.21401800 -0.97123700

H -3.67577200 3.85998200 -0.91610400

C -1.42052500 1.88341400 0.31277900

C -1.29768900 1.11625500 1.64246700

H -1.33288200 1.83760400 2.47101300

H -2.12715500 0.42001600 1.77183200

C -2.76560900 2.63304500 0.31539000

H -3.57942700 1.93202000 0.55365900

H -2.72542400 3.39674100 1.09963000

C -0.25399300 2.88020900 0.18622200

H -0.30482700 3.36450000 -0.79453300

O -0.36811300 3.85053900 1.22155900

C 0.03490400 -0.39678000 2.99335500

H -0.78776400 -1.11254500 3.02985600

N 2.06412000 -0.88555200 -0.01807400

C 2.35727100 0.21100100 -0.75704500

C 4.35172100 -1.57867400 -0.16452400

H 5.11204600 -2.30694100 0.09631000

C 3.04139100 -1.75802600 0.27400300

H 2.74085900 -2.60782900 0.87626000

C 3.63754800 0.44911400 -1.24389500

H 3.82638800 1.33483500 -1.84216100

C 4.65403300 -0.46213800 -0.94339100

H 5.66204400 -0.30186500 -1.31325700

H 0.95977900 -0.82826200 -2.63043600

C 1.19380200 1.16625100 -0.93083700

H 1.35751200 1.81147600 -1.80170800

O 2.20359600 3.85851400 -0.85801800

H 2.93926500 4.48955300 -0.85282500

C 1.07503500 2.09855200 0.31177300

C 1.08289800 1.32417500 1.64246500

H 0.98274200 2.04588100 2.46496800

H 2.02207000 0.78655800 1.78088600

C 2.25680900 3.08046400 0.33344200

H 3.20371000 2.52760200 0.41186400

H 2.16371700 3.71057700 1.22824600

H -0.00491400 4.68441300 0.88801400

H 0.96936800 -0.95889000 3.03016500

H -0.02534200 0.28909300 3.85015200

O 0.25983300 -2.88146900 1.27542000

C 0.31532800 -3.51472300 0.16618700

O 0.26301100 -2.82910200 -0.90531000

C 0.42264000 -5.01492600 0.12106500

H 1.23866800 -5.30843400 -0.54662100

H 0.58577000 -5.42172500 1.12063200

H -0.50038800 -5.43239500 -0.29696000

[(L^2^)Fe^IV^=O]^1+^ (^5^P_HS_)

Fe 1.68386900 -0.14032800 0.37901000

N 1.22045500 -2.23323500 -0.07871200

C 0.09588100 -2.42120100 -0.79490200

C 1.78267000 -4.54281700 -0.35505600

H 2.47483600 -5.35489500 -0.16073400

C 2.04996300 -3.26493900 0.13167900

H 2.94111300 -3.03734100 0.70859700

C -0.23973600 -3.66442200 -1.32274100

H -1.15301500 -3.78136200 -1.89737200

C 0.61711100 -4.74369900 -1.09468200

H 0.37937800 -5.72472800 -1.49420600

N -0.05048200 0.01713500 1.80188400

N 0.05349400 -0.00108300 -1.13375700

C 0.69075500 -0.06814000 -2.47374900

H 1.24886600 -1.00013800 -2.56120400

H -0.06392300 -0.00782900 -3.27096400

C -0.80541000 -1.20161200 -0.92556900

H -1.46242100 -1.33360600 -1.79518200

C -1.73704000 -1.08635000 0.32566300

C -1.00014200 -1.11165200 1.67873700

H -1.75369200 -1.06783100 2.47532700

H -0.45262300 -2.04540900 1.79758200

C -2.52895400 0.22283500 0.18570000

H -3.00429200 0.24907900 -0.80149500

O -3.50434800 0.25440700 1.21048000

C 0.63123500 -0.03492700 3.11406300

H 1.18826700 -0.97250500 3.19080000

N 1.59242600 1.99744500 -0.09567600

C 0.53309900 2.37033400 -0.83829800

C 2.60623000 4.13287800 -0.46665800

H 3.44434800 4.79997900 -0.29679700

C 2.60820700 2.85343300 0.08397600

H 3.43187200 2.48067500 0.68505900

C 0.46110300 3.62532200 -1.43386200

H -0.40222100 3.89654800 -2.03284000

C 1.51342000 4.52241200 -1.24106300

H 1.48080500 5.50877800 -1.69320400

H 1.40583800 0.74781300 -2.57612900

C -0.57943800 1.33535400 -0.94195200

H -1.20744200 1.56988600 -1.81538500

C -1.52065800 1.38531700 0.31145000

C -0.78497100 1.29252200 1.66419600

H -1.53721700 1.38109100 2.46079300

H -0.08459200 2.11897100 1.77148600

H -4.17445700 0.90858500 0.95579500

H 1.34114800 0.79373100 3.18235600

H -0.08586300 0.03014200 3.94491900

O 3.48184900 -0.30532000 1.26092700

C -2.70469400 -2.28072300 0.30870300

O -2.74180000 -3.13865500 1.15867200

O -3.48290800 -2.26368600 -0.78922500

C -2.22922600 2.74671700 0.28376300

O -1.78697000 3.75034100 0.79311100

O -3.38775800 2.69983000 -0.40573600

C -4.09728800 3.95907900 -0.51901800

H -4.99163300 3.73572600 -1.09923200

H -4.35527300 4.33472300 0.47369700

H -3.47300100 4.69505200 -1.03081800

C -4.45644900 -3.33022000 -0.87447200

H -5.13708300 -3.28272300 -0.02142100

H -4.99010800 -3.16221100 -1.80927200

H -3.95394000 -4.30074300 -0.87904000

C 4.15702100 -0.36083100 0.15097000

O 3.55582200 -0.30044500 -0.94885800

C 5.65542000 -0.49690300 0.24155400

H 6.06594200 0.34756600 0.80547400

H 5.90746400 -1.40432100 0.80070300

H 6.10133000 -0.53438800 -0.75327800

[(L^2^)Fe^IV^=O]^1+^ (^3^P_IS_)

Fe 1.59167200 -0.08785700 0.20988500

N 1.36298200 -2.03958700 0.00138800

C 0.24281400 -2.38274900 -0.68024300

C 2.05268800 -4.33032600 0.00866500

H 2.78864700 -5.07069200 0.30253300

C 2.24917900 -2.99122000 0.33676700

H 3.11920000 -2.63832300 0.87842800

C -0.01106800 -3.69650300 -1.05431600

H -0.91427300 -3.93622600 -1.60573100

C 0.90797000 -4.68840700 -0.70217600

H 0.73069200 -5.72233900 -0.98089500

N -0.10973500 0.02545900 1.77186300

N 0.05213800 -0.00174500 -1.15142500

C 0.64954400 -0.04779700 -2.51533400

H 1.23571700 -0.96032500 -2.61995900

H -0.13884800 -0.01223300 -3.27905900

C -0.74376800 -1.24931100 -0.91304000

H -1.35314100 -1.46318700 -1.79908900

C -1.72585900 -1.14257900 0.29834000

C -1.02394100 -1.12843400 1.66893400

H -1.79272200 -1.09268100 2.45193300

H -0.45646900 -2.04710100 1.81341600

C -2.54865500 0.14198100 0.11261200

H -2.97999200 0.14784900 -0.89529300

O -3.56792100 0.14572800 1.09386800

C 0.63168600 -0.00494700 3.04674700

H 1.22441200 -0.92229700 3.09110000

N 1.58996600 1.87610700 -0.01613700

C 0.52872400 2.34170000 -0.71990500

C 2.58472000 4.05069200 -0.08432900

H 3.41194800 4.69556600 0.19137800

C 2.59379400 2.71176500 0.29679800

H 3.40410900 2.26350100 0.86014300

C 0.46383900 3.66056400 -1.15106400

H -0.39302300 4.00475000 -1.71967600

C 1.50729700 4.53071700 -0.82747700

H 1.47552000 5.56624600 -1.15100400

H 1.33293200 0.79195200 -2.63832600

C -0.59272800 1.33415700 -0.93182800

H -1.17282000 1.60601600 -1.82512800

C -1.58979200 1.34185800 0.27846100

C -0.88966400 1.26968000 1.64978400

H -1.66051800 1.32162800 2.43237000

H -0.22741900 2.12452000 1.77957700

H -4.24958500 0.77361800 0.80593600

H 1.31667100 0.84609900 3.08041200

H -0.04440300 0.03430900 3.91359100

O 3.42225400 -0.18114700 1.00174000

C -2.65989100 -2.36279600 0.26410600

O -2.70140300 -3.21098600 1.12347200

O -3.40063000 -2.38011500 -0.85925400

C -2.35107100 2.67340900 0.22447600

O -1.97579700 3.69064600 0.75999700

O -3.47164200 2.58424200 -0.52121300

C -4.22839700 3.81323500 -0.66128300

H -5.08603500 3.55526100 -1.28114800

H -4.54524000 4.17233300 0.32041300

H -3.61432100 4.57754200 -1.14305500

C -4.34755300 -3.46929900 -0.96083700

H -5.05897300 -3.42225100 -0.13325500

H -4.85072200 -3.32840900 -1.91672700

H -3.82506900 -4.42873200 -0.93094700

C 4.09257000 -0.23342700 -0.10745600

O 3.49456900 -0.22114100 -1.20839000

C 5.60004700 -0.27458500 -0.01791700

H 5.97753700 0.74421600 0.13298800

H 5.91918700 -0.87351000 0.83904400

H 6.02573900 -0.66652400 -0.94347400

[(L^2^)Fe^IV^=O]^1+^ (^1^P_LS_)

Fe 1.50006400 -0.28082500 0.17718200

N 1.04738200 -2.20095800 -0.04024900

C -0.08200500 -2.39130700 -0.76148600

C 1.45384900 -4.55941900 -0.08926800

H 2.08472500 -5.39171500 0.20337700

C 1.79871100 -3.26302600 0.28848800

H 2.68356900 -3.04298200 0.87453900

C -0.48484700 -3.65089900 -1.18546500

H -1.39597900 -3.76148400 -1.76446400

C 0.29788000 -4.75712100 -0.84278200

H 0.00662300 -5.75384500 -1.15875300

N 0.05115500 0.00515400 1.67618200

N 0.06076900 -0.01089400 -1.18778900

C 0.63889000 -0.13337200 -2.55354100

H 1.09386900 -1.11742500 -2.66058700

H -0.13789900 0.00928300 -3.31675200

C -0.90491900 -1.13267800 -0.95810500

H -1.56683000 -1.23764400 -1.82570200

C -1.80782300 -0.91340400 0.29643600

C -1.03719200 -1.00482000 1.62591300

H -1.74507500 -0.84188500 2.44704700

H -0.61033200 -1.99637900 1.75824600

C -2.46458200 0.46869600 0.16084200

H -2.94405300 0.54201300 -0.82212800

O -3.42092700 0.59797400 1.19580700

C 0.78749400 -0.12447800 2.95593200

H 1.25209000 -1.11062700 2.99601700

N 1.79064000 1.66694800 -0.06546400

C 0.82011800 2.25295900 -0.80493400

C 3.07947000 3.67990600 -0.19152800

H 3.98340100 4.21390300 0.08076200

C 2.89403100 2.36769800 0.23822300

H 3.62486600 1.84359400 0.84322500

C 0.94164800 3.54987400 -1.28507300

H 0.14690200 3.98668300 -1.88034700

C 2.09263600 4.27793000 -0.97341800

H 2.21145600 5.29510600 -1.33289900

H 1.42732700 0.60835300 -2.67584100

C -0.41888800 1.39331300 -0.97723700

H -0.99793400 1.72349800 -1.85110400

C -1.34521800 1.52598400 0.27799000

C -0.58743400 1.34275100 1.60714800

H -1.30724300 1.45471500 2.42853000

H 0.17284000 2.11138300 1.72439600

H -4.02730200 1.31352800 0.94661700

H 1.58044500 0.62423600 2.98383300

H 0.11416400 0.00820600 3.81388100

O 3.22099500 -0.60816100 1.21792800

C -2.89065300 -2.00302600 0.30757800

O -3.00387400 -2.84045900 1.17111100

O -3.67591800 -1.92047400 -0.78187500

C -1.91847000 2.94927100 0.27216000

O -1.38247200 3.89840500 0.79569700

O -3.07624800 3.02278200 -0.41612000

C -3.66338400 4.34520700 -0.51009000

H -4.57634600 4.21542100 -1.08976200

H -3.88196600 4.73016700 0.48849200

H -2.97351900 5.02539700 -1.01480400

C -4.75602200 -2.88108000 -0.84141900

H -5.41922200 -2.74948400 0.01654800

H -5.27874900 -2.67284800 -1.77427400

H -4.35737400 -3.89861100 -0.83444300

C 3.83816700 -0.72979900 0.10461200

O 3.15346500 -0.60546400 -0.96170300

C 5.31895200 -0.98916500 0.05151800

H 5.83150100 -0.08846100 -0.30596700

H 5.70051100 -1.25109200 1.04001100

H 5.53163700 -1.78811600 -0.66493100

[(L^1^)Fe^V^=O]^2+^ (^6^P_HS_)

Fe -0.00750400 1.21953100 0.43812500

N 2.06195500 1.06537800 -0.01720000

C 2.38805100 -0.01738600 -0.76097600

C 4.28132200 1.91896100 -0.25283500

H 5.00133600 2.69998500 -0.03442000

C 2.98216800 2.01674500 0.23207300

H 2.64779700 2.85556800 0.83299900

C 3.66747100 -0.17894900 -1.27980800

H 3.89757400 -1.06518000 -1.86208200

C 4.62682200 0.80513700 -1.02306000

H 5.63136500 0.70497800 -1.42259200

N 0.00286800 -0.45964000 1.81939300

N 0.00172900 -0.28927400 -1.10879800

C -0.00219000 0.35339100 -2.45691700

H 0.87383100 0.99269200 -2.55784700

H 0.00247400 -0.41590900 -3.23777800

C 1.28436200 -1.04425200 -0.91443900

H 1.47127000 -1.65426700 -1.80347400

O 2.65978100 -3.42291500 -1.04605400

H 3.33870400 -4.11575200 -1.05177100

C 1.26759900 -2.02211400 0.30140300

C 1.22367600 -1.30708400 1.66472500

H 1.21908900 -2.06634000 2.45677100

H 2.09685400 -0.66996200 1.81360800

C 2.54801400 -2.88237000 0.26232700

H 3.42283500 -2.26523800 0.51657400

H 2.45198200 -3.66480800 1.02385900

C 0.01833100 -2.92451600 0.17127300

H 0.02169400 -3.41441400 -0.80711000

O 0.02543200 -3.87892300 1.22261400

C -0.00134200 0.21584400 3.14730100

H 0.88495100 0.85044700 3.23451400

N -2.07521200 1.03928500 -0.01695700

C -2.38771500 -0.04706400 -0.76132500

C -4.30484400 1.86561500 -0.25301200

H -5.03449600 2.63760300 -0.03445500

C -3.00718200 1.97907600 0.23250500

H -2.68334600 2.82154800 0.83407700

C -3.66479800 -0.22397300 -1.28088900

H -3.88379100 -1.11245600 -1.86402200

C -4.63628100 0.74814000 -1.02410100

H -5.63929900 0.63594300 -1.42426200

H -0.88592300 0.98200300 -2.55780800

C -1.27140200 -1.06020600 -0.91461900

H -1.45062900 -1.67258600 -1.80363300

O -2.61423700 -3.45941000 -1.04446000

H -3.28846400 -4.15683400 -1.05087000

C -1.24228600 -2.03761200 0.30127900

C -1.20716600 -1.32228000 1.66469200

H -1.19278700 -2.08165500 2.45648700

H -2.08829700 -0.69624100 1.81382200

C -2.51164100 -2.91392700 0.26257000

H -3.39461200 -2.30735300 0.51398200

H -2.40720100 -3.69305900 1.02652400

H 0.01809300 -4.77324400 0.85143300

H -0.89539600 0.83945400 3.23450300

H 0.00325700 -0.52200000 3.95853300

O -0.01983000 3.00073000 1.22049400

C -0.02364600 3.67781200 0.09053600

O -0.01878600 2.99822900 -0.96403700

C -0.03321500 5.16903200 0.12923700

H -0.91446600 5.51400300 0.68171200

H 0.84386300 5.52527400 0.68122300

H -0.03604600 5.57996400 -0.88092700

[(L^1^)Fe^V^=O]^2+^ (^4^P_IS_)

Fe -0.00876800 1.16800900 0.19139900

N 1.96836800 1.09866000 -0.04947500

C 2.36776600 -0.00440300 -0.73656800

C 4.19055700 1.96662800 -0.11784500

H 4.88375800 2.75714900 0.14778400

C 2.85615900 2.06228700 0.25996200

H 2.46854000 2.90220300 0.82353200

C 3.68375000 -0.15620900 -1.15315400

H 3.96887100 -1.05088200 -1.69680100

C 4.60726900 0.84732300 -0.84299400

H 5.64076800 0.75448700 -1.16239200

N 0.00328000 -0.42826400 1.77014700

N 0.00226500 -0.31428900 -1.12633300

C -0.00224400 0.29254300 -2.50121200

H 0.87574100 0.92540500 -2.62539700

H 0.00370700 -0.50936500 -3.24678500

C 1.29836500 -1.05762800 -0.91044500

H 1.50032800 -1.66770900 -1.79511100

O 2.68812700 -3.41316800 -1.02330900

H 3.36126900 -4.11168300 -1.02235800

C 1.27267600 -2.02402600 0.31409000

C 1.21740800 -1.28240000 1.66253700

H 1.20827800 -2.02490200 2.47124100

H 2.09420500 -0.64793300 1.80488200

C 2.55289300 -2.88366000 0.28815700

H 3.42322600 -2.26941600 0.56318400

H 2.44350100 -3.67120400 1.04216000

C 0.02191300 -2.92697600 0.18419800

H 0.02573800 -3.42091900 -0.79288300

O 0.02978000 -3.87676500 1.23841100

C -0.00227900 0.31813200 3.05354900

H 0.87917800 0.96169200 3.09884400

N -1.98471800 1.06906900 -0.04940300

C -2.36756200 -0.03972000 -0.73669900

C -4.21956500 1.90391400 -0.11799700

H -4.92447500 2.68401100 0.14763100

C -2.88678700 2.01933800 0.26002500

H -2.51177800 2.86486700 0.82373900

C -3.68106300 -0.21095000 -1.15359500

H -3.95278300 -1.10955300 -1.69761900

C -4.61946500 0.77868200 -0.84343400

H -5.65140100 0.67057500 -1.16308100

H -0.88952300 0.91231300 -2.62538100

C -1.28258900 -1.07690500 -0.91049500

H -1.47539700 -1.68998100 -1.79514600

O -2.63540900 -3.45513000 -1.02212200

H -3.30024400 -4.16156900 -1.02132100

C -1.24231200 -2.04270800 0.31409500

C -1.19797900 -1.30038100 1.66254600

H -1.17761000 -2.04276900 2.47114200

H -2.08413900 -0.67908900 1.80506000

C -2.50939800 -2.92147300 0.28854800

H -3.38913200 -2.32006800 0.56200000

H -2.38892100 -3.70605300 1.04403200

H 0.02869400 -4.77285700 0.87138000

H -0.89316900 0.94857200 3.09880700

H 0.00280000 -0.37005300 3.90810500

O -0.02141200 2.85401500 1.14993100

C -0.02726400 3.61754100 0.09052000

O -0.02301600 3.03862200 -1.02661400

C -0.03827700 5.10452100 0.25966900

H -0.91927400 5.40482600 0.83733100

H 0.83946900 5.41800700 0.83529700

H -0.04300300 5.59601700 -0.71392900

[(L^1^)Fe^V^=O]^2+^ (^2^P_LS_)

Fe 0.00006100 -1.14800900 0.20778800

N -1.96569700 -1.07166300 -0.05527600

C -2.35888400 0.01951000 -0.75884700

C -4.18948800 -1.93845400 -0.13990600

H -4.88599100 -2.72431300 0.13093000

C -2.85882700 -2.03101200 0.25204700

H -2.48470200 -2.86543600 0.83244300

C -3.66926100 0.16818400 -1.19465900

H -3.94552800 1.05717200 -1.75202700

C -4.59849100 -0.82867300 -0.88348700

H -5.62848900 -0.73840300 -1.21450100

N -0.00004200 0.31719900 1.68972100

N -0.00002200 0.31186300 -1.13190700

C 0.00001700 -0.28553400 -2.50638300

H -0.88186000 -0.91228900 -2.63185500

H -0.00001100 0.51517900 -3.25324300

C -1.28309500 1.06661500 -0.92529000

H -1.47906000 1.68965600 -1.80194800

O -2.65318300 3.44874000 -0.96793000

H -3.32812800 4.14513700 -0.94651400

C -1.25646900 2.00119200 0.31828500

C -1.20304900 1.20645500 1.63268300

H -1.15247000 1.91174600 2.47051300

H -2.09076900 0.58899400 1.77131800

C -2.53106400 2.86805500 0.32237100

H -3.40676200 2.24865000 0.56740200

H -2.42171100 3.62439200 1.10807400

C -0.00017100 2.89939700 0.22202500

H -0.00019500 3.42839700 -0.73609600

O -0.00023900 3.80914800 1.31159900

C -0.00000600 -0.43516600 2.97693200

H -0.88562500 -1.07046200 3.02207500

N 1.96584900 -1.07145700 -0.05524700

C 2.35887500 0.01976200 -0.75884100

C 4.18976000 -1.93794500 -0.13993600

H 4.88637300 -2.72370900 0.13089000

C 2.85912400 -2.03067700 0.25206000

H 2.48513600 -2.86514900 0.83247500

C 3.66921900 0.16860400 -1.19470000

H 3.94534700 1.05762100 -1.75209100

C 4.59859400 -0.82812200 -0.88354600

H 5.62856900 -0.73772100 -1.21459400

H 0.88195300 -0.91221100 -2.63183700

C 1.28296700 1.06674800 -0.92527900

H 1.47887200 1.68981200 -1.80193400

O 2.65275600 3.44912600 -0.96784500

H 3.32768600 4.14553800 -0.94641600

C 1.25622400 2.00132700 0.31830000

C 1.20287000 1.20658800 1.63269700

H 1.15219400 1.91187600 2.47052400

H 2.09065500 0.58922600 1.77135200

C 2.53071000 2.86835200 0.32242500

H 3.40649000 2.24905800 0.56743400

H 2.42125900 3.62464000 1.10816400

H -0.00009200 4.71840800 0.97855100

H 0.88567400 -1.07037500 3.02207500

H -0.00004400 0.26152000 3.82263100

O 0.00025300 -2.80663900 1.25186100

C 0.00024500 -3.49014000 0.16143200

O 0.00013400 -2.77377100 -0.89786900

C 0.00028300 -4.97996100 0.13169000

H 0.87997500 -5.33430300 -0.41684000

H 0.00063500 -5.38517400 1.14470300

H -0.87985200 -5.33431000 -0.41614500

[(L^2^)Fe^V^=O]^2+^ (^6^P_HS_)

Fe 1.60635500 -0.13766900 0.40228900

N 1.23650100 -2.18051800 -0.03291200

C 0.12786600 -2.41071100 -0.77327400

C 1.82607700 -4.49238600 -0.16617300

H 2.51426900 -5.28901800 0.09422500

C 2.07294100 -3.19311200 0.26349900

H 2.94129300 -2.93305700 0.85914400

C -0.17550600 -3.68124400 -1.24437500

H -1.07009800 -3.84034900 -1.83714200

C 0.68630200 -4.73823800 -0.93484200

H 0.46999100 -5.74039500 -1.29162000

N -0.05573000 0.02774500 1.77863600

N 0.08765600 -0.00639200 -1.15951300

C 0.72861200 -0.07959800 -2.50656800

H 1.27840900 -1.01563000 -2.59485600

H -0.03531400 -0.01756800 -3.29073300

C -0.78293200 -1.21006300 -0.94894600

H -1.41656600 -1.34895100 -1.83220800

C -1.73439900 -1.08034600 0.28157800

C -1.01530100 -1.10768800 1.64312700

H -1.77173900 -1.03342800 2.43140500

H -0.48101900 -2.04383300 1.79168000

C -2.52324900 0.23197500 0.13621200

H -3.00352500 0.25686300 -0.84819000

O -3.47746200 0.26371500 1.17313000

C 0.62440000 -0.02068400 3.10441700

H 1.17058600 -0.96342200 3.19724700

N 1.61889100 1.92740200 -0.05792500

C 0.58675800 2.34714400 -0.82555300

C 2.68509300 4.05243800 -0.29434100

H 3.52337800 4.69937500 -0.06031200

C 2.64753500 2.75587900 0.20510900

H 3.43773400 2.34920700 0.82690700

C 0.56893100 3.62296700 -1.37216000

H -0.26485900 3.94052700 -1.98834300

C 1.63259500 4.48848400 -1.10202100

H 1.63756700 5.49134400 -1.51731700

H 1.44216600 0.73607000 -2.61429100

C -0.54386500 1.34317200 -0.97083600

H -1.13887200 1.58002100 -1.86289200

C -1.51376800 1.39621600 0.25936100

C -0.79596100 1.31245800 1.62129700

H -1.55554100 1.37399800 2.40952600

H -0.10601300 2.14354400 1.75274600

H -4.18088200 0.88501600 0.92334300

H 1.32810800 0.81269100 3.18195400

H -0.10848500 0.04996100 3.91680500

O 3.38381500 -0.30829800 1.16172400

C -2.69975900 -2.28387100 0.26148200

O -2.70054600 -3.14481000 1.10864300

O -3.48209100 -2.25327900 -0.82194200

C -2.21205600 2.77056000 0.22106600

O -1.69914700 3.77499200 0.65968600

O -3.40758800 2.71214100 -0.37555400

C -4.13203500 3.97545600 -0.47969900

H -5.06325600 3.72873500 -0.98622500

H -4.31639700 4.37642800 0.51868800

H -3.54483700 4.68935300 -1.06027500

C -4.47838200 -3.31199900 -0.91357200

H -5.15627900 -3.24998700 -0.06021800

H -5.00522900 -3.12970700 -1.84853600

H -3.98671100 -4.28711000 -0.91939000

C 4.04804500 -0.37483900 0.02477300

O 3.36078700 -0.30970300 -1.02250300

C 5.53204300 -0.51818800 0.05139800

H 5.96996200 0.32799700 0.59292900

H 5.80148300 -1.42213500 0.60918600

H 5.93215900 -0.56555900 -0.96197600

[(L^2^)Fe^V^=O]^2+^ (^4^P_IS_)

Fe 1.58947400 -0.05035200 0.18745400

N 1.40365500 -2.02091400 -0.01286500

C 0.28646000 -2.38359100 -0.70135100

C 2.12346800 -4.29536100 0.05865500

H 2.86070100 -5.02315600 0.37914600

C 2.30362300 -2.95039900 0.36153400

H 3.16086200 -2.58065800 0.91133300

C 0.05832700 -3.70736400 -1.04726000

H -0.83570500 -3.97616800 -1.59933800

C 0.99055300 -4.67714900 -0.66238000

H 0.82991000 -5.71893400 -0.92177100

N -0.10706000 0.02725200 1.76931500

N 0.07943600 -0.00018900 -1.15789400

C 0.68609400 -0.03596700 -2.53323300

H 1.28100900 -0.94196700 -2.64311000

H -0.11241300 -0.01435400 -3.28261000

C -0.71451600 -1.26782300 -0.93019400

H -1.31344400 -1.46611500 -1.82487400

C -1.69757100 -1.17735800 0.27775100

C -0.99700100 -1.15499400 1.64837300

H -1.76579400 -1.12540000 2.42876100

H -0.41302600 -2.06052500 1.80549400

C -2.55122300 0.09108700 0.10421800

H -3.00510500 0.08829300 -0.89354200

O -3.53283400 0.06716000 1.11418400

C 0.62472300 0.01483500 3.05943700

H 1.22927000 -0.89350900 3.11969700

N 1.55415100 1.92140800 -0.03956300

C 0.48034900 2.36149700 -0.75291800

C 2.50418200 4.11035200 -0.07157700

H 3.30999100 4.77424500 0.22138000

C 2.54186300 2.77186100 0.30081800

H 3.35440800 2.34320900 0.87502700

C 0.39924600 3.68011100 -1.17430300

H -0.45801800 4.01764900 -1.74477800

C 1.42514800 4.56630600 -0.83056900

H 1.37626200 5.60281900 -1.14912600

H 1.34797400 0.81969200 -2.66153500

C -0.61620500 1.32971300 -0.95138800

H -1.19412700 1.55555400 -1.85575700

C -1.61620400 1.31476300 0.25564300

C -0.91337700 1.26376000 1.62675900

H -1.68358000 1.28719200 2.40801200

H -0.27195600 2.13266800 1.76585100

H -4.26036800 0.65325800 0.84881300

H 1.28633000 0.88339300 3.10558200

H -0.07162400 0.04277200 3.90698600

O 3.35906000 -0.11902200 0.97916800

C -2.60034400 -2.42853000 0.24106300

O -2.57549300 -3.28721400 1.08956200

O -3.35819200 -2.44080300 -0.86091600

C -2.39880400 2.64282800 0.20294500

O -1.95928400 3.67780700 0.65014700

O -3.57657300 2.51054400 -0.41724700

C -4.37955800 3.72498000 -0.53132800

H -5.28496600 3.41950100 -1.05238500

H -4.60452300 4.11107800 0.46455600

H -3.83106900 4.47636000 -1.10239800

C -4.30344700 -3.54429300 -0.96759700

H -5.00386300 -3.50609700 -0.13121300

H -4.81498400 -3.39304200 -1.91647400

H -3.76903600 -4.49658100 -0.95314200

C 4.10277900 -0.15396000 -0.10567900

O 3.51500100 -0.13369900 -1.21049200

C 5.59013000 -0.21369600 0.05703900

H 5.93583500 0.66152400 0.61775800

H 5.86365300 -1.09662500 0.64460200

H 6.07413100 -0.24767100 -0.91971200

[(L^2^)Fe^V^=O]^2+^ (^2^P_LS_)

Fe -1.50437400 0.28938700 0.16687700

N -1.04499500 2.20350600 -0.06939800

C 0.09638400 2.38875100 -0.77788600

C -1.42210500 4.56152200 -0.04658200

H -2.03809300 5.39565900 0.27080700

C -1.79117600 3.26377800 0.29088200

H -2.67959900 3.04902900 0.87188100

C 0.51267000 3.65459500 -1.16457500

H 1.42813400 3.77248100 -1.73437700

C -0.25951400 4.75969000 -0.79351300

H 0.04664600 5.76086500 -1.08013000

N -0.07166800 -0.00920000 1.63735700

N -0.07562100 0.01429500 -1.19296200

C -0.67230900 0.14727200 -2.56146600

H -1.11518300 1.13645600 -2.66885000

H 0.10568700 0.00065300 -3.31834600

C 0.90995900 1.12850200 -0.97555100

H 1.56007700 1.21258900 -1.85236100

C 1.81084900 0.90232200 0.27246600

C 1.02962600 1.00222900 1.59198500

H 1.72099600 0.81744600 2.41983900

H 0.61259500 1.99564600 1.73702400

C 2.46354200 -0.48535500 0.15185100

H 2.96887000 -0.56420500 -0.81732100

O 3.37550800 -0.60865100 1.21874000

C -0.80571300 0.11948000 2.92978500

H -1.25737200 1.11048000 2.98833200

N -1.81299800 -1.64706800 -0.09951000

C -0.83951500 -2.24404500 -0.83158900

C -3.11240900 -3.64884700 -0.17536200

H -4.01293400 -4.17788000 0.11642400

C -2.92404700 -2.33215600 0.22873800

H -3.65050600 -1.80373300 0.83363300

C -0.97757900 -3.54550600 -1.29127800

H -0.18994000 -4.00068400 -1.88070900

C -2.13233500 -4.25931400 -0.95975700

H -2.25926200 -5.28105000 -1.30327200

H -1.46133000 -0.59267000 -2.68782200

C 0.40634600 -1.39834700 -0.99911500

H 0.97276700 -1.70625500 -1.88629900

C 1.33729100 -1.54113600 0.24800100

C 0.56729800 -1.35845300 1.56749600

H 1.27796800 -1.45039000 2.39612700

H -0.19145700 -2.12711500 1.69259800

H 4.01822800 -1.30144500 0.99485800

H -1.59685300 -0.63033600 2.96971900

H -0.11646800 -0.02274800 3.76922200

O -3.12487600 0.60839600 1.21333200

C 2.89397600 1.99921200 0.28989100

O 2.95704700 2.85261800 1.14209300

O 3.70047000 1.89095500 -0.77068800

C 1.89413300 -2.97850000 0.24545300

O 1.27121100 -3.92117900 0.67874900

O 3.10629600 -3.04708100 -0.31523300

C 3.70500500 -4.37718400 -0.38329700

H 4.67104100 -4.23018600 -0.86262200

H 3.81822000 -4.78080900 0.62456900

H 3.06801500 -5.03727600 -0.97492200

C 4.80538100 2.83885400 -0.82577800

H 5.44775900 2.70104700 0.04598200

H 5.33691600 2.60644900 -1.74685600

H 4.41994900 3.86055900 -0.83785500

C -3.79899200 0.75187900 0.12626400

O -3.09849500 0.61784600 -0.93562100

C -5.25975000 1.04280100 0.10409600

H -5.78537600 0.22656600 -0.40434100

H -5.64438900 1.15875500 1.11853500

H -5.44380600 1.95180300 -0.47858600

# Cartesian coordinates (O trans to N^7^ isomer)

[(L^1^)Fe^IV^=O]^1+^ (^1^R_LS_)

Fe 0.63282000 -1.05355100 -0.35780400

N -1.15057100 -1.93766900 -0.53004200

C -2.15252100 -1.10804700 -0.88498900

C -2.56089400 -3.82358900 -0.88246100

H -2.68432000 -4.90101200 -0.87345700

C -1.33402000 -3.26350600 -0.53332000

H -0.46793100 -3.84727500 -0.24643400

C -3.40175600 -1.59518300 -1.25637000

H -4.18439900 -0.89652600 -1.53339100

C -3.60714700 -2.97716800 -1.25160600

H -4.57057100 -3.38641900 -1.54005000

N -0.08044700 0.02781600 1.65747500

N -0.37120500 0.50347900 -1.18509100

C -0.30163000 0.39594500 -2.68201600

H -0.74233600 -0.54982700 -2.99228400

H -0.84430700 1.23624600 -3.13123900

C -1.81983500 0.36633200 -0.79347400

H -2.42160800 0.92667300 -1.51445800

O -4.26396400 1.57238300 -0.36937300

H -5.18676700 1.79598600 -0.17655500

C -2.16011800 0.93997300 0.60876600

C -1.55820600 0.09982600 1.74073600

H -1.85446500 0.54425400 2.70242700

H -1.94351400 -0.92205500 1.71442600

C -3.69040200 0.95907100 0.78208700

H -4.06203700 -0.06883400 0.90558500

H -3.91795200 1.52090500 1.69410400

C -1.59532700 2.37335500 0.68027200

H -1.97250000 2.95060600 -0.17106700

O -2.01754700 2.96305800 1.90463700

C 0.40280400 -0.72269400 2.84137600

H 0.01335700 -1.73939800 2.80492900

N 2.20108100 0.23750800 -0.56132000

C 1.81500200 1.48494900 -0.91337900

C 4.45778200 0.80241900 -1.12092100

H 5.49154700 0.48395100 -1.19707100

C 3.49250800 -0.10430900 -0.68738400

H 3.73976500 -1.11825400 -0.41510600

C 2.72038600 2.44155500 -1.36160200

H 2.35970900 3.42799300 -1.63489700

C 4.06991100 2.09723500 -1.45722300

H 4.79877300 2.82278500 -1.80541700

H 0.74140200 0.40670100 -2.99242600

C 0.33507300 1.77151600 -0.78919400

H 0.05567200 2.55409100 -1.50201500

O 0.08194600 4.53531800 -0.24187900

H 0.42549000 5.43175500 -0.10780400

C -0.05087500 2.29890900 0.61495500

C 0.46991400 1.40029400 1.74107700

H 0.18851800 1.85164300 2.70382200

H 1.55907000 1.32287800 1.70933600

C 0.55179700 3.69959400 0.81193500

H 1.64903200 3.63763800 0.81698600

H 0.23324600 4.07251000 1.79406600

H -2.17033200 3.90674400 1.75042000

H 1.49090900 -0.75900300 2.82256700

H 0.06356500 -0.23036800 3.76426100

O 1.08658600 -1.71838800 -1.76910200

O 1.12257200 -2.45314900 0.77508500

C 2.29231800 -2.68946500 1.34483000

O 3.16427100 -1.85366800 1.54404900

C 2.42823800 -4.14114300 1.77137000

H 1.56840100 -4.43832900 2.38070300

H 2.43798700 -4.78269300 0.88305400

H 3.35235300 -4.27861600 2.33459800

[(L^1^)Fe^IV^=O]^1+^ (^3^R_IS_)

Fe -1.17361000 0.16090900 -0.27139000

N -0.68167300 2.08937200 -0.43489600

C 0.56863000 2.30245200 -0.89505400

C -1.20596600 4.39376500 -0.72724800

H -1.93420400 5.19359500 -0.65007000

C -1.55735900 3.09982600 -0.35207300

H -2.53739900 2.82715600 0.01876700

C 0.98609600 3.56736700 -1.29636800

H 2.00210600 3.70283800 -1.65212800

C 0.08189600 4.62887500 -1.21111000

H 0.37920700 5.62549200 -1.52281100

N 0.15854400 -0.00178500 1.66038100

N 0.63237000 -0.10037500 -1.21446700

C 0.44864700 -0.09842500 -2.69689700

H 0.00041800 0.84537200 -3.00284900

H 1.41980800 -0.23586400 -3.18824400

C 1.47470200 1.09209300 -0.84859900

H 2.27285800 1.19641500 -1.58895600

O 3.98653900 2.18411200 -0.48778600

H 4.75865800 2.74358000 -0.31579500

C 2.16898200 0.99186600 0.54094700

C 1.17407800 1.07961500 1.70924300

H 1.73695600 1.01467200 2.65195800

H 0.64746900 2.03587100 1.70402300

C 3.17495700 2.15037300 0.68253700

H 2.63318100 3.09889300 0.81218600

H 3.77204800 1.96802600 1.58227600

C 2.90394400 -0.36097300 0.59176200

H 3.55505100 -0.44860300 -0.28454100

O 3.67297100 -0.41530600 1.78810600

C -0.71399900 0.11687600 2.85271000

H -1.22647000 1.07675000 2.83066300

N -1.21926700 -1.84834100 -0.38144400

C -0.05648400 -2.38444900 -0.82451800

C -2.29852800 -3.95832600 -0.67792600

H -3.21127600 -4.54059900 -0.61492500

C -2.32608700 -2.60939900 -0.32416500

H -3.23159200 -2.08202100 -0.03531100

C 0.03106100 -3.71560800 -1.21445400

H 0.97889600 -4.10216200 -1.57499600

C -1.10846200 -4.52034600 -1.13242000

H -1.06570000 -5.56259800 -1.43398900

H -0.23380300 -0.89952500 -2.97553700

C 1.13793100 -1.45730200 -0.80947500

H 1.87507700 -1.79179800 -1.54669600

O 3.45323100 -3.03231100 -0.32218000

H 3.92151900 -3.87226600 -0.20061400

C 1.84894700 -1.49134400 0.57152100

C 0.87296300 -1.29905900 1.73899100

H 1.44201700 -1.34595700 2.67858200

H 0.12666800 -2.09589000 1.76092000

C 2.54080000 -2.85238800 0.75602400

H 1.79062900 -3.65483900 0.78694300

H 3.05827500 -2.84219100 1.72423400

H 4.48831900 -0.90362000 1.60224400

H -1.47091200 -0.66718800 2.82120100

H -0.11737800 0.02464400 3.77155400

O -2.04378800 0.25657000 -1.63242900

O -2.56467400 0.65384300 0.91313700

C -3.84259600 0.33207400 0.91662500

O -4.36512800 -0.57029000 0.27543100

C -4.65484100 1.24112900 1.82986200

H -4.14525600 1.38727400 2.78737100

H -4.76278900 2.22638500 1.35992200

H -5.64740600 0.81735100 1.99003200

[(L^1^)Fe^IV^=O]^1+^ (^5^R_HS_)

Fe 1.01752 -0.66256 0.51812

N -0.35175 -2.30946 0.13804

C -1.34614 -2.01233 -0.71968

C -0.81285 -4.63851 -0.10876

H -0.57047 -5.66289 0.15254

C -0.07955 -3.58731 0.43582

H 0.75016 -3.7425 1.11702

C -2.12398 -3.007 -1.3071

H -2.92897 -2.72825 -1.97906

C -1.85086 -4.3405 -0.99399

H -2.43844 -5.13725 -1.44002

N -0.60402 0.31056 1.84434

N -0.24148 0.13334 -1.04893

C 0.33213 -0.20947 -2.38245

H 0.40364 -1.29129 -2.47798

H -0.30502 0.20124 -3.17626

C -1.58338 -0.53057 -0.93294

H -2.12922 -0.38149 -1.8699

O -4.32826 -0.43529 -1.26986

H -5.28062 -0.60914 -1.30818

C -2.48635 0.0432 0.19846

C -1.95464 -0.26004 1.60834

H -2.65656 0.16418 2.34036

H -1.89715 -1.335 1.78586

C -3.89134 -0.57863 0.07833

H -3.85414 -1.63937 0.36824

H -4.55366 -0.05253 0.77425

C -2.56212 1.56843 0.00019

H -2.88915 1.77981 -1.02298

O -3.49076 2.10424 0.93662

C -0.21053 0.04183 3.24858

H -0.15495 -1.03579 3.40377

N 1.89327 1.28256 0.18261

C 1.20708 2.05231 -0.6862

C 3.80561 2.68841 -0.05899

H 4.83661 2.89812 0.20284

C 3.16478 1.57664 0.48593

H 3.66037 0.88925 1.15926

C 1.782 3.1711 -1.27804

H 1.20164 3.7718 -1.97095

C 3.10435 3.49329 -0.9546

H 3.5808 4.35792 -1.40677

H 1.34074 0.19174 -2.45607

C -0.23078 1.6297 -0.90846

H -0.6019 2.07356 -1.83898

O -1.71134 4.05378 -1.12785

H -1.76507 5.01997 -1.18378

C -1.15135 2.15892 0.22786

C -0.66733 1.77917 1.63634

H -1.3675 2.21062 2.36533

H 0.3243 2.18732 1.8374

C -1.21178 3.69451 0.15614

H -0.21303 4.11833 0.33219

H -1.87289 4.05009 0.95773

H -3.93523 2.8583 0.52204

H 0.78006 0.45897 3.42882

H -0.93865 0.48485 3.94209

O 1.84074 -1.15215 1.82515

O 2.17704 -1.40885 -0.7347

C 3.46299 -1.70544 -0.47116

O 4.34942 -0.8834 -0.59967

C 3.68392 -3.12901 -0.01192

H 3.30511 -3.20967 1.01405

H 3.12059 -3.82752 -0.63856

H 4.7469 -3.3749 -0.02581

[(L^2^)Fe^IV^=O]^1+^ (^1^R_LS_)

Fe 1.32603800 -0.92228600 -0.36433400

N 0.03693900 -2.44832700 -0.53605400

C -1.19384800 -2.09773700 -0.95852400

C -0.52459600 -4.75136600 -0.76449300

H -0.22369100 -5.78953400 -0.67693400

C 0.37891800 -3.73879600 -0.44759000

H 1.39157000 -3.92265500 -0.11047900

C -2.14453700 -3.05142500 -1.30322100

H -3.12718600 -2.74076700 -1.64224000

C -1.80279300 -4.40231300 -1.19939200

H -2.52513500 -5.16925300 -1.46091700

N 0.17295500 -0.13913400 1.55987500

N -0.18075500 0.08267400 -1.30607100

C -0.00685900 -0.04531600 -2.79271000

H -0.02389400 -1.09981200 -3.06219600

H -0.81588700 0.49289600 -3.30181500

C -1.46923700 -0.60773500 -0.94345200

H -2.21821600 -0.35626900 -1.70160600

O -4.27222600 -0.29610800 -0.40248500

C -2.04584900 -0.16948000 0.43129900

C -1.21114000 -0.66470300 1.61684700

H -1.70510900 -0.34275100 2.54188900

H -1.17225300 -1.75347000 1.63238500

C -2.12115800 1.36635500 0.44038000

H -2.65684100 1.70325700 -0.45501000

O -2.80950800 1.75527200 1.61230200

C 0.87415800 -0.58803100 2.78908500

H 0.91408600 -1.67622300 2.80054900

N 2.26026000 0.87472900 -0.58450100

C 1.43658500 1.85622000 -1.01874800

C 4.14075500 2.26880100 -1.07919000

H 5.21883700 2.38447800 -1.09108500

C 3.58669400 1.06898800 -0.63689400

H 4.19552300 0.24718500 -0.29467700

C 1.91740700 3.07378300 -1.48339300

H 1.22524300 3.83673100 -1.82284800

C 3.29609700 3.29021200 -1.50379300

H 3.69793900 4.23445300 -1.85766400

H 0.96105800 0.36459100 -3.07431700

C -0.04370500 1.53976100 -0.95681800

H -0.57755900 2.12304000 -1.71837200

O -1.76002000 3.99624000 0.01419800

C -0.67335100 1.90520400 0.41793800

C 0.12486000 1.33979900 1.59798600

H -0.35594700 1.67565800 2.52693000

H 1.14353000 1.72515700 1.59127400

H -3.11294700 2.66860000 1.48658800

H 1.88887900 -0.19285800 2.78610500

H 0.33732400 -0.22987000 3.67857000

O 2.05401400 -1.39687600 -1.73817700

O 2.27333600 -1.99011100 0.83230600

C 3.42635800 -1.75961100 1.44007000

O 3.92775100 -0.65724100 1.61173000

C 4.05917000 -3.04381100 1.94779200

H 3.34141800 -3.60497700 2.55512500

H 4.33493800 -3.67620800 1.09644900

H 4.94768400 -2.81190700 2.53667200

C -3.46082700 -0.75513100 0.56831300

O -3.79047300 -1.54305600 1.42180800

C -0.64858900 3.43634900 0.53523500

O 0.26770100 4.06481300 1.00981600

C -1.80185000 5.44606400 0.04645300

H -2.75547000 5.71748200 -0.40432100

H -1.73819300 5.79961500 1.07792200

H -0.96746000 5.85671800 -0.52663900

C -5.64585100 -0.74750000 -0.32794200

H -6.14965000 -0.28508200 -1.17585200

H -5.68911900 -1.83754000 -0.39279600

H -6.09171500 -0.42578600 0.61583400

[(L^2^)Fe^IV^=O]^1+^ (^3^R_IS_)

Fe 1.57118700 0.05856500 -0.28863400

N 1.41740900 -1.93876000 -0.39906400

C 0.22390800 -2.36170800 -0.87911100

C 2.25778300 -4.16878900 -0.54893700

H 3.09274800 -4.84729000 -0.41312800

C 2.42598800 -2.81543800 -0.25584300

H 3.37327000 -2.38151000 0.05130700

C -0.00231500 -3.69142500 -1.20868100

H -0.97025400 -3.99308800 -1.59504600

C 1.03072900 -4.61545800 -1.03084200

H 0.87784500 -5.66131200 -1.27853600

N 0.14779200 0.02594600 1.57493300

N -0.21585300 -0.01325700 -1.32164900

C 0.03728700 -0.03639800 -2.79375600

H 0.64833800 -0.90368500 -3.03745000

H -0.91796100 -0.07860200 -3.33241900

C -0.86651900 -1.31330000 -0.94037800

H -1.59639500 -1.57845900 -1.71268900

C -1.63185900 -1.27126200 0.41331700

C -0.69689800 -1.19237600 1.62606700

H -1.31100700 -1.19185000 2.53478400

H -0.05292900 -2.06926400 1.67277000

C -2.57454100 -0.05917200 0.38461300

H -3.18068800 -0.09862800 -0.52801100

O -3.39417500 -0.12105000 1.53511600

C 0.97511700 0.05844000 2.80613500

H 1.64728700 -0.79961200 2.80629100

N 1.28112100 2.02889500 -0.44854100

C 0.09262500 2.37351400 -0.98633800

C 2.06129000 4.26624300 -0.68580100

H 2.86083000 4.98603300 -0.54984000

C 2.24898500 2.94185800 -0.29874000

H 3.16822800 2.56721600 0.13349200

C -0.15702000 3.67187000 -1.41257400

H -1.11798200 3.92803400 -1.84496600

C 0.84433000 4.63354800 -1.25895800

H 0.67341600 5.65501900 -1.58380400

H 0.59391200 0.85528800 -3.07681700

C -0.94007700 1.26360600 -0.99093200

H -1.69306100 1.45192700 -1.76728000

C -1.69505400 1.20995700 0.37568200

C -0.75010400 1.20457000 1.58880400

H -1.36807100 1.19284800 2.49695200

H -0.14935100 2.11194800 1.61116100

H -4.16882700 0.44160100 1.37642200

H 1.58157600 0.96202800 2.80941900

H 0.33070700 0.02849700 3.69567500

O 2.49598100 0.07690700 -1.61689400

O 2.95462800 0.38345300 0.95034400

C -2.46045900 -2.56056600 0.54206600

O -2.30335900 -3.38422700 1.41099600

O -3.36286000 -2.65959100 -0.45096100

C -2.55284600 2.48037800 0.46968900

O -2.17028400 3.52173700 0.95034300

O -3.77124300 2.30416900 -0.07937800

C -4.63569900 3.46949400 -0.07123100

H -5.56161700 3.14513400 -0.54410400

H -4.81104100 3.79810300 0.95550500

H -4.17240300 4.28203200 -0.63545900

C -4.22824100 -3.81873600 -0.38946200

H -4.80362300 -3.80398500 0.53883800

H -4.88277400 -3.73841000 -1.25647800

H -3.63442200 -4.73519800 -0.42998800

C 4.20115100 -0.05009200 0.96338600

O 4.65621000 -0.96067500 0.28470100

C 5.06283400 0.73529400 1.94272900

H 5.25706900 1.73585400 1.53747100

H 4.54629800 0.86246000 2.89918100

H 6.01516600 0.22431800 2.09194300

[(L^2^)Fe^IV^=O]^1+^ (^5^R_HS_)

Fe -1.22775 -1.01869 0.68297

N -2.26583 0.79208 0.4183

C -1.72819 1.62309 -0.49559

C -4.27824 2.06863 0.42382

H -5.28329 2.20964 0.80506

C -3.51237 0.99294 0.86402

H -3.87716 0.26162 1.57633

C -2.43728 2.70607 -0.99687

H -1.98305 3.35914 -1.73471

C -3.73448 2.93069 -0.52624

H -4.31237 3.76878 -0.90318

N 0.38375 0.15584 1.91084

N -0.12472 -0.15393 -0.96323

C -0.7373 -0.57234 -2.25972

H -1.77978 -0.25668 -2.28088

H -0.17944 -0.12405 -3.09261

C -0.28064 1.33476 -0.84008

H -0.03077 1.7944 -1.80283

O 0.94986 3.91714 -1.12776

C 0.6588 1.97874 0.224

C 0.30052 1.61711 1.67373

H 0.99607 2.1446 2.33762

H -0.70376 1.95572 1.92032

C 0.5528 3.50721 0.09006

C 2.09408 1.53068 -0.09255

H 2.31073 1.74182 -1.14605

O 2.96987 2.24982 0.75355

C 0.09035 -0.11638 3.33859

H -0.92248 0.21655 3.56447

N 0.26055 -2.51576 0.32977

C 1.16495 -2.19054 -0.61376

C 0.87116 -4.81375 0.15347

H 0.72704 -5.83789 0.47962

C 0.10164 -3.79184 0.70412

H -0.66915 -3.96799 1.44656

C 1.95753 -3.1573 -1.2211

H 2.67838 -2.87522 -1.98112

C 1.80959 -4.48912 -0.82646

H 2.41804 -5.26325 -1.28358

H -0.71462 -1.65801 -2.33038

C 1.27165 -0.70336 -0.90076

H 1.75228 -0.54934 -1.87678

O 4.30781 0.23213 -0.88503

C 2.16822 0.00949 0.16294

C 1.76195 -0.29654 1.61457

H 2.46785 0.223 2.27662

H 1.83793 -1.36236 1.81919

C 3.59577 -0.52421 -0.02637

H 3.85949 2.1962 0.36928

H 0.14195 -1.19112 3.51466

H 0.80988 0.40559 3.98408

O -1.94474 -1.51409 2.04475

O -2.30452 -2.10884 -0.48331

O 4.01853 -1.52343 0.50772

O 0.14255 4.23775 0.96006

C -3.4767 -1.89489 -1.05592

O -3.90133 -0.78859 -1.37262

C -4.24662 -3.17661 -1.31593

H -3.61241 -3.8976 -1.84196

H -4.52538 -3.62831 -0.35716

H -5.14393 -2.96774 -1.90062

C 5.65667 -0.22949 -1.15434

H 6.07297 0.49052 -1.85751

H 6.23489 -0.25593 -0.22811

H 5.62827 -1.2306 -1.59031

C 0.92298 5.34889 -1.3394

H 1.5849 5.84302 -0.62473

H 1.27029 5.49759 -2.36112

H -0.0933 5.72957 -1.21132

[(L^1^)Fe^IV^=O]^1+^ (^5^TS1_HS_)

Fe -0.71859 0.3435 -0.11338

N -0.99757 -1.77305 0.49099

C 0.05959 -2.2626 1.16684

C -2.33063 -3.60137 1.25503

H -3.29229 -4.10308 1.25643

C -2.1717 -2.42111 0.52869

H -2.98663 -1.9959 -0.04927

C -0.02213 -3.42982 1.92011

H 0.8558 -3.80035 2.43973

C -1.24289 -4.10924 1.96447

H -1.33807 -5.02285 2.5436

N 0.63623 -0.31955 -1.78811

N 1.02035 -0.02362 1.10078

C 0.72972 0.26647 2.53288

H -0.10541 -0.34677 2.86347

H 1.62007 0.0618 3.14139

C 1.34555 -1.48638 0.97726

H 2.05432 -1.75877 1.76749

O 3.28471 -3.60081 0.8545

C 2.03158 -1.86847 -0.36551

C 1.1189 -1.71397 -1.5904

H 1.68681 -2.01774 -2.4811

H 0.24395 -2.3606 -1.51438

C 2.47814 -3.34985 -0.2885

C 3.25731 -0.95526 -0.50874

H 3.84768 -1.00965 0.41397

O 4.03756 -1.40363 -1.60931

C -0.13263 -0.26355 -3.05741

H -1.0009 -0.91663 -2.97652

N 0.25895 2.31354 -0.06482

C 1.47213 2.2989 0.51997

C 0.2323 4.69825 0.02885

H -0.28967 5.62536 -0.18095

C -0.35609 3.47947 -0.31052

H -1.34204 3.40359 -0.74866

C 2.11322 3.47139 0.90571

H 3.08683 3.42404 1.38324

C 1.4782 4.69213 0.6538

H 1.95252 5.62418 0.94632

H 0.43638 1.30938 2.63735

C 2.09344 0.92322 0.64149

H 2.89167 0.94606 1.39292

O 4.91607 1.23798 0.04571

C 2.76265 0.4969 -0.69836

C 1.82196 0.56688 -1.91261

H 2.39427 0.25934 -2.79899

H 1.46411 1.58427 -2.07802

C 3.95891 1.41903 -0.99321

H 4.93063 -1.03972 -1.50415

H -0.48955 0.75368 -3.21609

H 0.50094 -0.5782 -3.89788

O -1.96042 0.62477 -1.27048

O -1.75567 0.75379 1.42625

H -3.07178 0.69983 -0.90561

C -4.40657 0.54606 -0.63945

H -4.79286 1.55061 -0.78751

H -4.38314 0.1914 0.38716

C -4.78078 -0.49228 -1.6161

O -4.6003 -1.69102 -1.45426

O -5.29811 0.01778 -2.75305

H -5.48676 -0.73676 -3.34294

C -2.58401 1.75672 1.66227

O -2.82444 2.6774 0.89289

C -3.23251 1.64595 3.03386

H -3.80511 0.71389 3.09797

H -2.46296 1.605 3.81222

H -3.89147 2.49806 3.20674

H 4.37667 1.14411 -1.97194

H 1.59882 -3.99575 -0.20452

H 2.99897 -3.60724 -1.21567

H 4.21408 -3.49741 0.60236

H 5.6592 1.84251 -0.10304

H 3.62543 2.46338 -1.05954

[(L^1^)Fe^IV^=O]^1+^ (^3^TS1_IS_)

Fe -0.6969900 0.42190000 0.03639000

N 0.3801000 2.36963000 -0.37452000

C 1.5566000 2.18792000 -0.99752000

C 0.3982700 4.67781000 -0.99328000

H -0.0918300 5.64546000 -0.97263000

C -0.1938000 3.57989000 -0.37123000

H -1.1463800 3.64392000 0.14549000

C 2.2145100 3.23353000 -1.64457000

H 3.1710700 3.05556000 -2.12487000

C 1.6215700 4.49814000 -1.64025000

H 2.1079000 5.33078000 -2.13986000

N 0.7246800 -0.01093000 1.74333000

N 1.0111500 -0.19652000 -1.15840000

C 0.6776400 -0.12109000 -2.61038000

H 0.3952300 0.89766000 -2.87137000

H 1.5480500 -0.42831000 -3.20532000

C 2.1239500 0.78160000 -0.89238000

H 2.8912400 0.64986000 -1.66293000

O 4.8368900 1.33161000 -0.62190000

H 5.7053400 1.73801000 -0.48146000

C 2.8376900 0.56414000 0.47566000

C 1.9378900 0.85554000 1.68915000

H 2.5279700 0.68325000 2.60024000

H 1.6064000 1.89503000 1.69825000

C 4.0598500 1.50176000 0.55943000

H 3.7249100 2.54458000 0.66060000

H 4.6314400 1.23273000 1.45395000

C 3.2972300 -0.90655000 0.52580000

H 3.8944400 -1.12392000 -0.36480000

O 4.0817800 -1.10299000 1.69761000

C 0.0356300 0.25017000 3.03486000

H -0.2705400 1.29541000 3.07487000

N -1.0156300 -1.86296000 -0.22923000

C 0.0214000 -2.41827000 -0.88327000

C -2.3790700 -3.73964000 -0.81860000

H -3.3506100 -4.21988000 -0.77685000

C -2.1931900 -2.50384000 -0.19747000

H -2.98773000 -2.00040000 0.3389700

C -0.08356000 -3.64815000 -1.5298700

H 0.77579000 -4.06507000 -2.0461600

C -1.30890000 -4.31955000 -1.4955500

H -1.42332000 -5.27551000 -1.9982000

H -0.16712000 -0.77214000 -2.8286400

C 1.31955000 -1.63081000 -0.8280100

H 2.00492000 -2.02325000 -1.5891400

O 3.36077000 -3.60730000 -0.3404100

H 3.61387000 -4.53909000 -0.2530000

C 2.03887000 -1.80718000 0.5398500

C 1.16705000 -1.43496000 1.7498200

H 1.76447000 -1.60217000 2.6566600

H 0.27603000 -2.06071000 1.8098300

C 2.45868000 -3.27945000 0.7115400

H 1.57332000 -3.92916000 0.6956700

H 2.93833000 -3.38959000 1.6933100

H 4.75514000 -1.77075000 1.4982600

H -0.84621000 -0.38681000 3.1069700

H 0.71421000 0.03561000 3.8714800

O -1.66513000 0.57831000 -1.3946300

O -1.89222000 1.39166000 1.1453900

C -2.82377000 0.82537000 1.8919400

O -3.07117000 -0.37674000 1.8911600

C -3.58821000 1.81698000 2.7514000

H -4.38087000 2.26490000 2.1423200

H -4.05108000 1.29588000 3.5920200

H -2.94006000 2.62038000 3.1115800

H -2.86184000 0.89511000 -1.3212900

C -4.09230000 1.19061000 -1.5415000

H -4.12333000 1.04140000 -2.6207700

H -4.20977000 2.22537000 -1.2242800

C -4.89162000 0.18082000 -0.7915200

O -4.95647000 -0.99868000 -1.0721900

O -5.52722000 0.72575000 0.2706800

H -5.95933000 -0.00995000 0.7464100

[(L^2^)Fe^IV^=O]^1+^ (^5^TS1_HS_)

Fe -0.98131 -0.84884 -0.04974

N -1.74894 1.16985 -0.69899

C -0.81004 1.88078 -1.35911

C -3.4145 2.71703 -1.44762

H -4.45664 3.01679 -1.44764

C -3.02546 1.57807 -0.74023

H -3.73089 0.98175 -0.18417

C -1.11931 3.01669 -2.09728

H -0.33455 3.55874 -2.61508

C -2.45063 3.44237 -2.14123

H -2.72345 4.3277 -2.70755

N 0.16906 0.17196 1.61934

N 0.64118 -0.08475 -1.24701

C 0.44795 -0.45501 -2.67951

H -0.48855 -0.03079 -3.03443

H 1.2872 -0.07266 -3.27516

C 0.61856 1.41125 -1.15951

H 1.25315 1.81545 -1.95671

O 2.0722 3.86346 -0.93349

C 1.17604 1.9612 0.18273

C 0.30214 1.63675 1.40085

H 0.76586 2.09833 2.27853

H -0.69465 2.05828 1.29283

C 1.261 3.49413 0.07414

C 2.58824 1.38822 0.40355

H 3.20808 1.58597 -0.48458

O 3.11431 2.02811 1.53902

C -0.59164 -0.02628 2.88063

H -1.55801 0.4652 2.77827

N 0.4454 -2.52255 -0.03562

C 1.61219 -2.23876 -0.64165

C 0.87013 -4.85892 -0.25446

H 0.54541 -5.88008 -0.08621

C 0.07054 -3.79305 0.15375

H -0.89556 -3.92095 0.63106

C 2.45857 -3.24735 -1.09394

H 3.38873 -2.99572 -1.5883

C 2.07817 -4.57723 -0.89339

H 2.71729 -5.38353 -1.24059

H 0.37868 -1.53627 -2.77142

C 1.90371 -0.74943 -0.76735

H 2.68231 -0.59202 -1.52099

O 4.48282 -1.22094 -0.12502

C 2.43085 -0.15988 0.5716

C 1.51632 -0.43001 1.78417

H 1.99952 -0.00538 2.67021

H 1.38987 -1.49989 1.95003

C 3.83517 -0.67382 0.90841

H 3.83216 1.49265 1.92311

H -0.75285 -1.09024 3.04453

H -0.04029 0.41384 3.72228

O -1.99246 -1.65779 1.09132

O -1.7401 -1.65657 -1.58052

H -3.11872 -1.44331 1.26209

C -4.38902 -1.20092 1.7544

H -4.66326 -2.12842 2.25118

H -4.87027 -0.99468 0.80386

C -4.13679 -0.0358 2.61423

O -3.88783 1.09118 2.20701

O -4.11977 -0.3464 3.93077

H -3.94267 0.48282 4.41452

C -3.02602 -1.76492 -1.8691

O -3.92816 -1.20219 -1.26506

C -3.27345 -2.66715 -3.06596

H -2.92505 -3.68054 -2.83826

H -4.33719 -2.68721 -3.30683

H -2.70113 -2.31229 -3.92969

O 0.66231 4.25971 0.7903

O 4.3408 -0.49697 2.00155

C 2.25757 5.29069 -1.08626

H 2.69345 5.70823 -0.17599

H 2.9348 5.40634 -1.93168

H 1.29803 5.77559 -1.28222

C 5.85293 -1.62592 0.1328

H 6.2127 -2.03436 -0.81054

H 6.44592 -0.76091 0.43668

H 5.87544 -2.37916 0.92297

[(L^2^)Fe^IV^=O]^1+^ (^3^TS1_IS_)

Fe 0.96557300 -0.51406400 -0.06498700

N 0.14854600 -2.33970600 -0.35604500

C -1.05841300 -2.32404300 -0.95681400

C 0.26535000 -4.70593600 -0.62549400

H 0.81922500 -5.62634900 -0.47617100

C 0.80638700 -3.49620100 -0.19569600

H 1.77290800 -3.40968400 0.28557100

C -1.65238200 -3.49117200 -1.42622600

H -2.61952400 -3.45061900 -1.91635900

C -0.97855400 -4.70278400 -1.25435700

H -1.41895800 -5.62807100 -1.61290600

N -0.47860500 -0.07024400 1.63516100

N -0.63048000 0.04382600 -1.27468500

C -0.30003300 -0.03315200 -2.73112500

H 0.01029700 -1.04491500 -2.98427500

H -1.18389900 0.25032400 -3.31742500

C -1.71928800 -0.96093000 -1.00236300

H -2.44505100 -0.92524900 -1.82228900

O -4.55764700 -1.40541400 -0.68039300

C -2.50875500 -0.69084600 0.31397400

C -1.68139100 -0.94245500 1.58487300

H -2.31903200 -0.74653700 2.45527400

H -1.36664900 -1.98337400 1.64491700

C -2.98932900 0.77103400 0.26933000

H -3.50660900 0.94792000 -0.68040400

O -3.86965500 0.97116400 1.35811700

C 0.21078500 -0.30718200 2.93159000

H 0.51074000 -1.35222600 2.99338200

N 1.40284300 1.49564100 -0.18288200

C 0.43839400 2.17971400 -0.85340500

C 2.86454200 3.38092100 -0.42081200

H 3.84455700 3.80200700 -0.22925000

C 2.58344200 2.09527400 0.04524800

H 3.29081900 1.52389500 0.63115400

C 0.65433600 3.45783100 -1.34886000

H -0.13469400 3.97054500 -1.88953300

C 1.89434100 4.06909400 -1.13732400

H 2.08735000 5.06437600 -1.52584800

H 0.52698800 0.63783400 -2.95299000

C -0.90606700 1.48599200 -0.94814100

H -1.49205800 1.92392500 -1.76698200

O -3.29655700 3.41035600 -0.19359200

C -1.73919400 1.67603500 0.36157700

C -0.94184400 1.34123500 1.63179000

H -1.59585000 1.50797800 2.49789100

H -0.08292500 2.00051500 1.73692700

H -4.40122100 1.76059200 1.16664800

H 1.10014700 0.32124000 2.98903400

H -0.46681700 -0.06859100 3.76253300

O 1.93924200 -0.86960000 -1.45831500

O 2.16805100 -1.36083500 1.15084400

C 3.18978900 -0.98610500 1.88406900

O 3.58182600 0.16907200 2.03618000

C 3.89177600 -2.15569600 2.55951700

H 4.50782500 -1.79425000 3.38526500

H 3.17520200 -2.89807400 2.92295700

H 4.54040600 -2.64452400 1.82309700

H 3.07827800 -0.57779100 -1.50451000

C 4.36430200 -0.39593500 -1.96302300

H 4.20819700 0.30398600 -2.78117600

H 4.58349100 -1.41833600 -2.26850500

C 5.20488400 0.14775800 -0.87629800

O 5.43381700 1.33141500 -0.70139100

O 5.68347800 -0.81793100 -0.05693200

H 6.13914100 -0.35556000 0.67283500

C -2.14031400 3.15990600 0.45622400

O -1.49317100 4.00255200 1.03166900

C -3.73280800 -1.62677000 0.35948600

O -3.91803000 -2.45974600 1.21438300

C -3.73559400 4.79274800 -0.18214300

H -4.66866500 4.80303300 -0.74381200

H -3.89143900 5.12816900 0.84513600

H -2.98654200 5.42752400 -0.65963000

C -5.77840600 -2.18319500 -0.68556000

H -6.31091400 -1.87952400 -1.58597800

H -5.54780600 -3.25047500 -0.70870100

H -6.36452800 -1.95978200 0.20818000

[(L^1^)Fe^IV^=O]^1+^ (^1^INT_LS_)

Fe -1.08225800 0.31193300 -0.15997800

N -0.40333900 2.16257000 -0.34705400

C 0.84353300 2.24379000 -0.85491600

C -0.69087300 4.51022900 -0.63154900

H -1.32715100 5.38276400 -0.53207800

C -1.16356900 3.25989500 -0.24123200

H -2.15597500 3.09690400 0.15977300

C 1.37932600 3.45621200 -1.27557800

H 2.38841700 3.48320900 -1.67343900

C 0.59694500 4.60812300 -1.16029500

H 0.98678000 5.56786700 -1.48546000

N 0.20935000 -0.02344800 1.67159000

N 0.60785600 -0.13461600 -1.20488300

C 0.37439300 -0.08083800 -2.67844900

H 0.01904500 0.91058900 -2.95101600

H 1.30862400 -0.31609000 -3.20376100

C 1.60273900 0.93529000 -0.85024000

H 2.38975800 0.95574400 -1.60953500

O 4.25249200 1.67723000 -0.52426400

H 5.08165700 2.15319400 -0.36720600

C 2.30876400 0.72609700 0.52001000

C 1.35765600 0.92011900 1.71162800

H 1.92520400 0.76451100 2.64022900

H 0.95747300 1.93511900 1.73373100

C 3.45456500 1.74698300 0.65336900

H 3.03753000 2.75622000 0.78683000

H 4.03245700 1.49037000 1.54787300

C 2.86784900 -0.70898200 0.53823300

H 3.48516700 -0.86444400 -0.35264000

O 3.64682100 -0.88002500 1.71769700

C -0.61391600 0.18853700 2.88724200

H -1.01485300 1.20054600 2.87864100

N -1.42359800 -1.67455100 -0.31997300

C -0.35256800 -2.32814500 -0.83283100

C -2.75001500 -3.64469300 -0.61531100

H -3.71849100 -4.12311900 -0.51912300

C -2.60041100 -2.31636800 -0.22058500

H -3.43410100 -1.72308400 0.13131600

C -0.43674800 -3.64786000 -1.26381000

H 0.44404000 -4.12908900 -1.67665100

C -1.65518300 -4.32076300 -1.14927100

H -1.74705300 -5.35018800 -1.48198500

H -0.40546100 -0.79072900 -2.94480500

C 0.94814300 -1.55151900 -0.83791200

H 1.62043500 -1.96318600 -1.59768700

O 3.05598900 -3.41469600 -0.42332900

H 3.42218700 -4.30612600 -0.31947300

C 1.67882400 -1.69810600 0.52637700

C 0.76115700 -1.40171500 1.72032000

H 1.34314300 -1.52559700 2.64429700

H -0.07667200 -2.10044500 1.75681400

C 2.19730100 -3.13786300 0.67805500

H 1.35259100 -3.84018900 0.71576500

H 2.73293800 -3.20765500 1.63386500

H 4.40172200 -1.44696000 1.50256200

H -1.45574100 -0.50327300 2.87266100

H -0.00768000 0.02567900 3.78926600

O -2.02896100 0.60787600 -1.63398200

O -2.50323800 0.83572500 1.01662800

C -3.78946900 0.73355500 0.82337100

O -4.31966400 0.19840100 -0.15943400

C -4.62137600 1.34528600 1.93760100

H -4.32771300 0.92165600 2.90391000

H -4.43472700 2.42407000 1.98766400

H -5.68261300 1.16817100 1.75799300

H -2.94399000 0.31393300 -1.41854800

C 4.44033724 -1.21670688 3.02836743

H 3.94544924 -2.15381388 2.81333543

H 4.39488224 -0.79621188 4.02687543

C 5.29860224 -0.60917788 2.00764643

O 5.06004924 -0.69104588 0.79936043

O 6.32746724 0.05730812 2.51947243

H 6.88091624 0.41207912 1.79468243

C -4.50382680 1.35425538 -4.39550692

H -3.58039807 1.91077883 -4.31254024

H -4.51190471 0.38741068 -4.88647179

C -5.69927930 1.84276510 -3.70280695

O -5.94387950 3.04403975 -3.55901922

O -6.49521772 0.86581982 -3.28233988

H -7.25670059 1.24811664 -2.80121433

[(L^1^)Fe^IV^=O]^1+^ (^3^INT_IS_)

Fe -1.08225800 0.31193300 -0.15997800

N 0.07858300 2.33391500 -0.41749000

C 1.28935100 2.08102300 -0.94005800

C 0.29558100 4.63960600 -0.99376500

H -0.13301900 5.63594300 -1.00418200

C -0.42004700 3.57543800 -0.44917200

H -1.41394200 3.68899400 -0.02875900

C 2.06953500 3.08745300 -1.50410100

H 3.05084400 2.84746300 -1.90003900

C 1.56125600 4.38838400 -1.52728600

H 2.14322700 5.19424200 -1.96435300

N 0.11189700 -0.03097800 1.71717700

N 0.57763300 -0.23863300 -1.16080300

C 0.30654400 -0.12518300 -2.61894300

H 0.09872400 0.91366400 -2.86783100

H 1.16924800 -0.49100000 -3.19172700

C 1.74221900 0.63760700 -0.82019500

H 2.54627600 0.45293200 -1.54065800

O 4.46190000 0.95896500 -0.36818800

H 5.34041400 1.32459400 -0.18577000

C 2.34111600 0.36675000 0.59067600

C 1.39089900 0.73470400 1.74365300

H 1.90794700 0.52451000 2.69012700

H 1.13946500 1.79582600 1.72550200

C 3.62411100 1.20341700 0.75750200

H 3.36453800 2.27000800 0.83073700

H 4.10849900 0.89898200 1.69202700

C 2.67432700 -1.13432100 0.66848100

H 3.31323900 -1.40376200 -0.17816900

O 3.34559700 -1.39475900 1.89794600

C -0.64921100 0.29151100 2.95192400

H -0.88394000 1.35444900 2.96289700

N -1.64956000 -1.61368000 -0.39420800

C -0.63841800 -2.33184900 -0.91580800

C -3.18429500 -3.35648000 -0.95730500

H -4.20604600 -3.72038500 -0.96595600

C -2.90016200 -2.09933400 -0.42608000

H -3.67168300 -1.43705600 -0.04538400

C -0.84365700 -3.59149200 -1.47268600

H -0.00124500 -4.14173500 -1.87970600

C -2.13901400 -4.11398900 -1.48652500

H -2.32967800 -5.09230700 -1.91746800

H -0.58457000 -0.69849600 -2.86556000

C 0.73082400 -1.68576700 -0.81301400

H 1.40590700 -2.15693800 -1.53684600

O 2.49284100 -3.84553100 -0.27714100

H 2.70457300 -4.78338700 -0.15443400

C 1.34978000 -1.92662800 0.59270500

C 0.43676500 -1.48486600 1.74700600

H 0.95584700 -1.70594600 2.68933200

H -0.50490300 -2.03552400 1.74163500

C 1.62930700 -3.42862700 0.77535400

H 0.68466300 -3.99091200 0.76534100

H 2.09622700 -3.57321800 1.75865100

H 4.02814300 -2.06168000 1.73476400

H -1.58922900 -0.26073700 2.94494100

H -0.06219300 0.02173000 3.83987000

O -2.03017400 0.91184800 -1.51433100

O -2.40654200 1.34349800 1.11656500

C -3.71041800 1.36557800 0.94466700

O -4.30465900 0.73393400 0.06728000

C -4.43859400 2.23917000 1.94576400

H -4.20341400 1.91449500 2.96497900

H -4.09352600 3.27444300 1.84827300

H -5.51507500 2.19141900 1.77727900

H -2.98076100 0.79151900 -1.29295300

C -4.23216388 2.01011612 -4.48469117

H -3.80135477 1.23388887 -5.10210286

H -3.66354861 2.90726960 -4.26630447

C -5.63808105 1.90882644 -4.08379345

O -6.18943443 0.82987934 -3.84832038

O -6.23553594 3.08996097 -3.97109267

H -7.17595084 2.95986471 -3.73406188

[(L^1^)Fe^IV^=O]^1+^ (^5^INT_HS_)

Fe -1.26101200 0.52581600 -0.19638400

N -0.06372600 2.30348700 -0.44308900

C 1.19175500 2.13875100 -0.89867700

C 0.07450000 4.65302600 -0.84554500

H -0.40051600 5.62742300 -0.81380300

C -0.61866000 3.52467900 -0.41891300

H -1.63902800 3.56769300 -0.05295900

C 1.94679500 3.21916000 -1.35057700

H 2.95978600 3.04947300 -1.70037000

C 1.37789500 4.49360300 -1.32153000

H 1.94336600 5.35182800 -1.67190300

N 0.21802400 -0.02511300 1.74979600

N 0.62397300 -0.21269400 -1.17214100

C 0.36602700 -0.12282400 -2.63673100

H 0.12751400 0.90692600 -2.90064600

H 1.24448800 -0.46363100 -3.20147700

C 1.73439200 0.72392400 -0.82383700

H 2.53627400 0.61265600 -1.56221800

O 4.44509900 1.17528000 -0.45749600

H 5.32737600 1.53496600 -0.28159700

C 2.38560100 0.46471500 0.56875000

C 1.45563900 0.78735900 1.75117600

H 2.01571900 0.60946900 2.68251200

H 1.16352000 1.83994700 1.73848400

C 3.64273100 1.34518700 0.70823300

H 3.34802700 2.39794200 0.83121200

H 4.17566000 1.02869000 1.61115400

C 2.77637200 -1.02406900 0.62017600

H 3.39318300 -1.26354400 -0.25248800

O 3.50425900 -1.26609700 1.82052700

C -0.55249000 0.28303200 2.97314400

H -0.84045300 1.33433700 2.96319900

N -1.57971000 -1.61948500 -0.43228600

C -0.53021400 -2.33540500 -0.87895100

C -3.04075100 -3.45734900 -0.87715100

H -4.04801000 -3.85937100 -0.87171500

C -2.81267200 -2.15387600 -0.44372500

H -3.61558600 -1.49465000 -0.12791700

C -0.68475000 -3.64037100 -1.34210200

H 0.18428300 -4.18619100 -1.69527400

C -1.95864000 -4.21136100 -1.33340800

H -2.10555800 -5.22582900 -1.69176200

H -0.50320700 -0.72799500 -2.88966600

C 0.82006400 -1.64779200 -0.80545900

H 1.49157200 -2.11230800 -1.53766100

O 2.64805900 -3.75514600 -0.32720200

H 2.91704600 -4.67666600 -0.19311300

C 1.48027900 -1.86520700 0.58735400

C 0.58178000 -1.45893500 1.76724400

H 1.12187700 -1.69078400 2.69816900

H -0.34430800 -2.03893900 1.76644500

C 1.82246000 -3.35516500 0.76280700

H 0.89952600 -3.95085400 0.80494800

H 2.34082800 -3.47419600 1.72301300

H 4.19570400 -1.91605300 1.62896200

H -1.46816000 -0.30956100 2.97957700

H 0.03837800 0.06390100 3.87563100

O -2.24470000 0.86286800 -1.69266800

O -2.53194600 1.09906800 1.13643700

C -3.84293300 1.13114600 0.99601200

O -4.44284600 0.64784500 0.03283700

C -4.56556200 1.80886500 2.14103100

H -4.35178500 1.27813200 3.07536900

H -4.19150900 2.83158100 2.25938700

H -5.64083800 1.82347200 1.95939900

H -3.19991000 0.82044300 -1.45827400

C -4.52685581 2.58011170 -4.21375727

H -4.52237872 1.83442159 -4.99685920

H -3.70609415 3.28391227 -4.13059779

C -5.71881760 2.73041479 -3.37458043

O -6.43248940 1.77671133 -3.05102637

O -5.92476927 3.98175141 -2.97901355

H -6.74777502 4.02715049 -2.45167185

[(L^2^)Fe^IV^=O]^1+^ (^1^INT_LS_)

Fe 1.47438400 -0.31525200 -0.18824900

N 0.90926800 -2.20612000 -0.35753300

C -0.31079700 -2.37238800 -0.90703300

C 1.31905300 -4.54680700 -0.52499300

H 1.99069400 -5.38191800 -0.35952200

C 1.71627700 -3.25830600 -0.17512800

H 2.68204000 -3.02951600 0.25741400

C -0.77041900 -3.62525000 -1.29330600

H -1.75586900 -3.72919300 -1.73514900

C 0.06010900 -4.73172700 -1.09582600

H -0.27208200 -5.72272800 -1.38888800

N 0.11238500 -0.03108900 1.59098400

N -0.20974100 0.01760700 -1.30228100

C 0.07703700 -0.04276400 -2.76648700

H 0.50207200 -1.01439000 -3.00844300

H -0.85110600 0.12665600 -3.32760400

C -1.14385000 -1.10888000 -0.96148800

H -1.90717900 -1.19022500 -1.74272800

C -1.89036600 -0.91659500 0.39158800

C -0.97427500 -1.04394600 1.61800600

H -1.58786800 -0.91487100 2.51760400

H -0.53137100 -2.03674100 1.66936400

C -2.55116100 0.46970000 0.36300600

H -3.13692100 0.56788300 -0.55804900

O -3.38370300 0.57828700 1.50160700

C 0.91011400 -0.17299500 2.83520100

H 1.37098500 -1.15874200 2.85568400

N 1.69961900 1.68217800 -0.35568400

C 0.61709700 2.27124400 -0.91931200

C 2.91675400 3.72826500 -0.59153200

H 3.84765900 4.26539500 -0.44720100

C 2.82882200 2.39325100 -0.20098100

H 3.67862400 1.85245800 0.19273700

C 0.64302400 3.59073500 -1.35146300

H -0.23876200 4.02931000 -1.80601800

C 1.81126300 4.33600900 -1.18051400

H 1.85431200 5.36991700 -1.50821400

H 0.82185700 0.70910700 -3.01686600

C -0.63710800 1.41836100 -0.96670700

H -1.30636800 1.78263100 -1.75706700

C -1.42077400 1.52124300 0.37989000

C -0.52229900 1.30982000 1.60724300

H -1.14459600 1.41320800 2.50579100

H 0.25252300 2.07242100 1.65274500

H -4.02398400 1.28780800 1.33321500

H 1.70614000 0.57104000 2.83385800

H 0.26601300 -0.03520900 3.71417700

O 2.47337600 -0.56887700 -1.63442000

O 2.88835700 -0.73752000 1.02974600

C -2.97204400 -2.00360200 0.50175200

O -2.98880700 -2.86062500 1.35289000

O -3.87623000 -1.89152600 -0.48753100

C -1.99159500 2.94360800 0.47037800

O -1.42096600 3.87192900 0.99390300

O -3.19313800 3.03849900 -0.13364200

C -3.78872300 4.36119400 -0.13603600

H -4.73931300 4.24881000 -0.65561300

H -3.93806900 4.70663200 0.88933500

H -3.13700500 5.06332500 -0.66098700

C -4.96480500 -2.84478200 -0.44466100

H -5.52465700 -2.72790600 0.48593700

H -5.58801100 -2.61238200 -1.30733900

H -4.57616600 -3.86433600 -0.50491900

C 4.17241400 -0.56702900 0.86514600

O 4.69517600 -0.02269800 -0.11616300

C 5.00728900 -1.10535200 2.01377300

H 4.67485700 -0.66446300 2.95958700

H 4.86802100 -2.18919000 2.09633800

H 6.06278400 -0.88510200 1.84891500

H 3.36732500 -0.22895800 -1.39623900

C 4.71348404 -0.29783990 -4.49141549

H 4.16972039 0.08336263 -5.34483693

H 4.55160035 -1.31634948 -4.15625531

C 5.77800053 0.51625674 -3.89842900

O 5.72723061 1.74852203 -3.84833273

O 6.77286131 -0.20839810 -3.39856903

H 7.46625203 0.38585389 -3.04705417

[(L^2^)Fe^IV^=O]^1+^ (^3^INT_IS_)

Fe 1.56183400 -0.41578400 -0.04604700

N 0.67372900 -2.39425300 -0.43580600

C -0.53640800 -2.36158400 -1.01533700

C 0.84037300 -4.73101900 -0.88858600

H 1.41551100 -5.64816200 -0.82265300

C 1.35868000 -3.54177200 -0.37889600

H 2.33651500 -3.47712200 0.08670300

C -1.12238500 -3.50135900 -1.55689200

H -2.10212400 -3.44336800 -2.02018600

C -0.42006700 -4.70736000 -1.48663700

H -0.84975000 -5.61425100 -1.90087400

N 0.17667200 -0.03424600 1.62651100

N -0.21247400 0.04367500 -1.28100000

C 0.14618700 -0.05357400 -2.72110000

H 0.53317600 -1.04936100 -2.92828400

H -0.73394800 0.15340700 -3.34538400

C -1.22361800 -1.00875000 -0.96299900

H -2.02347200 -0.97748700 -1.71307800

C -1.89875400 -0.80912100 0.42797100

C -0.95536200 -1.00193100 1.62797700

H -1.54834800 -0.86948400 2.54019300

H -0.54848100 -2.01052100 1.64583200

C -2.49301000 0.60702400 0.44812000

H -3.11994900 0.74360700 -0.43984200

O -3.26067000 0.74695200 1.62871500

C 0.92674200 -0.20023800 2.90022100

H 1.32969700 -1.20996700 2.95029300

N 1.73375600 1.75832400 -0.43633300

C 0.65569300 2.30502200 -1.02587100

C 3.00479400 3.71789600 -0.93631700

H 3.95365700 4.24086600 -0.88701000

C 2.89137300 2.43543600 -0.40210700

H 3.73366200 1.90654300 0.03297300

C 0.69553800 3.57127600 -1.59844200

H -0.18861000 3.98346900 -2.07414000

C 1.88967000 4.29320600 -1.54430400

H 1.94866700 5.28541100 -1.98089400

H 0.94424300 0.65221600 -2.94126000

C -0.60116800 1.45293000 -0.97390500

H -1.31231000 1.80437100 -1.73568400

C -1.30954600 1.59668900 0.41049200

C -0.38232500 1.34433400 1.61128800

H -0.97549300 1.49741100 2.52170800

H 0.43852500 2.05751100 1.62355400

H -3.86910800 1.49157400 1.49786100

H 1.76151200 0.50093500 2.91420800

H 0.26410600 -0.01219700 3.75473600

O 2.57382900 -0.67508300 -1.48575800

O 2.89931000 -1.02224700 1.16512800

C -3.02534500 -1.84664500 0.56496900

O -3.05318100 -2.71298600 1.40650400

O -3.95816000 -1.67998600 -0.38962000

C -1.79507600 3.04912900 0.52299200

O -1.14273700 3.94652800 1.00335400

O -3.02404000 3.20834000 -0.00769800

C -3.54110100 4.56322600 0.01156300

H -4.52490500 4.50350700 -0.45194900

H -3.61128200 4.92293700 1.04052500

H -2.88066200 5.22289300 -0.55581700

C -5.08100600 -2.59072400 -0.32429800

H -5.60146200 -2.47269400 0.62874800

H -5.72611400 -2.31610300 -1.15806600

H -4.73476100 -3.62284600 -0.41952700

C 4.19653600 -0.83464400 1.04812100

O 4.71780500 -0.12874000 0.18094300

C 5.01064400 -1.55932500 2.09987600

H 4.69250800 -1.24325500 3.09914800

H 4.82827100 -2.63716800 2.02846300

H 6.07298600 -1.35345900 1.96407300

H 3.48467800 -0.40426300 -1.23069700

C 5.57953752 -0.55502786 -3.62651433

H 4.86168849 -0.80413477 -4.39590431

H 6.07243473 -1.34379771 -3.06902451

C 5.99363917 0.84057276 -3.45799014

O 5.22843321 1.79130925 -3.64271951

O 7.25425010 0.96679634 -3.05841501

H 7.48395414 1.91581454 -2.99446720

[(L^2^)Fe^IV^=O]^1+^ (^5^INT_HS_)

Fe 1.68487900 -0.47046600 -0.19923400

N 0.69439400 -2.36988600 -0.44458800

C -0.54702300 -2.36109500 -0.96225600

C 0.81185400 -4.73907700 -0.71491100

H 1.37829400 -5.65679600 -0.60156300

C 1.36717700 -3.52362900 -0.32557500

H 2.36574300 -3.44130200 0.09024500

C -1.16444400 -3.53386500 -1.38694300

H -2.16537800 -3.49845200 -1.80427100

C -0.47380200 -4.74060400 -1.25809700

H -0.93327200 -5.66945900 -1.58159000

N 0.08925000 -0.05238500 1.65478700

N -0.24280800 0.04067800 -1.28670400

C 0.08622800 -0.04707200 -2.73657300

H 0.44827000 -1.04902300 -2.96383300

H -0.79893900 0.18316900 -3.34584800

C -1.24578700 -1.01302100 -0.95620200

H -2.03272400 -1.01188100 -1.72103900

C -1.95628700 -0.79971800 0.41737700

C -1.04636200 -1.00138200 1.64083000

H -1.66452400 -0.87207200 2.53901700

H -0.65507800 -2.01773600 1.66458600

C -2.53040300 0.62446600 0.41773300

H -3.12740800 0.76994400 -0.48980500

O -3.33390500 0.77108200 1.57290700

C 0.84797200 -0.24812900 2.91067200

H 1.25178700 -1.26010300 2.93284600

N 1.76349500 1.68891900 -0.46033000

C 0.67273700 2.28050100 -0.98248500

C 3.04226100 3.66720200 -0.85953600

H 3.99722800 4.17766700 -0.80024400

C 2.92962900 2.35368600 -0.41134200

H 3.77940500 1.79163700 -0.03666100

C 0.71621200 3.58204700 -1.47143500

H -0.17431900 4.03376000 -1.89565600

C 1.91724400 4.28938100 -1.39996000

H 1.97414500 5.30776800 -1.77160100

H 0.89508800 0.64459200 -2.96612700

C -0.60065300 1.45175800 -0.95499500

H -1.29191400 1.83352200 -1.72134200

C -1.33360500 1.59870600 0.41843100

C -0.43601500 1.32892800 1.63857700

H -1.03985700 1.51202400 2.53921500

H 0.39995500 2.02677300 1.65615500

H -3.92093800 1.53001700 1.42749900

H 1.68813800 0.44702000 2.93355200

H 0.20356800 -0.08198100 3.78649000

O 2.72980800 -0.70674000 -1.67384800

O 2.97228800 -0.90357600 1.16310400

C -3.10481700 -1.81716500 0.52244300

O -3.16496600 -2.69339500 1.35198900

O -4.01764800 -1.62484000 -0.44801400

C -1.80771700 3.05553500 0.52411600

O -1.15611800 3.94780100 1.01508500

O -3.02723500 3.22874700 -0.02650700

C -3.53293100 4.58743300 -0.00769400

H -4.51090700 4.53846300 -0.48470600

H -3.61395500 4.94321600 1.02185700

H -2.85993700 5.24481200 -0.56298600

C -5.16356800 -2.50697000 -0.40452900

H -5.69680700 -2.37841400 0.54007800

H -5.78761700 -2.21414600 -1.24809600

H -4.84313200 -3.54778700 -0.49682200

C 4.28429800 -0.79708700 1.05685700

O 4.85253000 -0.26951100 0.09843100

C 5.04249200 -1.37116900 2.23408200

H 4.74907400 -0.84659100 3.15008600

H 4.77453200 -2.42483300 2.36751000

H 6.11776000 -1.27581700 2.07903400

H 3.67038600 -0.57165600 -1.41473800

C 5.57953752 -0.55502786 -3.62651433

H 4.86168849 -0.80413477 -4.39590431

H 6.07243473 -1.34379771 -3.06902451

C 5.99363917 0.84057276 -3.45799014

O 5.22843321 1.79130925 -3.64271951

O 7.25425010 0.96679634 -3.05841501

H 7.48395414 1.91581454 -2.99446720

[(L^1^)Fe^IV^=O]^1+^ (^3^TS2_IS_)

Fe 0.64945000 -0.17359300 0.09174900

N 0.23651800 -2.11286700 -0.21407700

C -0.92806500 -2.32428500 -0.86235200

C 0.83647100 -4.40003700 -0.51947800

H 1.55846700 -5.19502600 -0.36724200

C 1.10625300 -3.11777000 -0.04579400

H 2.01388300 -2.84809700 0.48047600

C -1.26244700 -3.57595800 -1.36962400

H -2.20796400 -3.70935600 -1.88525300

C -0.36372400 -4.63124300 -1.19289400

H -0.59637400 -5.61800300 -1.58163900

N -0.93654300 0.03336300 1.75425400

N -0.94427100 0.07797800 -1.16614300

C -0.51810600 0.05733800 -2.59714200

H 0.02592000 -0.86180000 -2.80222600

H -1.40192500 0.13728400 -3.24259600

C -1.83501400 -1.11438000 -0.93431400

H -2.51812400 -1.21347700 -1.78419000

O -4.47546400 -2.11373400 -0.87084800

H -5.15678100 -2.80280800 -0.84866400

C -2.72610100 -0.99846200 0.33478200

C -1.92743300 -1.06247500 1.64729400

H -2.63763500 -0.99929800 2.48475900

H -1.38980500 -2.00922600 1.73319000

C -3.73817900 -2.15637100 0.34709900

H -3.21065200 -3.11412600 0.46259800

H -4.39271600 -2.02673300 1.21858200

C -3.47158800 0.35478000 0.25591400

H -3.98253900 0.43812300 -0.70913700

O -4.40633000 0.47115300 1.32359800

C -0.25747100 -0.05670700 3.06797300

H 0.24925300 -1.01675900 3.14542900

N 0.75394800 1.84046300 -0.10781400

C -0.33590600 2.37053700 -0.72029600

C 1.85751200 3.95755300 -0.28456900

H 2.75147200 4.54322400 -0.10155100

C 1.83378000 2.61721600 0.10341100

H 2.66866000 2.12155900 0.58911700

C -0.37479600 3.69530100 -1.13503500

H -1.27059700 4.07223500 -1.61801800

C 0.74329400 4.50556600 -0.91225000

H 0.74033100 5.54261800 -1.23419200

H 0.14986200 0.89560100 -2.78773000

C -1.51588800 1.43511500 -0.87162900

H -2.12925100 1.74878400 -1.72177800

O -3.75015600 3.07913200 -0.85793200

H -4.35656100 3.83155400 -0.78762400

C -2.43734100 1.49056500 0.37982900

C -1.65630700 1.32793100 1.69070400

H -2.36296300 1.39813900 2.53068400

H -0.92085700 2.12644000 1.80771400

C -3.14996500 2.85559300 0.41488200

H -2.42348700 3.64637800 0.65216200

H -3.89971200 2.82306500 1.21285900

H -5.28316700 0.23655800 0.98685100

H 0.49522300 0.73037700 3.13491300

H -0.98663300 0.05513800 3.88345700

O 1.94477400 -0.33720200 -1.26016400

O 1.88453700 -0.76106500 1.44433900

C 3.00160300 -0.30960100 1.93919900

O 3.47680900 0.81743500 1.76607800

C 3.75796200 -1.34174500 2.76196200

H 3.08319800 -2.06572500 3.22600500

H 4.43101400 -1.87874200 2.08217000

H 4.36676700 -0.84609300 3.52137500

H 2.20800500 0.56949800 -1.50064600

C 3.85696900 -0.91718000 -2.02281600

H 3.61816500 -0.67443500 -3.04937300

H 3.88649700 -1.95447100 -1.71800300

C 4.47709700 0.11006700 -1.19470400

O 4.31906900 1.31127600 -1.38675800

O 5.15048100 -0.39265500 -0.13974000

H 5.24230400 0.33564500 0.50899800

[(L^1^)Fe^IV^=O]^1+^ (^5^TS2_HS_)

Fe -0.74235600 0.22949100 0.23943400

N -0.04508200 2.23640500 -0.31331900

C 1.13607100 2.30969700 -0.94668200

C -0.44823200 4.54325800 -0.77223700

H -1.10266400 5.40411600 -0.68611600

C -0.82897400 3.31853100 -0.22662000

H -1.76521400 3.16596300 0.29903000

C 1.59130400 3.49585600 -1.51767000

H 2.56072600 3.51793300 -2.00479100

C 0.78224500 4.63075600 -1.42627400

H 1.10706300 5.56984400 -1.86431600

N 0.87127700 -0.04681700 1.76603200

N 0.98645200 -0.10320200 -1.16709100

C 0.49825000 -0.04354500 -2.56852500

H -0.00990100 0.90385600 -2.73754200

H 1.33713600 -0.15718600 -3.26934500

C 1.93912000 1.02174700 -0.94209100

H 2.66495200 1.04916000 -1.76277900

O 4.53806800 1.98826900 -0.81950200

H 5.32704300 2.54304000 -0.72739000

C 2.76323000 0.88830500 0.37193200

C 1.91377900 1.00867100 1.65160400

H 2.59009700 0.93604200 2.51529600

H 1.40806700 1.97437700 1.69858700

C 3.82452700 2.00437400 0.41368200

H 3.33391800 2.97639400 0.57114600

H 4.48827400 1.80893800 1.26312800

C 3.44585000 -0.49161100 0.33819200

H 4.00591000 -0.59092100 -0.59698400

O 4.32425100 -0.59908300 1.45481300

C 0.22668200 0.06187100 3.10073700

H -0.21635300 1.04996400 3.20569500

N -0.78624900 -1.90664900 -0.16711900

C 0.29150300 -2.40130800 -0.81161300

C -1.92146400 -3.99132600 -0.45498600

H -2.81906300 -4.57982100 -0.30121800

C -1.87274300 -2.67863900 0.01318100

H -2.69948800 -2.20219600 0.52863600

C 0.30935400 -3.69946200 -1.31071500

H 1.19483900 -4.06097600 -1.82403700

C -0.81738100 -4.50732900 -1.12979600

H -0.83050800 -5.52140500 -1.51806000

H -0.22608500 -0.83978400 -2.73498000

C 1.49310000 -1.47605500 -0.89263300

H 2.13923000 -1.79839900 -1.71825400

O 3.77127400 -3.16398500 -0.69534300

H 4.21818000 -4.02210600 -0.63731900

C 2.34726500 -1.57754900 0.40405200

C 1.52900500 -1.37857200 1.68853000

H 2.21346200 -1.48208900 2.54176200

H 0.75203700 -2.13870200 1.78631600

C 2.99625000 -2.97020100 0.48351700

H 2.21995000 -3.74186400 0.58425700

H 3.62192200 -3.00462200 1.38512300

H 5.11351000 -1.08412000 1.17370700

H -0.57165300 -0.67952500 3.17072400

H 0.96657500 -0.11106100 3.89422400

O -1.99643900 0.42591500 -1.10229500

O -1.88885300 1.05489000 1.57827600

C -3.02830900 0.55166300 1.96320300

O -3.38270300 -0.61477600 1.74541900

C -3.93907700 1.52443300 2.68726900

H -4.57645500 0.99009900 3.39546700

H -3.36962600 2.30436400 3.19797100

H -4.58437300 1.99733200 1.93715100

H -2.34971500 -0.46353900 -1.28920500

C -4.14991400 0.91631800 -2.06680000

H -3.80066600 0.62448000 -3.04777600

H -4.25853000 1.96332800 -1.81684000

C -4.59517000 -0.11972500 -1.14733700

O -4.31167100 -1.30670800 -1.29028000

O -5.26239100 0.36071600 -0.08249200

H -5.21604000 -0.32870200 0.61363800

[(L^2^)Fe^IV^=O]^1+^ (^3^TS2_IS_)

Fe 1.03895400 -0.18959400 0.07025800

N 0.64734500 -2.13700900 -0.20754700

C -0.51242500 -2.37456200 -0.85421000

C 1.23534800 -4.43936400 -0.40064500

H 1.95046400 -5.23051400 -0.20314100

C 1.51030400 -3.13717400 0.01264800

H 2.41443800 -2.84908000 0.53480200

C -0.85250400 -3.64561700 -1.30232100

H -1.79343500 -3.80574300 -1.81832700

C 0.03794200 -4.69677300 -1.06789800

H -0.20106400 -5.70039500 -1.40567500

N -0.55530800 0.00464000 1.71483100

N -0.55622200 0.03121900 -1.21007500

C -0.11823100 -0.00492700 -2.63743100

H 0.43694100 -0.92147700 -2.82238100

H -0.99729400 0.05557700 -3.29217900

C -1.42773000 -1.17207000 -0.96584500

H -2.10402600 -1.29406800 -1.81888700

O -4.10809900 -2.19095600 -0.78060000

C -2.32062800 -1.05056700 0.30466900

C -1.53371900 -1.10392800 1.62375900

H -2.25251600 -1.04941300 2.45084300

H -1.00182500 -2.04969700 1.71702400

C -3.09097800 0.27496800 0.19738600

H -3.58473700 0.32329900 -0.78010800

O -4.04394100 0.30812600 1.24185300

C 0.11910800 -0.06263700 3.03397100

H 0.63524200 -1.01636700 3.12375200

N 1.11668800 1.82217500 -0.14614800

C 0.03391500 2.33728500 -0.78351500

C 2.20371600 3.94777100 -0.32126800

H 3.08730300 4.54422900 -0.12368300

C 2.18451500 2.61065300 0.07847500

H 3.01662000 2.12567400 0.58061300

C -0.00459900 3.65387400 -1.21730800

H -0.88423200 4.03100200 -1.72780600

C 1.10004000 4.47778500 -0.97983400

H 1.09229900 5.51123700 -1.31207800

H 0.54420400 0.83625100 -2.83305000

C -1.13822300 1.38789900 -0.93445000

H -1.75507300 1.68708800 -1.79255900

O -3.90408500 2.77468200 -0.37756100

C -2.06283800 1.42063000 0.32241700

C -1.28628600 1.28912000 1.64004400

H -2.00667400 1.35790000 2.46656500

H -0.57827200 2.10901500 1.74949400

H -4.71451800 0.96976100 1.00848300

H 0.86034400 0.73534900 3.09642900

H -0.61684900 0.04787600 3.84271900

O 2.33353200 -0.34949500 -1.28243800

O 2.27854600 -0.74198000 1.42684800

C 3.40119100 -0.28555500 1.90687300

O 3.87915100 0.83603000 1.71060800

C 4.15831700 -1.30648700 2.74200000

H 3.48417500 -2.02258600 3.21875300

H 4.82934400 -1.85381600 2.06831800

H 4.76972100 -0.80014400 3.49215300

H 2.60236600 0.55805800 -1.51437300

C 4.24735600 -0.92174500 -2.05440900

H 3.99910700 -0.68838100 -3.08090700

H 4.28931600 -1.95649700 -1.74236400

C 4.86118100 0.11841300 -1.23680100

O 4.68133200 1.31598800 -1.43354700

O 5.55182600 -0.36893700 -0.18704700

H 5.63848000 0.36317700 0.45833400

C -3.31205200 -2.22614300 0.30396200

O -3.35117200 -3.08250100 1.15460800

C -2.75976800 2.78896800 0.33662100

O -2.32295700 3.76743900 0.89551300

C -5.11175400 -3.23184200 -0.84695500

H -5.65563400 -3.05190900 -1.77350800

H -4.63548400 -4.21538700 -0.85634900

H -5.77649100 -3.16290700 0.01700600

C -4.60776300 4.04078900 -0.45377300

H -5.49376100 3.84299400 -1.05557900

H -4.87930300 4.37939600 0.54854100

H -3.97317200 4.79301300 -0.92787100

[(L^2^)Fe^IV^=O]^1+^ (^5^TS2_HS_)

Fe 1.13198200 -0.24981100 0.21740800

N 0.47516700 -2.27326900 -0.31839200

C -0.69402900 -2.38796200 -0.96703400

C 0.91212600 -4.59150100 -0.68056100

H 1.57361300 -5.44063100 -0.54655400

C 1.27002900 -3.34119800 -0.17795000

H 2.19475000 -3.15753600 0.35764600

C -1.12253500 -3.59816000 -1.50371700

H -2.07402200 -3.65731800 -2.02248000

C -0.30320500 -4.71974800 -1.35364500

H -0.60949700 -5.67848200 -1.76082800

N -0.50396200 0.00730100 1.71340400

N -0.60788300 0.02880000 -1.23272000

C -0.09551000 -0.03809900 -2.62472100

H 0.44121800 -0.97374200 -2.76851000

H -0.92466000 0.04185700 -3.34229900

C -1.52674400 -1.11860500 -0.99955900

H -2.25082600 -1.18047400 -1.82222100

O -4.22592400 -2.05621300 -0.68506200

C -2.35074700 -0.98286700 0.31730200

C -1.51692100 -1.07560800 1.60875700

H -2.21091700 -1.01509400 2.45549700

H -1.00439900 -2.03357300 1.67210500

C -3.08044900 0.36718100 0.25217900

H -3.62086400 0.43469500 -0.69848100

O -3.97858000 0.43683100 1.34367700

C 0.12915400 -0.06753200 3.05761600

H 0.59433900 -1.04321100 3.17969800

N 1.11961800 1.86874100 -0.21486400

C 0.04719500 2.34286900 -0.88288300

C 2.21853900 3.97515400 -0.47846400

H 3.09876600 4.58285100 -0.30130500

C 2.18609100 2.66185900 -0.01070500

H 3.01099300 2.20165400 0.52279500

C 0.01805100 3.63567500 -1.38890300

H -0.85478200 3.98789300 -1.92898600

C 1.12278700 4.46677200 -1.18246500

H 1.12297900 5.48052300 -1.57096100

H 0.61068900 0.77426900 -2.78990900

C -1.13747900 1.39403500 -0.97543000

H -1.78438300 1.70056000 -1.81131000

O -3.81956600 2.89614300 -0.30309400

C -2.00450600 1.47213000 0.32264600

C -1.19138700 1.32025400 1.61826500

H -1.89105500 1.42083900 2.45809200

H -0.45104500 2.11260500 1.70633000

H -4.63986200 1.11658900 1.13781600

H 0.90600400 0.69630900 3.12650900

H -0.62471800 0.09560000 3.83919300

O 2.39348500 -0.43298100 -1.12119200

O 2.28515800 -1.03519900 1.56911800

C 3.41860200 -0.51526900 1.95264700

O 3.76184500 0.65107900 1.71980800

C 4.33540800 -1.46906200 2.69323900

H 4.96002500 -0.91932500 3.40096200

H 3.77198900 -2.25103300 3.20733800

H 4.99318800 -1.94062900 1.95304400

H 2.74152100 0.45960100 -1.30412600

C 4.55949600 -0.89144800 -2.07102700

H 4.20947800 -0.61286400 -3.05559200

H 4.68912700 -1.93449500 -1.81436000

C 4.98139900 0.15965700 -1.15671700

O 4.66730800 1.33822800 -1.30592700

O 5.66037600 -0.29911200 -0.09076800

H 5.59808100 0.39227600 0.60246400

C -3.37846200 -2.12585600 0.35792000

O -3.40470400 -2.98803700 1.20369000

C -2.64621600 2.86645500 0.36119900

O -2.14859000 3.82850700 0.89820700

C -5.25723400 -3.07063700 -0.71320000

H -5.84293900 -2.86429500 -1.60830000

H -4.80673200 -4.06522900 -0.75998600

H -5.87557900 -2.99849800 0.18435400

C -4.47342500 4.18927000 -0.35585300

H -5.39032900 4.02658300 -0.92080000

H -4.69054900 4.54124200 0.65514200

H -3.82834800 4.91402000 -0.85782500

[(L^1^)Fe^IV^=O]^1+^ (^1^P_LS_)

Fe 0.10811700 -1.13938800 0.21882800

N -1.86079300 -1.23570600 -0.02223100

C -2.34512500 -0.19964800 -0.74694300

C -4.00171500 -2.30064500 -0.14295700

H -4.62556000 -3.14781300 0.12104800

C -2.67320300 -2.26231200 0.27479900

H -2.22640100 -3.05254400 0.86718600

C -3.65668200 -0.17004400 -1.20690100

H -4.00052200 0.68656400 -1.77753100

C -4.50050900 -1.24115900 -0.90082600

H -5.52902800 -1.24608300 -1.24831800

N -0.02969700 0.34205600 1.71140300

N -0.01974300 0.31344900 -1.14514800

C 0.02935300 -0.27392800 -2.51085700

H -0.79139900 -0.98111200 -2.62671100

H -0.04156200 0.51387000 -3.27243200

C -1.36369600 0.94023100 -0.92775600

H -1.63721100 1.54126000 -1.80072200

O -2.95589500 3.21401800 -0.97123700

H -3.67577200 3.85998200 -0.91610400

C -1.42052500 1.88341400 0.31277900

C -1.29768900 1.11625500 1.64246700

H -1.33288200 1.83760400 2.47101300

H -2.12715500 0.42001600 1.77183200

C -2.76560900 2.63304500 0.31539000

H -3.57942700 1.93202000 0.55365900

H -2.72542400 3.39674100 1.09963000

C -0.25399300 2.88020900 0.18622200

H -0.30482700 3.36450000 -0.79453300

O -0.36811300 3.85053900 1.22155900

C 0.03490400 -0.39678000 2.99335500

H -0.78776400 -1.11254500 3.02985600

N 2.06412000 -0.88555200 -0.01807400

C 2.35727100 0.21100100 -0.75704500

C 4.35172100 -1.57867400 -0.16452400

H 5.11204600 -2.30694100 0.09631000

C 3.04139100 -1.75802600 0.27400300

H 2.74085900 -2.60782900 0.87626000

C 3.63754800 0.44911400 -1.24389500

H 3.82638800 1.33483500 -1.84216100

C 4.65403300 -0.46213800 -0.94339100

H 5.66204400 -0.30186500 -1.31325700

H 0.95977900 -0.82826200 -2.63043600

C 1.19380200 1.16625100 -0.93083700

H 1.35751200 1.81147600 -1.80170800

O 2.20359600 3.85851400 -0.85801800

H 2.93926500 4.48955300 -0.85282500

C 1.07503500 2.09855200 0.31177300

C 1.08289800 1.32417500 1.64246500

H 0.98274200 2.04588100 2.46496800

H 2.02207000 0.78655800 1.78088600

C 2.25680900 3.08046400 0.33344200

H 3.20371000 2.52760200 0.41186400

H 2.16371700 3.71057700 1.22824600

H -0.00491400 4.68441300 0.88801400

H 0.96936800 -0.95889000 3.03016500

H -0.02534200 0.28909300 3.85015200

O 0.25983300 -2.88146900 1.27542000

C 0.31532800 -3.51472300 0.16618700

O 0.26301100 -2.82910200 -0.90531000

C 0.42264000 -5.01492600 0.12106500

H 1.23866800 -5.30843400 -0.54662100

H 0.58577000 -5.42172500 1.12063200

H -0.50038800 -5.43239500 -0.29696000

[(L^1^)Fe^IV^=O]^1+^ (^3^P_IS_)

Fe 1.19899600 -0.09695200 0.22865100

N 0.92319400 -2.04582700 0.00508900

C -0.20217600 -2.34972900 -0.68776200

C 1.58121800 -4.34522900 -0.06425100

H 2.30770100 -5.10489500 0.20308500

C 1.79427100 -3.02168600 0.31129300

H 2.66703000 -2.70048400 0.86789700

C -0.47352700 -3.64785300 -1.10724700

H -1.38585800 -3.84588200 -1.66055200

C 0.43309500 -4.66251500 -0.79065500

H 0.24558200 -5.68336900 -1.10905600

N -0.51948900 0.03239000 1.79297400

N -0.32280200 0.03328000 -1.12368800

C 0.27953200 -0.01853600 -2.48540900

H 0.83656300 -0.94841100 -2.59947400

H -0.50424200 0.04827000 -3.25121100

C -1.16088800 -1.19215700 -0.90506700

H -1.76376900 -1.37808900 -1.79970600

O -3.66420400 -2.37068800 -1.08182300

H -4.43204700 -2.96123800 -1.06535700

C -2.16376000 -1.07824400 0.28680400

C -1.46897700 -1.08952300 1.66218900

H -2.23794100 -1.03334600 2.44852000

H -0.91574000 -2.02053300 1.80468100

C -3.13623600 -2.27159300 0.23784300

H -2.60410900 -3.19294100 0.51774000

H -3.92656100 -2.09191200 0.97443100

C -2.93382500 0.24499100 0.11205400

H -3.36418200 0.27749600 -0.89468200

O -3.96535100 0.30351800 1.09182000

C 0.20580100 -0.03377700 3.07372800

H 0.76661900 -0.97092600 3.11670000

N 1.25155900 1.86803800 0.00546400

C 0.19808200 2.35805000 -0.69414400

C 2.30356100 4.01594400 -0.07974300

H 3.15101400 4.63875200 0.18524000

C 2.27876400 2.67888000 0.30819500

H 3.07974500 2.21454800 0.87185000

C 0.16491800 3.67796700 -1.13109700

H -0.68831900 4.02870000 -1.70283300

C 1.23416400 4.52172900 -0.81865700

H 1.23190100 5.55517600 -1.15118800

H 0.98785100 0.80191600 -2.59968400

C -0.94439400 1.38012000 -0.90446000

H -1.51514900 1.66623700 -1.79535400

O -3.37634100 2.86327400 -0.98538600

H -3.89165700 3.68413000 -0.99628200

C -1.94496200 1.42177400 0.29246100

C -1.25877900 1.30233900 1.66676700

H -2.02962700 1.37149200 2.44979600

H -0.55408200 2.12353300 1.81818300

C -2.71049700 2.75477200 0.26893000

H -2.01365700 3.59013600 0.42778600

H -3.42414700 2.75309600 1.10313300

H -4.72377100 0.76254400 0.70202800

H 0.92289500 0.79006200 3.12032900

H -0.47586200 0.02546400 3.93597000

O 2.98616000 -0.25964100 1.10280800

C 3.70428600 -0.30163700 0.02369700

O 3.15851700 -0.24053700 -1.10221800

C 5.20496700 -0.39114200 0.17757000

H 5.61158200 0.61879500 0.31236700

H 5.47001200 -0.97590300 1.06201300

H 5.65303000 -0.82188900 -0.72004400

[(L^1^)Fe^IV^=O]^1+^ (^5^P_HS_)

Fe 1.21549100 -0.48637000 0.36946000

N 0.17715900 -2.35988000 -0.11104000

C -0.97124900 -2.20131800 -0.79733800

C 0.07549600 -4.72224800 -0.48235700

H 0.52365300 -5.69988700 -0.34134000

C 0.69393100 -3.58824800 0.03868500

H 1.62564000 -3.64016100 0.59340800

C -1.65130800 -3.28420500 -1.35041700

H -2.58138100 -3.11322400 -1.88280000

C -1.11877500 -4.56475900 -1.18758600

H -1.62603500 -5.42626200 -1.61133300

N -0.38694400 0.14827700 1.83039500

N -0.31550800 0.12587600 -1.10113500

C 0.24978900 -0.10370100 -2.45519400

H 0.51134700 -1.15621400 -2.56646400

H -0.46963000 0.18405800 -3.23476900

C -1.48795200 -0.77362700 -0.89025500

H -2.15904600 -0.69764300 -1.75368300

O -4.24027800 -1.15636800 -0.92130200

H -5.13977000 -1.51191800 -0.86498500

C -2.34664800 -0.41130000 0.36327000

C -1.62384500 -0.65626700 1.70285700

H -2.31614700 -0.40512000 2.52073000

H -1.34910900 -1.70801100 1.80656100

C -3.62527700 -1.27074800 0.35914500

H -3.36859700 -2.31782100 0.57888600

H -4.28301500 -0.90141500 1.15348100

C -2.71174600 1.08082300 0.25001900

H -3.18066300 1.26112200 -0.72274900

O -3.61350500 1.41423200 1.30152700

C 0.25900500 -0.11021400 3.13472400

H 0.51867600 -1.16986700 3.20223300

N 1.74986400 1.59402900 -0.10722700

C 0.81516900 2.26138000 -0.81148300

C 3.31260800 3.36471400 -0.49760400

H 4.31089800 3.76417600 -0.35498900

C 2.97067900 2.12738400 0.04350000

H 3.67385300 1.53035700 0.61590900

C 1.07919400 3.50149800 -1.38879000

H 0.29855800 4.00924500 -1.94647700

C 2.34819500 4.06190000 -1.22678300

H 2.58196300 5.02525500 -1.66988300

H 1.16732000 0.47414300 -2.56917600

C -0.54473300 1.58586900 -0.89562500

H -1.08930200 1.99300000 -1.75679700

O -2.41148100 3.72726800 -0.84748400

H -2.66648600 4.66220700 -0.82983200

C -1.41135900 1.90967600 0.36130000

C -0.72265100 1.58404100 1.70153800

H -1.40292500 1.87739100 2.51503600

H 0.20190500 2.15492400 1.81158300

C -1.74814300 3.41061200 0.37270400

H -0.82810700 4.00057300 0.49273900

H -2.39001000 3.61078400 1.24069300

H -4.24657400 2.06260600 0.96059200

H 1.18403800 0.46877600 3.19809100

H -0.39961600 0.15889600 3.97369400

O 2.86687100 -1.10052000 1.35583500

C 3.53936000 -1.38523600 0.28159000

O 3.01905600 -1.20836000 -0.84725600

C 4.93857000 -1.92362900 0.44354600

H 5.54608500 -1.20268700 1.00113900

H 4.91051800 -2.84248100 1.03900900

H 5.39202800 -2.12168200 -0.52863600

[(L^2^)Fe^IV^=O]^1+^ (^1^P_LS_)

Fe 1.50006400 -0.28082500 0.17718200

N 1.04738200 -2.20095800 -0.04024900

C -0.08200500 -2.39130700 -0.76148600

C 1.45384900 -4.55941900 -0.08926800

H 2.08472500 -5.39171500 0.20337700

C 1.79871100 -3.26302600 0.28848800

H 2.68356900 -3.04298200 0.87453900

C -0.48484700 -3.65089900 -1.18546500

H -1.39597900 -3.76148400 -1.76446400

C 0.29788000 -4.75712100 -0.84278200

H 0.00662300 -5.75384500 -1.15875300

N 0.05115500 0.00515400 1.67618200

N 0.06076900 -0.01089400 -1.18778900

C 0.63889000 -0.13337200 -2.55354100

H 1.09386900 -1.11742500 -2.66058700

H -0.13789900 0.00928300 -3.31675200

C -0.90491900 -1.13267800 -0.95810500

H -1.56683000 -1.23764400 -1.82570200

C -1.80782300 -0.91340400 0.29643600

C -1.03719200 -1.00482000 1.62591300

H -1.74507500 -0.84188500 2.44704700

H -0.61033200 -1.99637900 1.75824600

C -2.46458200 0.46869600 0.16084200

H -2.94405300 0.54201300 -0.82212800

O -3.42092700 0.59797400 1.19580700

C 0.78749400 -0.12447800 2.95593200

H 1.25209000 -1.11062700 2.99601700

N 1.79064000 1.66694800 -0.06546400

C 0.82011800 2.25295900 -0.80493400

C 3.07947000 3.67990600 -0.19152800

H 3.98340100 4.21390300 0.08076200

C 2.89403100 2.36769800 0.23822300

H 3.62486600 1.84359400 0.84322500

C 0.94164800 3.54987400 -1.28507300

H 0.14690200 3.98668300 -1.88034700

C 2.09263600 4.27793000 -0.97341800

H 2.21145600 5.29510600 -1.33289900

H 1.42732700 0.60835300 -2.67584100

C -0.41888800 1.39331300 -0.97723700

H -0.99793400 1.72349800 -1.85110400

C -1.34521800 1.52598400 0.27799000

C -0.58743400 1.34275100 1.60714800

H -1.30724300 1.45471500 2.42853000

H 0.17284000 2.11138300 1.72439600

H -4.02730200 1.31352800 0.94661700

H 1.58044500 0.62423600 2.98383300

H 0.11416400 0.00820600 3.81388100

O 3.22099500 -0.60816100 1.21792800

C -2.89065300 -2.00302600 0.30757800

O -3.00387400 -2.84045900 1.17111100

O -3.67591800 -1.92047400 -0.78187500

C -1.91847000 2.94927100 0.27216000

O -1.38247200 3.89840500 0.79569700

O -3.07624800 3.02278200 -0.41612000

C -3.66338400 4.34520700 -0.51009000

H -4.57634600 4.21542100 -1.08976200

H -3.88196600 4.73016700 0.48849200

H -2.97351900 5.02539700 -1.01480400

C -4.75602200 -2.88108000 -0.84141900

H -5.41922200 -2.74948400 0.01654800

H -5.27874900 -2.67284800 -1.77427400

H -4.35737400 -3.89861100 -0.83444300

C 3.83816700 -0.72979900 0.10461200

O 3.15346500 -0.60546400 -0.96170300

C 5.31895200 -0.98916500 0.05151800

H 5.83150100 -0.08846100 -0.30596700

H 5.70051100 -1.25109200 1.04001100

H 5.53163700 -1.78811600 -0.66493100

[(L^2^)Fe^IV^=O]^1+^ (^3^P_IS_)

Fe 1.59167200 -0.08785700 0.20988500

N 1.36298200 -2.03958700 0.00138800

C 0.24281400 -2.38274900 -0.68024300

C 2.05268800 -4.33032600 0.00866500

H 2.78864700 -5.07069200 0.30253300

C 2.24917900 -2.99122000 0.33676700

H 3.11920000 -2.63832300 0.87842800

C -0.01106800 -3.69650300 -1.05431600

H -0.91427300 -3.93622600 -1.60573100

C 0.90797000 -4.68840700 -0.70217600

H 0.73069200 -5.72233900 -0.98089500

N -0.10973500 0.02545900 1.77186300

N 0.05213800 -0.00174500 -1.15142500

C 0.64954400 -0.04779700 -2.51533400

H 1.23571700 -0.96032500 -2.61995900

H -0.13884800 -0.01223300 -3.27905900

C -0.74376800 -1.24931100 -0.91304000

H -1.35314100 -1.46318700 -1.79908900

C -1.72585900 -1.14257900 0.29834000

C -1.02394100 -1.12843400 1.66893400

H -1.79272200 -1.09268100 2.45193300

H -0.45646900 -2.04710100 1.81341600

C -2.54865500 0.14198100 0.11261200

H -2.97999200 0.14784900 -0.89529300

O -3.56792100 0.14572800 1.09386800

C 0.63168600 -0.00494700 3.04674700

H 1.22441200 -0.92229700 3.09110000

N 1.58996600 1.87610700 -0.01613700

C 0.52872400 2.34170000 -0.71990500

C 2.58472000 4.05069200 -0.08432900

H 3.41194800 4.69556600 0.19137800

C 2.59379400 2.71176500 0.29679800

H 3.40410900 2.26350100 0.86014300

C 0.46383900 3.66056400 -1.15106400

H -0.39302300 4.00475000 -1.71967600

C 1.50729700 4.53071700 -0.82747700

H 1.47552000 5.56624600 -1.15100400

H 1.33293200 0.79195200 -2.63832600

C -0.59272800 1.33415700 -0.93182800

H -1.17282000 1.60601600 -1.82512800

C -1.58979200 1.34185800 0.27846100

C -0.88966400 1.26968000 1.64978400

H -1.66051800 1.32162800 2.43237000

H -0.22741900 2.12452000 1.77957700

H -4.24958500 0.77361800 0.80593600

H 1.31667100 0.84609900 3.08041200

H -0.04440300 0.03430900 3.91359100

O 3.42225400 -0.18114700 1.00174000

C -2.65989100 -2.36279600 0.26410600

O -2.70140300 -3.21098600 1.12347200

O -3.40063000 -2.38011500 -0.85925400

C -2.35107100 2.67340900 0.22447600

O -1.97579700 3.69064600 0.75999700

O -3.47164200 2.58424200 -0.52121300

C -4.22839700 3.81323500 -0.66128300

H -5.08603500 3.55526100 -1.28114800

H -4.54524000 4.17233300 0.32041300

H -3.61432100 4.57754200 -1.14305500

C -4.34755300 -3.46929900 -0.96083700

H -5.05897300 -3.42225100 -0.13325500

H -4.85072200 -3.32840900 -1.91672700

H -3.82506900 -4.42873200 -0.93094700

C 4.09257000 -0.23342700 -0.10745600

O 3.49456900 -0.22114100 -1.20839000

C 5.60004700 -0.27458500 -0.01791700

H 5.97753700 0.74421600 0.13298800

H 5.91918700 -0.87351000 0.83904400

H 6.02573900 -0.66652400 -0.94347400

[(L^2^)Fe^IV^=O]^1+^ (^5^P_HS_)

Fe 1.68386900 -0.14032800 0.37901000

N 1.22045500 -2.23323500 -0.07871200

C 0.09588100 -2.42120100 -0.79490200

C 1.78267000 -4.54281700 -0.35505600

H 2.47483600 -5.35489500 -0.16073400

C 2.04996300 -3.26493900 0.13167900

H 2.94111300 -3.03734100 0.70859700

C -0.23973600 -3.66442200 -1.32274100

H -1.15301500 -3.78136200 -1.89737200

C 0.61711100 -4.74369900 -1.09468200

H 0.37937800 -5.72472800 -1.49420600

N -0.05048200 0.01713500 1.80188400

N 0.05349400 -0.00108300 -1.13375700

C 0.69075500 -0.06814000 -2.47374900

H 1.24886600 -1.00013800 -2.56120400

H -0.06392300 -0.00782900 -3.27096400

C -0.80541000 -1.20161200 -0.92556900

H -1.46242100 -1.33360600 -1.79518200

C -1.73704000 -1.08635000 0.32566300

C -1.00014200 -1.11165200 1.67873700

H -1.75369200 -1.06783100 2.47532700

H -0.45262300 -2.04540900 1.79758200

C -2.52895400 0.22283500 0.18570000

H -3.00429200 0.24907900 -0.80149500

O -3.50434800 0.25440700 1.21048000

C 0.63123500 -0.03492700 3.11406300

H 1.18826700 -0.97250500 3.19080000

N 1.59242600 1.99744500 -0.09567600

C 0.53309900 2.37033400 -0.83829800

C 2.60623000 4.13287800 -0.46665800

H 3.44434800 4.79997900 -0.29679700

C 2.60820700 2.85343300 0.08397600

H 3.43187200 2.48067500 0.68505900

C 0.46110300 3.62532200 -1.43386200

H -0.40222100 3.89654800 -2.03284000

C 1.51342000 4.52241200 -1.24106300

H 1.48080500 5.50877800 -1.69320400

H 1.40583800 0.74781300 -2.57612900

C -0.57943800 1.33535400 -0.94195200

H -1.20744200 1.56988600 -1.81538500

C -1.52065800 1.38531700 0.31145000

C -0.78497100 1.29252200 1.66419600

H -1.53721700 1.38109100 2.46079300

H -0.08459200 2.11897100 1.77148600

H -4.17445700 0.90858500 0.95579500

H 1.34114800 0.79373100 3.18235600

H -0.08586300 0.03014200 3.94491900

O 3.48184900 -0.30532000 1.26092700

C -2.70469400 -2.28072300 0.30870300

O -2.74180000 -3.13865500 1.15867200

O -3.48290800 -2.26368600 -0.78922500

C -2.22922600 2.74671700 0.28376300

O -1.78697000 3.75034100 0.79311100

O -3.38775800 2.69983000 -0.40573600

C -4.09728800 3.95907900 -0.51901800

H -4.99163300 3.73572600 -1.09923200

H -4.35527300 4.33472300 0.47369700

H -3.47300100 4.69505200 -1.03081800

C -4.45644900 -3.33022000 -0.87447200

H -5.13708300 -3.28272300 -0.02142100

H -4.99010800 -3.16221100 -1.80927200

H -3.95394000 -4.30074300 -0.87904000

C 4.15702100 -0.36083100 0.15097000

O 3.55582200 -0.30044500 -0.94885800

C 5.65542000 -0.49690300 0.24155400

H 6.06594200 0.34756600 0.80547400

H 5.90746400 -1.40432100 0.80070300

H 6.10133000 -0.53438800 -0.75327800

[(L^1^)Fe^V^=O]^2+^ (^2^R_LS_)

Fe 1.07913600 -0.22504700 -0.17392500

N 0.54214900 -2.14027300 -0.26807300

C -0.68989000 -2.30799200 -0.80125800

C 0.93348800 -4.49064600 -0.41342700

H 1.59991100 -5.32867700 -0.24113300

C 1.34042300 -3.20371700 -0.07845500

H 2.31304400 -2.99618700 0.34669400

C -1.15743100 -3.56308700 -1.17348900

H -2.15503800 -3.65458300 -1.59018700

C -0.33068300 -4.67231400 -0.97891000

H -0.66851300 -5.66372700 -1.26416500

N -0.22375900 0.05001200 1.65084800

N -0.57506500 0.07487000 -1.20440200

C -0.30888700 0.00196600 -2.68300800

H 0.09529800 -0.97835300 -2.92797900

H -1.24780700 0.17102700 -3.21989600

C -1.52018700 -1.05063900 -0.84228500

H -2.28239300 -1.11572500 -1.62246900

C -2.26435900 -0.85603200 0.50862000

C -1.31867200 -0.96411700 1.71336000

H -1.90070000 -0.80638200 2.63026300

H -0.86333000 -1.95340700 1.77717500

C -2.91852000 0.54436500 0.50201400

H -3.55078600 0.65007300 -0.38543500

O -3.67676600 0.70054000 1.69177700

C 0.60888900 -0.08691900 2.87738700

H 1.05167200 -1.08262000 2.90619400

N 1.28418300 1.75758800 -0.32068600

C 0.19473200 2.34406300 -0.87153500

C 2.50014300 3.80116200 -0.55058600

H 3.42760600 4.34337700 -0.40284000

C 2.41507900 2.46666600 -0.16553100

H 3.25763100 1.95062200 0.27344400

C 0.21319200 3.66795100 -1.29359200

H -0.68477900 4.09929900 -1.72356600

C 1.38663500 4.40903200 -1.13265300

H 1.43023700 5.44391000 -1.45749300

H 0.42360300 0.75971400 -2.95443400

C -1.03876200 1.47829100 -0.87960400

H -1.72807400 1.79583000 -1.66593200

C -1.79584100 1.60764900 0.47114500

C -0.87029400 1.39632200 1.67724300

H -1.46613100 1.48779200 2.59419000

H -0.08384800 2.15120600 1.71713100

H -4.59027700 0.93056900 1.46785900

H 1.40984700 0.65211700 2.85748400

H -0.00769600 0.06360200 3.77189800

O 2.07201500 -0.42271600 -1.43992500

O 2.67908500 -0.64596500 1.08904300

C 3.93942000 -0.57045500 0.90116500

O 4.42206100 0.37616800 1.58983400

C 4.75206600 -1.44237300 0.01961700

H 4.74168800 -2.46481400 0.41670200

H 4.29640900 -1.44620300 -0.97681900

H 5.78597900 -1.09117600 -0.02876400

C -2.40527700 3.02077700 0.56404500

H -1.61006800 3.76571100 0.71358300

H -3.06064800 3.04618200 1.44222400

C -3.34836000 -1.94572100 0.63695100

H -2.88016400 -2.92216700 0.83024900

H -3.97540300 -1.69383000 1.49938100

O -4.08782500 -1.95952700 -0.57672700

H -4.89818900 -2.47852200 -0.45523900

O -3.11761000 3.25868900 -0.64238000

H -3.65827400 4.05829600 -0.54613600

[(L^1^)Fe^V^=O]^2+^ (^4^R_HS_)

Fe -1.14570100 0.14090700 -0.11217200

N -0.71038600 2.06541100 -0.37811600

C 0.52518400 2.31392700 -0.87111800

C -1.31354200 4.35238800 -0.65711900

H -2.06474800 5.12887200 -0.56308400

C -1.62248400 3.05001400 -0.28198200

H -2.59722100 2.76839200 0.09326600

C 0.89208800 3.59222200 -1.27405900

H 1.89362300 3.75653700 -1.65778600

C -0.04091900 4.62677400 -1.16436000

H 0.21924300 5.63265400 -1.47906800

N 0.21512900 -0.00643100 1.67793700

N 0.58161200 -0.07062700 -1.18935500

C 0.28894900 -0.05127300 -2.66162200

H -0.18391500 0.89294900 -2.92795700

H 1.22595100 -0.16829200 -3.21588400

C 1.44500300 1.12217700 -0.86108800

H 2.20780800 1.21915200 -1.63823800

O 3.92949500 2.23275800 -0.60314100

H 4.69608400 2.81687300 -0.49249100

C 2.18753600 1.02011200 0.49884500

C 1.22841300 1.09133900 1.69438000

H 1.81929200 1.01303500 2.61560100

H 0.69387700 2.04234100 1.72503500

C 3.18595000 2.19111500 0.60678400

H 2.64418500 3.13360700 0.77584000

H 3.82423000 2.00814700 1.47851600

C 2.94293500 -0.32915300 0.53596800

H 3.59220900 -0.41183000 -0.34174300

O 3.69279300 -0.40502700 1.73815000

C -0.58526800 0.09438200 2.93149000

H -1.10976800 1.04827200 2.95412700

N -1.17692000 -1.83317400 -0.36381600

C -0.02235000 -2.37000100 -0.82799200

C -2.28359600 -3.92587900 -0.63545000

H -3.19862400 -4.50278500 -0.55822500

C -2.29628500 -2.57876200 -0.28989400

H -3.20091300 -2.07126800 0.01296000

C 0.04854600 -3.70525400 -1.20431100

H 0.99192600 -4.09956800 -1.56756400

C -1.09764600 -4.49779700 -1.09977400

H -1.06857900 -5.54266100 -1.39294500

H -0.39340300 -0.86285700 -2.90967700

C 1.14798400 -1.42396100 -0.83579900

H 1.86414400 -1.70905600 -1.61123600

O 3.31789000 -3.06546400 -0.56075500

H 3.93025100 -3.80831200 -0.44112900

C 1.90043600 -1.47143900 0.52052000

C 0.95169300 -1.30554200 1.71390500

H 1.54428300 -1.35047800 2.63621600

H 0.21463700 -2.10959600 1.75509000

C 2.60727100 -2.83668200 0.64803700

H 1.86642600 -3.62864400 0.83268400

H 3.27347800 -2.78905500 1.51680300

H 4.63243700 -0.51559000 1.53189800

H -1.32091400 -0.70868500 2.95852000

H 0.07837700 0.01606800 3.80073700

O -2.14832100 0.26151700 -1.44347800

O -2.49720400 0.37639200 1.06513900

C -3.81462300 0.34646200 0.76662800

O -4.23920200 0.00985700 -0.32401500

C -4.68274400 0.73968300 1.93642600

H -4.53496100 0.02819600 2.75604800

H -4.38564700 1.72752100 2.30371000

H -5.73037900 0.74685300 1.63419600

[(L^2^)Fe^V^=O]^2+^ (^2^R_LS_)

Fe 1.47600100 -0.19822100 -0.20356500

N 1.05952400 -2.14224500 -0.28461700

C -0.13671700 -2.39669800 -0.86234900

C 1.56698000 -4.47476500 -0.29383600

H 2.26182700 -5.26977600 -0.04655000

C 1.89727900 -3.15396100 -0.00786300

H 2.83665600 -2.87903300 0.45204800

C -0.52523300 -3.68844400 -1.19271200

H -1.49112700 -3.86093700 -1.65515700

C 0.34153100 -4.74588300 -0.90427100

H 0.06162000 -5.76532300 -1.15001600

N 0.10451500 0.00689200 1.56166200

N -0.15846600 -0.00786700 -1.30634700

C 0.16721500 -0.07819200 -2.77316000

H 0.64021200 -1.03417200 -2.98936200

H -0.75857000 0.02617500 -3.34872100

C -1.03973500 -1.19123600 -0.96482700

H -1.77004600 -1.31430500 -1.77000600

C -1.82745400 -1.02476500 0.36456300

C -0.92367700 -1.07444600 1.60406100

H -1.55239200 -0.95245300 2.49240200

H -0.42821600 -2.03879500 1.69328000

C -2.58180200 0.31379300 0.31306200

H -3.17481000 0.36005700 -0.60753900

O -3.40438300 0.37278500 1.45530300

C 0.90118400 -0.06479200 2.81867000

H 1.40473100 -1.02986100 2.87311300

N 1.56075500 1.78786400 -0.35065800

C 0.47208600 2.30829200 -0.96564000

C 2.66904900 3.89632100 -0.52504700

H 3.55141800 4.49335200 -0.32281400

C 2.63691800 2.56141100 -0.13340000

H 3.47912100 2.09738100 0.36050400

C 0.44670000 3.62360500 -1.40757100

H -0.43649700 4.01450700 -1.89981500

C 1.56367000 4.43227500 -1.18507400

H 1.56608900 5.46515900 -1.51818000

H 0.86239500 0.72014200 -3.02553000

C -0.71333500 1.37262700 -1.01297400

H -1.39072500 1.64081800 -1.83170100

C -1.52429200 1.44101300 0.31832400

C -0.62527700 1.30744300 1.55736400

H -1.26334100 1.36424700 2.44720400

H 0.08994300 2.12522500 1.61155300

H -4.10915200 1.02072100 1.29247700

H 1.65056100 0.72651800 2.81956500

H 0.24274400 0.05094600 3.68768700

O 2.51431800 -0.34338800 -1.44188800

O 3.05591800 -0.49727400 1.10416800

C -2.83261900 -2.19017600 0.46998700

O -2.77281000 -3.04293800 1.32247000

O -3.73093400 -2.13785200 -0.52050700

C -2.18071400 2.83505300 0.38062500

O -1.60544100 3.81456700 0.79620900

O -3.42770400 2.82306500 -0.10632700

C -4.12464600 4.10541700 -0.10710900

H -5.10555600 3.89640900 -0.53001700

H -4.20406000 4.48136200 0.91464300

H -3.57604700 4.82258800 -0.72061600

C -4.77547900 -3.15183600 -0.48109600

H -5.34922200 -3.04882200 0.44187200

H -5.39660000 -2.95837100 -1.35391300

H -4.33045900 -4.14821800 -0.52478800

C 4.31539100 -0.37361600 0.95306100

O 4.74610700 0.58618100 1.66192400

C 5.19190400 -1.20530800 0.09355200

H 5.23118200 -2.22333500 0.50045900

H 4.75788300 -1.24262900 -0.91152900

H 6.20597500 -0.79832000 0.06300700

[(L^2^)Fe^V^=O]^2+^ (^4^R_HS_)

Fe 1.53168800 0.07448100 -0.12202100

N 1.42421000 -1.89744500 -0.36618200

C 0.25927800 -2.34919000 -0.88943200

C 2.35520700 -4.08722700 -0.50067700

H 3.20753300 -4.73878100 -0.34254200

C 2.46509000 -2.73402200 -0.19760600

H 3.38937700 -2.29829200 0.15291500

C 0.09442400 -3.68451300 -1.22967300

H -0.84861800 -4.01991900 -1.64749000

C 1.15520600 -4.57078300 -1.02297500

H 1.04884700 -5.61995800 -1.28000300

N 0.10038800 0.04092100 1.58700300

N -0.15483400 -0.00499200 -1.29643500

C 0.21468500 -0.01516400 -2.75117100

H 0.84458900 -0.87840600 -2.96051000

H -0.69701900 -0.06286300 -3.35633500

C -0.83284800 -1.31300300 -0.96835300

H -1.53485100 -1.55009500 -1.77424200

C -1.63174200 -1.28909300 0.36111800

C -0.72843300 -1.20130700 1.59713200

H -1.36759600 -1.20721400 2.48601000

H -0.07148300 -2.06612600 1.67042600

C -2.59654500 -0.09277900 0.32296400

H -3.19813000 -0.14147900 -0.59206700

O -3.40454100 -0.17550900 1.47337500

C 0.84732400 0.09117200 2.87791900

H 1.51679800 -0.76550200 2.94268600

N 1.26315600 2.02270500 -0.39994000

C 0.08838500 2.37181800 -0.97448800

C 2.07464200 4.24972300 -0.63589500

H 2.87822400 4.96199100 -0.48447800

C 2.24448600 2.92869200 -0.23868900

H 3.16219200 2.56984600 0.20679100

C -0.13237500 3.66967400 -1.41405900

H -1.07887400 3.93287500 -1.87159000

C 0.87325000 4.62359500 -1.24026600

H 0.72003900 5.64438800 -1.57561700

H 0.77423200 0.88749000 -2.99206300

C -0.93208500 1.25863300 -1.00921300

H -1.64641900 1.41286300 -1.82688700

C -1.73866200 1.19338800 0.32424700

C -0.82790400 1.20887300 1.56158100

H -1.46877600 1.17136100 2.45022200

H -0.24872200 2.12901600 1.61073500

H -4.20407900 0.35565700 1.32541500

H 1.43815200 1.00465100 2.92195000

H 0.13807700 0.06732100 3.71312600

O 2.60645300 0.10863200 -1.41575100

O 2.85849300 0.20199500 1.10291600

C -2.43073500 -2.60581000 0.46077400

O -2.22630600 -3.44192800 1.30720400

O -3.32822000 -2.69563900 -0.52693900

C -2.61061900 2.46444900 0.38980200

O -2.17922700 3.53457400 0.75474700

O -3.86010300 2.24065900 -0.03121900

C -4.75481400 3.39502200 -0.02222100

H -5.70779800 3.01910500 -0.38972000

H -4.84478400 3.78151900 0.99462500

H -4.35859300 4.17234300 -0.67816400

C -4.19292000 -3.86800600 -0.49261100

H -4.77380300 -3.86515400 0.43157000

H -4.83877800 -3.77357200 -1.36373100

H -3.59053100 -4.77752700 -0.54264100

C 4.17077200 0.06914900 0.83408000

O 4.59454900 -0.25281800 -0.26441000

C 5.04615000 0.32888300 2.03484000

H 4.83374600 1.32496900 2.43705200

H 4.81204900 -0.39774900 2.82028000

H 6.09659600 0.24963600 1.75353800

[(L^1^)Fe^V^=O]^2+^ (^2^TS1_LS_)

Fe -0.63434700 0.27122700 0.02828500

N 0.00718700 2.15561800 -0.11914700

C 1.15529200 2.27533500 -0.82922100

C -0.30982700 4.51454700 -0.17094100

H -0.90640700 5.37475700 0.11252800

C -0.71476200 3.23838300 0.20842300

H -1.61403300 3.04319500 0.77938500

C 1.60600000 3.52068100 -1.25098200

H 2.53403100 3.58576900 -1.80968400

C 0.85981200 4.65608800 -0.92107700

H 1.19050400 5.63870800 -1.24317300

N 0.93729400 -0.00517600 1.66396300

N 0.93715600 -0.10468200 -1.22733700

C 0.52439400 -0.04293000 -2.67023200

H 0.15338500 0.95451800 -2.89971000

H 1.39375000 -0.27151600 -3.29563500

C 1.94088000 0.99520800 -0.99438600

H 2.59158200 1.05706700 -1.87003000

O 4.56124800 1.77704500 -1.10537600

H 5.41390600 2.23928100 -1.10212600

C 2.85672000 0.77447900 0.23901400

C 2.08669100 0.94496900 1.54840100

H 2.77292700 0.75195800 2.38179100

H 1.70195300 1.95805900 1.66934400

C 4.00089600 1.80837000 0.19989500

H 3.61244100 2.80862200 0.44318900

H 4.72943700 1.53110000 0.96946600

C 3.43104000 -0.66085600 0.19205100

H 3.94290400 -0.82012600 -0.76254600

O 4.32300500 -0.82041800 1.28441400

C 0.33225800 0.22045700 3.00967300

H -0.14433300 1.20030100 3.02735900

N -0.93987400 -1.69844300 -0.21277500

C 0.10464000 -2.32911900 -0.80835400

C -2.28383700 -3.64694800 -0.51516900

H -3.25107300 -4.12218700 -0.39562100

C -2.12498500 -2.32709600 -0.10259800

H -2.94873100 -1.76172300 0.30193500

C 0.00720400 -3.64381100 -1.24479200

H 0.87260300 -4.11156900 -1.70270700

C -1.20414100 -4.32125400 -1.08263500

H -1.30603100 -5.34939100 -1.41603200

H -0.26712300 -0.76666200 -2.85737400

C 1.37028400 -1.52013200 -0.92926300

H 1.95917600 -1.88022700 -1.77721200

O 3.38765100 -3.39792200 -0.87316500

H 3.87289200 -4.23619200 -0.81865600

C 2.26147600 -1.66479500 0.32761200

C 1.48383200 -1.39370700 1.62134900

H 2.16297900 -1.52543500 2.47313900

H 0.64740400 -2.08314400 1.74647400

C 2.80613900 -3.10565600 0.39016500

H 1.99257200 -3.80535300 0.63014600

H 3.54123000 -3.15218600 1.20238000

H 5.16199600 -1.18674500 0.96869500

H -0.40412000 -0.55192400 3.20931200

H 1.12131000 0.18310700 3.76966000

O -1.64821300 0.52178500 -1.30723700

O -1.89111500 0.81509900 1.27333100

C -2.61177000 0.06522300 2.13565700

O -2.35139900 -1.09263400 2.39859900

C -3.75726000 0.83445700 2.75219000

H -4.64882400 0.63864900 2.14676500

H -3.94003600 0.45536400 3.75984900

H -3.56805800 1.91014700 2.77881300

H -2.78563500 1.09323500 -1.03466800

C -3.92697500 1.57111800 -0.90410300

H -4.03038500 2.25134800 -1.74946100

H -3.87782700 2.04868200 0.07256400

C -4.80894800 0.35670800 -0.91935700

O -4.74490400 -0.52440200 -0.08133800

O -5.64462500 0.36235700 -1.95886500

H -6.21918500 -0.42822000 -1.90736100

[(L^1^)Fe^V^=O]^2+^ (^4^TS1_HS_)

Fe -0.63495500 0.26592000 0.06631800

N 0.00997500 2.16017400 -0.12188700

C 1.15712500 2.28911400 -0.83046400

C -0.33564100 4.51654500 -0.19013700

H -0.94349400 5.37083000 0.08761800

C -0.72602300 3.23868100 0.19715400

H -1.62775800 3.04428100 0.76523400

C 1.58992400 3.53863400 -1.26397600

H 2.51207600 3.61438100 -1.83092700

C 0.83063100 4.66694000 -0.94292500

H 1.14761400 5.65076200 -1.27523500

N 0.96630900 -0.01900000 1.68713400

N 0.92917100 -0.08760800 -1.23058600

C 0.50427800 -0.00921000 -2.66904700

H 0.12550900 0.98769500 -2.88855000

H 1.37191200 -0.22373900 -3.30243000

C 1.94257000 1.00927900 -0.99648400

H 2.59097900 1.07135100 -1.87468700

O 4.56675700 1.79967700 -1.11288400

H 5.41594200 2.26893900 -1.10824600

C 2.86677200 0.78073800 0.23271600

C 2.11094600 0.93622900 1.55563600

H 2.81139300 0.74097300 2.37715800

H 1.72652100 1.94846000 1.69046000

C 4.01041000 1.81821800 0.19410300

H 3.62480000 2.81659900 0.44797500

H 4.74481500 1.53632200 0.95613000

C 3.44719500 -0.65365400 0.16635800

H 3.95140100 -0.80378700 -0.79340300

O 4.35263400 -0.82639100 1.24566500

C 0.40189800 0.18343100 3.05385300

H -0.06853400 1.16624800 3.11119700

N -0.93226300 -1.69089200 -0.25276500

C 0.11556800 -2.32392900 -0.83747800

C -2.28663500 -3.63020100 -0.55200400

H -3.25519300 -4.10310900 -0.43328100

C -2.12459100 -2.30962700 -0.14375000

H -2.95050600 -1.73891000 0.25575200

C 0.00785000 -3.63733800 -1.27888900

H 0.86634100 -4.11187100 -1.74256200

C -1.20850900 -4.30674700 -1.11997600

H -1.31618800 -5.33271300 -1.45861300

H -0.28374100 -0.73342700 -2.86731800

C 1.37725000 -1.50597700 -0.95040000

H 1.96583600 -1.85348600 -1.80457200

O 3.38879600 -3.39192500 -0.92861400

H 3.89435500 -4.21869100 -0.87836100

C 2.27945900 -1.66185000 0.30280100

C 1.51735900 -1.40589700 1.61211400

H 2.20997500 -1.54864500 2.45187100

H 0.68755100 -2.10319600 1.74314100

C 2.83051100 -3.10354700 0.34589100

H 2.02620900 -3.80892900 0.59905300

H 3.58293700 -3.15171100 1.14120000

H 5.20851300 -1.13179800 0.90977900

H -0.32947400 -0.59116600 3.26686600

H 1.21159000 0.13227100 3.79155600

O -1.71798000 0.55121800 -1.26109700

O -1.94257000 0.75536900 1.26134600

C -2.56113800 0.01657800 2.21432800

O -2.29497600 -1.15079000 2.42428500

C -3.62756100 0.79330400 2.94919600

H -3.36122100 1.84684900 3.06704300

H -4.55270000 0.72770500 2.36531700

H -3.80139800 0.33302600 3.92370000

H -2.82305400 1.10239500 -1.04477300

C -4.02167900 1.58943200 -1.00059100

H -4.11303600 2.19063200 -1.90475200

H -3.99217900 2.15349200 -0.06901600

C -4.88750400 0.37193700 -0.92593900

O -4.80111300 -0.46065900 -0.03855200

O -5.74203300 0.29732800 -1.94916800

H -6.30020800 -0.49779200 -1.83224900

[(L^2^)Fe^V^=O]^2+^ (^2^TS1_LS_)

Fe 1.02774700 -0.31492700 -0.00356400

N 0.45961600 -2.22085600 -0.14776900

C -0.66523400 -2.39602000 -0.88354700

C 0.89090900 -4.56181300 -0.18830300

H 1.51847700 -5.39254900 0.11529000

C 1.22353900 -3.26743900 0.19952800

H 2.09908400 -3.02849800 0.79002100

C -1.03509700 -3.65923900 -1.32423100

H -1.93825300 -3.78649700 -1.90984000

C -0.24497200 -4.75792200 -0.97448900

H -0.52067100 -5.75401400 -1.30592500

N -0.55092400 -0.09608700 1.61325700

N -0.55655400 -0.00384300 -1.28202100

C -0.12955100 -0.04642300 -2.72134600

H 0.28506300 -1.02704100 -2.94835400

H -1.00063700 0.14913800 -3.35617900

C -1.51166800 -1.15039000 -1.05393900

H -2.15281300 -1.23932100 -1.93853200

O -4.64462300 -1.63471000 -0.40054800

C -2.43321000 -0.94060500 0.18540600

C -1.65981100 -1.08970200 1.49747500

H -2.36078200 -0.91578500 2.32165300

H -1.25861300 -2.09373600 1.61355000

C -3.06844000 0.46895300 0.12988700

H -3.56732000 0.61952100 -0.83443000

O -3.95717400 0.67044500 1.20420300

C 0.05422700 -0.29912300 2.96407300

H 0.56462200 -1.26151300 2.98615100

N 1.25314000 1.66589400 -0.24022600

C 0.18799700 2.25767100 -0.83912200

C 2.49635300 3.69038200 -0.46651100

H 3.43462900 4.21236900 -0.31559900

C 2.40202900 2.35118600 -0.09645900

H 3.24924800 1.81746700 0.30211000

C 0.22238700 3.58617000 -1.23678900

H -0.65092600 4.02843100 -1.70410400

C 1.39151900 4.32390100 -1.02986400

H 1.44094500 5.36679900 -1.32679500

H 0.63399900 0.70910400 -2.89826600

C -1.04185200 1.39543700 -0.98381600

H -1.63960400 1.73666500 -1.83462000

O -3.27801900 3.17334200 -0.74042000

C -1.93341000 1.49662100 0.27625600

C -1.15005600 1.27026300 1.57458700

H -1.83771400 1.38665700 2.41867000

H -0.35505600 2.00345600 1.69828200

H -4.81508700 0.27961700 0.97104100

H 0.75792900 0.50196700 3.16764300

H -0.74180100 -0.28882600 3.71707200

O 2.05130800 -0.52392800 -1.33968400

O 2.30036900 -0.80870700 1.23849600

C 2.99424600 -0.04087300 2.11056500

O 2.69820900 1.10574500 2.37903000

C 4.15677400 -0.78312100 2.72834800

H 3.99835400 -1.86390400 2.74528900

H 5.04674100 -0.55657400 2.13146600

H 4.32060800 -0.40705500 3.74042700

H 3.21978600 -1.05534000 -1.06588800

C 4.37185200 -1.48456500 -0.93434700

H 4.51398300 -2.14704100 -1.78826400

H 4.33762300 -1.98205400 0.03337700

C 5.20780600 -0.23686500 -0.92050400

O 5.07989700 0.64243800 -0.08809500

O 6.08294400 -0.21448900 -1.92637200

H 6.62707900 0.59594900 -1.85516500

C -3.50162300 -2.05187100 0.15532500

O -3.29439700 -3.17173300 0.56465700

C -2.52683500 2.91979600 0.33907200

O -2.30753100 3.69034900 1.24095500

C -3.95022000 4.46537300 -0.75025900

H -4.49964600 4.49605400 -1.68956600

H -4.62600700 4.53185900 0.10445000

H -3.21320400 5.26943800 -0.69565100

C -5.71102800 -2.62756800 -0.49118000

H -6.54500700 -2.10763000 -0.95891600

H -5.37923100 -3.46922800 -1.10200400

H -5.97181700 -2.97883800 0.50890000

[(L^2^)Fe^V^=O]^2+^ (^4^TS1_HS_)

Fe -0.90090900 0.39366600 0.19987600

N -0.29392700 2.27542600 -0.13362000

C 0.86626900 2.36076700 -0.82868100

C -0.53210800 4.64981500 -0.24194400

H -1.11520100 5.52965000 0.00644400

C -0.99282400 3.39279000 0.13726000

H -1.93798900 3.26218000 0.64220700

C 1.37057800 3.58023000 -1.25753000

H 2.30065500 3.61286200 -1.81479400

C 0.66435500 4.74710200 -0.95178600

H 1.03980100 5.71387900 -1.27197600

N 0.62077500 0.01404300 1.68310800

N 0.53358400 -0.00606200 -1.18549600

C -0.01064100 0.11821100 -2.57610800

H -0.40624800 1.12105800 -2.72556100

H 0.78671200 -0.08394100 -3.29990000

C 1.59248500 1.04816700 -0.99825000

H 2.22943400 1.07045200 -1.88847900

O 4.41278000 1.65912300 -0.89253400

C 2.50621900 0.79256100 0.22925100

C 1.76877500 0.96419400 1.56270800

H 2.48021200 0.77611600 2.37304200

H 1.40276100 1.98123500 1.68636700

C 3.07480400 -0.63083100 0.11432700

H 3.54570900 -0.75308600 -0.86752200

O 4.01055200 -0.79477500 1.15539500

C 0.03825200 0.18157900 3.04794300

H -0.35042500 1.19380300 3.15209700

N -1.27716500 -1.54049400 -0.02748200

C -0.34307800 -2.21270300 -0.74696800

C -2.63603600 -3.49960500 0.06121100

H -3.54893700 -3.97057700 0.40848000

C -2.39366500 -2.16678000 0.38304100

H -3.10704700 -1.57738600 0.93883100

C -0.53972500 -3.53182600 -1.12656400

H 0.21495200 -4.04781900 -1.70896900

C -1.70613400 -4.18696200 -0.71744100

H -1.87647100 -5.22163800 -0.99786600

H -0.81915600 -0.59863300 -2.71266200

C 0.93911900 -1.43973400 -0.96448200

H 1.46709300 -1.79780700 -1.85655400

O 3.53721200 -3.22625600 -0.37784500

C 1.89183400 -1.61562500 0.25972400

C 1.17460400 -1.37001300 1.59601700

H 1.90568900 -1.50682700 2.40085900

H 0.37365500 -2.08901800 1.75260200

H 4.61151700 -1.51930000 0.91711500

H -0.77843600 -0.52531100 3.18352100

H 0.81416600 0.00488300 3.80115000

O -2.18623500 0.90048700 -1.05175800

O -2.17691600 0.63092800 1.55626100

C -3.32909500 1.20346600 1.37879400

O -3.68433900 1.73224500 0.30517000

C -4.27976400 1.18983500 2.54812700

H -3.73863600 1.06827500 3.48784100

H -4.88345900 2.09981400 2.55900200

H -4.95114000 0.33574000 2.39564800

H -3.07842200 0.14562700 -1.41699500

C -4.07793400 -0.55782300 -1.91979900

H -3.65837700 -1.56145800 -1.96955000

H -4.24197300 -0.07370200 -2.88158000

C -5.17510400 -0.41202300 -0.91587600

O -5.15947800 -0.93640900 0.18720100

O -6.15547500 0.37263000 -1.36383300

H -6.84745800 0.43488800 -0.67583000

C 2.37310300 -3.07989200 0.26666100

O 1.74158200 -3.97832600 0.77434000

C 3.65452400 1.82150300 0.19816700

O 3.80711300 2.67154900 1.04178700

C 4.06447700 -4.58531200 -0.44227200

H 5.00005500 -4.50233500 -0.99231800

H 4.22962200 -4.96420300 0.56809100

H 3.35648400 -5.23112900 -0.96517000

C 5.57130300 2.53556300 -0.99258300

H 6.05405900 2.26740400 -1.93072500

H 5.25044600 3.57948600 -0.99669100

H 6.23520100 2.36163900 -0.14365000

[(L^1^)Fe^V^=O]^2+^ (^2^INT_LS_)

Fe -1.10240300 0.12905400 -0.10359600

N -0.74979400 2.07489200 -0.28288100

C 0.46044900 2.34143800 -0.83031900

C -1.37484300 4.36642700 -0.52387500

H -2.12412800 5.13854600 -0.38833800

C -1.65152400 3.05782800 -0.13794700

H -2.60346500 2.77198200 0.29081800

C 0.80040800 3.62194000 -1.24981900

H 1.78063500 3.79845900 -1.68056000

C -0.13291100 4.65118700 -1.09361400

H 0.10593400 5.65953800 -1.41703900

N 0.23700800 0.00283400 1.65429600

N 0.57978900 -0.05155700 -1.17622600

C 0.28376500 -0.02443800 -2.64964500

H -0.23067400 0.90240500 -2.89952800

H 1.22232600 -0.09552700 -3.20890300

C 1.41354800 1.16683800 -0.85800600

H 2.15553500 1.29604600 -1.65038500

O 3.94180300 2.25166700 -0.61329300

H 4.67387000 2.88129700 -0.52024900

C 2.18760900 1.05754000 0.48048100

C 1.24496100 1.10721800 1.68665600

H 1.84268000 1.00872200 2.60089200

H 0.70671000 2.05414400 1.74050200

C 3.17727300 2.23411700 0.58380000

H 2.62830500 3.17678600 0.72463700

H 3.80181100 2.07335200 1.47061300

C 2.95676500 -0.28644100 0.49454100

H 3.58905400 -0.35880700 -0.39627900

O 3.73138900 -0.37292700 1.68085400

C -0.56304000 0.09349700 2.91098200

H -1.10442100 1.03779100 2.92962700

N -1.16188700 -1.86277100 -0.31533100

C -0.00799000 -2.37001200 -0.81899300

C -2.18868300 -4.01775200 -0.50971200

H -3.06991400 -4.63480000 -0.37229900

C -2.22970500 -2.66982100 -0.16541500

H -3.12894600 -2.21069200 0.22424900

C 0.10018700 -3.70493200 -1.19283700

H 1.04482400 -4.06846500 -1.58423900

C -1.00724700 -4.54269200 -1.03635500

H -0.94849700 -5.58784700 -1.32395000

H -0.35129700 -0.87172900 -2.90725000

C 1.15638700 -1.40417700 -0.84882800

H 1.86099400 -1.68390700 -1.63627100

O 3.34256600 -3.02953000 -0.61029900

H 3.98858900 -3.74286400 -0.48956800

C 1.93117600 -1.44419800 0.49412800

C 0.99120000 -1.28801900 1.69467600

H 1.58945200 -1.31681700 2.61366200

H 0.26582900 -2.10128700 1.74632000

C 2.65813100 -2.79941300 0.61387900

H 1.93095700 -3.59897700 0.81976000

H 3.34466500 -2.73742700 1.46504300

H 4.67292700 -0.33005000 1.45846900

H -1.28072800 -0.72492600 2.94503500

H 0.10706600 0.03257500 3.77624200

O -2.18293700 0.32634300 -1.43922500

O -2.51472000 0.29685500 1.11046700

C -3.76016300 0.28229100 0.72616800

O -4.11260800 0.11033900 -0.45123700

C -4.79636200 0.52877200 1.80227800

H -4.41325800 0.18955900 2.76691300

H -4.98532500 1.60758100 1.85315700

H -5.73339200 0.02904500 1.55130900

H -2.55176700 -0.49806600 -1.81938800

C -4.50382680 1.35425538 -4.39550692

H -3.58039807 1.91077883 -4.31254024

H -4.51190471 0.38741068 -4.88647179

C -5.69927930 1.84276510 -3.70280695

O -5.94387950 3.04403975 -3.55901922

O -6.49521772 0.86581982 -3.28233988

H -7.25670059 1.24811664 -2.80121433

[(L^1^)Fe^V^=O]^2+^ (^4^INT_IS_)

Fe -1.07290000 0.07517500 -0.03209200

N -0.82708000 2.02341600 -0.30839900

C 0.37802700 2.35571100 -0.83059500

C -1.59457600 4.26073900 -0.61185000

H -2.39696900 4.98393600 -0.51679800

C -1.80179100 2.94411100 -0.21509100

H -2.74935900 2.59976700 0.17604200

C 0.64869400 3.64920000 -1.25776100

H 1.62598800 3.87982000 -1.66894700

C -0.35278200 4.61799800 -1.14202600

H -0.16893000 5.63568800 -1.47231500

N 0.25069800 -0.01286200 1.67108800

N 0.58716100 -0.01675400 -1.17002600

C 0.24938500 0.01179400 -2.62999700

H -0.33493600 0.90216900 -2.85564100

H 1.17397800 0.00842300 -3.21700900

C 1.37860500 1.22498900 -0.85025800

H 2.11906300 1.38443200 -1.63880100

O 3.84184900 2.43750900 -0.58553400

H 4.54519600 3.09767500 -0.48279800

C 2.14833900 1.14549200 0.49340600

C 1.20212200 1.14213900 1.69973100

H 1.80445800 1.07285700 2.61350300

H 0.61500500 2.05977600 1.75602400

C 3.07788600 2.36938800 0.60979900

H 2.48160800 3.28187300 0.75847800

H 3.70855500 2.23196700 1.49624000

C 2.98125500 -0.15932600 0.49928100

H 3.62016900 -0.19263400 -0.38909000

O 3.75318000 -0.22113900 1.68875900

C -0.54489800 0.02501300 2.93413300

H -1.12632400 0.94458300 2.96963200

N -1.05931700 -1.89360100 -0.34293700

C 0.11501900 -2.35549300 -0.84164400

C -2.02648700 -4.06627900 -0.58684600

H -2.89186000 -4.71017700 -0.47431600

C -2.11276000 -2.72676500 -0.22455600

H -3.03107500 -2.29229400 0.14828400

C 0.26498000 -3.68057700 -1.23379500

H 1.22241700 -4.01002400 -1.62412600

C -0.82091600 -4.55002100 -1.10179300

H -0.72970500 -5.58887800 -1.40364500

H -0.33935000 -0.86836800 -2.88610500

C 1.23329900 -1.33951700 -0.86232500

H 1.94839400 -1.57714400 -1.65445500

O 3.48711800 -2.87072600 -0.64534500

H 4.16569900 -3.55492100 -0.53471800

C 2.01010300 -1.36259800 0.48139400

C 1.06764800 -1.26648200 1.68735100

H 1.67077300 -1.27789800 2.60328800

H 0.38216000 -2.11429600 1.73244100

C 2.79737200 -2.68553200 0.58303300

H 2.10706900 -3.51914900 0.78147900

H 3.48289800 -2.60338500 1.43343900

H 4.69329600 -0.13743100 1.47198100

H -1.22966900 -0.82164500 2.95718100

H 0.13182400 -0.02208000 3.79479600

O -2.29470600 0.25748700 -1.45781500

O -2.55626100 0.20633200 1.14763900

C -3.79324500 0.14613100 0.77791000

O -4.17148300 -0.01480800 -0.40293600

C -4.83172400 0.27273700 1.86945300

H -4.63263300 -0.47358900 2.64459800

H -4.73604700 1.26306200 2.32817800

H -5.83787800 0.14709500 1.46889200

H -2.57755300 -0.57951900 -1.86927900

C -4.98990818 0.99674881 -4.14650182

H -4.50270920 0.23006488 -4.73314936

H -4.65797738 2.02664592 -4.21745476

C -6.19517150 0.65823733 -3.38470658

O -6.36461414 -0.44523719 -2.85821048

O -7.05722970 1.66510043 -3.29630431

H -7.85282729 1.37293070 -2.80727452

[(L^1^)Fe^V^=O]^2+^ (^6^INT_HS_)

Fe -1.22091200 0.14577700 -0.03032100

N -0.68945200 2.15628700 -0.42392600

C 0.56139800 2.36504500 -0.89097000

C -1.25078700 4.43921200 -0.84106200

H -1.99160700 5.23073500 -0.81539600

C -1.58421200 3.16127200 -0.40705100

H -2.57526200 2.91148900 -0.04624700

C 0.96339300 3.61655200 -1.34394900

H 1.97874400 3.75108300 -1.70209000

C 0.04348800 4.66864300 -1.31571400

H 0.33099600 5.65410100 -1.66923800

N 0.23907800 -0.04296900 1.74256100

N 0.64076900 -0.03992500 -1.16705100

C 0.28761000 0.00192400 -2.61824100

H -0.26265100 0.91661300 -2.83743700

H 1.19712700 -0.03882800 -3.22921200

C 1.47490000 1.15955600 -0.83190300

H 2.26458300 1.26276300 -1.58305100

O 3.95400400 2.30555200 -0.46732600

H 4.68316600 2.92994400 -0.32794200

C 2.18154300 1.06146500 0.55041400

C 1.20769200 1.09461000 1.73993300

H 1.79995200 1.04909700 2.66316800

H 0.63150200 2.02175600 1.75839400

C 3.14788000 2.25401100 0.70175200

H 2.57815300 3.18544100 0.83671700

H 3.74615000 2.09133500 1.60590200

C 2.97232900 -0.26747100 0.58333200

H 3.63920300 -0.32013500 -0.28310500

O 3.70476300 -0.35420600 1.79708300

C -0.54919100 0.01218900 3.00470700

H -1.09551100 0.95374000 3.05171300

N -1.08341700 -1.95748300 -0.42554100

C 0.10827900 -2.39215100 -0.89214200

C -2.03860400 -4.11803400 -0.79933500

H -2.90607100 -4.76742200 -0.75392200

C -2.13906700 -2.79433000 -0.38754400

H -3.07424800 -2.37095800 -0.03556900

C 0.27941400 -3.70283800 -1.32893900

H 1.25359900 -4.01744900 -1.68891300

C -0.80805900 -4.57794500 -1.27812000

H -0.69980300 -5.60397700 -1.61621700

H -0.34716300 -0.84951600 -2.86335600

C 1.23301000 -1.37842900 -0.84576800

H 1.97808500 -1.62563800 -1.60861900

O 3.44508100 -2.97371600 -0.55732200

H 4.09429900 -3.68225900 -0.42611000

C 1.96246800 -1.43593800 0.52810100

C 1.00382000 -1.32454200 1.72354100

H 1.59778200 -1.39197700 2.64430000

H 0.28184000 -2.14351300 1.73394400

C 2.70732100 -2.78289600 0.64246500

H 1.98645100 -3.59906700 0.80027400

H 3.35924000 -2.73187000 1.52138900

H 4.65357800 -0.32687400 1.60603000

H -1.26967600 -0.80672700 3.01575400

H 0.11723000 -0.07306200 3.87176200

O -2.57129300 0.27804800 -1.40956000

O -2.71760000 0.42281600 1.26285800

C -3.94290100 0.29858100 0.91660500

O -4.32530700 0.03893000 -0.26096700

C -5.01716000 0.46410100 1.96394400

H -4.79478100 -0.20037200 2.80451200

H -4.97770700 1.49663000 2.32917900

H -6.00810600 0.25400500 1.56037900

H -2.91880600 -0.43555600 -1.97340400

C -5.11381915 1.21415046 -4.27825304

H -4.69935103 0.69571601 -5.13192998

H -4.84590491 2.24776385 -4.08913470

C -6.15513121 0.56137206 -3.48004803

O -6.18218763 -0.65724888 -3.28563630

O -7.03433676 1.41609188 -2.96898617

H -7.72094679 0.92359134 -2.47553030

[(L^2^)Fe^V^=O]^2+^ (^2^INT_LS_)

Fe 1.48418900 -0.11070600 -0.11638200

N 1.19535100 -2.06956500 -0.27930300

C 0.01849300 -2.38287900 -0.87181000

C 1.86006500 -4.36120200 -0.37607500

H 2.61019000 -5.11454200 -0.16234500

C 2.10049700 -3.03065700 -0.04314600

H 3.02429300 -2.71226300 0.42213800

C -0.28389800 -3.68594300 -1.24363000

H -1.23517000 -3.90785900 -1.71559100

C 0.65231200 -4.69303000 -0.98969400

H 0.43971600 -5.71998600 -1.26932100

N 0.08995300 -0.00226700 1.56842800

N -0.15832000 0.00737900 -1.27798300

C 0.20105500 -0.03588800 -2.73559100

H 0.75712500 -0.94894400 -2.94342600

H -0.71275100 -0.00820200 -3.33879300

C -0.96351200 -1.23352000 -0.97059200

H -1.67317300 -1.39968000 -1.78698500

C -1.77635200 -1.12267300 0.34485000

C -0.88072700 -1.13881200 1.58706400

H -1.51889400 -1.05723100 2.47257600

H -0.33238300 -2.07419400 1.67038200

C -2.59839900 0.17684700 0.30189700

H -3.18744200 0.20169400 -0.62206400

O -3.42883900 0.18072500 1.44038100

C 0.84601800 -0.04612600 2.85630500

H 1.41204100 -0.97422500 2.91173700

N 1.49416800 1.87479700 -0.34190900

C 0.36527600 2.34776600 -0.92840400

C 2.48766600 4.04719500 -0.50477800

H 3.34180900 4.68737100 -0.31316700

C 2.53088200 2.70694300 -0.13201500

H 3.40594800 2.27485500 0.33569000

C 0.26330800 3.66876000 -1.34461900

H -0.64830500 4.02153300 -1.81321700

C 1.33953100 4.53338500 -1.12993400

H 1.27818800 5.57064000 -1.44340300

H 0.81854100 0.82757500 -2.98209400

C -0.77901000 1.35293100 -0.99345400

H -1.45887900 1.60320500 -1.81668300

C -1.60488100 1.36266700 0.32724300

C -0.70451300 1.26207400 1.56586100

H -1.34643200 1.27736600 2.45393000

H -0.02896300 2.11230300 1.63124800

H -4.16867200 0.78853100 1.27867800

H 1.53562100 0.79551600 2.90121600

H 0.14124900 0.00800500 3.69350100

O 2.61766200 -0.28920300 -1.41963400

O 2.87438300 -0.22417200 1.13263600

C -2.71960700 -2.33945300 0.43245300

O -2.62489100 -3.19207600 1.28188000

O -3.60836200 -2.32804600 -0.56774700

C -2.33551100 2.71768700 0.40466400

O -1.80976700 3.72357600 0.82424900

O -3.58239400 2.64376300 -0.07543700

C -4.34698500 3.88703300 -0.06206700

H -5.31801200 3.62877400 -0.48050000

H -4.43966800 4.25053700 0.96304600

H -3.84169700 4.63696200 -0.67359600

C -4.59475300 -3.39918000 -0.54869400

H -5.18337800 -3.33719200 0.36856900

H -5.21630600 -3.23285800 -1.42680000

H -4.09386800 -4.36847800 -0.59604900

C 4.12253200 -0.21480100 0.76799300

O 4.49068000 -0.08386300 -0.41279600

C 5.15205600 -0.40929600 1.85976100

H 4.74337900 -0.06822100 2.81316800

H 5.37835400 -1.47995400 1.92861400

H 6.07522100 0.11832400 1.61354800

H 2.96831200 0.54202100 -1.80249000

C 5.57953752 -0.55502786 -3.62651433

H 4.86168849 -0.80413477 -4.39590431

H 6.07243473 -1.34379771 -3.06902451

C 5.99363917 0.84057276 -3.45799014

O 5.22843321 1.79130925 -3.64271951

O 7.25425010 0.96679634 -3.05841501

H 7.48395414 1.91581454 -2.99446720

[(L^2^)Fe^V^=O]^2+^ (^4^INT_IS_)

Fe 1.45349300 -0.07227800 -0.05789000

N 1.23333900 -2.02542200 -0.31491900

C 0.05456300 -2.37603400 -0.88234200

C 2.00134500 -4.27670400 -0.48584900

H 2.79269500 -4.99869000 -0.31757000

C 2.19655400 -2.94460600 -0.13603900

H 3.12454500 -2.58908500 0.28983600

C -0.20177700 -3.68381500 -1.26838100

H -1.15393400 -3.93787400 -1.72172700

C 0.78579400 -4.65182500 -1.06009600

H 0.60967700 -5.68256200 -1.35127300

N 0.07521600 0.02300900 1.58456800

N -0.16536500 -0.00179800 -1.27365000

C 0.23338400 -0.04612900 -2.71797700

H 0.83321000 -0.93491900 -2.90525900

H -0.66522600 -0.05799900 -3.34459700

C -0.95209600 -1.25072300 -0.96790800

H -1.66338700 -1.42924400 -1.78064000

C -1.75838900 -1.15767900 0.35340700

C -0.86026200 -1.14418500 1.59562500

H -1.50115700 -1.08406000 2.48078600

H -0.28113200 -2.06122000 1.67903400

C -2.61585700 0.11805700 0.30660800

H -3.21094100 0.12028900 -0.61374400

O -3.43862200 0.10796700 1.45035700

C 0.82160500 0.01163700 2.87934000

H 1.40761000 -0.90262300 2.95112200

N 1.43771000 1.88895000 -0.37447300

C 0.29602700 2.34436600 -0.94892500

C 2.42281000 4.05643100 -0.58539200

H 3.27813100 4.70266800 -0.42151400

C 2.48168200 2.72264400 -0.19814300

H 3.37052200 2.29308600 0.24448100

C 0.18190000 3.65917200 -1.37991700

H -0.73755100 4.00143400 -1.84036500

C 1.25804900 4.52990300 -1.19230000

H 1.18684800 5.56308300 -1.51719800

H 0.82774500 0.83415800 -2.95943800

C -0.82509600 1.32515200 -1.00478100

H -1.50880100 1.55140100 -1.83220200

C -1.65297100 1.32887700 0.31637500

C -0.75365300 1.26579300 1.55921900

H -1.39909300 1.27369600 2.44465500

H -0.10063300 2.13435900 1.61771800

H -4.19270100 0.69875700 1.29108600

H 1.49511900 0.86662700 2.91704600

H 0.10917700 0.06279100 3.70995300

O 2.72310500 -0.25653600 -1.44231400

O 2.89487300 -0.17913700 1.17216100

C -2.66530500 -2.40147500 0.45058700

O -2.53626100 -3.25045300 1.29933100

O -3.56287200 -2.41713200 -0.54096500

C -2.41536500 2.66754600 0.37731200

O -1.90183800 3.69609600 0.75610800

O -3.67203800 2.55243800 -0.06558000

C -4.46585300 3.77783800 -0.06418900

H -5.44102400 3.48558900 -0.44919500

H -4.53988100 4.16622500 0.95325000

H -3.99492100 4.52213500 -0.70902400

C -4.51779600 -3.51665200 -0.51237200

H -5.10012100 -3.47022600 0.40979400

H -5.15117300 -3.36941000 -1.38538200

H -3.98936400 -4.47102300 -0.56284400

C 4.14430900 -0.14218600 0.84413500

O 4.56559700 -0.02127900 -0.32711200

C 5.14307400 -0.24759500 1.97414300

H 4.92321800 0.52059500 2.72181400

H 5.02312200 -1.22465100 2.45513400

H 6.16351800 -0.14024800 1.60563400

H 3.03319500 0.58042200 -1.83366400

C 5.57953752 -0.55502786 -3.62651433

H 4.86168849 -0.80413477 -4.39590431

H 6.07243473 -1.34379771 -3.06902451

C 5.99363917 0.84057276 -3.45799014

O 5.22843321 1.79130925 -3.64271951

O 7.25425010 0.96679634 -3.05841501

H 7.48395414 1.91581454 -2.99446720

[(L^2^)Fe^V^=O]^2+^ (^6^INT_HS_)

Fe 1.51979000 -0.36173500 0.03874400

N 0.76905300 -2.38822400 -0.39110100

C -0.44133400 -2.39723700 -0.98397000

C 0.99074000 -4.73301800 -0.78289800

H 1.58511400 -5.63523000 -0.68905400

C 1.48060600 -3.52212800 -0.29939600

H 2.45518000 -3.43824900 0.16914700

C -0.99491300 -3.56285900 -1.49850300

H -1.97226900 -3.54144000 -1.96920000

C -0.26567500 -4.75167100 -1.39070400

H -0.67234600 -5.67834600 -1.78341700

N 0.12582400 -0.01734500 1.65905200

N -0.14397500 0.01547100 -1.24608700

C 0.28371900 -0.10230500 -2.67508600

H 0.74460300 -1.07465800 -2.83911700

H -0.58515000 0.01680600 -3.33260100

C -1.15824800 -1.06175200 -0.96149000

H -1.91994400 -1.03255400 -1.74826600

C -1.88865400 -0.85986200 0.39479500

C -0.98037900 -1.02423500 1.62208700

H -1.59966400 -0.89250800 2.51514900

H -0.54599600 -2.02014500 1.67046000

C -2.51791400 0.54333400 0.39348700

H -3.13966300 0.65787800 -0.50090600

O -3.28621000 0.66623100 1.56930200

C 0.84041800 -0.15500900 2.96271600

H 1.25979400 -1.15641200 3.04161600

N 1.71513300 1.82035200 -0.43181100

C 0.62077000 2.32752800 -1.03488800

C 2.91115400 3.84245300 -0.88043800

H 3.83140000 4.41074200 -0.79999300

C 2.84015700 2.55369300 -0.35876900

H 3.69333000 2.07795700 0.11456100

C 0.62126100 3.59920900 -1.59596500

H -0.26940600 3.98394200 -2.08120700

C 1.78344300 4.37036800 -1.51215400

H 1.80810300 5.36823700 -1.93855800

H 1.01199700 0.67484300 -2.90141600

C -0.60182100 1.42685000 -0.99002200

H -1.30100100 1.70848100 -1.78829500

C -1.36056800 1.56717500 0.36574200

C -0.45888300 1.35652100 1.59301200

H -1.07890700 1.50004200 2.48514500

H 0.34718800 2.08597700 1.62450500

H -3.94736200 1.36443100 1.43374200

H 1.64816200 0.57447700 3.00999100

H 0.13422600 0.01837000 3.78190200

O 2.75242900 -0.67503800 -1.34050800

O 2.89090800 -0.74582700 1.28078900

C -2.99319300 -1.93124600 0.50757700

O -3.00325900 -2.78663900 1.35930900

O -3.89392200 -1.79278500 -0.47195000

C -1.88068100 3.01638900 0.45255900

O -1.20781300 3.93053900 0.87057100

O -3.12923600 3.12759100 -0.01544400

C -3.70049500 4.47057900 0.00448800

H -4.70125300 4.36081900 -0.40916900

H -3.73291300 4.84112100 1.03078600

H -3.09199100 5.13883300 -0.60775000

C -5.01872200 -2.71729200 -0.43032400

H -5.57085300 -2.57947400 0.50121300

H -5.63215500 -2.46171900 -1.29256200

H -4.65728600 -3.74598700 -0.49169800

C 4.15657000 -0.78427700 0.96727500

O 4.59667000 -0.52822600 -0.16924800

C 5.10728100 -1.14811700 2.08005100

H 4.93624200 -0.48411700 2.93282600

H 4.88679500 -2.17212300 2.40218100

H 6.14224000 -1.08098000 1.74342500

H 3.15261600 0.09899100 -1.77882000

C 5.57953752 -0.55502786 -3.62651433

H 4.86168849 -0.80413477 -4.39590431

H 6.07243473 -1.34379771 -3.06902451

C 5.99363917 0.84057276 -3.45799014

O 5.22843321 1.79130925 -3.64271951

O 7.25425010 0.96679634 -3.05841501

H 7.48395414 1.91581454 -2.99446720

[(L^1^)Fe^V^=O]^2+^ (^2^TS2_LS_)

Fe -0.56213700 0.29574000 0.28207700

N 0.02166000 2.15468500 -0.13729600

C 1.13030800 2.22609600 -0.90878300

C -0.33092100 4.49789100 -0.38226700

H -0.92783400 5.37349900 -0.15174500

C -0.70390500 3.25498500 0.11778100

H -1.58180300 3.10861900 0.73320200

C 1.55404400 3.43466700 -1.44999300

H 2.45232300 3.45736500 -2.05822100

C 0.81184200 4.58774500 -1.18144800

H 1.11972500 5.54370000 -1.59372500

N 1.10451600 -0.02000400 1.72760800

N 0.83422900 -0.15728700 -1.14413100

C 0.25947100 -0.12359000 -2.52798600

H -0.15307700 0.86281700 -2.72829000

H 1.05064200 -0.35653700 -3.24884400

C 1.88012600 0.92330400 -1.05279700

H 2.46371200 0.92287600 -1.97720500

O 4.50406000 1.68363700 -1.36456900

H 5.33403800 2.18025800 -1.43735100

C 2.88268900 0.73611600 0.11623500

C 2.22422100 0.93943000 1.48632800

H 2.98630500 0.79072600 2.26149300

H 1.82952100 1.95042100 1.59803200

C 4.01954400 1.76698900 -0.03126800

H 3.64394200 2.77595100 0.19538400

H 4.79375700 1.52154700 0.70472300

C 3.45165200 -0.70017600 0.03619500

H 3.87337000 -0.87362200 -0.95904800

O 4.43660900 -0.86638200 1.04565600

C 0.62044700 0.19073300 3.12296400

H 0.24758800 1.20711500 3.23048200

N -0.93397400 -1.65647000 0.13313300

C 0.01730900 -2.34579700 -0.55031800

C -2.26749400 -3.63242200 0.27637700

H -3.18258000 -4.10338600 0.61748600

C -2.06146500 -2.27936300 0.52945600

H -2.80112800 -1.65684500 1.01450200

C -0.14430500 -3.68976400 -0.85771800

H 0.63732000 -4.20172500 -1.40945400

C -1.30337600 -4.34646000 -0.43301200

H -1.45093800 -5.39740200 -0.66174600

H -0.53867300 -0.85981500 -2.61156100

C 1.27317700 -1.56915600 -0.86396700

H 1.75057400 -1.97019000 -1.76203100

O 3.24865200 -3.47282800 -1.00600000

H 3.77576800 -4.28682900 -0.98863100

C 2.30011400 -1.70054100 0.29152200

C 1.66644000 -1.40235700 1.65407500

H 2.43692000 -1.50947500 2.42766100

H 0.86397800 -2.10462700 1.88622600

C 2.84790300 -3.14182500 0.31665400

H 2.07440300 -3.83197300 0.68438600

H 3.68748500 -3.16867300 1.02031700

H 5.29192200 -1.06215400 0.63638500

H -0.19192400 -0.50384500 3.33347500

H 1.44528000 0.01919000 3.82461200

O -1.82724700 0.59887900 -0.94667500

O -1.56166000 0.80512300 1.72674700

C -2.88887600 0.85308600 1.87849200

O -3.67751800 0.24442000 1.17295500

C -3.30133500 1.71774800 3.04884500

H -2.90563800 2.73262800 2.93306300

H -4.38879100 1.74557000 3.12611200

H -2.87706900 1.31101000 3.97326400

H -2.37692100 -0.20108500 -1.15937900

C -4.27573700 1.26698000 -1.47087700

H -3.58469200 1.62141000 -2.22231400

H -4.80196800 1.96588900 -0.83263900

C -4.62743500 -0.15773100 -1.43007800

O -3.81556500 -1.05441700 -1.66702400

O -5.89500600 -0.36925600 -1.09883200

H -6.07022100 -1.33101700 -1.09238400

[(L^1^)Fe^V^=O]^2+^ (^4^TS2_IS_)

Fe 0.59982900 -0.24410200 0.32305300

N 0.10592700 -2.12854400 -0.09416200

C -1.00495600 -2.27373100 -0.85161800

C 0.58031100 -4.45313700 -0.29026400

H 1.22711100 -5.28982400 -0.05003500

C 0.89546300 -3.18074400 0.17423700

H 1.77888500 -2.97260700 0.76343500

C -1.37075900 -3.51550300 -1.35869600

H -2.27033900 -3.59756900 -1.95985500

C -0.56728300 -4.62172500 -1.06951600

H -0.82981800 -5.60227100 -1.45451300

N -1.18956000 0.03484700 1.76265800

N -0.80291900 0.11373900 -1.12522300

C -0.17960400 0.07392800 -2.48903600

H 0.27620000 -0.90004800 -2.65139100

H -0.95655800 0.26120300 -3.23796500

C -1.80715500 -1.00955200 -1.03489700

H -2.36188600 -1.04767300 -1.97639800

O -4.36376500 -1.90913000 -1.42415200

H -5.17565700 -2.43218500 -1.51409400

C -2.85553600 -0.84012300 0.09714300

C -2.24204300 -0.98426600 1.49618500

H -3.04420900 -0.87329800 2.23777600

H -1.79567600 -1.97103800 1.63558400

C -3.93968900 -1.92461700 -0.06748700

H -3.53276300 -2.90772600 0.21223800

H -4.75758300 -1.68854700 0.62249100

C -3.48352400 0.56697500 -0.03876700

H -3.87644800 0.69794000 -1.05239800

O -4.51111900 0.70975600 0.93046600

C -0.73122300 -0.12669100 3.16819500

H -0.30832200 -1.12119200 3.30209400

N 0.87784800 1.72046500 0.12149600

C -0.10551100 2.35381800 -0.57140800

C 2.13153300 3.74984000 0.21579900

H 3.03287200 4.26136800 0.53393900

C 1.98353700 2.39521000 0.49736600

H 2.75592700 1.81525800 0.98527200

C -0.00180500 3.69785500 -0.90216800

H -0.80476500 4.16496100 -1.46299000

C 1.13180800 4.40955500 -0.49712200

H 1.23516900 5.46060200 -0.74851300

H 0.59490200 0.83553300 -2.55858800

C -1.31606900 1.51164200 -0.88698500

H -1.78743400 1.86320600 -1.80894900

O -3.35799500 3.32288700 -1.14531600

H -3.91875800 4.11401800 -1.16851100

C -2.38615100 1.62319000 0.23269000

C -1.79414500 1.38838700 1.62717400

H -2.59659000 1.49866700 2.36842500

H -1.02342300 2.12600000 1.86113600

C -2.99599000 3.03939000 0.19963900

H -2.26846200 3.77105300 0.58052900

H -3.86317000 3.04552100 0.86949400

H -5.35341600 0.88990000 0.48832800

H 0.04021100 0.61195800 3.38554900

H -1.57684700 0.01154800 3.85362800

O 1.97446300 -0.54702100 -0.83240500

O 1.54713500 -0.62921300 1.80573600

C 2.88247100 -0.64404000 2.03530500

O 3.68925900 -0.05315900 1.34820600

C 3.22206600 -1.44829400 3.26822500

H 2.76304900 -0.98339300 4.14773000

H 2.81544600 -2.46184700 3.18814800

H 4.30459900 -1.48461500 3.39592600

H 2.49718200 0.28368900 -0.90763300

C 3.98256100 -1.01987900 -2.16163900

H 3.27656500 -0.87198500 -2.96691800

H 4.33773100 -2.01615700 -1.92793000

C 4.60658100 0.15641800 -1.54964200

O 4.01580500 1.23086200 -1.43621000

O 5.83765800 -0.07563900 -1.09980600

H 6.17080700 0.73448100 -0.66620600

[(L^1^)Fe^V^=O]^2+^ (^6^TS2_HS_)

Fe -0.64503400 0.35377800 0.41314200

N 0.16476800 2.27336500 -0.12430100

C 1.23529400 2.24353300 -0.94141900

C -0.06913900 4.62116700 -0.47569600

H -0.60666800 5.54048700 -0.27029700

C -0.48447100 3.42654500 0.10424700

H -1.34569800 3.36340900 0.76044700

C 1.70687400 3.39655900 -1.56097800

H 2.58259700 3.33950600 -2.19906900

C 1.04257000 4.60291400 -1.32261100

H 1.38698700 5.51779300 -1.79499200

N 1.13493900 -0.03612300 1.78282900

N 0.82975600 -0.14710000 -1.12499600

C 0.17478600 -0.06192000 -2.46435400

H -0.21495500 0.94323000 -2.61408800

H 0.90478600 -0.30178700 -3.24650400

C 1.90895300 0.89166600 -1.05211900

H 2.49636000 0.84800500 -1.97454600

O 4.55251000 1.58800100 -1.35454300

H 5.39867300 2.05702000 -1.42311500

C 2.90365200 0.68492500 0.12385200

C 2.26149600 0.90435400 1.50188600

H 3.03486100 0.74461300 2.26409300

H 1.88301700 1.92053500 1.61694300

C 4.06575500 1.68885300 -0.02322500

H 3.71344800 2.70756500 0.19704500

H 4.83250400 1.42729100 0.71488100

C 3.44172200 -0.76116200 0.04924200

H 3.87294900 -0.94074600 -0.94025900

O 4.41197200 -0.94657700 1.07059400

C 0.68539500 0.17539100 3.18696900

H 0.32149500 1.19523600 3.30235000

N -0.94087600 -1.77599100 0.11043000

C 0.00641000 -2.38338900 -0.63629400

C -2.25997200 -3.76524000 0.08323000

H -3.16957000 -4.27507900 0.38040900

C -2.05814900 -2.44007500 0.45957300

H -2.79712100 -1.87034600 1.01176300

C -0.13814200 -3.69867500 -1.06114200

H 0.64859700 -4.15630300 -1.65195000

C -1.28994600 -4.40076300 -0.69187000

H -1.42822900 -5.42859300 -1.01319300

H -0.65682700 -0.76348400 -2.50873300

C 1.25533600 -1.56443100 -0.88901300

H 1.75649700 -1.93140300 -1.79034400

O 3.21988500 -3.49867600 -1.01232500

H 3.70817800 -4.33658500 -0.99534800

C 2.26172100 -1.73208200 0.28307100

C 1.63570200 -1.43782100 1.65398100

H 2.40645700 -1.59398000 2.41960600

H 0.80497700 -2.11008500 1.87249300

C 2.77708500 -3.18577600 0.30120000

H 1.97688200 -3.86623800 0.62698100

H 3.59093000 -3.24525900 1.03320800

H 5.24742100 -1.23549700 0.67580000

H -0.12678300 -0.51381200 3.41515900

H 1.52481900 -0.00053400 3.87038600

O -1.96032900 0.69886300 -0.73918500

O -1.63691400 0.76689300 1.90764900

C -2.97861100 0.80568800 2.06561200

O -3.73489300 0.12186200 1.40395300

C -3.40296400 1.74713000 3.16651000

H -3.00407400 1.39300600 4.12347100

H -2.98980200 2.74608800 2.99202600

H -4.49151400 1.79144900 3.21713800

H -2.55637400 -0.09100400 -0.87205800

C -4.23058100 1.25179600 -2.04901200

H -3.39353300 1.27525100 -2.73324100

H -4.77154600 2.16127300 -1.81396100

C -4.68888300 -0.04006800 -1.53569500

O -3.93371500 -1.00784800 -1.40953800

O -5.97501000 -0.04726700 -1.20581500

H -6.20837000 -0.92503700 -0.84392200

[(L^2^)Fe^V^=O]^2+^ (^2^TS2_LS_)

Fe 1.03320000 -0.14296200 -0.06427800

N 0.67625800 -2.10660100 -0.29593500

C -0.49664900 -2.37981200 -0.91296800

C 1.30618900 -4.39939800 -0.43610200

H 2.03677800 -5.17282600 -0.22581900

C 1.56561800 -3.08397100 -0.06283800

H 2.47270700 -2.77232700 0.43808200

C -0.81498400 -3.66845500 -1.32007200

H -1.76416800 -3.86235800 -1.80814100

C 0.10202800 -4.69550400 -1.07716900

H -0.12337200 -5.71215600 -1.38368500

N -0.41389000 0.04082800 1.59151700

N -0.60860900 0.01215700 -1.29000300

C -0.22701500 -0.03167000 -2.73986200

H 0.30836900 -0.95520100 -2.94843200

H -1.13661900 0.02399000 -3.34798900

C -1.44480400 -1.20765500 -0.99352200

H -2.16116200 -1.34641800 -1.80928500

O -4.08290200 -2.30478200 -0.59117700

C -2.25644200 -1.09278300 0.32483500

C -1.37420700 -1.10515100 1.58106900

H -2.02750600 -1.03195000 2.45660200

H -0.81676700 -2.03468000 1.67076400

C -3.07620300 0.20690000 0.26210300

H -3.65350100 0.22423400 -0.66902700

O -3.92175000 0.22405900 1.38957700

C 0.31346400 0.02648500 2.89619700

H 0.83477400 -0.92087800 3.00904500

N 1.03439700 1.85832900 -0.25785000

C -0.06438900 2.32909800 -0.91198300

C 2.04505300 4.01878400 -0.42170600

H 2.89787600 4.65108400 -0.20127700

C 2.07016300 2.68518300 -0.01626000

H 2.91013500 2.24481000 0.50615300

C -0.13343100 3.63791100 -1.35935300

H -1.02055300 3.98384200 -1.87780300

C 0.93903200 4.50156300 -1.11291400

H 0.90158100 5.53096600 -1.45500400

H 0.42645900 0.80762100 -2.96879600

C -1.21812700 1.35911900 -1.01021300

H -1.87250900 1.62295200 -1.84955000

O -4.03249300 2.67021200 -0.20216100

C -2.07824800 1.38708900 0.28838700

C -1.21236500 1.30268400 1.54977100

H -1.87859500 1.31827100 2.41951900

H -0.54271600 2.15576200 1.62979200

H -4.65871200 0.83102700 1.21183400

H 1.03238800 0.84599600 2.91618300

H -0.40737500 0.15150400 3.71187600

O 2.12944500 -0.22340100 -1.42770200

O 2.33127900 -0.67295500 1.18917800

C 3.34939100 -0.12789800 1.84351100

O 3.73663900 1.02538000 1.72118700

C 4.04427600 -1.11971500 2.75673400

H 4.44777600 -0.59075300 3.62296400

H 3.37986900 -1.92525900 3.07608000

H 4.88722900 -1.55425400 2.20605500

H 2.81557700 0.50512000 -1.45884900

C 4.52541400 -1.21515900 -1.57573500

H 4.14038100 -1.20747400 -2.58629400

H 4.74385200 -2.15689100 -1.08587500

C 4.91347200 0.04850700 -0.95419000

O 4.39825600 1.12461900 -1.27227400

O 5.80741200 -0.08756700 0.02099900

H 5.90519200 0.77309700 0.47854900

C -5.07130900 -3.37420500 -0.56943200

H -5.66486700 -3.30397200 0.34402300

H -5.68767000 -3.21333100 -1.45218000

H -4.57176700 -4.34465500 -0.60642200

C -4.79286600 3.91568800 -0.22940600

H -5.74603400 3.65771000 -0.68727600

H -4.92801500 4.28648200 0.78838000

H -4.25928700 4.66008500 -0.82341400

C -3.20020000 -2.30944300 0.41396800

O -3.10967400 -3.15704900 1.26910100

C -2.80658800 2.74531400 0.33043100

O -2.29842100 3.75071100 0.77035500

[(L^2^)Fe^V^=O]^2+^ (^4^TS2_IS_)

Fe 1.00490200 -0.27532800 0.30575500

N 0.54494700 -2.17892000 -0.05339300

C -0.57324900 -2.36814800 -0.78944100

C 1.02610600 -4.50915800 -0.13281000

H 1.67747000 -5.33006500 0.14620900

C 1.34175200 -3.21236900 0.25947100

H 2.22895500 -2.97121800 0.82987400

C -0.93850400 -3.63484300 -1.22687200

H -1.83936900 -3.76423600 -1.81696600

C -0.12925900 -4.72279400 -0.88730800

H -0.39602600 -5.72327700 -1.21304500

N -0.74936700 -0.00871800 1.76752100

N -0.43574700 0.02591500 -1.13557100

C 0.16460800 -0.02886700 -2.50867200

H 0.63893800 -0.99632900 -2.65621500

H -0.62730900 0.12461700 -3.25021800

C -1.40480100 -1.12399900 -0.99928500

H -1.98034300 -1.20004100 -1.92748500

O -4.16103900 -1.96676200 -1.09872200

C -2.42037200 -0.95084500 0.16190700

C -1.77903700 -1.06164900 1.55087200

H -2.57294700 -0.96337100 2.29952900

H -1.32051600 -2.03868100 1.69605100

C -3.09875200 0.41953000 -0.00065200

H -3.50076600 0.50542500 -1.01662800

O -4.12400300 0.49473700 0.96320200

C -0.25998900 -0.12559900 3.16857000

H 0.18785700 -1.10676200 3.31679100

N 1.23475200 1.68567200 0.06968200

C 0.23628700 2.29116500 -0.62729000

C 2.45242100 3.73812000 0.13173900

H 3.34352200 4.27109600 0.44330700

C 2.32847500 2.38541100 0.43228400

H 3.11376400 1.82713100 0.92373400

C 0.32180300 3.62831300 -0.98333500

H -0.48086900 4.09082800 -1.54551500

C 1.44206200 4.36716900 -0.59140400

H 1.52273000 5.41627300 -0.85759800

H 0.92226500 0.74649600 -2.60610500

C -0.96860200 1.42109400 -0.91113500

H -1.46206500 1.74735000 -1.83481600

O -3.75396600 2.96226700 -0.53414300

C -2.02245600 1.50447200 0.23488200

C -1.39250000 1.32371600 1.62232800

H -2.18806700 1.42379200 2.37079900

H -0.65531700 2.10107700 1.81872900

H -4.76153500 1.17051200 0.68071100

H 0.49491100 0.63828400 3.35394900

H -1.09744900 0.00690900 3.86440400

O 2.35649600 -0.56958600 -0.87915300

O 1.99484700 -0.61316500 1.77129600

C 3.33582400 -0.59298800 1.96890900

O 4.11670200 -0.02882400 1.23152400

C 3.71478400 -1.32255200 3.23648200

H 3.31824500 -2.34305300 3.22475200

H 4.80057400 -1.33967700 3.33804600

H 3.27219800 -0.81262300 4.09929100

H 2.86123300 0.27046600 -0.97661800

C 4.34319100 -1.01848500 -2.23316500

H 3.61884500 -0.92042800 -3.02974000

H 4.73187500 -1.99504400 -1.97124200

C 4.94803000 0.19742200 -1.67951900

O 4.32155800 1.25192900 -1.57033400

O 6.20436900 0.02353000 -1.27867800

H 6.52742000 0.85728900 -0.88369600

C -3.47131600 -2.07416300 0.04372500

O -3.60956900 -2.94420400 0.86909500

C -2.63117100 2.92045600 0.19453200

O -2.12075800 3.87254700 0.73880500

C -5.23180600 -2.93580200 -1.28362200

H -5.66748400 -2.70099700 -2.25323900

H -4.82557100 -3.94947100 -1.26720700

H -5.96668000 -2.82494100 -0.48401200

C -4.39305300 4.26919100 -0.64619800

H -5.27783200 4.10241300 -1.25784400

H -4.65955800 4.63497200 0.34718600

H -3.71057400 4.97363500 -1.12558200

[(L^2^)Fe^V^=O]^2+^ (^6^TS2_HS_)

Fe 1.05129000 -0.40406500 0.38409100

N 0.27583500 -2.34354700 -0.10001100

C -0.80937000 -2.36870000 -0.89705500

C 0.52480400 -4.70515800 -0.32417700

H 1.07203000 -5.60749100 -0.07422900

C 0.94010700 -3.47607200 0.17972300

H 1.81044900 -3.36903200 0.81770300

C -1.27695000 -3.55657000 -1.44672400

H -2.15534100 -3.55186100 -2.08359300

C -0.59926200 -4.74334800 -1.15218900

H -0.94479600 -5.68559300 -1.56591500

N -0.70596000 -0.03942700 1.77138100

N -0.47957800 0.03368100 -1.15774200

C 0.15637100 -0.06257600 -2.50461700

H 0.57379800 -1.05924800 -2.63559600

H -0.58986100 0.13721300 -3.28337500

C -1.52053800 -1.03717800 -1.03806200

H -2.13448000 -1.03682200 -1.94600800

O -4.33019700 -1.70587000 -1.04125900

C -2.48090900 -0.83037200 0.16766900

C -1.80906900 -1.01721400 1.53630500

H -2.57662600 -0.87276500 2.30451100

H -1.41756400 -2.02444200 1.65744400

C -3.07433300 0.58355100 0.06488500

H -3.51472800 0.71769400 -0.92889800

O -4.04861400 0.71210900 1.07678800

C -0.22398300 -0.20823300 3.17186000

H 0.16777900 -1.21573100 3.30118800

N 1.26798500 1.71610400 0.04773200

C 0.30994000 2.29288600 -0.70947200

C 2.54045000 3.73451500 0.00494200

H 3.43352200 4.27049900 0.30595100

C 2.36721600 2.40926100 0.39490100

H 3.11744700 1.86285800 0.95532700

C 0.43442300 3.59998000 -1.15832300

H -0.34350700 4.04183100 -1.77155100

C 1.56564400 4.33465600 -0.79001500

H 1.68141800 5.36056800 -1.12514800

H 0.96739200 0.66057900 -2.57648400

C -0.92831800 1.44539800 -0.93443100

H -1.45677700 1.79231200 -1.83252000

O -3.53409500 3.18147200 -0.45889300

C -1.91467600 1.58401400 0.26596100

C -1.24947800 1.34206200 1.63045700

H -2.01592200 1.48800300 2.40100200

H -0.45261700 2.05873400 1.81565700

H -4.66226900 1.42095700 0.82444800

H 0.57119400 0.50945500 3.36981000

H -1.05678600 -0.04121600 3.86503100

O 2.34960900 -0.72641900 -0.79274200

O 2.08471200 -0.78169600 1.86094800

C 3.42943900 -0.78120300 1.99593800

O 4.15472600 -0.09224500 1.30521700

C 3.89819300 -1.68404200 3.11082300

H 3.51367100 -1.31155400 4.06675500

H 3.50199500 -2.69554700 2.97363700

H 4.98830400 -1.70438000 3.13760000

H 2.91671000 0.07983400 -0.95753000

C 4.65633100 -1.22498900 -2.07416000

H 3.82062000 -1.32076500 -2.75383700

H 5.24322000 -2.09470100 -1.80169400

C 5.04822600 0.10928400 -1.61659800

O 4.24168000 1.03850900 -1.51778600

O 6.33528300 0.20022200 -1.30660800

H 6.52574100 1.10277700 -0.98248100

C -3.60542900 -1.88122700 0.07005400

O -3.77029900 -2.75653400 0.88559900

C -2.42596900 3.03777000 0.28006200

O -1.86320100 3.93085600 0.87006600

C -5.46179300 -2.60641900 -1.20510000

H -5.92125300 -2.32141900 -2.15001100

H -5.11446800 -3.64158400 -1.23014800

H -6.15701800 -2.47561400 -0.37364400

C -4.08120000 4.53139500 -0.52862200

H -4.96390000 4.44960600 -1.16030800

H -4.34090300 4.87638900 0.47404400

H -3.34405600 5.20640400 -0.96788000

[(L^1^)Fe^V^=O]^2+^ (^2^P_LS_)

Fe 0.00006100 -1.14800900 0.20778800

N -1.96569700 -1.07166300 -0.05527600

C -2.35888400 0.01951000 -0.75884700

C -4.18948800 -1.93845400 -0.13990600

H -4.88599100 -2.72431300 0.13093000

C -2.85882700 -2.03101200 0.25204700

H -2.48470200 -2.86543600 0.83244300

C -3.66926100 0.16818400 -1.19465900

H -3.94552800 1.05717200 -1.75202700

C -4.59849100 -0.82867300 -0.88348700

H -5.62848900 -0.73840300 -1.21450100

N -0.00004200 0.31719900 1.68972100

N -0.00002200 0.31186300 -1.13190700

C 0.00001700 -0.28553400 -2.50638300

H -0.88186000 -0.91228900 -2.63185500

H -0.00001100 0.51517900 -3.25324300

C -1.28309500 1.06661500 -0.92529000

H -1.47906000 1.68965600 -1.80194800

O -2.65318300 3.44874000 -0.96793000

H -3.32812800 4.14513700 -0.94651400

C -1.25646900 2.00119200 0.31828500

C -1.20304900 1.20645500 1.63268300

H -1.15247000 1.91174600 2.47051300

H -2.09076900 0.58899400 1.77131800

C -2.53106400 2.86805500 0.32237100

H -3.40676200 2.24865000 0.56740200

H -2.42171100 3.62439200 1.10807400

C -0.00017100 2.89939700 0.22202500

H -0.00019500 3.42839700 -0.73609600

O -0.00023900 3.80914800 1.31159900

C -0.00000600 -0.43516600 2.97693200

H -0.88562500 -1.07046200 3.02207500

N 1.96584900 -1.07145700 -0.05524700

C 2.35887500 0.01976200 -0.75884100

C 4.18976000 -1.93794500 -0.13993600

H 4.88637300 -2.72370900 0.13089000

C 2.85912400 -2.03067700 0.25206000

H 2.48513600 -2.86514900 0.83247500

C 3.66921900 0.16860400 -1.19470000

H 3.94534700 1.05762100 -1.75209100

C 4.59859400 -0.82812200 -0.88354600

H 5.62856900 -0.73772100 -1.21459400

H 0.88195300 -0.91221100 -2.63183700

C 1.28296700 1.06674800 -0.92527900

H 1.47887200 1.68981200 -1.80193400

O 2.65275600 3.44912600 -0.96784500

H 3.32768600 4.14553800 -0.94641600

C 1.25622400 2.00132700 0.31830000

C 1.20287000 1.20658800 1.63269700

H 1.15219400 1.91187600 2.47052400

H 2.09065500 0.58922600 1.77135200

C 2.53071000 2.86835200 0.32242500

H 3.40649000 2.24905800 0.56743400

H 2.42125900 3.62464000 1.10816400

H -0.00009200 4.71840800 0.97855100

H 0.88567400 -1.07037500 3.02207500

H -0.00004400 0.26152000 3.82263100

O 0.00025300 -2.80663900 1.25186100

C 0.00024500 -3.49014000 0.16143200

O 0.00013400 -2.77377100 -0.89786900

C 0.00028300 -4.97996100 0.13169000

H 0.87997500 -5.33430300 -0.41684000

H 0.00063500 -5.38517400 1.14470300

H -0.87985200 -5.33431000 -0.41614500

[(L^1^)Fe^V^=O]^2+^ (^4^P_IS_)

Fe -0.00876800 1.16800900 0.19139900

N 1.96836800 1.09866000 -0.04947500

C 2.36776600 -0.00440300 -0.73656800

C 4.19055700 1.96662800 -0.11784500

H 4.88375800 2.75714900 0.14778400

C 2.85615900 2.06228700 0.25996200

H 2.46854000 2.90220300 0.82353200

C 3.68375000 -0.15620900 -1.15315400

H 3.96887100 -1.05088200 -1.69680100

C 4.60726900 0.84732300 -0.84299400

H 5.64076800 0.75448700 -1.16239200

N 0.00328000 -0.42826400 1.77014700

N 0.00226500 -0.31428900 -1.12633300

C -0.00224400 0.29254300 -2.50121200

H 0.87574100 0.92540500 -2.62539700

H 0.00370700 -0.50936500 -3.24678500

C 1.29836500 -1.05762800 -0.91044500

H 1.50032800 -1.66770900 -1.79511100

O 2.68812700 -3.41316800 -1.02330900

H 3.36126900 -4.11168300 -1.02235800

C 1.27267600 -2.02402600 0.31409000

C 1.21740800 -1.28240000 1.66253700

H 1.20827800 -2.02490200 2.47124100

H 2.09420500 -0.64793300 1.80488200

C 2.55289300 -2.88366000 0.28815700

H 3.42322600 -2.26941600 0.56318400

H 2.44350100 -3.67120400 1.04216000

C 0.02191300 -2.92697600 0.18419800

H 0.02573800 -3.42091900 -0.79288300

O 0.02978000 -3.87676500 1.23841100

C -0.00227900 0.31813200 3.05354900

H 0.87917800 0.96169200 3.09884400

N -1.98471800 1.06906900 -0.04940300

C -2.36756200 -0.03972000 -0.73669900

C -4.21956500 1.90391400 -0.11799700

H -4.92447500 2.68401100 0.14763100

C -2.88678700 2.01933800 0.26002500

H -2.51177800 2.86486700 0.82373900

C -3.68106300 -0.21095000 -1.15359500

H -3.95278300 -1.10955300 -1.69761900

C -4.61946500 0.77868200 -0.84343400

H -5.65140100 0.67057500 -1.16308100

H -0.88952300 0.91231300 -2.62538100

C -1.28258900 -1.07690500 -0.91049500

H -1.47539700 -1.68998100 -1.79514600

O -2.63540900 -3.45513000 -1.02212200

H -3.30024400 -4.16156900 -1.02132100

C -1.24231200 -2.04270800 0.31409500

C -1.19797900 -1.30038100 1.66254600

H -1.17761000 -2.04276900 2.47114200

H -2.08413900 -0.67908900 1.80506000

C -2.50939800 -2.92147300 0.28854800

H -3.38913200 -2.32006800 0.56200000

H -2.38892100 -3.70605300 1.04403200

H 0.02869400 -4.77285700 0.87138000

H -0.89316900 0.94857200 3.09880700

H 0.00280000 -0.37005300 3.90810500

O -0.02141200 2.85401500 1.14993100

C -0.02726400 3.61754100 0.09052000

O -0.02301600 3.03862200 -1.02661400

C -0.03827700 5.10452100 0.25966900

H -0.91927400 5.40482600 0.83733100

H 0.83946900 5.41800700 0.83529700

H -0.04300300 5.59601700 -0.71392900

[(L^1^)Fe^V^=O]^2+^ (^6^P_HS_)

Fe -0.00750400 1.21953100 0.43812500

N 2.06195500 1.06537800 -0.01720000

C 2.38805100 -0.01738600 -0.76097600

C 4.28132200 1.91896100 -0.25283500

H 5.00133600 2.69998500 -0.03442000

C 2.98216800 2.01674500 0.23207300

H 2.64779700 2.85556800 0.83299900

C 3.66747100 -0.17894900 -1.27980800

H 3.89757400 -1.06518000 -1.86208200

C 4.62682200 0.80513700 -1.02306000

H 5.63136500 0.70497800 -1.42259200

N 0.00286800 -0.45964000 1.81939300

N 0.00172900 -0.28927400 -1.10879800

C -0.00219000 0.35339100 -2.45691700

H 0.87383100 0.99269200 -2.55784700

H 0.00247400 -0.41590900 -3.23777800

C 1.28436200 -1.04425200 -0.91443900

H 1.47127000 -1.65426700 -1.80347400

O 2.65978100 -3.42291500 -1.04605400

H 3.33870400 -4.11575200 -1.05177100

C 1.26759900 -2.02211400 0.30140300

C 1.22367600 -1.30708400 1.66472500

H 1.21908900 -2.06634000 2.45677100

H 2.09685400 -0.66996200 1.81360800

C 2.54801400 -2.88237000 0.26232700

H 3.42283500 -2.26523800 0.51657400

H 2.45198200 -3.66480800 1.02385900

C 0.01833100 -2.92451600 0.17127300

H 0.02169400 -3.41441400 -0.80711000

O 0.02543200 -3.87892300 1.22261400

C -0.00134200 0.21584400 3.14730100

H 0.88495100 0.85044700 3.23451400

N -2.07521200 1.03928500 -0.01695700

C -2.38771500 -0.04706400 -0.76132500

C -4.30484400 1.86561500 -0.25301200

H -5.03449600 2.63760300 -0.03445500

C -3.00718200 1.97907600 0.23250500

H -2.68334600 2.82154800 0.83407700

C -3.66479800 -0.22397300 -1.28088900

H -3.88379100 -1.11245600 -1.86402200

C -4.63628100 0.74814000 -1.02410100

H -5.63929900 0.63594300 -1.42426200

H -0.88592300 0.98200300 -2.55780800

C -1.27140200 -1.06020600 -0.91461900

H -1.45062900 -1.67258600 -1.80363300

O -2.61423700 -3.45941000 -1.04446000

H -3.28846400 -4.15683400 -1.05087000

C -1.24228600 -2.03761200 0.30127900

C -1.20716600 -1.32228000 1.66469200

H -1.19278700 -2.08165500 2.45648700

H -2.08829700 -0.69624100 1.81382200

C -2.51164100 -2.91392700 0.26257000

H -3.39461200 -2.30735300 0.51398200

H -2.40720100 -3.69305900 1.02652400

H 0.01809300 -4.77324400 0.85143300

H -0.89539600 0.83945400 3.23450300

H 0.00325700 -0.52200000 3.95853300

O -0.01983000 3.00073000 1.22049400

C -0.02364600 3.67781200 0.09053600

O -0.01878600 2.99822900 -0.96403700

C -0.03321500 5.16903200 0.12923700

H -0.91446600 5.51400300 0.68171200

H 0.84386300 5.52527400 0.68122300

H -0.03604600 5.57996400 -0.88092700

[(L^2^)Fe^V^=O]^2+^ (^2^P_LS_)

Fe -1.50437400 0.28938700 0.16687700

N -1.04499500 2.20350600 -0.06939800

C 0.09638400 2.38875100 -0.77788600

C -1.42210500 4.56152200 -0.04658200

H -2.03809300 5.39565900 0.27080700

C -1.79117600 3.26377800 0.29088200

H -2.67959900 3.04902900 0.87188100

C 0.51267000 3.65459500 -1.16457500

H 1.42813400 3.77248100 -1.73437700

C -0.25951400 4.75969000 -0.79351300

H 0.04664600 5.76086500 -1.08013000

N -0.07166800 -0.00920000 1.63735700

N -0.07562100 0.01429500 -1.19296200

C -0.67230900 0.14727200 -2.56146600

H -1.11518300 1.13645600 -2.66885000

H 0.10568700 0.00065300 -3.31834600

C 0.90995900 1.12850200 -0.97555100

H 1.56007700 1.21258900 -1.85236100

C 1.81084900 0.90232200 0.27246600

C 1.02962600 1.00222900 1.59198500

H 1.72099600 0.81744600 2.41983900

H 0.61259500 1.99564600 1.73702400

C 2.46354200 -0.48535500 0.15185100

H 2.96887000 -0.56420500 -0.81732100

O 3.37550800 -0.60865100 1.21874000

C -0.80571300 0.11948000 2.92978500

H -1.25737200 1.11048000 2.98833200

N -1.81299800 -1.64706800 -0.09951000

C -0.83951500 -2.24404500 -0.83158900

C -3.11240900 -3.64884700 -0.17536200

H -4.01293400 -4.17788000 0.11642400

C -2.92404700 -2.33215600 0.22873800

H -3.65050600 -1.80373300 0.83363300

C -0.97757900 -3.54550600 -1.29127800

H -0.18994000 -4.00068400 -1.88070900

C -2.13233500 -4.25931400 -0.95975700

H -2.25926200 -5.28105000 -1.30327200

H -1.46133000 -0.59267000 -2.68782200

C 0.40634600 -1.39834700 -0.99911500

H 0.97276700 -1.70625500 -1.88629900

C 1.33729100 -1.54113600 0.24800100

C 0.56729800 -1.35845300 1.56749600

H 1.27796800 -1.45039000 2.39612700

H -0.19145700 -2.12711500 1.69259800

H 4.01822800 -1.30144500 0.99485800

H -1.59685300 -0.63033600 2.96971900

H -0.11646800 -0.02274800 3.76922200

O -3.12487600 0.60839600 1.21333200

C 2.89397600 1.99921200 0.28989100

O 2.95704700 2.85261800 1.14209300

O 3.70047000 1.89095500 -0.77068800

C 1.89413300 -2.97850000 0.24545300

O 1.27121100 -3.92117900 0.67874900

O 3.10629600 -3.04708100 -0.31523300

C 3.70500500 -4.37718400 -0.38329700

H 4.67104100 -4.23018600 -0.86262200

H 3.81822000 -4.78080900 0.62456900

H 3.06801500 -5.03727600 -0.97492200

C 4.80538100 2.83885400 -0.82577800

H 5.44775900 2.70104700 0.04598200

H 5.33691600 2.60644900 -1.74685600

H 4.41994900 3.86055900 -0.83785500

C -3.79899200 0.75187900 0.12626400

O -3.09849500 0.61784600 -0.93562100

C -5.25975000 1.04280100 0.10409600

H -5.78537600 0.22656600 -0.40434100

H -5.64438900 1.15875500 1.11853500

H -5.44380600 1.95180300 -0.47858600

[(L^2^)Fe^V^=O]^2+^ (^4^P_IS_)

Fe 1.58947400 -0.05035200 0.18745400

N 1.40365500 -2.02091400 -0.01286500

C 0.28646000 -2.38359100 -0.70135100

C 2.12346800 -4.29536100 0.05865500

H 2.86070100 -5.02315600 0.37914600

C 2.30362300 -2.95039900 0.36153400

H 3.16086200 -2.58065800 0.91133300

C 0.05832700 -3.70736400 -1.04726000

H -0.83570500 -3.97616800 -1.59933800

C 0.99055300 -4.67714900 -0.66238000

H 0.82991000 -5.71893400 -0.92177100

N -0.10706000 0.02725200 1.76931500

N 0.07943600 -0.00018900 -1.15789400

C 0.68609400 -0.03596700 -2.53323300

H 1.28100900 -0.94196700 -2.64311000

H -0.11241300 -0.01435400 -3.28261000

C -0.71451600 -1.26782300 -0.93019400

H -1.31344400 -1.46611500 -1.82487400

C -1.69757100 -1.17735800 0.27775100

C -0.99700100 -1.15499400 1.64837300

H -1.76579400 -1.12540000 2.42876100

H -0.41302600 -2.06052500 1.80549400

C -2.55122300 0.09108700 0.10421800

H -3.00510500 0.08829300 -0.89354200

O -3.53283400 0.06716000 1.11418400

C 0.62472300 0.01483500 3.05943700

H 1.22927000 -0.89350900 3.11969700

N 1.55415100 1.92140800 -0.03956300

C 0.48034900 2.36149700 -0.75291800

C 2.50418200 4.11035200 -0.07157700

H 3.30999100 4.77424500 0.22138000

C 2.54186300 2.77186100 0.30081800

H 3.35440800 2.34320900 0.87502700

C 0.39924600 3.68011100 -1.17430300

H -0.45801800 4.01764900 -1.74477800

C 1.42514800 4.56630600 -0.83056900

H 1.37626200 5.60281900 -1.14912600

H 1.34797400 0.81969200 -2.66153500

C -0.61620500 1.32971300 -0.95138800

H -1.19412700 1.55555400 -1.85575700

C -1.61620400 1.31476300 0.25564300

C -0.91337700 1.26376000 1.62675900

H -1.68358000 1.28719200 2.40801200

H -0.27195600 2.13266800 1.76585100

H -4.26036800 0.65325800 0.84881300

H 1.28633000 0.88339300 3.10558200

H -0.07162400 0.04277200 3.90698600

O 3.35906000 -0.11902200 0.97916800

C -2.60034400 -2.42853000 0.24106300

O -2.57549300 -3.28721400 1.08956200

O -3.35819200 -2.44080300 -0.86091600

C -2.39880400 2.64282800 0.20294500

O -1.95928400 3.67780700 0.65014700

O -3.57657300 2.51054400 -0.41724700

C -4.37955800 3.72498000 -0.53132800

H -5.28496600 3.41950100 -1.05238500

H -4.60452300 4.11107800 0.46455600

H -3.83106900 4.47636000 -1.10239800

C -4.30344700 -3.54429300 -0.96759700

H -5.00386300 -3.50609700 -0.13121300

H -4.81498400 -3.39304200 -1.91647400

H -3.76903600 -4.49658100 -0.95314200

C 4.10277900 -0.15396000 -0.10567900

O 3.51500100 -0.13369900 -1.21049200

C 5.59013000 -0.21369600 0.05703900

H 5.93583500 0.66152400 0.61775800

H 5.86365300 -1.09662500 0.64460200

H 6.07413100 -0.24767100 -0.91971200

[(L^2^)Fe^V^=O]^2+^ (^6^P_HS_)

Fe 1.60635500 -0.13766900 0.40228900

N 1.23650100 -2.18051800 -0.03291200

C 0.12786600 -2.41071100 -0.77327400

C 1.82607700 -4.49238600 -0.16617300

H 2.51426900 -5.28901800 0.09422500

C 2.07294100 -3.19311200 0.26349900

H 2.94129300 -2.93305700 0.85914400

C -0.17550600 -3.68124400 -1.24437500

H -1.07009800 -3.84034900 -1.83714200

C 0.68630200 -4.73823800 -0.93484200

H 0.46999100 -5.74039500 -1.29162000

N -0.05573000 0.02774500 1.77863600

N 0.08765600 -0.00639200 -1.15951300

C 0.72861200 -0.07959800 -2.50656800

H 1.27840900 -1.01563000 -2.59485600

H -0.03531400 -0.01756800 -3.29073300

C -0.78293200 -1.21006300 -0.94894600

H -1.41656600 -1.34895100 -1.83220800

C -1.73439900 -1.08034600 0.28157800

C -1.01530100 -1.10768800 1.64312700

H -1.77173900 -1.03342800 2.43140500

H -0.48101900 -2.04383300 1.79168000

C -2.52324900 0.23197500 0.13621200

H -3.00352500 0.25686300 -0.84819000

O -3.47746200 0.26371500 1.17313000

C 0.62440000 -0.02068400 3.10441700

H 1.17058600 -0.96342200 3.19724700

N 1.61889100 1.92740200 -0.05792500

C 0.58675800 2.34714400 -0.82555300

C 2.68509300 4.05243800 -0.29434100

H 3.52337800 4.69937500 -0.06031200

C 2.64753500 2.75587900 0.20510900

H 3.43773400 2.34920700 0.82690700

C 0.56893100 3.62296700 -1.37216000

H -0.26485900 3.94052700 -1.98834300

C 1.63259500 4.48848400 -1.10202100

H 1.63756700 5.49134400 -1.51731700

H 1.44216600 0.73607000 -2.61429100

C -0.54386500 1.34317200 -0.97083600

H -1.13887200 1.58002100 -1.86289200

C -1.51376800 1.39621600 0.25936100

C -0.79596100 1.31245800 1.62129700

H -1.55554100 1.37399800 2.40952600

H -0.10601300 2.14354400 1.75274600

H -4.18088200 0.88501600 0.92334300

H 1.32810800 0.81269100 3.18195400

H -0.10848500 0.04996100 3.91680500

O 3.38381500 -0.30829800 1.16172400

C -2.69975900 -2.28387100 0.26148200

O -2.70054600 -3.14481000 1.10864300

O -3.48209100 -2.25327900 -0.82194200

C -2.21205600 2.77056000 0.22106600

O -1.69914700 3.77499200 0.65968600

O -3.40758800 2.71214100 -0.37555400

C -4.13203500 3.97545600 -0.47969900

H -5.06325600 3.72873500 -0.98622500

H -4.31639700 4.37642800 0.51868800

H -3.54483700 4.68935300 -1.06027500

C -4.47838200 -3.31199900 -0.91357200

H -5.15627900 -3.24998700 -0.06021800

H -5.00522900 -3.12970700 -1.84853600

H -3.98671100 -4.28711000 -0.91939000

C 4.04804500 -0.37483900 0.02477300

O 3.36078700 -0.30970300 -1.02250300

C 5.53204300 -0.51818800 0.05139800

H 5.96996200 0.32799700 0.59292900

H 5.80148300 -1.42213500 0.60918600

H 5.93215900 -0.56555900 -0.96197600
